# Nickel-catalyzed cross-electrophile allylation of vinyl bromides and the modification of anti-tumour natural medicine β-elemene†

**DOI:** 10.1039/d2sc02054h

**Published:** 2022-05-12

**Authors:** Yang Ye, Xiang Qi, Bing Xu, Ying Lin, Huan Xiang, Liang Zou, Xiang-Yang Ye, Tian Xie

**Affiliations:** School of Pharmacy, Hangzhou Normal University Hangzhou Zhejiang 311121 PR China yangye@hznu.edu.cn yeyang0711@163.com; Key Laboratory of Elemene Class Anti-Cancer Chinese Medicines, Engineering Laboratory of Development and Application of Traditional Chinese Medicines, Collaborative Innovation Center of Traditional Chinese Medicines of Zhejiang Province, Hangzhou Normal University Hangzhou Zhejiang 311121 PR China

## Abstract

Herein, we present a facile and efficient allylation method *via* Ni-catalyzed cross-electrophile coupling of readily available allylic acetates with a variety of substituted alkenyl bromides using zinc as the terminal reductant. This Ni-catalyzed modular approach displays excellent functional group tolerance and a broad substrate scope, which the creation of a series of 1,4-dienes including several structurally complex natural products and pharmaceutical motifs. Moreover, the coupling strategy has the potential to realize enantiomeric control. The practicality of this transformation is demonstrated through the potent modification of the naturally antitumor active molecule β-elemene.

## Abstract 2

Herein, we present a facile and efficient allylation method *via* Ni-catalyzed cross-electrophile coupling of readily available allylic acetates with a variety of substituted alkenyl bromides using zinc as the terminal reductant.

## Graphical abstract

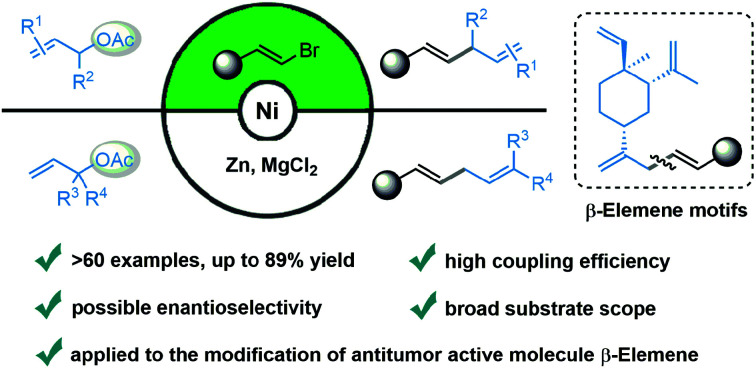

## Introduction

**Scheme 1:**
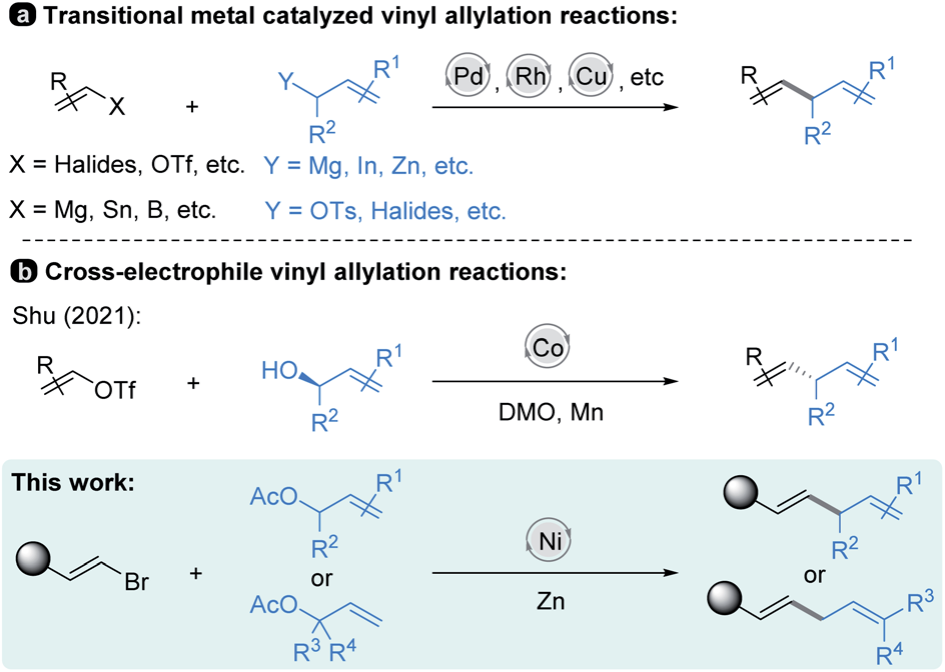
Allylation of vinyl reagents.

Allylic substitution reactions have been the subject of intense study,^1^ finding broad applications in the synthesis of natural products.^2^ In recent decades, vinyl allylation has been generally achieved *via* transition-metal-catalyzed cross-coupling of vinyl electrophiles with allyl-metallic reagents.^3^ And the scope of vinyl coupling partners was expanded to organomagnesium, organotin, organoboron reagents and so on, which are coupled with allylic electrophiles to realize the allylation reactions^4^ (Scheme 1, top). Studies in this field have resulted in numerous well-established synthetic methods by using Pd, Rh, Cu and Ni catalysts;^3–6^ in particular, asymmetric versions have been developed.^7^

On the other hand, reductive cross-coupling of two electrophiles^8,9^ including allylation of vinyl electrophiles with allylic electrophiles affords an alternative choice by its facile carbon–carbon bond formation without pre-preparation of organometallic nucleophiles.^10^ For instance, Shu and co-workers recently reported a Co-catalyzed cross-electrophile protocol in which a variety of substituted chiral allylic alcohols undergo highly efficient coupling with mainly vinyl triflates (Scheme 1, middle).^11^ In this process, alcohols are activated *in situ* by dimethyl oxalate (DMO)^12^ through transesterification, and the benzyl oxalates formed participate in the coupling as they are generated.

Although recent years have seen significant progress in advancing the transition-metal-catalyzed vinyl allylation reactions, several challenges remain to be addressed. For example, most of the reactions are limited to the cross-coupling strategy of electrophiles with nucleophilic reagents. In addition, the present methods still suffer from a limited substrate scope and lack of further exploration for the practicality of these transformations. And no examples of the corresponding Ni-catalyzed reductive cross-coupling of vinyl electrophiles^13^ with allylic electrophiles have been reported. Therefore, systematic studies on the transition-metal-catalyzed coupling of substituted vinyl electrophiles with allylic electrophiles are still in need.

In this paper, we have explored a Ni-catalyzed reductive coupling of various substituted allylic acetates with a series of (*E*)-alkenyl bromides that efficiently generates 1,4-dienes at mild temperatures (Scheme 1, bottom). The use of the nickel catalyst, zinc and bipyridine ligand L3 (Table 1) is critical in the present study. Structurally complex substrates derived from natural products and approved drugs are well tolerated. More specifically, the vinylation of β-elemene at site 13 could enhance its anti-tumour activity successfully. In addition, we disclose the asymmetric version of the allylic vinylation through the coupling of two electrophiles, although the coupling efficiency and enantioselectivity are still low. And the preliminary mechanistic studies are also discussed.

**Table 1:** Optimization of the reaction conditions

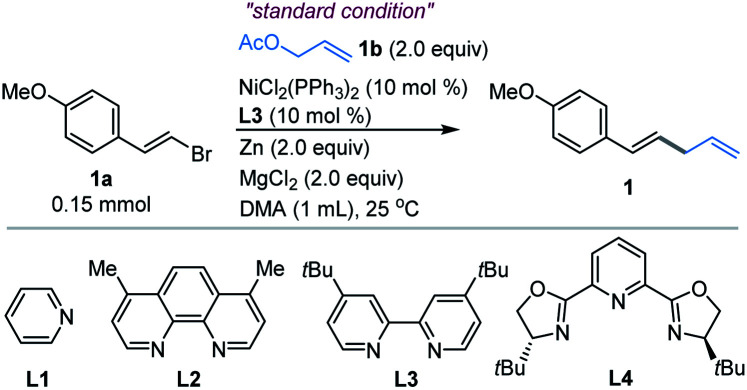		
Entry	Variation from standard conditions	Yield [%]^a^
1	None	96 (85^b^)
2	w/o NiCl_2_(PPh)_3_	Trace
3	w/o Zn	No reaction
4	w/o L3	Trace
5	w/o MgCl_2_	14
6	NiCl_2_ instead of NiCl_2_(PPh)_3_	64
7	Ni(COD)_2_ instead of NiCl_2_(PPh)_3_	17
8	Co(acac)_3_ instead of NiCl_2_(PPh)_3_	No reaction
9	Mn instead of Zn	68
10	TDAE instead of Zn	88
11	L1 instead of L3	48
12	L2 instead of L3	33
13	L4 instead of L3	56
14	PPh_3_ instead of L4	34
15	5.0 mmol of 1a	72^b^

a NMR yield using 2,5-dimethyl furan as the internal standard from a mixture containing other impurities after a quick flash column chromatography.

b Isolated yield (average of 2 independent runs).

## Results and discussion

### Optimization of the reaction conditions

We first examined the coupling of (*E*)-1-(2-bromovinyl)-4-methoxybenzene 1a with allyl acetate 1b.^14^ The reported Ni-catalyzed conditions for allylation of aryl halides in *N*,*N*-dimethylacetamide (DMA) gave 1 in <15% yield.^15^ An extensive survey of the reaction conditions led us to identify that a combination of NiCl_2_(PPh_3_)_2_ (10 mol%), 4,4′-di-*tert*-butyl-2,2′-bipyridine (L3, 10 mol%) with Zn and MgCl_2_ in DMA at ambient temperature (25 °C) provided the coupling product 1 in an optimal 85% isolated yield (Table 1, entry 1). The control experiments indicated the necessity of Ni, Zn and ligand L3 (entries 2–4). However, without MgCl_2_, a significant decrease of the yield was observed (entry 5). According to the literature,^16^ we speculated that the role of MgCl_2_ is possibly to activate zinc powder by removal of salts on its surface, while other nickel sources such as NiCl_2_ and Ni(COD)_2_ were not satisfactory (entries 6–7, and Table S1†). And Co(acac)_3_ could not trigger this reaction, which is distinguished from Shu's work^11^ (entry 8). By contrast, copper salt and iron salt were unreactive (Table S1, entries 15–16†). The use of Mn and TDAE to replace Zn also generated 1 in 68% and 88% yields, respectively (entry 9–10). Replacement of L3 with other pyridine-containing additives did not improve the results (entries 11–13, and Table S2†). The ligand of PPh_3_ proved to be unsuitable (entry 14). The reactions at 40 °C and 60 °C led to comparable yields, while somewhat lower yield was obtained at 0 °C (Table S3, entries 8–10†). Finally, the reaction on a gram scale gave 1 in 72% isolated yield (entry 15).

### Substrate scope

**Fig. 1:**
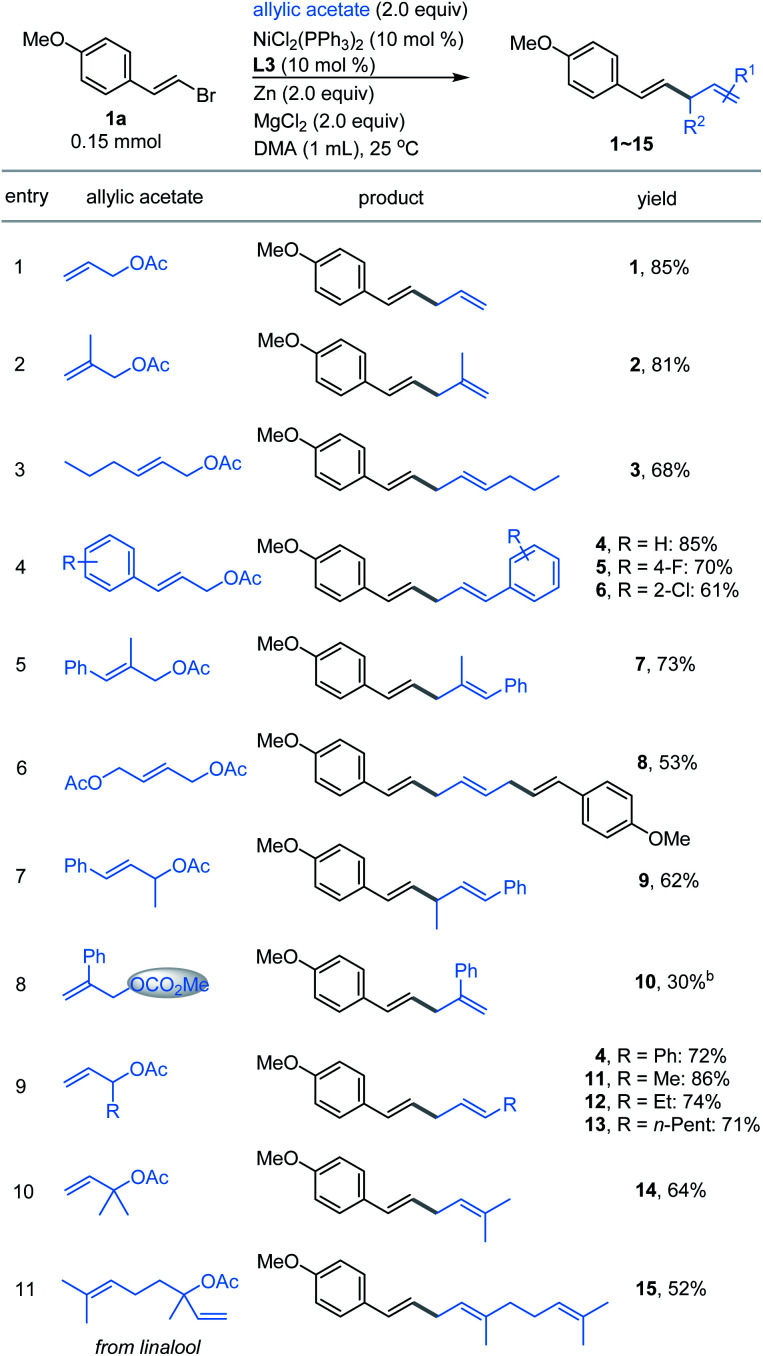
Scope of allylic acetates. ^*a*^The standard reaction conditions; isolated yields are provided (average of 2 independent runs). ^*b*^NMR yield using 2,5-dimethyl furan as the internal reference.

With the optimized conditions in hand, the allylated products 1–15 were produced in fairly good yields by the cross-coupling of (*E*)-1-(2-bromovinyl)-4-methoxybenzene 1a with a broad range of substituted allylic acetates (Fig. 1). The 1,4-dienes were effectively generated (*e.g.*, 1–8) when using the primary allylic acetates. 81% yield was obtained for 2-substituted allylic acetate 2. The reaction also exhibited good coupling efficiency for 3-substituted allylic acetates within alkyl or aryl groups, as evident in 3–6 including F- and Cl-membered aryl ones. Moreover, (*E*)-2-methyl-3-phenylallyl acetate gave product 7 in 73% yield. The simultaneous vinylation of the diacetate was successful as exemplified by 8. However, coupling of the sterically more hindered 1-methyl-3-phenyl-allylic acetate with 1a generated product 9 in moderate yield. Finally, the allylic carbonate (10) was proved to be compatible under these reaction conditions, although a low yield was observed. Next, exposure of the 1-substituted allylic acetates to the same conditions delivered the stereoinverted products (11–15) in moderate to excellent yields (Fig. 1, bottom). These secondary and tertiary allylic acetates were tolerated well with a range of functional alkyl or unsaturated groups including methyl, *n*-pentyl and phenyl. The notable one includes linalyl acetate generated 15 in moderate yield, which derived from linalool. These results indicate that the reactions proceed through a similar (*η*^3^-π-allyl) nickel intermediate.^15^

**Fig. 2:**
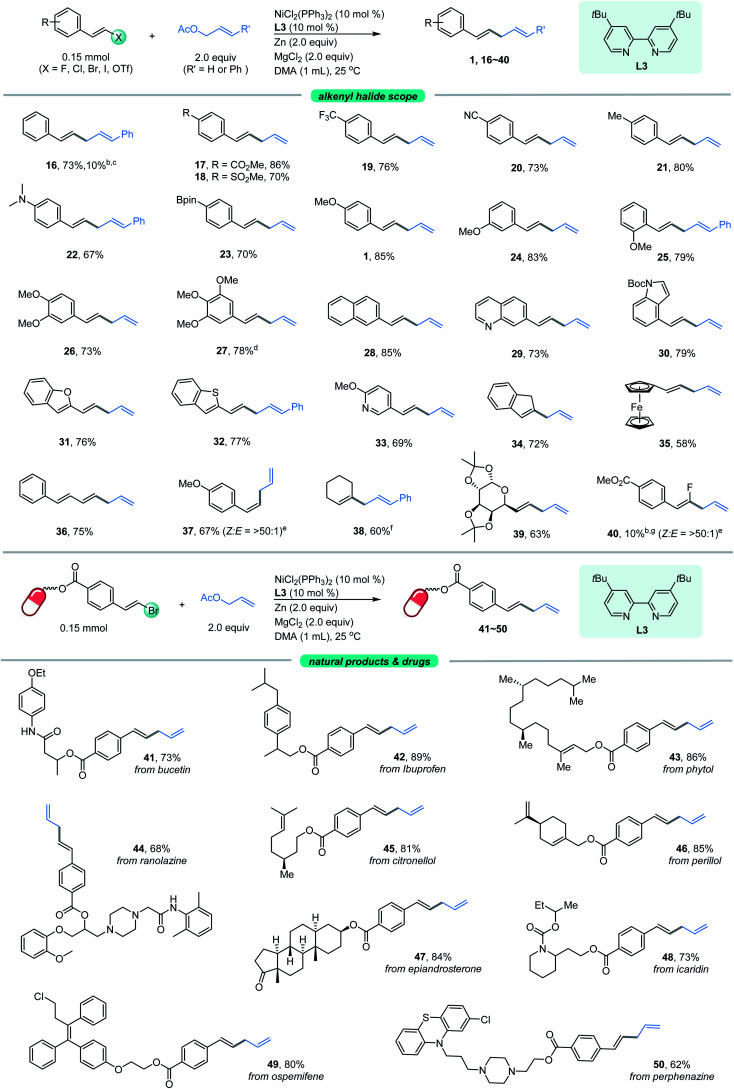
The scope of alkenyl halides. ^*a*^The standard reaction conditions; the isolated yields are provided (average of 2 independent runs). ^*b*^NMR yield using 2,5-dimethyl furan as the internal reference. ^*c*^(*E*)-(2-Chlorovinyl)benzene instead of alkenyl bromide. ^*d*^(*E*)-5-(2-Iodovinyl)-1,2,3-trimethoxybenzene instead of alkenyl bromide. ^*e*^The *E*/*Z* ratio was determined by ^1^H NMR. ^*f*^Cyclohex-1-en-1-yl trifluoromethanesulfonate instead of alkenyl bromide. ^*g*^Methyl 4-(2,2-difluorovinyl)benzoate instead of alkenyl bromide.

Our attention was then shifted to the scope of the alkenyl halide partner. As shown in Fig. 2, a wide range of β-aryl-substituted (*E*)-alkenyl bromides bearing electron-deficient substituents such as ester, trifluoromethyl and cyano (17–20) or electron-rich substituents such as methyl, *N*,*N*-dimethyl, Bpin and methoxy (21–27) on the arene afforded the desired products smoothly. The electronic properties of alkenyl halides did not show an obvious effect on the efficiency of this transformation. Among the methoxy-substituted products (1, 24–25), the *para*-one was the most effective. Signally, the β-aryl-substituted (*E*)-alkenyl bromides bearing multi-substituents on the aromatic ring (26–27) underwent this allylation smoothly with high levels of transformation. Emphatically, (*E*)-alkenyl chloride (16) and (*E*)-alkenyl iodide (27) were shown to participate in the reaction to provide low to high yields. In addition, β-naphthyl-substituted (*E*)-alkenyl bromide (28) was also proved to be compatible. Good coupling results with excellent chemoselectivities were also observed for β-heteroaromatic-substituted (*E*)-alkenyl bromides, containing various functional groups or moieties such as quinoline (29), indole (30), benzofuran (31), thianaphthene (32) or pyridine (33). Notably, α-alkyl substituted alkenyl bromide, such as 2-bromoindene (34), is also a competent coupling partner. Ferrocene alkenyl bromide (35) could also undergo the reaction, providing access to good stereo-controlled ferrocene derivatives. However, a moderate yield was observed when 1,3-dienyl bromide (36) was used as the substrate. Notably, (*Z*)-alkenyl bromide (37) is a competent coupling partner, while vinyl triflates such as cyclohexenyl triflate (38) is also an active substrate with 60% yield, which indicated that the Ni-catalyzed conditions seem to be more effective than the analogous Co- or Fe-catalyzed coupling methods.^10*a*–*c*,16*a*^ β-Alkyl-substituted (*E*)-alkenyl bromides, such as those containing a structurally complex galactose derivative (39), were also shown to be viable substrates with a slight decrease in yield. Moreover, 2,2-difluorovinyl benzene afforded the *cis*-product 40 in low yield.

To further expand the practicality of this tactic, we attached the alkenyl bromide moiety to a variety of more structurally complex drugs and natural products (Fig. 2, bottom). Remarkably, all of these substrates examined in our work underwent the transformation in good to excellent yields (41–50). The reaction system is capable of distinguishing the activated alkenyl bromides from the unactivated ones,^14^ providing the site specific transformation. The alkenyl bromide derivatives from pharmaceuticals such as the analgesic-antipyretic bucetin (41) and ibuprofen (42), antianginal drug ranolazine (44), hormone drug epiandrosterone (47), repellent icaridin (48), selective estrogen receptor modulator ospemifene (49) and psychiatric drug perphenazine (50) were amenable to this reaction, demonstrating the viability of this nickel-catalyzed allylation for modifications of bioactive molecules. Furthermore, derivatives from naturally occurring alcohols such as phytol (43), citronellol (45) and perillol (46) were also competent substrates, enabling access to the desired adducts in synthetically useful yields. It is worth noting that many of these substrates exhibit extremely high transformation, up to 89% yield.

**Fig. 3:**
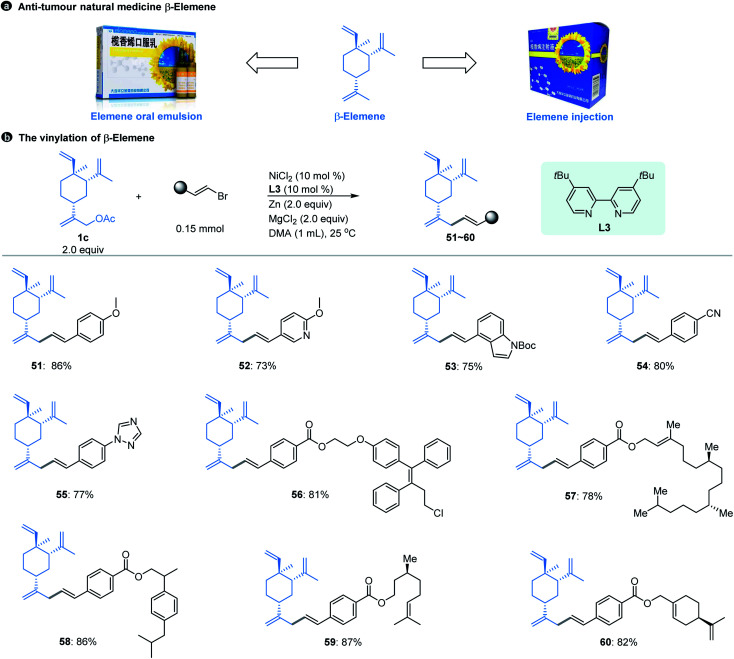
β-Elemene and its vinyl modification. ^*a*^The standard reaction conditions; isolated yields are provided (average of 2 independent runs).

Natural products have always been a significant direct or indirect source of anti-tumour drugs. β-Elemene is an anti-tumour natural medicine, which is a sesquiterpene exacted from the rhizome of Traditional Chinese Medicine (TCM) *Curcuma wenyujin*.^21^ After years of experimentation and efforts,^22^ elemene oral emulsion (CFDA number H20010338) and elemene injection (CFDA number H10960114) developed by our research team were approved by the China Food and Drug Administration (CFDA) as broad-spectrum anti-tumour drugs in the 1990s (Fig. 3a). Although β-elemene has achieved good clinical effects and feedback, its structure still has certain defects. For example, β-elemene is a volatile oil that is insoluble in water and can only be formulated in liposome formats, and its anti-tumour activity is moderate. Therefore, it is necessary to carry out purposeful structural modifications to improve its physical and chemical properties and enhance its anti-tumour effect.

However, the majority of the modifications of β-elemene reported previously are limited to the nucleophilic displacement of amino, hydroxyl or thiol groups at the allylic position due to the chemistry feasibility.^23^ Using the Ni-catalyzed cross-electrophile coupling method developed here, we realize for the first time the construction of a Csp^3^–Csp^2^ bond at the allylic position of β-elemene that was achieved by using the substrates of pre-acetylated β-elemene derivatives, which allows the diverse structures of β-elemene analogs to be constructed in a feasible manner. As shown in Fig. 3b, our allylic vinylation method displayed excellent compatibility for various vinyl bromides when coupling with β-elemene derived-acetate 1c. The reactions were also compatible with 1-aryl-conjugated vinyl bromides. The aryl moieties bearing a variety of functional groups in general resulted in good coupling yields (51–55). Moreover, the conversion of 1c with a series of structurally complex drug or natural product conjugated vinyl bromides was efficient as manifested by the examples of 56–60. Notably, our vinylation strategy of β-elemene at site 13 could be a potential solution to enhance its hydrophilicity by introducing the groups such as methoxy (51, 52), amino (53), triazole (55), ester (56–60), *etc*.

With the β-elemene vinylated compounds in hand, we subsequently investigated the *in vitro* anti-tumour activities of these compounds towards HCT116 (colon cancer cells) and A549 (lung cancer cells) tumour cell lines.^24^ After the first preliminary screening at a concentration of 50 μM, compound 55 was selected to further test its IC50 values against these two tumour cell lines. For the HCT116 cell line, compound 55 is over 15-fold more active than β-elemene (IC50: 50.9 μM and 781.9 μM for compound 55 and β-elemene, respectively, Fig. S1–S2†); for the A549 cell line, the activity of compound 55 is about 4-fold greater than that of β-elemene (IC50: 57.2 μM and 227.0 μM for compound 55 and β-elemene, respectively, Fig. S3–S4†). The above results indicate that the anti-proliferative activities of compound 55 are absolutely more potent than those of β-elemene, demonstrating that the introduction of the vinyl motif has successfully enhanced the anti-tumour effect of β-elemene, and our cross-coupling strategy is feasible for the structural modification of β-elemene in further investigation.

**Fig. 4:**
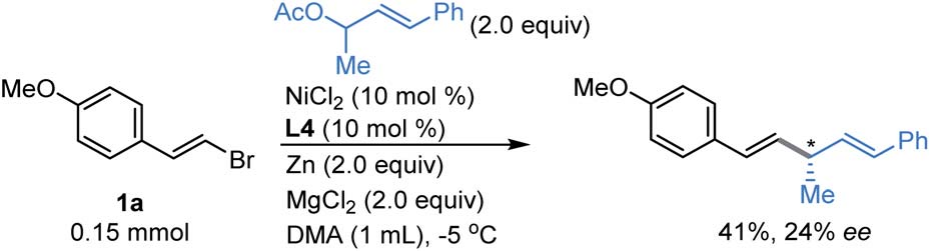
Asymmetric vinylation of 1,3-disubstituted allyl acetate.

Next, we investigated whether the allylation strategy was possible to achieve enantioselectivity for the coupling of vinyl bromide 1a with (*E*)-4-phenylbut-3-en-2-yl acetate using a chiral tridentate pybox ligand L4.^14^ Although a moderate yield was obtained, only 24% of enantiomeric excess was observed (Fig. 4). Efforts will be continued in our lab to promote the enantioselectivity of this direct reductive coupling of vinyl bromides with allylic electrophiles.

### Mechanistic consideration

**Fig. 5:**
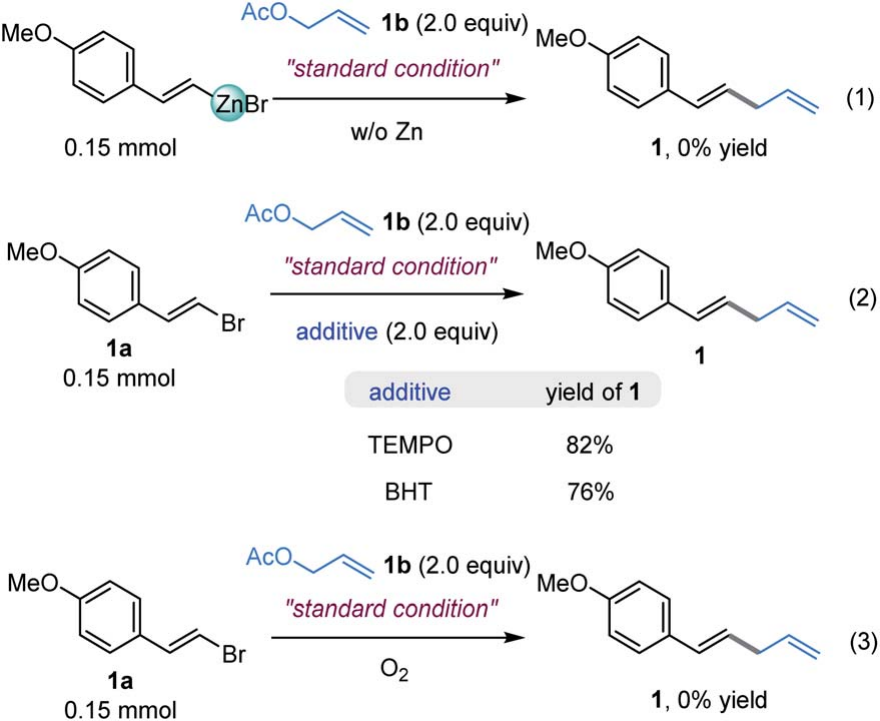
Mechanistic investigation and competition experiments.

In order to elucidate the underlying mechanism of this reaction, the assembly of an organozinc reagent prepared from (*E*)-1-(2-bromovinyl)-4-methoxybenzene 1a with allyl acetate 1b was performed in the absence of zinc powder using the standard method to examine whether an *in situ* Negishi process is possible. However, the corresponding allylation product was not obtained (Fig. 5, eqn (1)).^14^ In addition, coupling of vinyl bromide 1a with allyl acetate 1b as in Table 1, entry 4, but in the absence of a ligand mainly gave recovered starting materials.^14^ These results suggest that *in situ* formation of the organozinc/Negishi process does not seem to occur. To gain insight into the reaction details, a set of control experiments were executed (Fig. 5, eqn (2)). Considering the radical mechanism proposed by Bandar,^17*a*^ radical scavengers were added into the reaction of 1a and 2a. As a result, the yield of 1 was hardly affected with either TEMPO or BHT, thus precluding the engagement of a SET-initiated radical process.^17*b*^ Moreover, when the reaction was carried out under aerobic conditions, it did not work (Fig. 5, eqn (3)). These data demonstrated that the reaction was sensitive to the O_2_.

**Scheme 2:**
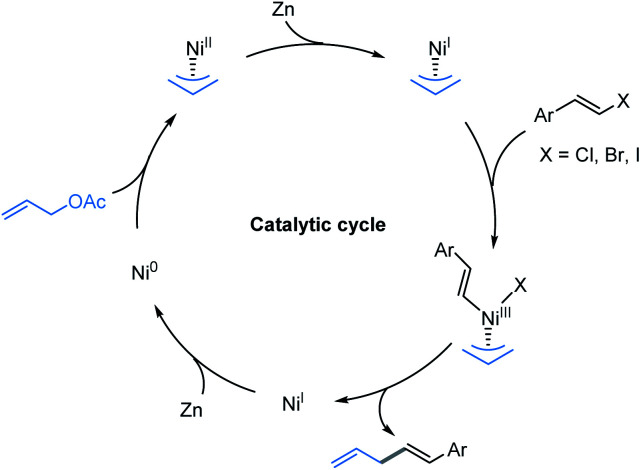
Proposed catalytic pathway.

On the basis of the results described above and the stoichiometric studies of allylic-Ni(i) with electrophiles reported by Corey, Heghdus and Gong,^18^ a Ni(i) to Ni(iii) process may account for the catalytic pathways in the present study.^19^ Moreover, similar to the Co-catalyzed reductive allylation in Shu’s previous studies,^11^ we tentatively propose a catalytic cycle as shown in Scheme 2. First, the oxidative addition of allyl acetate to Ni(0) affords the π-allyl-Ni(ii) complex. Then one electron reduction of the π-allyl-Ni(ii) generates an allyl-Ni(i) intermediate by Zn.^20^ This intermediate undergoes the oxidative addition process with alkenyl halide which results in the allyl-Ni(iii)-alkenyl species. Subsequent reductive elimination produces the desired product and generates a Ni(i) intermediate, which can be reduced to Ni(0) by Zn.

## Conclusions

In conclusion, we have reported an efficient Ni-catalyzed method for the allylation of alkenyl bromides to afford 1,4-dienes. The reaction demonstrates excellent functional group tolerance and a broad substrate scope for a variety of substituted substrates including a series of alkenyl bromide derived natural products and drugs. Significantly, this work provides a convenient tool for modification of bioactive β-elemene by facile introduction of alkenyl subunits, supporting the conclusion that this synthetic method can be a strong impetus for explorations in medicinal chemistry and chemical biology. In addition, although a low *ee* was observed for the 1,3-disubstituted allylic acetate, it represents the rare asymmetric version of direct reductive coupling of two electrophiles wherein *in situ* organometallic reagents may not be involved. Efforts to further apply this reaction, the Ni-catalyzed asymmetric cross-electrophile allylation version and further biological evaluations of synthetic products are currently in progress in our laboratory.

## Data availability

All experimental data and detailed procedures are available in the ESI.†

## Author contributions

Y. Y. designed the research, performed the experiments, analyzed the data, and wrote the paper. X. Q. performed the *in vitro* anti-tumour activity experiments and synthesized some of the allylic acetates. B. X., Y. L., H. X. and L. Z. synthesized some of the vinyl halides. X.-Y. Y. and T. X. revised the paper.

## Conflicts of interest

There are no conflicts to declare.

## Supplementary Material

SC-013-D2SC02054H-s001

## References

[cit1] Li C., Xing J., Zhao J., Huynh P., Zhang W., Jiang P., Zhang Y. J. (2012). Org. Lett..

[cit2] Trost B. M., Crawley M. L. (2003). Chem. Rev..

[cit3] Krasovskiy A. L., Haley S., Voigtritter K., Lipshutz B. H. (2014). Org. Lett..

[cit4] Alacid E., Najera C. (2009). J. Org. Chem..

[cit5] Naofumi T., Tetsuo S., Yoshio I. (2001). Chem. Commun..

[cit6] Cherney A. H., Kadunce N. T., Reisman S. E. (2015). Chem. Rev..

[cit7] Lu Z., Ma S. (2008). Angew. Chem., Int. Ed..

[cit8] Min Y., Ma G., Wang X., Gong H. (2021). Angew. Chem., Int. Ed..

[cit9] Liu J., Ye Y., Sessler J. L., Gong H. (2020). Acc. Chem. Res..

[cit10] Gomes P., Gosmini C., Périchon J. (2003). Org. Lett..

[cit11] Ma W.-Y., Han G.-Y., Kang S., Pang X., Liu X.-Y., Shu X.-Z. (2021). J. Am. Chem. Soc..

[cit12] Guo P., Wang K., Jin W.-J., Xie H., Qi L., Liu X.-Y., Shu X.-Z. (2021). J. Am. Chem. Soc..

[cit13] Ye Y., Liu J., Xu B., Jiang S., Bai R., Li S., Xie T., Ye X.-Y. (2021). Chem. Sci..

[cit14] See the ESI† for details

[cit15] Cui X., Wang S., Zhang Y., Deng W., Qian Q., Gong H. (2013). Org. Biomol. Chem..

[cit16] Wang S., Qian Q., Gong H. (2012). Org. Lett..

[cit17] Luo C., Bandar J. S. (2019). J. Am. Chem. Soc..

[cit18] Hegedus L. S., Thompson D. H. P. (1985). J. Am. Chem. Soc..

[cit19] Tsou T. T., Kochi J. K. (1979). J. Am. Chem. Soc..

[cit20] Ikeda S., Suzuki K., Odashima K. (2006). Chem. Commun..

[cit21] Zhai B., Zhang N., Han X., Li Q., Zhang M., Chen X., Li G., Zhang R., Chen P., Wang W., Li C., Xiang Y., Liu S., Duan T., Lou J., Xie T., Sui X. (2019). Biomed. Pharmacother..

[cit22] Wang X., Liu Z., Sui X., Wu Q., Wang J., Xu C. (2019). Phytomedicine.

[cit23] Liu G., Kong Z., Shen Y. (2013). Med. Chem. Res..

[cit24] Bai R., Zhu J., Bai Z., Mao Q., Zhang Y., Hui Z., Luo X., Ye X.-Y., Xie T. (2022). J. Enzyme Inhib. Med. Chem..

