# Supplementary material for: Nickel-catalyzed cross-electrophile allylation of vinyl bromides and the modification of anti-tumour natural medicine β-elemene
Source: Chem Sci. 2022 May 12;13(23):6959–66. doi: 10.1039/d2sc02054h (PMC9200125; doi:10.1039/d2sc02054h)
Supplement: SC-013-D2SC02054H-s001 [file SC-013-D2SC02054H-s001.pdf]

Electronic Supplementary Material (ESI) for Chemical Science.
This journal is © The Royal Society of Chemistry 2022

Supporting Information

Nickel-Catalyzed Cross-Electrophile Allylation of Vinyl Bromides and The Modification of Anti-Tumour Natural Medicine β-Elemene

Yang Ye,*[a,b,†] Xiang Qi,[a,b,†] Bing Xu,[a,b] Ying Lin,[a,b] Huan Xiang,[a,b] Ling, Zou,[a,b] Xiang-Yang Ye*[a,b] and Tian Xie*[a,b]

[a]School of Pharmacy, Hangzhou Normal University, Hangzhou, Zhejiang 311121, PR China

[b]Key Laboratory of Elemene Class Anti-Cancer Chinese Medicines; Engineering Laboratory of Development and Application of Traditional Chinese Medicines; Collaborative Innovation Center of Traditional Chinese Medicines of Zhejiang Province, Hangzhou Normal University, Hangzhou, Zhejiang 311121, China

yangye@hznu.edu.cn; yeyang0711@163.com

Table of Contents

I. General Information……………………………..…………….…S2

II. Details of Optimization...S3-S7

III. Cross-Coupling of Allylic Acetates with Vinyl Bromides…..…......…S7-S31

IV. Vinylation of *β*-Elemene…...S31-S37

V. In Vitro Anti-Tumour Activity of Compound 55..............................S38-S39

VI. Preparation of Alkenyl Bromides ………………...………..........…S39-S42

VII. Preparation of β-Elemene Derived-Acetate 1c...................................S42-S43

VIII. Mechanistic Investigations S44

IX. References…………………………………………………….........S45

X. Spectroscopic Data (NMR Spectrum and HPLC Trace) ……......….S46-S108

I. General Information

1. Chemicals and Reagents

All manipulations were carried out under an atmosphere of nitrogen using standard Schlenk or glove box techniques. DMA (*N,N*-dimethylacetamide, 99.5%, extra dry, Acros) was purchased and used directly. Deuterated solvents were used as received ($CDCl_3$ from Maclin Co., China). NiF_2 (Alfa Aesar), $NiCl_2$ (Alfa Aesar), $NiBr_2$ (Alfa Aesar), NiI_2 (Alfa Aesar), $Ni(COD)_2$ (Strem), $Ni(OTf)_2$ (Alfa Aesar), $Ni(acac)_2$ (Maclin Co., China), $NiCl_2(PPh)_3$ (Alfa Aesar), $Co(acac)_2$ (Alfa Aesar), $Cu(acac)_2$ (Alfa Aesar) were used as received. Zinc powder (Aladdin) was activated with hydrochloric acid before use. Anhydrous $MgCl_2$ (Alfa Aesar) were purchased and used directly. 4,4'-Di-tert-butyl-2,2'-bipyridine (>99%, Alfa Aesar) was purchased and used directly. Procedures for the synthesis of the vinyl bromides used in this study have been reported in our previous publications.[1] Unless otherwise noted, all other reagents and starting materials were purchased from commercial sources and used without further purification.

2. Physical Methods

Column chromatography was performed using silica gel 200-300 mesh (purchased from Qingdao-Haiyang Co., China) as the solid support. All NMR spectra were recorded on Bruker Avance 500 MHz spectrometers. ^{1}H NMR and ^{13}C NMR chemical shifts are reported in δ units, parts per million (ppm) relative to the chemical shift of residual solvent. Reference peaks for chloroform in ^{1}H NMR and ^{13}C NMR spectra were set at 7.26 ppm and 77.16 ppm, respectively. High-resolution mass spectra (HRMS) were obtained using a Bruker APEXIII 7.0 or IonSpec 4.7 TESLA FTMS instruments. Melting points were recorded on a micro melting point apparatus (X-4, YUHUA Co., Ltd, Gongyi, China). GC chromatograms were recorded on a GCMS-QP2010 SE (SHIMADZU) using an Agilent column CP7502 and Rxi-5 ms (Restek). Ultra Fast liquid chromatography was performed on Shimadzu Chromatographs (LC-2030 Plus) using Daicel Chiralcel columns (250 mm).

II. Details of Optimization

1. Reaction Conditions Optimization

Table S1. Screening the catalyst for the reaction of **1a** with **1b**.

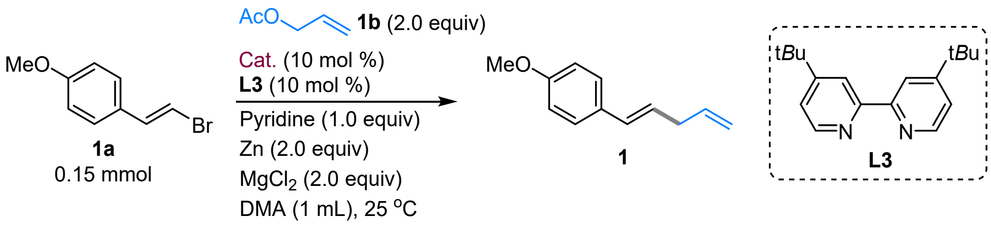

entry	Cat.	yield%[a]
1	$NiCl_2$•DME	40
2	$NiBr_2$•DME	33
3	$Ni(acac)_2$	46
4	$Ni(COD)_2$	17
5	NiI_2	43
6	$NiBr_2$	27
7	$NiCl_2$	64
8	NiF_2	24
9	$Ni(TMHD)_2$	44
10	$Ni(ClO_4)_2$•4(py)	36
11	$NiCl_2(PPh_3)_2$	79
12	$Ni(OTf)_2$	29
13	$Ni(ClO_4)_2$•$6H_2O$	34
14	$Co(acac)_2$	no reaction
15	$Cu(acac)_2$	no reaction
16	$Fe(acac)_3$	no reaction

[a]NMR yield using 2,5-dimethyl furan as the internal standard from a mixture containing other impurities after a quick flash column chromatography.

Table S2. Screening the ligand for the reaction of **1a** with **1b**.

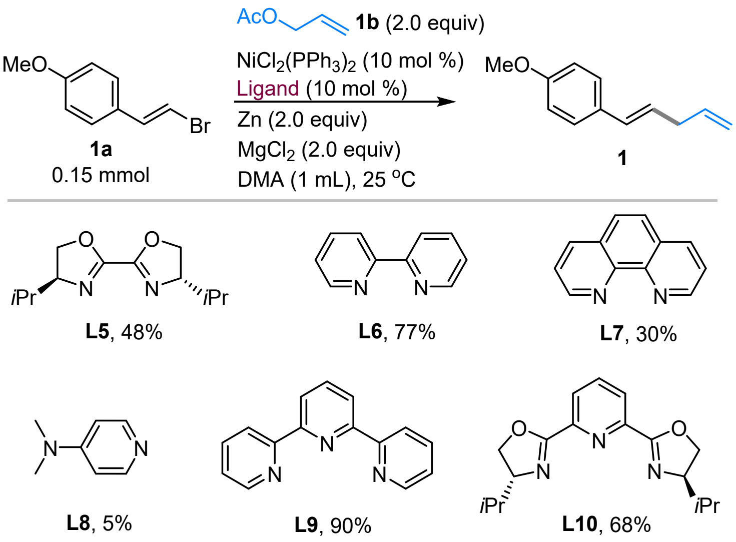

[a]NMR yield using 2,5-dimethyl furan as the internal standard from a mixture containing other impurities after a quick flash column chromatography; [b]Not detected.

Table S3. Screening the variations for the reaction of **1a** with **1b**.

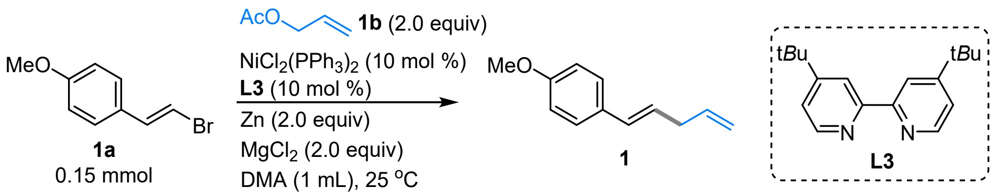

entry	Variations	yield%[a]
1	none	96
2	**1a** : **1b** = 1.0 : 1.5	26
3	**1a** : **1b** = 1.0 : 1.2	38
4	**1a** : **1b** = 1.0 : 1.0	trace
5	**1a** : **1b** = 1.2 : 1.0	64
6	**1a** : **1b** = 1.5 : 1.0	86
7	**1a** : **1b** = 2.0 : 1.0	79
8	0°C	59
9	40°C	76
10	60°C	65
11	Mn instead of Zn	68
12	TDAE instead of Zn	88
13	$NiCl_2$ (10 mol %) and PPh_3 (20 mol %) instead of $NiCl_2(PPh_3)_2$ (10 mol %)	42

[a]NMR yield using 2,5-dimethyl furan as the internal standard from a mixture containing other impurities after a quick flash column chromatography.

Table S4. Screening the chiral ligands for the Ni-catalyzed enantioselective reaction of (*E*)-4-phenylbut-3-en-2-yl acetate with **1a**.

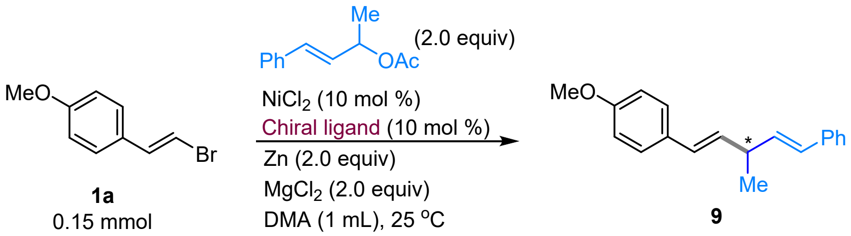

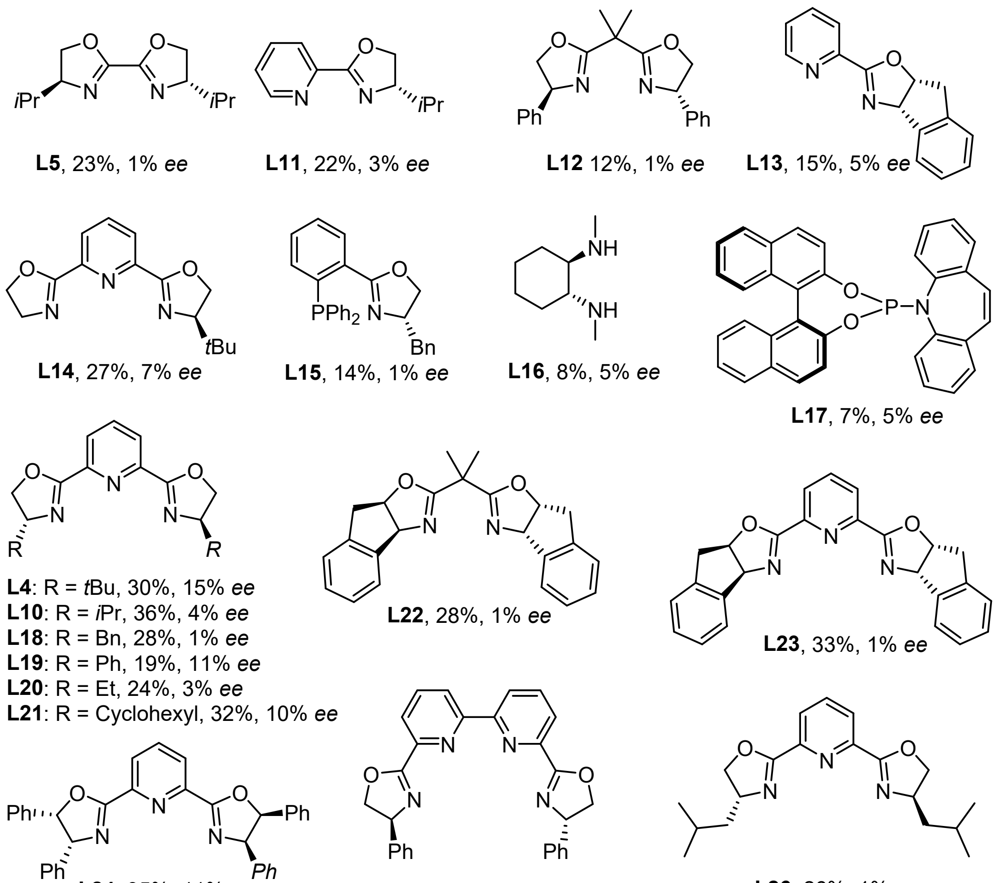

L5, 23%, 1% *ee*
L11, 22%, 3% *ee*
L12 12%, 1% *ee*
L13, 15%, 5% *ee*
L14, 27%, 7% *ee*
L15, 14%, 1% *ee*
L16, 8%, 5% *ee*
L17, 7%, 5% *ee*
L4: R = *t*Bu, 30%, 15% *ee*
L10: R = *i*Pr, 36%, 4% *ee*
L18: R = Bn, 28%, 1% *ee*
L19: R = Ph, 19%, 11% *ee*
L20: R = Et, 24%, 3% *ee*
L21: R = Cyclohexyl, 32%, 10% *ee*
L22, 28%, 1% *ee*
L23, 33%, 1% *ee*
L24, 35%, 11% *ee*
L25, 18%, 15% *ee*
L26, 26%, 1% *ee*

[a]NMR yield using 2,5-dimethyl furan as the internal standard from a mixture containing other impurities after a quick flash column chromatography. [b]The *ee* values were determined by HPLC on a chiral stationary phase.

Table S5. Screening the temperature for the Ni-catalyzed enantioselective reaction of (*E*)-4-phenylbut-3-en-2-yl acetate with **1a**.

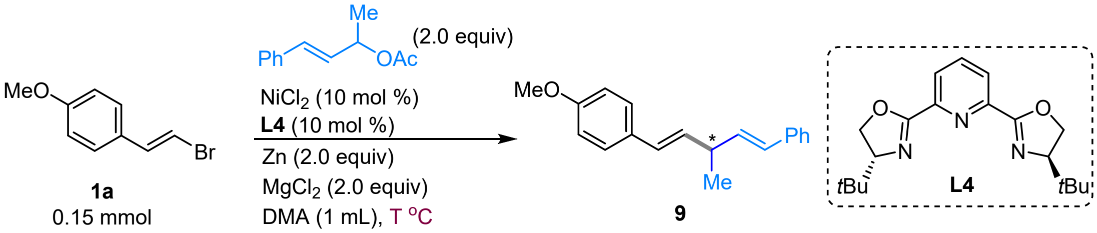

entry	T °C	yield%[a]	*ee*%[b]
1	40°C	37	12
2	25°C	36	15
3	0°C	43	22
4	-5°C	41	24
5	-10°C	38	20

[a]NMR yield using 2,5-dimethyl furan as the internal standard from a mixture containing other impurities after a quick flash column chromatography. [b]The *ee* values were determined by HPLC on a chiral stationary phase.

Table S6. Screening the variations for the reaction of **1a** with **1c**.

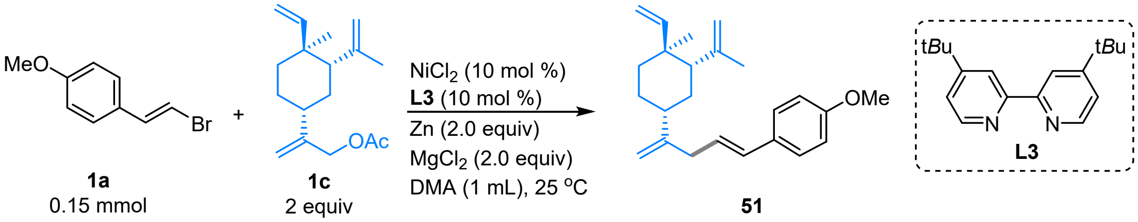

entry	Variations	yield%[a]
1	none (**1a** : **1c** = 1.0 : 2.0)	86
2	**1a** : **1c** = 1.0 : 1.0	40
3	**1a** : **1c** = 1.2 : 1.0	51
4	**1a** : **1c** = 1.5 : 1.0	78
5	**1a** : **1c** = 2.0 : 1.0	80

[a]Isolated yield.

Scheme S1. Ni-catalyzed enantiospecific cross-electrophile vinylation of allylic acetates with **1a**.

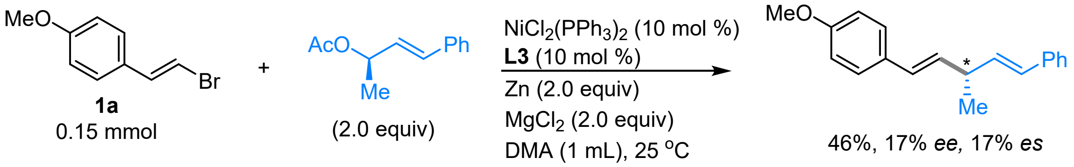

2. Ineffective Substrates:

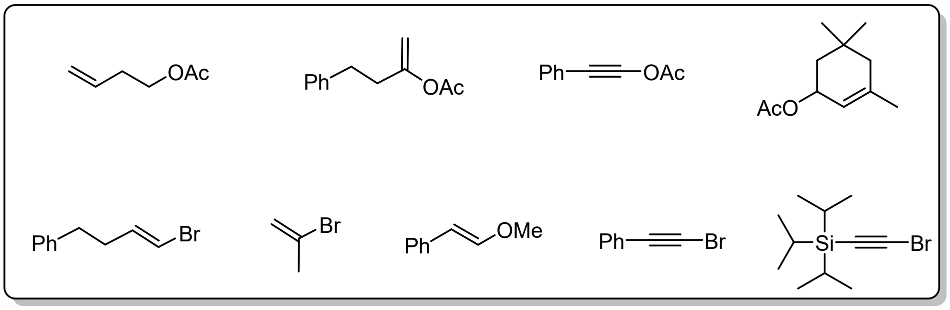

III. Cross-Coupling of Allylic Acetates with Vinyl Bromides

1. General Procedure

To an oven-dried 8 mL screw-cap vial equipped with a magnetic stir bar was charged with alkenyl bromide (0.150 mmol, 1.0 equiv, if solid), allylic acetate (0.300 mmol, 2.0 equiv, if solid), $NiCl_2(PPh_3)_2$ (9.8 mg, 0.015 mmol, 10 mol %), Zn (19.6 mg, 0.300 mmol, 2.0 equiv). The vial was introduced into a glove box, to which 4,4'-di-*tert*-butyl-2,2'-bipyridine (**L3**, 4.0 mg, 0.015 mmol, 10 mol %) and $MgCl_2$ (28.6 mg, 0.300 mmol, 2.0 equiv) was added. The tube was sealed with a teflon-lined screw cap, removed from the glove box. Alkenyl bromide (0.150 mmol, 1.0 equiv, if liquid), allylic acetate (0.300 mmol, 2.0 equiv, if liquid), and DMA (1.0 mL) were added via a syringe. The reaction mixture was allowed to stir at 25 ^{o}C for 12 h. After the reaction was complete, the reaction mixture was directly filtered through a short pad of silica gel (using ethyl acetate in petroleum ether) to give the product. All yields were an average of two runs.

2. Details of the Experimental Data

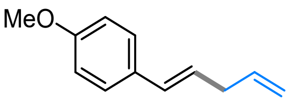

(*E*)-1-Methoxy-4-(penta-1,4-dien-1-yl)benzene (1).

The title compound was prepared following the general procedure using (*E*)-1-(2-bromovinyl)-4-methoxybenzene (32.0 mg, 0.150 mmol, 1.0 equiv), allyl acetate (30.0 mg, 0.300 mmol, 2.0 equiv). After purification by column chromatography (using 2% ethyl acetate in petroleum ether), the title compound was isolated in 85% yield (22.2 mg) as a colorless oil. The

spectral data were consistent with those which were reported in the literature[2] after comparison.

^{1}H NMR (500 MHz, $CDCl_3$): δ 7.29 (d, J = 8.7 Hz, 2H), 6.84 (d, J = 8.8 Hz, 2H), 6.35 (d, J = 15.8 Hz, 1H), 6.08 (dt, J = 15.8, 6.7 Hz, 1H), 5.90 (ddt, J = 16.6, 10.1, 6.4 Hz, 1H), 5.16–4.99 (m, 2H), 3.80 (s, 3H), 2.94 (ddd, J = 6.5, 2.9, 1.4 Hz, 2H).

^{13}C NMR (126 MHz, $CDCl_3$): δ 158.9, 136.9, 130.6, 130.3, 127.3, 126.1, 115.6, 114.1, 55.4, 37.1.

HRMS (ESI) m/z ($[M-H]^-$) calcd for $C_{12}H_{13}O$: 173.0972. Found: 173.0990.

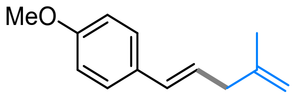

(*E*)-1-Methoxy-4-(4-methylpenta-1,4-dien-1-yl)benzene (2).

The title compound was prepared following the general procedure using (*E*)-1-(2-bromovinyl)-4-methoxybenzene (32.0 mg, 0.150 mmol, 1.0 equiv), 2-methylallyl acetate (34.2 mg, 0.300 mmol, 2.0 equiv). After purification by column chromatography (using 2% ethyl acetate in petroleum ether), the title compound was isolated in 81% yield (22.8 mg) as a colorless oil. The spectral data were consistent with those which were reported in the literature[2] after comparison.

^{1}H NMR (500 MHz, $CDCl_3$): δ 7.28 (d, J = 8.7 Hz, 2H), 6.88–6.79 (m, 2H), 6.33 (d, J = 15.8 Hz, 1H), 6.07 (dt, J = 15.8, 6.7 Hz, 1H), 5.52 (dt, J = 4.6, 3.8 Hz, 2H), 3.80 (s, 3H), 2.87 (ddd, J = 6.4, 3.5, 1.5 Hz, 2H), 1.72–1.66 (m, 3H).

^{13}C NMR (126 MHz, $CDCl_3$): δ 158.8, 132.3, 130.7, 129.7, 129.3, 127.2, 126.2, 114.0, 55.4, 36.1, 18.1.

HRMS (ESI) m/z ($[M-H]^-$) calcd for $C_{13}H_{15}O$: 187.1128. Found: 187.1120.

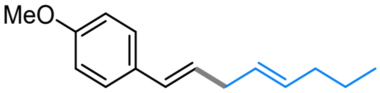

1-Methoxy-4-((1*E*,4*E*)-octa-1,4-dien-1-yl)benzene (3).

The title compound was prepared following the general procedure using (*E*)-1-(2-bromovinyl)-4-methoxybenzene (32.0 mg, 0.150 mmol, 1.0 equiv), (*E*)-hex-2-en-1-yl acetate (42.6 mg, 0.300 mmol, 2.0 equiv). After purification by column chromatography (using 2% ethyl acetate in petroleum ether), the title compound was isolated in 68% yield (22.1 mg) as a yellow oil. The spectral data were consistent with those which were reported in the literature[3] after comparison.

^{1}H NMR (500 MHz, $CDCl_3$): δ 7.29 (d, J = 8.7 Hz, 2H), 6.84 (d, J = 8.7 Hz, 2H), 6.33 (d, J = 15.8 Hz, 1H), 6.08 (dt, J = 15.8, 6.6 Hz, 1H), 5.49 (dt, J = 5.1, 3.6 Hz, 2H), 3.80 (s, 3H), 2.94–2.82 (m, 2H), 2.03–1.97 (m, 2H), 1.42–1.38 (m, 2H), 0.92 (d, J = 7.3 Hz, 3H).

^{13}C NMR (126 MHz, $CDCl_3$): δ 158.8, 131.7, 130.8, 129.7, 128.2, 127.3, 127.2, 114.0, 55.4, 36.1, 34.9, 29.8, 22.8.

HRMS (ESI) m/z ($[M-H]^-$) calcd for $C_{15}H_{19}O$: 215.1441. Found: 215.1445.

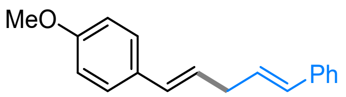

1-Methoxy-4-((1*E*,4*E*)-5-phenylpenta-1,4-dien-1-yl)benzene (4). The title compound was prepared following the general procedure using (*E*)-1-(2-bromovinyl)-4-methoxybenzene (32.0 mg, 0.150 mmol, 1.0 equiv), cinnamyl acetate (52.8 mg, 0.300 mmol, 2.0 equiv). After purification by column chromatography (using 2% ethyl acetate in petroleum ether), the title compound was isolated in 85% yield (31.8 mg) as a colorless oil. This compound was also prepared according to the general procedure using (*E*)-1-(2-bromovinyl)-4-methoxybenzene (32.0 mg, 0.150 mmol, 1.0 equiv), 1-phenylallyl acetate (52.8 mg, 0.300 mmol, 2.0 equiv). After purification by column chromatography (using 2% ethyl acetate in petroleum ether), the title compound was isolated in 72% yield (27.0 mg) as a colorless oil. The spectral data were consistent with those which were reported in the literature[4] after comparison.

^{1}H NMR (500 MHz, $CDCl_3$): δ 7.40 (d, J = 7.3 Hz, 2H), 7.33 (dd, J = 8.1, 5.5 Hz, 4H), 7.23 (t, J = 7.2 Hz, 1H), 6.87 (d, J = 8.7 Hz, 2H), 6.53–6.38 (m, 2H), 6.32 (dt, J = 15.8, 6.6 Hz, 1H), 6.17 (dt, J = 15.8, 6.6 Hz, 1H), 3.82 (s, 3H), 3.13 (t, J = 6.6 Hz, 2H).

^{13}C NMR (126 MHz, $CDCl_3$): δ 158.9, 137.7, 130.9, 130.54, 130.53, 128.7, 128.6, 127.3, 127.2, 126.2, 126.1, 114.1, 55.4, 36.3.

HRMS (ESI) m/z ($[M-H]^-$) calcd for $C_{18}H_{17}O$: 249.1285. Found: 249.1284.

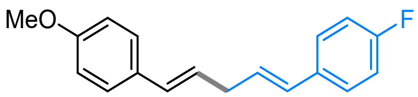

1-Fluoro-4-((1*E*,4*E*)-5-(4-methoxyphenyl)penta-1,4-dien-1-yl)benzene (5).

The title compound was prepared following the general procedure using (*E*)-1-(2-bromovinyl)-4-methoxybenzene (32.0 mg, 0.150 mmol, 1.0 equiv), (*E*)-3-(4-fluorophenyl)allyl acetate (58.2 mg, 0.300 mmol, 2.0 equiv). After purification by column chromatography (using 2% ethyl acetate in petroleum ether), the title compound was isolated in 70% yield (26.8 mg) as a colorless oil.

^{1}H NMR (500 MHz, $CDCl_3$): δ 7.54 (dd, J = 7.8, 1.6 Hz, 1H), 7.34 (d, J = 7.9 Hz, 1H), 7.32 (d, J = 8.7 Hz, 2H), 7.21 (t, J = 7.1 Hz, 1H), 7.15 (t, J = 8.4 Hz, 1H), 6.85 (dd, J = 8.0, 6.1 Hz, 3H), 6.43 (d, J = 15.8

Hz, 1H), 6.27 (dt, J = 15.8, 6.8 Hz, 1H), 6.16 (dt, J = 15.8, 6.7 Hz, 1H), 3.81 (s, 3H), 3.15 (t, J = 6.7 Hz, 2H).

^{13}C NMR (126 MHz, $CDCl_3$): δ 163.1, 161.1, 159.0, 133.9 (d, J = 3.3 Hz), 130.5 (d, J = 18.6 Hz), 129.8, 128.4 (d, J = 2.2 Hz), 127.6 (d, J = 7.9 Hz), 127.3, 125.9, 115.5 (d, J = 21.4 Hz), 114.1, 55.4, 36.2.

HRMS (ESI) m/z ($[M-H]^-$) calcd for $C_{18}H_{16}FO$: 267.1190. Found: 267.1193.

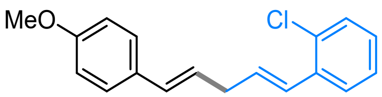

1-Chloro-2-((1*E*,4*E*)-5-(4-methoxyphenyl)penta-1,4-dien-1-yl)benzene (6).

The title compound was prepared following the general procedure using (*E*)-1-(2-bromovinyl)-4-methoxybenzene (32.0 mg, 0.150 mmol, 1.0 equiv), (*E*)-3-(2-chlorophenyl)allyl acetate (63.0 mg, 0.300 mmol, 2.0 equiv). After purification by column chromatography (using 2% ethyl acetate in petroleum ether), the title compound was isolated in 61% yield (26.0 mg) as a colorless oil.

^{1}H NMR (500 MHz, $CDCl_3$): δ 7.33 (ddd, J = 6.8, 5.8, 3.7 Hz, 4H), 7.00 (t, J = 8.7 Hz, 2H), 6.87 (d, J = 8.8 Hz, 2H), 6.51–6.36 (m, 2H), 6.18 (ddt, J = 31.9, 15.8, 6.7 Hz, 2H), 3.82 (s, 3H), 3.10 (t, J = 6.7 Hz, 2H).

^{13}C NMR (126 MHz, $CDCl_3$): δ 159.0, 135.8, 132.8, 131.6, 130.8, 130.5, 129.7, 128.2, 127.3, 127.2, 126.9, 126.8, 125.8, 114.1, 55.4, 36.6.

HRMS (ESI) m/z ($[M-H]^-$) calcd for $C_{18}H_{16}ClO$: 283.0895. Found: 283.0889.

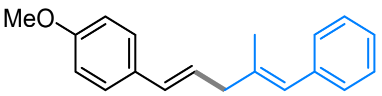

1-Methoxy-4-((1*E*,4*E*)-4-methyl-5-phenylpenta-1,4-dien-1-yl)benzene (7).

The title compound was prepared following the general procedure using (*E*)-1-(2-bromovinyl)-4-methoxybenzene (32.0 mg, 0.150 mmol, 1.0 equiv), (*E*)-2-methyl-3-phenylallyl acetate (57.0 mg, 0.300 mmol, 2.0 equiv). After purification by column chromatography (using 2% ethyl acetate in petroleum ether), the title compound was isolated in 73% yield (28.9 mg) as a colorless oil.

^{1}H NMR (500 MHz, $CDCl_3$): δ 7.32 (dd, J = 7.7, 5.3 Hz, 4H), 7.29–7.24 (m, 2H), 7.19 (t, J = 7.2 Hz, 1H), 6.86 (d, J = 8.6 Hz, 2H), 6.42 (d, J = 15.7 Hz, 1H), 6.35 (s, 1H), 6.22–6.09 (m, 1H), 3.81 (s, 3H), 3.04 (d, J = 7.0 Hz, 2H), 1.90 (s, 3H).

^{13}C NMR (126 MHz, $CDCl_3$): δ 159.0, 138.6, 137.9, 131.2, 130.6, 128.9, 128.2, 127.3, 126.2, 126.1,

125.9, 114.1, 55.5, 44.2, 18.2.

HRMS (ESI) m/z ([M-H]$^-$) calcd for $C_{19}H_{19}O$: 263.1441. Found: 263.1442.

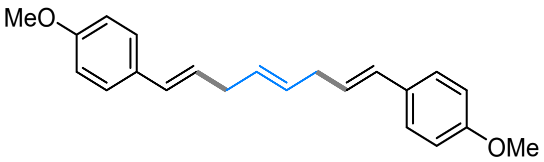

(1*E*,4*E*,7*E*)-1,8-Bis(4-methoxyphenyl)octa-1,4,7-triene (8).

The title compound was prepared following the general procedure using (*E*)-1-(2-bromovinyl)-4-methoxybenzene (64.0 mg, 0.300 mmol, 2.0 equiv), (*E*)-but-2-ene-1,4-diyl diacetate (25.8 mg, 0.150 mmol, 1.0 equiv). After purification by column chromatography (using 2% ethyl acetate in petroleum ether), the title compound was isolated in 53% yield (25.5 mg) as a yellow oil.

^{1}H NMR (500 MHz, $CDCl_3$): δ 7.29 (d, J = 8.6 Hz, 4H), 6.84 (d, J = 8.7 Hz, 4H), 6.35 (d, J = 15.8 Hz, 2H), 6.08 (dt, J = 15.7, 6.7 Hz, 2H), 5.58 (t, J = 3.4 Hz, 2H), 3.80 (s, 6H), 2.98–2.85 (m, 4H).

^{13}C NMR (126 MHz, $CDCl_3$): δ 158.9, 132.1, 129.9, 129.5, 127.3, 126.9, 114.0, 55.4, 36.1.

HRMS (ESI) m/z ([M-H]$^-$) calcd for $C_{22}H_{23}O_2$: 319.1703. Found: 319.1699.

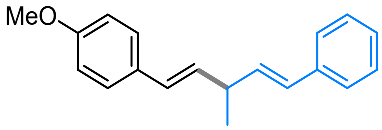

1-Methoxy-4-((*R*,1*E*,4*E*)-3-methyl-5-phenylpenta-1,4-dien-1-yl)benzene (9).

The title compound was prepared following the general procedure using (*E*)-1-(2-bromovinyl)-4-methoxybenzene (32.0 mg, 0.150 mmol, 1.0 equiv), (*E*)-4-phenylbut-3-en-2-yl acetate (57.0 mg, 0.300 mmol, 2.0 equiv). After purification by column chromatography (using 2% ethyl acetate in petroleum ether), the title compound was isolated in 62% yield (24.6 mg) as a colorless oil. The spectral data were consistent with those which were reported in the literature[5] after comparison.

This compound was also prepared according to the general procedure using 2,6-bis((*R*)-4-(tert-butyl)-4,5-dihydrooxazol-2-yl)pyridine (**L4**) as the ligand. After purification by column chromatography (using 2% ethyl acetate in petroleum ether), the title compound was isolated in 41% yield (16.2 mg, 24% *ee*) as a colorless oil.

^{1}H NMR (500 MHz, $CDCl_3$): δ 7.39 (d, J = 7.3 Hz, 2H), 7.32 (t, J = 8.4 Hz, 4H), 7.22 (t, J = 7.3 Hz, 1H), 6.87 (d, J = 8.7 Hz, 2H), 6.44 (d, J = 16.0 Hz, 1H), 6.39 (d, J = 15.9 Hz, 1H), 6.26 (dd, J = 15.9, 6.9

Hz, 1H), 6.12 (dd, J = 15.9, 6.9 Hz, 1H), 3.82 (s, 3H), 3.21 (h, J = 6.8 Hz, 1H), 1.31 (d, J = 6.9 Hz, 3H).

^{13}C NMR (126 MHz, $CDCl_3$): δ 158.9, 137.8, 134.7, 132.2, 130.6, 128.8, 128.6, 128.3, 127.3, 127.1, 126.2, 114.1, 55.4, 40.1, 20.5.

HRMS (ESI) m/z ([M-H]$^-$) calcd for $C_{19}H_{19}O$: 263.1441. Found: 263.1442.

HPLC analysis: CHIRALCEL OD-H column, 0.5% *i*PrOH in hexane, 0.5 mL/min, 254 nm UV detector, t_R (major) = 12.7 min, t_R (minor) = 14.5 min.

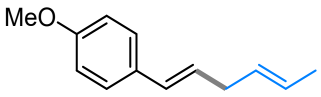

1-((1*E*,4*E*)-Hexa-1,4-dien-1-yl)-4-methoxybenzene (11).

The title compound was prepared following the general procedure using (*E*)-1-(2-bromovinyl)-4-methoxybenzene (32.0 mg, 0.150 mmol, 1.0 equiv), but-3-en-2-yl acetate (34.2 mg, 0.300 mmol, 2.0 equiv). After purification by column chromatography (using 2% ethyl acetate in petroleum ether), the title compound was isolated in 86% yield (24.3 mg) as a colorless oil.

^{1}H NMR (500 MHz, $CDCl_3$): δ 7.28 (d, J = 8.7 Hz, 2H), 6.88–6.79 (m, 2H), 6.33 (d, J = 15.8 Hz, 1H), 6.07 (dt, J = 15.8, 6.7 Hz, 1H), 5.52 (dt, J = 4.6, 3.8 Hz, 2H), 3.80 (s, 3H), 2.87 (ddd, J = 6.4, 3.5, 1.5 Hz, 2H), 1.72–1.66 (m, 3H).

^{13}C NMR (126 MHz, $CDCl_3$): δ 158.8, 132.3, 130.7, 129.7, 129.3, 127.2, 126.2, 114.0, 55.4, 36.1, 18.1.

HRMS (ESI) m/z ([M-H]$^-$) calcd for $C_{13}H_{15}O$: 187.1128. Found: 187.1127.

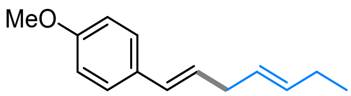

1-((1*E*,4*E*)-Hepta-1,4-dien-1-yl)-4-methoxybenzene (12).

The title compound was prepared following the general procedure using (*E*)-1-(2-bromovinyl)-4-methoxybenzene (32.0 mg, 0.150 mmol, 1.0 equiv), pent-1-en-3-yl acetate (38.4 mg, 0.300 mmol, 2.0 equiv). After purification by column chromatography (using 2% ethyl acetate in petroleum ether), the title compound was isolated in 74% yield (22.4 mg) as a pale colorless oil.

^{1}H NMR (500 MHz, $CDCl_3$): δ 7.29 (d, J = 8.7 Hz, 2H), 6.84 (d, J = 8.7 Hz, 2H), 6.34 (d, J = 15.8 Hz, 1H), 6.08 (dt, J = 15.8, 6.7 Hz, 1H), 5.58–5.44 (m, 2H), 3.80 (s, 3H), 2.88 (t, J = 6.2 Hz, 2H), 2.10–1.99 (m, 2H), 1.00 (t, J = 7.5 Hz, 3H).

^{13}C NMR (126 MHz, $CDCl_3$): δ 158.8, 133.4, 130.8, 129.7, 127.3, 127.2, 127.1, 114.0, 55.4, 36.0, 29.8,

25.7.

HRMS (ESI) m/z ([M+H]$^+$) calcd for $C_{14}H_{19}O$: 203.1430. Found: 203.1436.

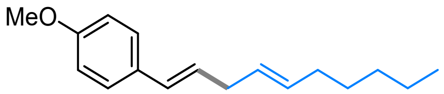

1-((1*E*,4*E*)-Deca-1,4-dien-1-yl)-4-methoxybenzene (13). The title compound was prepared following the general procedure using (*E*)-1-(2-bromovinyl)-4-methoxybenzene (32.0 mg, 0.150 mmol, 1.0 equiv), oct-1-en-3-yl acetate (51.0 mg, 0.300 mmol, 2.0 equiv). After purification by column chromatography (using 2% ethyl acetate in petroleum ether), the title compound was isolated in 71% yield (26.0 mg) as a colorless oil.

^{1}H NMR (500 MHz, $CDCl_3$): δ 7.29 (d, J = 8.7 Hz, 2H), 6.84 (d, J = 8.7 Hz, 2H), 6.34 (d, J = 15.8 Hz, 1H), 6.13–6.03 (m, 1H), 5.56–5.43 (m, 2H), 3.80 (s, 3H), 2.88 (t, J = 5.4 Hz, 2H), 2.06–1.99 (m, 2H), 1.40–1.36 (m, 2H), 1.32–1.29 (m, 4H), 0.89 (t, J = 6.8 Hz, 3H).

^{13}C NMR (126 MHz, $CDCl_3$): δ 158.7, 131.9, 130.7, 129.5, 127.8, 127.2, 127.1, 113.9, 55.3, 35.9, 32.6, 31.5, 29.2, 22.6, 14.1.

HRMS (ESI) m/z ([M-H]$^-$) calcd for $C_{17}H_{23}O$: 243.1754. Found: 243.1751.

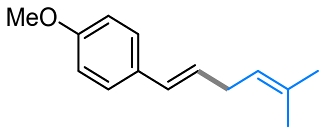

(*E*)-1-Methoxy-4-(5-methylhexa-1,4-dien-1-yl)benzene (14). The title compound was prepared following the general procedure using (*E*)-1-(2-bromovinyl)-4-methoxybenzene (32.0 mg, 0.150 mmol, 1.0 equiv), 2-methylbut-3-en-2-yl acetate (38.4 mg, 0.300 mmol, 2.0 equiv). After purification by column chromatography (using 2% ethyl acetate in petroleum ether), the title compound was isolated in 64% yield (19.4 mg) as a colorless oil. The spectral data were consistent with those which were reported in the literature[3] after comparison.

^{1}H NMR (500 MHz, $CDCl_3$): δ 7.27 (d, J = 8.8 Hz, 2H), 6.83 (d, J = 8.7 Hz, 2H), 6.32 (d, J = 15.8 Hz, 1H), 6.04 (dt, J = 15.8, 6.5 Hz, 1H), 5.21 (t, J = 7.2 Hz, 1H), 3.80 (s, 3H), 2.87 (t, J = 6.8 Hz, 2H), 1.74 (s, 3H), 1.66 (s, 3H).

^{13}C NMR (126 MHz, $CDCl_3$): δ 157.6, 131.0, 129.7, 127.9, 126.3, 126.0, 120.9, 112.9, 54.2, 30.6, 24.7, 16.7.

HRMS (ESI) m/z ([M-H]$^-$) calcd for $C_{14}H_{17}O$: 201.1285. Found: 201.1284.

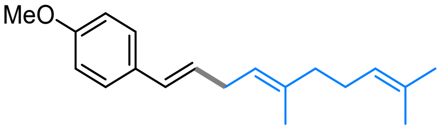

1-((1*E*,4*E*)-5,9-Dimethyldeca-1,4,8-trien-1-yl)-4-methoxybenzene (15).

The title compound was prepared following the general procedure using (*E*)-1-(2-bromovinyl)-4-methoxybenzene (32.0 mg, 0.150 mmol, 1.0 equiv), 3,7-dimethylocta-1,6-dien-3-yl acetate (58.8 mg, 0.300 mmol, 2.0 equiv). After purification by column chromatography (using 2% ethyl acetate in petroleum ether), the title compound was isolated in 52% yield (21.1 mg) as a colorless oil. The spectral data were consistent with those which were reported in the literature[4] after comparison.

^{1}H NMR (500 MHz, $CDCl_3$): δ 7.28 (d, J = 8.8 Hz, 2H), 6.84 (d, J = 8.7 Hz, 2H), 6.33 (d, J = 15.8 Hz, 1H), 6.05 (ddd, J = 14.4, 10.5, 6.6 Hz, 1H), 5.24 (t, J = 7.2 Hz, 1H), 5.14 (t, J = 7.0 Hz, 1H), 3.80 (s, 3H), 2.90 (t, J = 6.8 Hz, 2H), 2.17–2.02 (m, 4H), 1.69 (dd, J = 40.1, 23.1 Hz, 9H).

^{13}C NMR (126 MHz, $CDCl_3$): δ 158.8, 136.6, 131.6, 130.9, 129.0, 127.5, 127.2, 124.4, 121.8, 114.0, 55.4, 39.9, 31.6, 26.8, 25.9, 17.8, 16.2.

HRMS (ESI) m/z ($[M-H]^-$) calcd for $C_{19}H_{25}O$: 269.1911. Found: 269.1915.

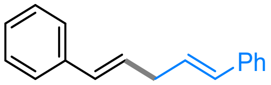

(1*E*,4*E*)-1,5-Diphenylpenta-1,4-diene (16).

The title compound was prepared following the general procedure using (*E*)-(2-bromovinyl)benzene (27.5 mg, 0.150 mmol, 1.0 equiv), cinnamyl acetate (52.8 mg, 0.300 mmol, 2.0 equiv). After purification by column chromatography (using 2% ethyl acetate in petroleum ether), the title compound was isolated in 73% yield (24.1 mg) as a colorless oil. The spectral data were consistent with those which were reported in the literature[4] after comparison.

^{1}H NMR (500 MHz, $CDCl_3$): δ 7.31–7.28 (m, 4H), 7.19 (ddd, J = 8.6, 5.7, 3.1 Hz, 1H), 6.55 (s, 2H), 6.37–6.26 (m, 2H), 3.84 (s, 6H), 3.82 (s, 3H), 3.78 (dd, J = 14.8, 8.5 Hz, 1H), 1.41–1.35 (m, 2H), 1.15 (s, 12H).

^{13}C NMR (126 MHz, $CDCl_3$): δ 137.8, 131.0, 130.5, 128.6, 127.3, 126.2, 36.3.

HRMS (ESI) m/z ($[M-H]^-$) calcd for $C_{17}H_{15}$: 219.1179. Found: 219.1169.

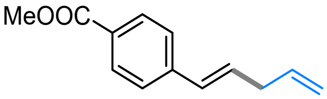

Methyl (*E*)-4-(penta-1,4-dien-1-yl)benzoate (17).

The title compound was prepared following the general procedure

using methyl (*E*)-4-(2-bromovinyl)benzoate (36.2 mg, 0.150 mmol, 1.0 equiv), allyl acetate (30.0 mg, 0.300 mmol, 2.0 equiv). After purification by column chromatography (using 2% ethyl acetate in petroleum ether), the title compound was isolated in 86% yield (26.1 mg) as a colorless oil.

^{1}H NMR (500 MHz, $CDCl_3$): δ 7.96 (d, J = 8.4 Hz, 2H), 7.39 (d, J = 8.4 Hz, 2H), 6.44 (d, J = 15.9 Hz, 1H), 6.35 (dt, J = 15.9, 6.4 Hz, 1H), 5.90 (ddt, J = 16.7, 10.1, 6.4 Hz, 1H), 5.15–5.05 (m, 2H), 3.89 (s, 3H), 2.98 (t, J = 6.4 Hz, 2H).

^{13}C NMR (126 MHz, $CDCl_3$): δ 167.0, 142.2, 135.9, 131.2, 130.1, 129.9, 128.6, 126.0, 116.2, 52.1, 37.1.

HRMS (ESI) m/z ($[M+H]^+$) calcd for $C_{13}H_{15}O_2$: 203.1067. Found: 203.1069.

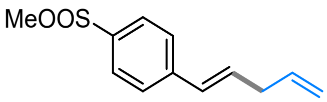

(*E*)-1-(Methylsulfonyl)-4-(penta-1,4-dien-1-yl)benzene (18).

The title compound was prepared following the general procedure using (*E*)-1-(2-bromovinyl)-4-(methylsulfonyl)benzene (39.2 mg, 0.150 mmol, 1.0 equiv), allyl acetate (30.0 mg, 0.300 mmol, 2.0 equiv). After purification by column chromatography (using 2% ethyl acetate in petroleum ether), the title compound was isolated in 70% yield (23.3 mg) as a colorless oil.

^{1}H NMR (500 MHz, $CDCl_3$): δ 7.85 (d, J = 8.4 Hz, 2H), 7.51 (d, J = 8.4 Hz, 2H), 6.51–6.36 (m, 2H), 5.89 (ddt, J = 16.7, 10.1, 6.4 Hz, 1H), 5.11 (ddd, J = 8.8, 6.1, 2.2 Hz, 2H), 3.04 (s, 3H), 3.00 (t, J = 5.9 Hz, 2H).

^{13}C NMR (126 MHz, $CDCl_3$): δ 143.2, 138.6, 135.6, 132.9, 129.4, 127.8, 126.8, 116.6, 44.7, 37.1.

HRMS (ESI) m/z ($[M+H]^+$) calcd for $C_{12}H_{15}O_2S$: 223.0787. Found: 223.0785.

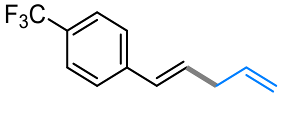

(*E*)-1-(Penta-1,4-dien-1-yl)-4-(trifluoromethyl)benzene (19).

The title compound was prepared following the general procedure using (*E*)-1-(2-bromovinyl)-4-(trifluoromethyl)benzene (37.7 mg, 0.150 mmol, 1.0 equiv), allyl acetate (30.0 mg, 0.300 mmol, 2.0 equiv). After purification by column chromatography (using 2% ethyl acetate in petroleum ether), the title compound was isolated in 76%

yield (24.2 mg) as a colorless oil. The spectral data were consistent with those which were reported in the literature[2] after comparison.

^{1}H NMR (500 MHz, $CDCl_3$): δ 7.54 (d, J = 8.2 Hz, 2H), 7.44 (d, J = 8.2 Hz, 2H), 6.44 (d, J = 15.9 Hz, 1H), 6.34 (dt, J = 15.9, 6.5 Hz, 1H), 5.90 (ddt, J = 16.7, 10.1, 6.4 Hz, 1H), 5.19–5.02 (m, 2H), 2.99 (t, J = 6.4 Hz, 2H).

^{13}C NMR (126 MHz, $CDCl_3$): δ 141.2, 136.0, 131.2, 129.8, 126.7, 126.4, 126.3, 125.6 (q, J = 3.8 Hz), 116.3, 37.1.

HRMS (ESI) m/z ($[M-H]^-$) calcd for $C_{12}H_{10}F_3$: 211.0740. Found: 211.1051.

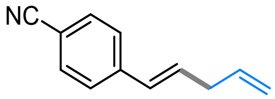

(*E*)-4-(Penta-1,4-dien-1-yl)benzonitrile (20).

The title compound was prepared following the general procedure using (*E*)-4-(2-bromovinyl)benzonitrile (31.2 mg, 0.150 mmol, 1.0 equiv), allyl acetate (30.0 mg, 0.300 mmol, 2.0 equiv). After purification by column chromatography (using 10% ethyl acetate in petroleum ether), the title compound was isolated in 73% yield (18.5 mg) as a white solid.

^{1}H NMR (500 MHz, $CDCl_3$): δ 7.75 (d, J = 8.3 Hz, 2H), 7.41 (d, J = 8.3 Hz, 2H), 6.37 (ddd, J = 27.9, 15.9, 11.2 Hz, 2H), 5.90 (ddt, J = 16.7, 10.1, 6.4 Hz, 1H), 5.22–5.01 (m, 2H), 2.99 (t, J = 6.4 Hz, 2H).

^{13}C NMR (126 MHz, $CDCl_3$): δ 141.5, 136.0, 131.6, 131.1, 130.0, 128.1, 127.8, 126.3, 116.3, 37.2.

HRMS (ESI) m/z ($[M+H^+)$ calcd for $C_{12}H_{12}N$: 170.0964. Found: 170.0969.

M.p.: 75-76 ºC.

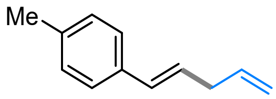

(*E*)-1-Methyl-4-(penta-1,4-dien-1-yl)benzene (21).

The title compound was prepared following the general procedure using (*E*)-1-(2-bromovinyl)-4-methylbenzene (29.5 mg, 0.150 mmol, 1.0 equiv), allyl acetate (30.0 mg, 0.300 mmol, 2.0 equiv). After purification by column chromatography (using 2% ethyl acetate in petroleum ether), the title compound was isolated in 80% yield (18.9 mg) as a colorless oil. The spectral data were consistent with those which were reported in the literature[6] after comparison.

^{1}H NMR (500 MHz, $CDCl_3$): δ 7.24 (d, J = 8.0 Hz, 2H), 7.10 (d, J = 7.9 Hz, 2H), 6.38 (d, J = 15.9 Hz, 1H), 6.17 (dt, J = 15.8, 6.7 Hz, 1H), 5.90 (ddt, J = 16.6, 10.1, 6.4 Hz, 1H), 5.15–4.99 (m, 2H), 2.95 (t, J = 6.5 Hz, 2H), 2.32 (s, 3H).

^{13}C NMR (126 MHz, $CDCl_3$): δ 136.9, 136.8, 135.0, 130.8, 129.3, 127.3, 126.1, 115.7, 37.2, 29.9.

HRMS (ESI) m/z ($[M-H]^-$) calcd for $C_{12}H_{13}$: 157.1023. Found: 157.1012.

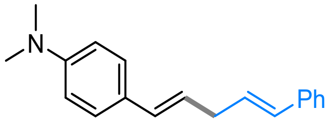

N,N-Dimethyl-4-((1*E*,4*E*)-5-phenylpenta-1,4-dien-1-yl)aniline (22).

The title compound was prepared following the general procedure using (*E*)-4-(2-bromovinyl)-N,N-dimethylaniline (33.9 mg, 0.150 mmol, 1.0 equiv), cinnamyl acetate (52.8 mg, 0.300 mmol, 2.0 equiv). After purification by column chromatography (using 2% ethyl acetate in petroleum ether), the title compound was isolated in 67% yield (26.4 mg) as a yellow oil.

^{1}H NMR (500 MHz, $CDCl_3$): δ 7.35 (d, J = 7.5 Hz, 2H), 7.27 (dd, J = 16.6, 8.2 Hz, 4H), 7.18 (t, J = 7.4 Hz, 1H), 6.66 (d, J = 8.8 Hz, 2H), 6.44 (d, J = 15.8 Hz, 1H), 6.37 (d, J = 15.8 Hz, 1H), 6.28 (dt, J = 15.8, 6.6 Hz, 1H), 6.06 (dt, J = 15.8, 6.7 Hz, 1H), 3.07 (t, J = 6.6 Hz, 2H), 2.92 (s, 6H).

^{13}C NMR (126 MHz, $CDCl_3$): δ 149.9, 137.8, 131.0, 130.7, 129.1, 128.6, 127.2, 127.1, 126.4, 126.2, 123.9, 112.7, 40.7, 36.4.

HRMS (ESI) m/z ($[M+H]^+$) calcd for $C_{19}H_{22}N$: 264.1747. Found: 264.1807.

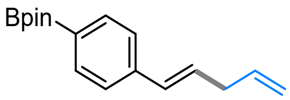

(*E*)-4,4,5,5-Tetramethyl-2-(4-(penta-1,4-dien-1-yl)phenyl)-1,3,2-dioxaborolane (23).

The title compound was prepared following the general procedure using (*E*)-2-(4-(2-bromovinyl)phenyl)-4,4,5,5-tetramethyl-1,3,2-dioxaborolane (46.4 mg, 0.150 mmol, 1.0 equiv), allyl acetate (30.0 mg, 0.300 mmol, 2.0 equiv). After purification by column chromatography (using 5% ethyl acetate in petroleum ether), the title compound was isolated in 70% yield (28.4 mg) as a yellow oil. The spectral data were consistent with those which were reported in the literature[7] after comparison.

^{1}H NMR (500 MHz, $CDCl_3$): δ 7.75 (d, J = 8.0 Hz, 2H), 7.36 (d, J = 8.0 Hz, 2H), 6.43 (d, J = 15.9 Hz, 1H), 6.31 (dt, J = 15.8, 6.6 Hz, 1H), 5.91 (ddt, J = 16.7, 10.1, 6.4 Hz, 1H), 5.10 (ddd, J = 13.6, 11.5, 1.5 Hz, 2H), 2.98 (t, J = 6.5 Hz, 2H), 1.35 (s, 12H).

^{13}C NMR (126 MHz, $CDCl_3$): δ 140.5, 136.4, 135.2, 131.0, 129.5, 125.7, 125.5, 116.0, 83.8, 37.2, 25.0.

HRMS (ESI) m/z ($[M+H]^+$) calcd for $C_{17}H_{24}BO_2$: 271.1864. Found: 271.1865.

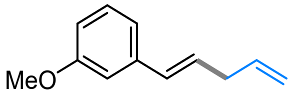

(*E*)-1-Methoxy-3-(penta-1,4-dien-1-yl)benzene (24).

The title compound was prepared following the general procedure using (*E*)-1-(2-bromovinyl)-3-methoxybenzene (32.0 mg, 0.150 mmol, 1.0 equiv), allyl acetate (30.0 mg, 0.300 mmol, 2.0 equiv). After purification by column chromatography (using 2% ethyl acetate in petroleum ether), the title compound was isolated in 83% yield (21.7 mg) as a colorless oil. The spectral data were consistent with those which were reported in the literature[8] after comparison.

^{1}H NMR (500 MHz, $CDCl_3$): δ 7.21 (t, J = 7.9 Hz, 1H), 6.95 (d, J = 7.6 Hz, 1H), 6.93–6.84 (m, 1H), 6.76 (dd, J = 8.0, 2.2 Hz, 1H), 6.38 (d, J = 15.8 Hz, 1H), 6.23 (dt, J = 15.8, 6.6 Hz, 1H), 5.96–5.84 (m, 1H), 5.15–5.02 (m, 2H), 3.81 (s, 3H), 2.96 (t, J = 6.5 Hz, 2H).

^{13}C NMR (126 MHz, $CDCl_3$): δ 159.9, 139.3, 136.6, 130.9, 129.6, 128.7, 118.9, 115.9, 112.8, 111.5, 55.3, 37.1.

HRMS (ESI) m/z ([M-H]$^-$) calcd for $C_{12}H_{13}O$: 173.0972. Found: 173.0990.

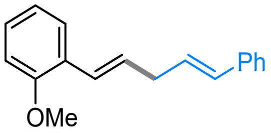

(*E*)-1-Methoxy-2-(penta-1,4-dien-1-yl)benzene (25).

The title compound was prepared following the general procedure using methyl (*E*)-1-(2-bromovinyl)-2-methoxybenzene (32.0 mg, 0.150 mmol, 1.0 equiv), cinnamyl acetate (52.8 mg, 0.300 mmol, 2.0 equiv). After purification by column chromatography (using 2% ethyl acetate in petroleum ether), the title compound was isolated in 79% yield (20.6 mg) as a colorless oil. The spectral data were consistent with those which were reported in the literature[4] after comparison.

^{1}H NMR (500 MHz, $CDCl_3$): δ 7.47 (dd, J = 7.6, 1.5 Hz, 1H), 7.40 (d, J = 7.3 Hz, 2H), 7.32 (t, J = 7.7 Hz, 2H), 7.23 (t, J = 7.5 Hz, 2H), 6.94 (t, J = 7.4 Hz, 1H), 6.89 (d, J = 8.2 Hz, 1H), 6.82 (d, J = 16.0 Hz, 1H), 6.50 (d, J = 15.8 Hz, 1H), 6.32 (ddt, J = 16.0, 11.2, 6.7 Hz, 2H), 3.87 (s, 3H), 3.17 (t, J = 6.7 Hz, 2H).

^{13}C NMR (126 MHz, $CDCl_3$): δ 156.5, 137.8, 130.9, 129.0, 128.7, 128.6, 128.2, 127.1, 126.77, 126.76, 126.2, 125.9, 120.8, 110.9, 55.6, 36.9.

HRMS (ESI) m/z ([M-H]$^-$) calcd for $C_{18}H_{17}O$: 249.1285. Found: 249.1284.

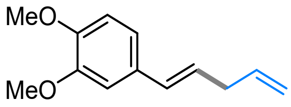

(*E*)-1,2-Dimethoxy-4-(penta-1,4-dien-1-yl)benzene (26).

The title compound was prepared following the general procedure using

(*E*)-4-(2-bromovinyl)-1,2-dimethoxybenzene (36.5 mg, 0.150 mmol, 1.0 equiv), allyl acetate (30.0 mg, 0.300 mmol, 2.0 equiv). After purification by column chromatography (using 5% ethyl acetate in petroleum ether), the title compound was isolated in 73% yield (22.3 mg) as a colorless oil.

^{1}H NMR (500 MHz, $CDCl_3$): δ 6.92 (d, J = 1.9 Hz, 1H), 6.88 (dd, J = 8.2, 1.9 Hz, 1H), 6.80 (d, J = 8.2 Hz, 1H), 6.35 (d, J = 15.8 Hz, 1H), 6.09 (dt, J = 15.8, 6.7 Hz, 1H), 5.91 (ddt, J = 16.6, 10.1, 6.4 Hz, 1H), 5.20–5.01 (m, 2H), 3.89 (s, 3H), 3.87 (s, 3H), 2.95 (t, J = 6.6 Hz, 2H).

^{13}C NMR (126 MHz, $CDCl_3$): δ 149.1, 148.5, 136.8, 130.9, 130.6, 126.4, 119.1, 115.7, 111.3, 108.7, 56.0, 55.9, 37.1.

HRMS (ESI) m/z ([M+H]$^+$) calcd for $C_{13}H_{17}O_2$: 205.1223. Found: 205.1227.

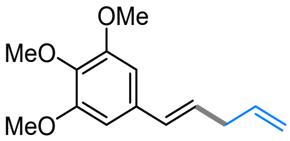

(*E*)-1,2,3-Trimethoxy-5-(penta-1,4-dien-1-yl)benzene (27).

The title compound was prepared following the general procedure using (*E*)-5-(2-iodovinyl)-1,2,3-trimethoxybenzene (48.0 mg, 0.150 mmol, 1.0 equiv), allyl acetate (30.0 mg, 0.300 mmol, 2.0 equiv). After purification by column chromatography (using 5% ethyl acetate in petroleum ether), the title compound was isolated in 78% yield (27.4 mg) as a yellow oil. The spectral data were consistent with those which were reported in the literature[9] after comparison.

^{1}H NMR (500 MHz, $CDCl_3$): δ 6.58 (s, 2H), 6.34 (d, J = 15.8 Hz, 1H), 6.14 (dt, J = 15.7, 6.7 Hz, 1H), 5.90 (ddt, J = 16.6, 10.1, 6.4 Hz, 1H), 5.21–4.98 (m, 2H), 3.87 (s, 6H), 3.83 (s, 3H), 2.96 (t, J = 6.5 Hz, 2H).

^{13}C NMR (126 MHz, $CDCl_3$): δ 153.4, 137.5, 136.5, 133.5, 130.9, 127.9, 115.9, 103.2, 61.0, 56.2, 37.0.

HRMS (ESI) m/z ([M+H]$^+$) calcd for $C_{14}H_{19}O_3$: 235.1329. Found: 235.1332.

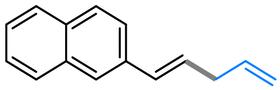

(*E*)-2-(Penta-1,4-dien-1-yl)naphthalene (28).

The title compound was prepared following the general procedure using (*E*)-2-(2-bromovinyl)naphthalene (34.9 mg, 0.150 mmol, 1.0 equiv), allyl acetate (30.0 mg, 0.300 mmol, 2.0 equiv). After purification by column chromatography (using 2% ethyl acetate in petroleum ether), the title compound was isolated in 85% yield (24.7 mg) as a yellow oil. The spectral data were consistent with those which were reported in the literature[10] after comparison.

^{1}H NMR (500 MHz, $CDCl_3$): δ 7.79 (t, J = 9.0 Hz, 3H), 7.70 (s, 1H), 7.60 (dd, J = 8.5, 1.7 Hz, 1H),

7.44 (tdd, *J* = 8.1, 6.9, 1.4 Hz, 2H), 6.59 (d, *J* = 15.9 Hz, 1H), 6.38 (dt, *J* = 15.8, 6.7 Hz, 1H), 5.96 (ddt, *J* = 16.6, 10.1, 6.4 Hz, 1H), 5.20–5.08 (m, 2H), 3.04 (t, *J* = 5.8 Hz, 2H).

^{13}C NMR (126 MHz, $CDCl_3$): *δ* 136.6, 135.2, 133.8, 132.9, 131.1, 128.8, 128.2, 128.0, 127.8, 126.3, 125.73, 125.69, 123.7, 115.9, 37.3.

HRMS (ESI) m/z ($[M-H]^-$) calcd for $C_{15}H_{13}$: 193.1023. Found: 193.1016.

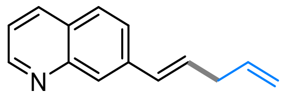

(*E*)-7-(Penta-1,4-dien-1-yl)quinoline (29).

The title compound was prepared following the general procedure using (*E*)-7-(2-bromovinyl)quinoline (35.1 mg, 0.150 mmol, 1.0 equiv), allyl acetate (30.0 mg, 0.300 mmol, 2.0 equiv). After purification by column chromatography (using 5% ethyl acetate in petroleum ether), the title compound was isolated in 73% yield (21.3 mg) as a yellow oil.

^{1}H NMR (500 MHz, $CDCl_3$): *δ* 8.95 (dd, *J* = 4.1, 1.7 Hz, 1H), 8.13 (dd, *J* = 8.3, 1.7 Hz, 1H), 7.88 (d, *J* = 7.0 Hz, 1H), 7.77–7.67 (m, 2H), 7.51 (t, *J* = 7.7 Hz, 1H), 7.40 (dd, *J* = 8.2, 4.2 Hz, 1H), 6.46 (dt, *J* = 16.0, 6.8 Hz, 1H), 6.00 (ddt, *J* = 16.7, 10.1, 6.4 Hz, 1H), 5.13 (ddd, *J* = 13.6, 11.6, 1.6 Hz, 2H), 3.14 (td, *J* = 6.7, 1.4 Hz, 2H).

^{13}C NMR (126 MHz, $CDCl_3$): *δ* 149.6, 145.8, 136.9, 136.5, 136.4, 130.8, 128.6, 127.0, 126.9, 126.6, 125.6, 121.2, 115.8, 37.8.

HRMS (ESI) m/z ($[M+H]^+$) calcd for $C_{14}H_{14}N$: 196.1121. Found: 196.1201.

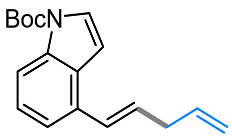

***tert*-Butyl (*E*)-4-(penta-1,4-dien-1-yl)-1H-indole-1-carboxylate (30)**.

The title compound was prepared following the general procedure using *tert*-butyl (*E*)-4-(2-bromovinyl)-1*H*-indole-1-carboxylate (48.3 mg, 0.150 mmol, 1.0 equiv), allyl acetate (30.0 mg, 0.300 mmol, 2.0 equiv). After purification by column chromatography (using 5% ethyl acetate in petroleum ether), the title compound was isolated in 79% yield (33.5 mg) as a colorless oil.

^{1}H NMR (500 MHz, $CDCl_3$): *δ* 8.05 (d, *J* = 7.8 Hz, 1H), 7.62 (d, *J* = 3.6 Hz, 1H), 7.34 (d, *J* = 7.5 Hz, 1H), 7.30–7.24 (m, 1H), 6.84–6.68 (m, 2H), 6.36 (dt, *J* = 15.8, 6.7 Hz, 1H), 5.97 (ddt, *J* = 16.6, 10.1, 6.4

Hz, 1H), 5.23–5.03 (m, 2H), 3.06 (td, J = 6.6, 1.2 Hz, 2H), 1.69 (s, 9H).

^{13}C NMR (126 MHz, $CDCl_3$): δ 149.9, 136.6, 135.6, 130.4, 129.8, 128.7, 128.1, 125.9, 124.5, 119.3, 115.9, 113.9, 105.6, 83.8, 37.5, 28.3.

HRMS (ESI) m/z ([M+H]$^+$) calcd for $C_{18}H_{22}NO_2$: 284.1645. Found: 284.1646.

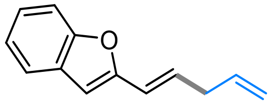

(*E*)-2-(Penta-1,4-dien-1-yl)benzofuran (31).

The title compound was prepared following the general procedure using (*E*)-2-(2-bromovinyl)benzofuran (33.5 mg, 0.150 mmol, 1.0 equiv), allyl acetate (30.0 mg, 0.300 mmol, 2.0 equiv). After purification by column chromatography (using 2% ethyl acetate in petroleum ether), the title compound was isolated in 76% yield (21.0 mg) as a yellow oil.

^{1}H NMR (500 MHz, $CDCl_3$): δ 7.49 (d, J = 7.4 Hz, 1H), 7.41 (d, J = 8.0 Hz, 1H), 7.24 (d, J = 6.8 Hz, 1H), 7.20 (d, J = 9.6 Hz, 1H), 6.54–6.44 (m, 2H), 6.39–6.31 (m, 1H), 6.00–5.84 (m, 1H), 5.12 (t, J = 14.4 Hz, 2H), 3.00 (t, J = 6.3 Hz, 2H).

^{13}C NMR (126 MHz, $CDCl_3$): δ 155.1, 154.8, 135.8, 131.0, 129.2, 124.3, 122.8, 120.8, 119.7, 116.4, 110.9, 103.3, 36.9.

HRMS (ESI) m/z ([M-H]$^-$) calcd for $C_{13}H_{13}O$: 183.0815. Found: 183.0813.

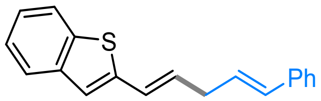

2-((1*E*,4*E*)-5-Phenylpenta-1,4-dien-1-yl)benzo[*b*]thiophene (32).

The title compound was prepared following the general procedure using (*E*)-2-(2-bromovinyl)benzo[*b*]thiophene (35.9 mg, 0.150 mmol, 1.0 equiv), cinnamyl acetate (52.8 mg, 0.300 mmol, 2.0 equiv). After purification by column chromatography (using 2% ethyl acetate in petroleum ether), the title compound was isolated in 77% yield (31.8 mg) as a yellow solid.

^{1}H NMR (500 MHz, $CDCl_3$): δ 7.73 (d, J = 7.5 Hz, 1H), 7.65 (d, J = 7.2 Hz, 1H), 7.38 (d, J = 7.5 Hz, 2H), 7.30 (d, J = 7.8 Hz, 2H), 7.29–7.25 (m, 2H), 7.24–7.20 (m, 1H), 7.07 (s, 1H), 6.67 (d, J = 15.6 Hz, 1H), 6.48 (d, J = 15.8 Hz, 1H), 6.25 (ddt, J = 22.3, 15.5, 6.6 Hz, 2H), 3.13 (t, J = 6.6 Hz, 2H).

^{13}C NMR (126 MHz, $CDCl_3$): δ 143.0, 140.3, 138.8, 137.5, 131.7, 131.1, 128.7, 127.5, 127.4, 126.3, 125.1, 124.52, 124.48, 123.4, 122.3, 121.9, 36.2.

HRMS (ESI) m/z ([M-H]$^-$) calcd for $C_{19}H_{15}S$: 275.0900. Found: 275.0901.

M.p.: 81-82 ºC.

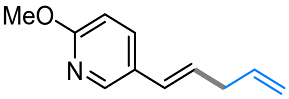

(*E*)-5-(Penta-1,4-dien-1-yl)-2-methoxylpyridine (33).

The title compound was prepared following the general procedure using (*E*)-5-(2-bromovinyl)-2-methoxylpyridine (32.1 mg, 0.150 mmol, 1.0 equiv), allyl acetate (30.0 mg, 0.300 mmol, 2.0 equiv). After purification by column chromatography (using 5% ethyl acetate in petroleum ether), the title compound was isolated in 69% yield (18.1 mg) as a colorless oil.

^{1}H NMR (500 MHz, $CDCl_3$): δ 8.07 (d, J = 2.3 Hz, 1H), 7.63 (dd, J = 8.6, 2.4 Hz, 1H), 6.69 (d, J = 8.6 Hz, 1H), 6.33 (d, J = 15.9 Hz, 1H), 6.11 (dt, J = 15.9, 6.6 Hz, 1H), 5.89 (ddt, J = 16.7, 10.1, 6.4 Hz, 1H), 5.17–5.01 (m, 2H), 3.93 (s, 3H), 2.96 (t, J = 6.5 Hz, 2H).

^{13}C NMR (126 MHz, $CDCl_3$): δ 163.4, 145.1, 136.4, 135.5, 129.7, 127.8, 127.1, 116.0, 110.9, 53.6, 37.2.

HRMS (ESI) m/z ($[M+H]^+$) calcd for $C_{11}H_{14}NO$: 176.1070. Found: 176.1071.

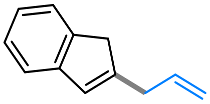

2-Allyl-1*H*-indene (34).

The title compound was prepared following the general procedure using 2-bromo-1*H*-indene (29.3 mg, 0.150 mmol, 1.0 equiv), allyl acetate (30.0 mg, 0.300 mmol, 2.0 equiv). After purification by column chromatography (using 2% ethyl acetate in petroleum ether), the title compound was isolated in 72% yield (16.8 mg) as a colorless oil. The spectral data were consistent with those which were reported in the literature[11] after comparison.

^{1}H NMR (500 MHz, $CDCl_3$): δ 7.38 (d, J = 7.4 Hz, 1H), 7.27 (d, J = 7.5 Hz, 1H), 7.22 (t, J = 7.4 Hz, 1H), 7.10 (t, J = 7.9 Hz, 1H), 6.54 (s, 1H), 5.97 (ddt, J = 16.9, 10.0, 6.8 Hz, 1H), 5.19–5.04 (m, 2H), 3.32 (s, 2H), 3.24 (d, J = 6.7 Hz, 2H).

^{13}C NMR (126 MHz, $CDCl_3$): δ 148.5, 145.6, 143.4, 136.3, 127.2, 126.4, 123.9, 123.6, 120.2, 116.3, 41.2, 35.9.

HRMS (ESI) m/z ($[M-H]^-$) calcd for $C_{12}H_{11}$: 155.0866. Found: 155.0843.

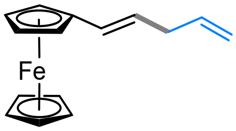

Ferrocene penta-1,4-diene (35).

The title compound was prepared following the general procedure using ferrocene vinyl bromide (43.5 mg, 0.150 mmol, 1.0 equiv), allyl acetate (30.0 mg, 0.300 mmol, 2.0 equiv). After purification by column chromatography (using 2% ethyl acetate in

petroleum ether), the title compound was isolated in 58% yield (21.9 mg) as a brown oil.

^{1}H NMR (500 MHz, $CDCl_3$): δ 6.13 (d, J = 15.7 Hz, 1H), 5.96–5.78 (m, 2H), 5.08 (dd, J = 24.8, 13.5 Hz, 2H), 4.31 (s, 2H), 4.17 (s, 2H), 4.10 (s, 5H), 2.83 (t, J = 5.7 Hz, 2H).

^{13}C NMR (126 MHz, $CDCl_3$): δ 137.1, 128.0, 125.5, 115.3, 84.0, 70.1, 69.2, 68.4, 66.5, 37.1, 29.8.

HRMS (ESI) m/z ($[M+H]^+$) calcd for $C_{15}H_{17}Fe$: 253.0674. Found: 253.0648.

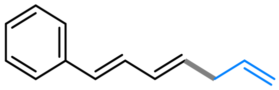

((1*E*,3*E*)-Hpta-1,3,6-trien-1-yl)benzene (36).

The title compound was prepared following the general procedure using ((1*E*,3*E*)-4-bromobuta-1,3-dien-1-yl)benzene (31.3 mg, 0.150 mmol, 1.0 equiv), allyl acetate (30.0 mg, 0.300 mmol, 2.0 equiv). After purification by column chromatography (using 2% ethyl acetate in petroleum ether), the title compound was isolated in 75% yield (19.2 mg) as a yellow oil. The spectral data were consistent with those which were reported in the literature[12] after comparison.

^{1}H NMR (500 MHz, $CDCl_3$): δ 7.39 (d, J = 7.3 Hz, 2H), 7.31 (t, J = 7.7 Hz, 2H), 7.21 (t, J = 7.3 Hz, 1H), 6.78 (dd, J = 15.7, 10.4 Hz, 1H), 6.48 (d, J = 15.7 Hz, 1H), 6.25 (dd, J = 14.9, 10.7 Hz, 1H), 5.95–5.80 (m, 2H), 5.15–5.01 (m, 2H), 2.91 (t, J = 6.1 Hz, 2H).

^{13}C NMR (126 MHz, $CDCl_3$): δ 137.7, 136.5, 132.8, 131.6, 130.7, 129.2, 128.7, 127.4, 126.3, 115.8, 37.0.

HRMS (ESI) m/z ($[M-H]^-$) calcd for $C_{13}H_{13}$: 169.1023. Found: 169.1008.

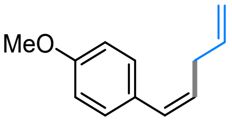

(*Z*)-1-Methoxy-4-(penta-1,4-dien-1-yl)benzene (37).

The title compound was prepared following the general procedure using (*Z*)-1-(2-bromovinyl)-4-methoxybenzene (32.0 mg, 0.150 mmol, 1.0 equiv), allyl acetate (30.0 mg, 0.300 mmol, 2.0 equiv). After purification by column chromatography (using 2% ethyl acetate in petroleum ether), the title compound was isolated in 67% yield (17.5 mg) as a colorless oil.

^{1}H NMR (500 MHz, $CDCl_3$): δ 7.21 (s, 2H), 6.87 (d, J = 8.7 Hz, 2H), 6.46 (d, J = 11.5 Hz, 1H), 5.92 (ddt, J = 16.2, 10.2, 6.0 Hz, 1H), 5.61 (dt, J = 11.5, 7.5 Hz, 1H), 5.18–5.01 (m, 2H), 3.81 (s, 3H), 3.06 (t, J

= 5.9 Hz, 2H).

^{13}C NMR (126 MHz, $CDCl_3$): δ 158.5, 137.0, 130.1, 130.0, 129.6, 128.1, 115.3, 113.7, 55.4, 32.9.

HRMS (ESI) m/z ($[M+Na]^+$) calcd for $C_{12}H_{14}NaO$: 197.0937. Found: 197.1141.

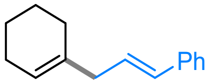

(*E*)-(3-(Cyclohex-1-en-1-yl)prop-1-en-1-yl)benzene (38).

The title compound was prepared following the general procedure using cyclohex-1-en-1-yl trifluoromethanesulfonate (34.5 mg, 0.150 mmol, 1.0 equiv), cinnamyl acetate (52.8 mg, 0.300 mmol, 2.0 equiv). After purification by column chromatography (using 2% ethyl acetate in petroleum ether), the title compound was isolated in 60% yield (17.8 mg) as a colorless oil. The spectral data were consistent with those which were reported in the literature[13] after comparison.

^{1}H NMR (500 MHz, $CDCl_3$): δ 7.37 (d, J = 7.3 Hz, 2H), 7.31 (t, J = 7.7 Hz, 2H), 7.21 (t, J = 7.3 Hz, 1H), 6.40 (d, J = 15.8 Hz, 1H), 6.23 (dt, J = 15.7, 7.1 Hz, 1H), 5.52 (s, 1H), 2.84 (d, J = 7.0 Hz, 2H), 2.10–1.93 (m, 4H), 1.72–1.53 (m, 4H).

^{13}C NMR (126 MHz, $CDCl_3$): δ 137.9, 136.6, 131.0, 129.1, 128.6, 127.0, 126.2, 122.3, 41.8, 28.6, 25.5, 23.1, 22.6.

HRMS (ESI) m/z ($[M-H]^-$) calcd for $C_{15}H_{17}$: 197.1336. Found: 197.1319.

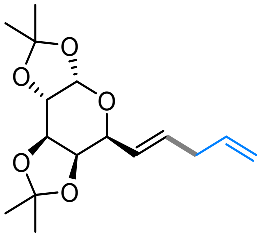

(3a*S*,5*S*,5a*R*,8a*R*,8b*S*)-2,2,7,7-Tetramethyl-5-((*E*)-penta-1,4-dien-1-yl)tetrahydro-5*H*-bis([1,3]dioxolo)[4,5-b:4',5'-d]pyran (39).

The title compound was prepared following the general procedure using (3a*S*,5*S*,5a*R*,8a*R*,8b*S*)-5-((*E*)-2-bromovinyl)-2,2,7,7-tetramethyltetrahydro-5*H*-bis([1,3]dioxolo)[4,5-*b*:4',5'-*d*]pyran (50.3 mg, 0.150 mmol, 1.0 equiv), allyl acetate (30.0 mg, 0.300 mmol, 2.0 equiv). After purification by column chromatography (using 5% ethyl acetate in petroleum ether), the title compound was isolated in 63% yield (27.9 mg) as a colorless oil.

^{1}H NMR (500 MHz, $CDCl_3$): δ 5.80 (dddd, J = 13.0, 8.3, 7.1, 3.5 Hz, 2H), 5.64 (dd, J = 15.6, 7.1 Hz, 1H), 5.55 (d, J = 5.0 Hz, 1H), 5.12–4.94 (m, 2H), 4.59 (dd, J = 7.9, 2.3 Hz, 1H), 4.29 (dd, J = 5.0, 2.4 Hz, 1H), 4.24 (d, J = 7.2 Hz, 1H), 4.17 (dd, J = 7.9, 2.0 Hz, 1H), 2.83 (t, J = 6.2 Hz, 2H), 1.53 (s, 3H), 1.46 (s, 3H), 1.34 (s, 3H), 1.33 (s, 3H).

^{13}C NMR (150 MHz, $CDCl_3$): δ 136.4, 132.7, 126.7, 115.8, 109.3, 108.5, 96.6, 73.7, 71.0, 70.5, 69.1, 36.7, 26.3, 26.1, 25.1, 24.5.

HRMS (ESI) m/z ($[M+H]^+$) calcd for $C_{16}H_{25}O_5$: 297.1697. Found: 297.1701.

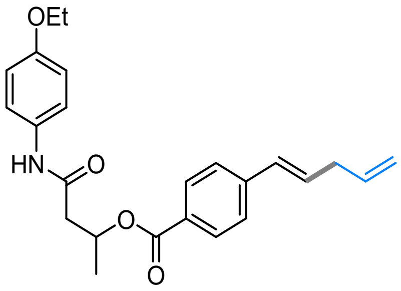

4-((4-Ethoxyphenyl)amino)-4-oxobutan-2-yl-(*E*)-4-(penta-1,4-dien-1-yl)benzoate (41).

The title compound was prepared following the general procedure using 4-((4-ethoxyphenyl)amino)-4-oxobutan-2-yl (*E*)-4-(2-bromovinyl)benzoate (64.8 mg, 0.150 mmol, 1.0 equiv), allyl acetate (30.0 mg, 0.300 mmol, 2.0 equiv). After purification by column chromatography (using 15% ethyl acetate in petroleum ether), the title compound was isolated in 73% yield (43.0 mg) as a white solid.

^{1}H NMR (500 MHz, $CDCl_3$): δ 7.94 (dt, J = 6.3, 4.8 Hz, 2H), 7.75 (s, 1H), 7.42–7.30 (m, 4H), 6.80 (d, J = 9.0 Hz, 2H), 6.51–6.19 (m, 2H), 5.99–5.77 (m, 1H), 5.54 (dd, J = 12.4, 6.2 Hz, 1H), 5.11 (ddd, J = 13.9, 12.0, 1.6 Hz, 1H), 3.97 (q, J = 7.0 Hz, 2H), 2.99 (td, J = 6.4, 1.2 Hz, 1H), 2.81 (dd, J = 14.5, 6.6 Hz, 1H), 2.66 (dd, J = 14.5, 5.8 Hz, 1H), 1.85 (d, J = 6.6 Hz, 2H), 1.49 (d, J = 6.3 Hz, 3H), 1.38 (t, J = 7.0 Hz, 3H).

^{13}C NMR (126 MHz, $CDCl_3$): δ 167.8, 166.0, 155.9, 142.5 (d, J = 18.5 Hz), 135.9, 132.4 (d, J = 28.9 Hz), 131.6 (d, J = 13.2 Hz), 130.8, 130.0 (d, J = 1.6 Hz), 128.6 (d, J = 7.4 Hz), 126.0, 122.0, 116.3, 114.8, 69.0, 63.8, 44.3, 37.2, 20.2, 14.9.

HRMS (ESI) m/z ($[M+H]^+$) calcd for $C_{24}H_{28}NO_4$: 394.2013. Found: 394.2014.

M.p.: 174-175 ºC.

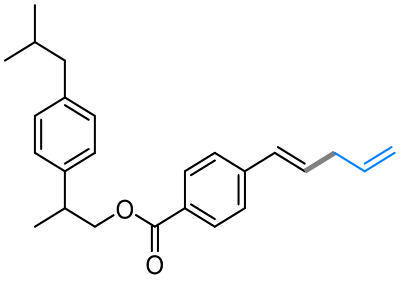

2-(4-Isobutylphenyl)propyl-(*E*)-4-(penta-1,4-dien-1-yl)benzoate (42).

The title compound was prepared following the general procedure using 2-(4-isobutylphenyl)propyl (*E*)-4-(2-bromovinyl)benzoate (60.1 mg, 0.150 mmol, 1.0 equiv), allyl acetate (30.0 mg, 0.300 mmol, 2.0 equiv). After purification by column chromatography (using 5% ethyl acetate in petroleum ether), the title compound was isolated in 89% yield (48.3 mg) as a

colorless oil.

^{1}H NMR (500 MHz, $CDCl_3$): δ 7.93 (d, J = 8.3 Hz, 2H), 7.39 (d, J = 8.3 Hz, 2H), 7.21 (d, J = 8.0 Hz, 2H), 7.12 (d, J = 7.9 Hz, 2H), 6.38 (ddd, J = 25.9, 15.8, 11.2 Hz, 2H), 5.98–5.83 (m, 1H), 5.24–5.01 (m, 2H), 4.39 (ddd, J = 27.7, 10.7, 7.1 Hz, 2H), 3.23 (dd, J = 14.0, 7.0 Hz, 1H), 3.00 (t, J = 5.9 Hz, 2H), 2.47 (d, J = 7.2 Hz, 2H), 1.87 (dt, J = 13.5, 6.8 Hz, 1H), 1.40 (d, J = 7.0 Hz, 3H), 0.91 (d, J = 6.6 Hz, 6H).

^{13}C NMR (150 MHz, $CDCl_3$): δ 166.5, 142.2, 140.5, 140.2, 136.0, 131.2, 130.2, 130.0, 129.3, 128.9, 127.1, 126.0, 116.2, 70.1, 45.2, 38.8, 37.2, 30.3, 22.5 (d, J = 3.0 Hz), 18.2.

HRMS (ESI) m/z ([M+Na]$^+$) calcd for $C_{25}H_{30}NaO_2$: 385.2138. Found: 385.2323.

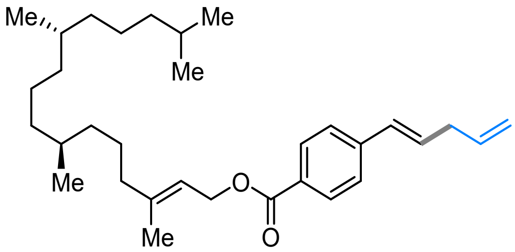

(7*S*,11*R*,*E*)-3,7,11,15-Tetramethylhexadec-2-en-1-yl 4-((*E*)-penta-1,4-dien-1-yl)benzoate (43).

The title compound was prepared following the general procedure using (7*S*,11*R*,*E*)-3,7,11,15-tetramethylhexadec-2-en-1-yl 4-((*E*)-2-bromovinyl)benzoate (75.7 mg, 0.150 mmol, 1.0 equiv), allyl acetate (30.0 mg, 0.300 mmol, 2.0 equiv). After purification by column chromatography (using 5% ethyl acetate in petroleum ether), the title compound was isolated in 86% yield (60.1 mg) as a colorless oil.

^{1}H NMR (500 MHz, $CDCl_3$): δ 7.97 (d, J = 8.4 Hz, 2H), 7.39 (d, J = 8.3 Hz, 2H), 6.38 (ddd, J = 26.3, 15.9, 11.2 Hz, 2H), 5.90 (ddt, J = 16.6, 10.1, 6.4 Hz, 1H), 5.46 (td, J = 7.0, 1.1 Hz, 1H), 5.15–5.06 (m, 2H), 4.83 (d, J = 7.0 Hz, 2H), 2.99 (td, J = 6.4, 1.3 Hz, 2H), 2.07–1.99 (m, 2H), 1.75 (s, 3H), 1.52 (dt, J = 13.3, 6.6 Hz, 1H), 1.45–1.34 (m, 4H), 1.32–1.20 (m, 10H), 1.16–1.10 (m, 3H), 0.85 (dd, J = 12.1, 6.2 Hz, 13H).

^{13}C NMR (126 MHz, $CDCl_3$): δ 166.7, 142.9, 142.1, 136.0, 131.2, 130.3, 130.1, 129.1, 126.0, 118.4, 116.3, 62.0, 40.0, 39.5, 37.6, 37.5, 37.4, 37.2, 36.8, 32.9, 32.8, 28.1, 25.2, 24.9, 24.6, 22.9, 22.8, 19.9 (d, J = 3.3 Hz), 16.6.

HRMS (ESI) m/z ([M+Na]$^+$) calcd for $C_{32}H_{50}NaO_2$: 489.3703. Found: 489.3702.

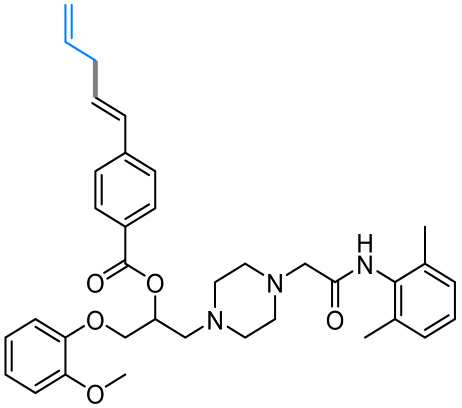

1-(4-(2-((2,6-Dimethylphenyl)amino)-2-oxoethyl)piperazin-1-yl)-3-(2-methoxyphenoxy)propan-2-yl-(*E*)-4-(penta-1,4-dien-1-

yl)benzoate (44).

The title compound was prepared following the general procedure using 1-(4-(2-((2,6-dimethylphenyl)amino)-2-oxoethyl)piperazin-1-yl)-3-(2-methoxyphenoxy)propan-2-yl-(*E*)-4-(2-bromovinyl)benzoate (95.4 mg, 0.150 mmol, 1.0 equiv), allyl acetate (30.0 mg, 0.300 mmol, 2.0 equiv). After purification by column chromatography (using 20% ethyl acetate in petroleum ether), the title compound was isolated in 68% yield (60.9 mg) as a yellow solid.

^{1}H NMR (500 MHz, $CDCl_3$): δ 8.66 (s, 1H), 7.94 (d, *J* = 8.3 Hz, 2H), 7.39 (d, *J* = 8.3 Hz, 2H), 7.09 (q, *J* = 4.3 Hz, 3H), 7.04–7.00 (m, 1H), 6.98–6.84 (m, 4H), 6.49 – 6.31 (m, 2H), 5.96–5.83 (m, 1H), 5.58–5.51 (m, 1H), 5.15–5.08 (m, 1H), 4.39–4.30 (m, 2H), 3.79 (s, 3H), 3.18 (s, 2H), 3.07–2.93 (m, 2H), 2.87–2.79 (m, 2H), 2.69 (s, 8H), 2.22 (s, 6H).

^{13}C NMR (126 MHz, $CDCl_3$): δ 168.6, 166.0, 150.3, 148.5, 142.4, 136.0, 135.1, 133.8, 131.4, 130.18, 130.16, 128.6, 128.4, 127.3, 126.0, 122.3, 121.1, 116.3, 115.6, 112.7, 70.8, 69.8, 61.8, 58.1, 56.1, 54.0 (d, *J* = 12.1 Hz), 37.2, 29.8, 18.8.

HRMS (ESI) m/z ($[M+H]^+$) calcd for $C_{36}H_{44}N_3O_5$: 598.3275. Found: 598.3275.

M.p.: 75-76 ºC.

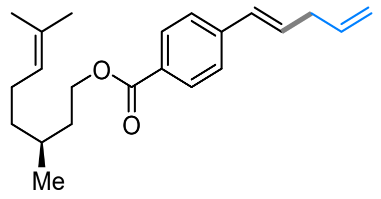

(*S*)-3,7-Dimethyloct-6-en-1-yl-(*E*)-4-(penta-1,4-dien-1-yl)benzoate (45).

The title compound was prepared following the general procedure using (*S*)-3,7-dimethyloct-6-en-1-yl (*E*)-4-(2-bromovinyl)benzoate (54.8 mg, 0.150 mmol, 1.0 equiv), allyl acetate (30.0 mg, 0.300 mmol, 2.0 equiv). After purification by column chromatography (using 5% ethyl acetate in petroleum ether), the title compound was isolated in 81% yield (39.6 mg) as a colorless oil.

^{1}H NMR (500 MHz, $CDCl_3$): δ 7.97 (d, *J* = 8.2 Hz, 2H), 7.40 (d, *J* = 8.2 Hz, 2H), 6.38 (ddd, *J* = 26.4, 15.8, 11.2 Hz, 2H), 5.90 (ddt, *J* = 16.7, 10.1, 6.4 Hz, 1H), 5.12 (dd, *J* = 25.5, 5.8 Hz, 3H), 4.42–4.28 (m, 2H), 2.99 (t, *J* = 6.0 Hz, 2H), 2.09–1.95 (m, 2H), 1.82 (dt, *J* = 12.7, 7.1 Hz, 1H), 1.68 (s, 3H), 1.61 (s, 3H), 1.56 (dd, *J* = 14.0, 6.9 Hz, 1H), 1.41 (ddd, *J* = 21.3, 9.5, 6.0 Hz, 1H), 1.30–1.21 (m, 2H), 0.97 (d, *J* = 6.6 Hz, 3H).

^{13}C NMR (126 MHz, $CDCl_3$): δ 166.6, 142.1, 136.0, 131.5, 131.2, 130.2, 130.0, 129.0, 126.0, 124.7, 116.2, 63.5, 37.1 (d, J = 7.8 Hz), 35.6, 29.7, 25.8, 25.5, 19.6, 17.8.

HRMS (ESI) m/z ([M+H]$^+$) calcd for $C_{22}H_{31}O_2$: 327.2319. Found: 327.2312.

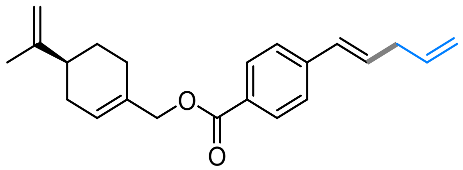

(*S*)-(4-(Prop-1-en-2-yl)cyclohex-1-en-1-yl)methyl-(*E*)-4-(penta-1,4-dien-1-yl)benzoate (46).

The title compound was prepared following the general procedure using (*S*)-(4-(prop-1-en-2-yl)cyclohex-1-en-1-yl)methyl (*E*)-4-(2-bromovinyl)benzoate (54.2 mg, 0.150 mmol, 1.0 equiv), allyl acetate (30.0 mg, 0.300 mmol, 2.0 equiv). After purification by column chromatography (using 5% ethyl acetate in petroleum ether), the title compound was isolated in 85% yield (41.1 mg) as a colorless oil.

^{1}H NMR (500 MHz, $CDCl_3$): δ 7.98 (d, J = 8.3 Hz, 2H), 7.40 (d, J = 8.3 Hz, 2H), 6.45 (d, J = 16.0 Hz, 1H), 6.36 (dt, J = 15.8, 6.4 Hz, 1H), 5.90 (ddt, J = 16.7, 10.1, 6.4 Hz, 1H), 5.84 (s, 1H), 5.17–5.05 (m, 2H), 4.73 (dd, J = 10.2, 6.1 Hz, 4H), 2.99 (t, J = 6.0 Hz, 2H), 2.20–2.14 (m, 3H), 2.05–1.83 (m, 3H), 1.75 (s, 3H), 1.60–1.49 (m, 1H).

^{13}C NMR (126 MHz, $CDCl_3$): δ 166.4, 149.7, 142.2, 136.0, 132.9, 131.2, 130.2, 130.1, 128.8, 126.0, 125.7, 116.2, 108.9, 68.9, 41.0, 37.2, 35.0, 30.6, 27.5, 26.6.

HRMS (ESI) m/z ([M+H]$^+$) calcd for $C_{22}H_{27}O_2$: 323.2006. Found: 323.2003.

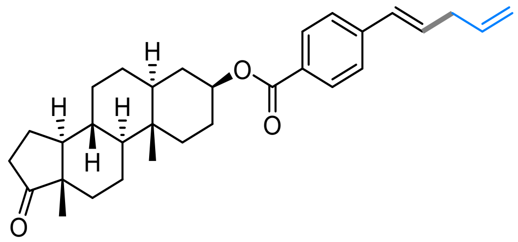

(3*S*,5*S*,8*R*,9*S*,10*S*,13*S*,14*S*)-10,13-Dimethyl-17-oxohexadecahydro-1*H*-cyclopenta[*a*]phenanthren-3-yl 4-((*E*)-penta-1,4-dien-1-yl)benzoate (47).

The title compound was prepared following the general procedure using (3*S*,5*S*,8*R*,9*S*,10*S*,13*S*,14*S*)-10,13-dimethyl-17-oxohexadecahydro-1*H*-cyclopenta[*a*]phenanthren-3-yl 4-((*E*)-2-bromovinyl)benzoate (75.0 mg, 0.150 mmol, 1.0 equiv), allyl acetate (30.0 mg, 0.300 mmol, 2.0 equiv). After purification by column chromatography (using 5% ethyl acetate in petroleum ether), the title compound was isolated in 84% yield (57.9 mg) as a white solid.

^{1}H NMR (500 MHz, $CDCl_3$): δ 7.93 ((d, J = 8.3 Hz, 2H)), 7.37 (d, J = 8.3 Hz, 2H), 6.45–6.29 (m, 2H), 5.93–5.82 (m, 1H), 5.15–5.01 (m, 2H), 4.90 (dt, J = 16.1, 5.5 Hz, 1H), 2.96 (t, J = 6.4 Hz, 2H), 2.41 (dd, J = 19.3, 8.6 Hz, 1H), 2.04 (dd, J = 16.5, 6.5 Hz, 1H), 1.91 (dd, J = 12.0, 6.0 Hz, 2H), 1.79 (dd, J = 10.3, 7.0 Hz, 4H), 1.63 (ddd, J = 12.1, 5.1, 2.4 Hz, 2H), 1.55–1.44 (m, 3H), 1.37–1.26 (m, 6H), 1.11–1.05 (m, 1H), 0.99–0.96 (m, 1H), 0.87 (s, 3H), 0.84 (s, 3H), 0.75–0.69 (m, 1H).

^{13}C NMR (126 MHz, $CDCl_3$): δ 166.0, 142.0, 135.9, 132.3, 131.0, 130.1, 129.9, 129.3, 125.8, 116.2, 74.0, 54.4, 51.4, 47.8, 44.7, 37.1, 36.8, 35.9, 35.7, 35.1, 34.1, 31.6, 30.9, 28.4, 27.6, 21.8, 20.5, 13.9, 12.3.

HRMS (ESI) m/z ($[M+H]^+$) calcd for $C_{31}H_{41}O_3$: 461.3050. Found: 461.3051.

M.p.: 137-139 ºC.

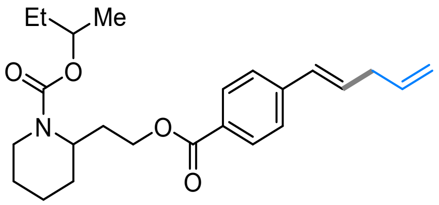

***sec*-Butyl-(*E*)-2-(2-((4-(penta-1,4-dien-1-yl)benzoyl)oxy)ethyl)piperidine-1-carboxylate (48)**.

The title compound was prepared following the general procedure using *sec*-butyl (*E*)-2-(2-((4-(2-bromovinyl)benzoyl)oxy)ethyl)piperidine-1-carboxylate (65.7 mg, 0.150 mmol, 1.0 equiv), allyl acetate (30.0 mg, 0.300 mmol, 2.0 equiv). After purification by column chromatography (using 20% ethyl acetate in petroleum ether), the title compound was isolated in 73% yield (43.7 mg) as a colorless oil.

^{1}H NMR (500 MHz, $CDCl_3$): δ 7.96 (d, J = 8.2 Hz, 2H), 7.39 (d, J = 8.3 Hz, 2H), 6.39 (ddd, J = 22.3, 15.9, 5.0 Hz, 2H), 5.95–5.83 (m, 1H), 5.18–5.03 (m, 2H), 4.74 (ddd, J = 12.6, 6.3, 1.6 Hz, 1H), 4.52 (s, 1H), 4.32–4.29 (m, 2H), 4.08 (s, 1H), 2.99 (t, J = 6.4 Hz, 2H), 2.86 (t, J = 13.2 Hz, 1H), 2.23–2.17 (m, 1H), 1.90–1.85 (m, 1H), 1.70–1.56 (m, 8H), 1.17 (d, J = 6.3 Hz, 3H), 0.87 (s, 3H).

^{13}C NMR (126 MHz, $CDCl_3$): δ 166.5, 155.7 (d, J = 4.0 Hz), 142.2, 136.0, 131.2, 130.2, 130.0, 128.8, 126.0, 116.2, 73.1, 62.7 (d, J = 4.9 Hz), 48.2 (d, J = 4.0 Hz), 39.1, 37.2, 29.8, 29.2, 25.6, 22.8, 19.9, 19.2, 9.8 (d, J = 3.1Hz).

HRMS (ESI) m/z ($[M+H]^+$) calcd for $C_{24}H_{34}NO_4$: 400.2482. Found: 400.2485.

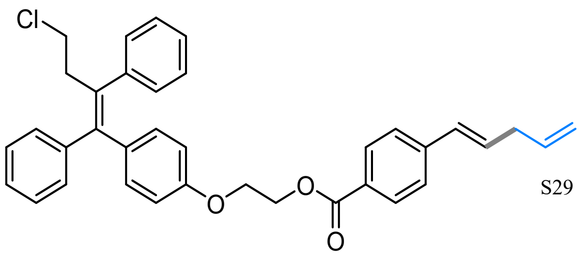

2-(4-((*Z*)-4-Chloro-1,2-diphenylbut-1-en-1-yl)phenoxy)ethyl-4-((*E*)-penta-1,4-dien-1-

yl)benzoate (49).

The title compound was prepared following the general procedure using 2-(4-((*Z*)-4-chloro-1,2-diphenylbut-1-en-1-yl)phenoxy)ethyl 4-((*E*)-2-bromovinyl)benzoate (88.2 mg, 0.150 mmol, 1.0 equiv), allyl acetate (30.0 mg, 0.300 mmol, 2.0 equiv). After purification by column chromatography (using 10% ethyl acetate in petroleum ether), the title compound was isolated in 80% yield (65.8 mg) as a colorless solid.

^{1}H NMR (500 MHz, $CDCl_3$): δ 7.96 (d, J = 8.4 Hz, 2H), 7.42–7.36 (m, 4H), 7.33–7.28 (m, 3H), 7.21 (d, J = 6.5 Hz, 2H), 7.16 (d, J = 7.2 Hz, 3H), 6.82 (d, J = 8.8 Hz, 2H), 6.61 (d, J = 8.8 Hz, 2H), 6.39 (ddd, J = 24.1, 15.9, 11.2 Hz, 2H), 5.92 (ddt, J = 16.7, 10.1, 6.4 Hz, 1H), 5.13 (ddd, J = 14.3, 13.0, 1.5 Hz, 2H), 4.60–4.55 (m, 2H), 4.20–4.14 (m, 2H), 3.43 (t, J = 7.4 Hz, 2H), 3.00 (t, J = 7.0 Hz, 2H), 2.94 (t, J = 7.5 Hz, 2H).

^{13}C NMR (126 MHz, $CDCl_3$): δ 166.4, 156.9, 143.0, 142.4, 141.8, 141.0, 135.9, 135.5, 135.4, 131.9, 131.4, 130.2, 130.1, 129.6, 129.5, 128.5, 128.4, 127.1, 126.7, 126.0, 116.3, 113.7, 65.9, 63.3, 43.0, 38.7, 37.2, 29.8.

HRMS (ESI) m/z ($[M+H]^+$) calcd for $C_{36}H_{34}ClO_3$: 549.2191. Found: 549.2192.

M.p.: 55-56 ºC.

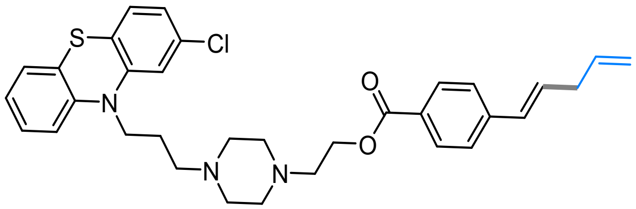

2-(4-(3-(2-Chloro-10*H*-phenothiazin-10-yl)propyl)piperazin-1-yl)ethyl-(*E*)-4-(penta-1,4-dien-1-yl)benzoate (50).

The title compound was prepared following the general procedure using 2-(4-(3-(2-chloro-10H-phenothiazin-10-yl)propyl)piperazin-1-yl)ethyl (E)-4-(2-bromovinyl)benzoate (91.9 mg, 0.150 mmol, 1.0 equiv), allyl acetate (30.0 mg, 0.300 mmol, 2.0 equiv). After purification by column chromatography (using 20% ethyl acetate in petroleum ether), the title compound was isolated in 62% yield (53.4 mg) as a yellow oil.

^{1}H NMR (500 MHz, $CDCl_3$): δ 7.95 (d, J = 8.2 Hz, 2H), 7.40 (d, J = 8.2 Hz, 2H), 7.18–7.07 (m, 2H), 7.01 (d, J = 8.1 Hz, 1H), 6.95–6.82 (m, 4H), 6.52–6.30 (m, 2H), 5.90 (ddt, J = 16.7, 10.0, 6.5 Hz, 1H),

5.17–5.04 (m, 2H), 4.43 (t, *J* = 5.7 Hz, 2H), 3.90 (t, *J* = 6.8 Hz, 2H), 2.99 (t, *J* = 6.0 Hz, 2H), 2.77 (t, *J* = 5.7 Hz, 2H), 2.51 (dd, *J* = 33.7, 26.9 Hz, 10H), 1.98–1.91 (m, 2H).

^{13}C NMR (126 MHz, $CDCl_3$): δ 166.4, 146.6, 144.6, 136.0, 134.0, 133.4, 131.4, 130.2, 130.1, 128.7, 128.0, 127.6, 127.5, 126.0, 124.9, 123.7, 123.0, 122.4, 116.3, 115.99, 115.96, 62.7, 56.7, 55.6, 53.3, 45.5, 37.2, 32.1, 29.8.

HRMS (ESI) m/z ($[M+H]^+$) calcd for $C_{33}H_{37}ClN_3O_2S$: 574.2290. Found: 574.2286.

IV. Vinylation of *β*-Elemene

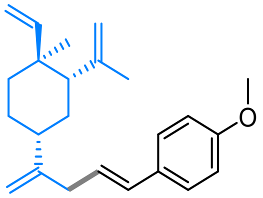

1-Methoxy-4-((*E*)-4-((1*R*,3*S*,4S)-4-methyl-3-(prop-1-en-2-yl)-4-vinylcyclohexyl)penta-1,4-dien-1-yl)benzene (51).

The title compound was prepared following the general procedure using (*E*)-1-(2-bromovinyl)-4-methoxybenzene (32.0 mg, 0.150 mmol, 1.0 equiv), 2-((1*R*,3*S*,4*S*)-4-methyl-3-(prop-1-en-2-yl)-4-vinylcyclohexyl)allyl acetate (78.7 mg, 0.300 mmol, 2.0 equiv). After purification by column chromatography (using 2% ethyl acetate in petroleum ether), the title compound was isolated in 86% yield (43.3 mg) as a colorless oil.

^{1}H NMR (500 MHz, $CDCl_3$): δ 7.31 (d, *J* = 8.7 Hz, 2H), 6.85 (d, *J* = 8.7 Hz, 2H), 6.37 (d, *J* = 15.8 Hz, 1H), 6.08 (dt, *J* = 15.7, 7.1 Hz, 1H), 5.83 (dd, *J* = 17.4, 10.9 Hz, 1H), 4.92 (dd, *J* = 8.1, 1.2 Hz, 1H), 4.89 (s, 1H), 4.87 (s, 1H), 4.83 (dd, *J* = 5.0, 1.3 Hz, 2H), 4.61 (s, 1H), 3.81 (s, 3H), 2.95 (d, *J* = 7.0 Hz, 2H), 2.02 (dd, *J* = 11.8, 4.3 Hz, 2H), 1.72 (s, 3H), 1.64–1.59 (m, 2H), 1.54–1.42 (m, 4H), 1.02 (s, 3H).

^{13}C NMR (126 MHz, $CDCl_3$): δ 158.9, 153.4, 150.4, 147.8, 130.8, 130.6, 127.3, 126.6, 114.1, 112.3, 110.0, 108.8, 55.4, 52.9, 44.3, 40.1, 40.0, 38.8, 33.4, 27.3, 24.9, 16.8.

HRMS (ESI) m/z ($[M-H]^-$) calcd for $C_{24}H_{31}O$: 335.2380. Found: 335.2380.

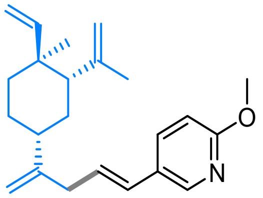

2-Methoxy-5-((*E*)-4-((1*R*,3*S*,4*S*)-4-methyl-3-(prop-1-en-2-yl)-4-vinylcyclohexyl)penta-1,4-dien-1-yl)pyridine (52).

The title compound was prepared following the general procedure using (*E*)-5-(2-bromovinyl)-2-methoxypyridine (32.1 mg, 0.150 mmol, 1.0 equiv), 2-

((1*R*,3*S*,4*S*)-4-methyl-3-(prop-1-en-2-yl)-4-vinylcyclohexyl)allyl acetate (78.7 mg, 0.300 mmol, 2.0 equiv). After purification by column chromatography (using 5% ethyl acetate in petroleum ether), the title compound was isolated in 73% yield (36.9 mg) as a colorless oil.

^{1}H NMR (500 MHz, $CDCl_3$): δ 8.08 (t, J = 5.5 Hz, 1H), 7.64 (dd, J = 8.6, 2.4 Hz, 1H), 6.69 (d, J = 8.6 Hz, 1H), 6.34 (d, J = 15.9 Hz, 1H), 6.10 (dt, J = 15.8, 7.0 Hz, 1H), 5.81 (dd, J = 17.3, 11.0 Hz, 1H), 4.99–4.75 (m, 5H), 4.59 (s, 1H), 3.93 (s, 3H), 2.95 (d, J = 6.9 Hz, 2H), 1.99 (ddd, J = 15.9, 11.2, 5.0 Hz, 2H), 1.71 (s, 3H), 1.65–1.56 (m, 3H), 1.50–1.41 (m, 3H), 1.01 (s, 3H).

^{13}C NMR (126 MHz, $CDCl_3$): δ 163.4, 153.1, 150.3, 147.8, 145.1, 135.5, 128.2, 127.5, 127.0, 112.3, 110.9, 110.1, 109.0, 53.6, 52.9, 44.4, 40.0 (d, J = 11.8 Hz), 38.8, 33.4, 29.8, 27.3, 24.9, 16.8.

HRMS (ESI) m/z ($[M+H]^+$) calcd for $C_{23}H_{32}NO$: 338.2478. Found: 338.2475.

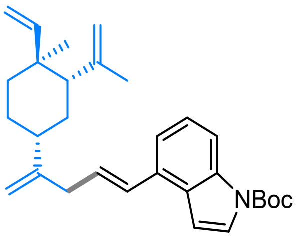

***tert*-Butyl-4-((*E*)-4-((1*R*,3*S*,4*S*)-4-methyl-3-(prop-1-en-2-yl)-4-vinylcyclohexyl)penta-1,4-dien-1-yl)-1*H*-indole-1-carboxylate (53)**. The title compound was prepared following the general procedure using *tert*-butyl (*E*)-4-(2-bromovinyl)-1*H*-indole-1-carboxylate (48.3 mg, 0.150 mmol, 1.0 equiv), 2-((1*R*,3*S*,4*S*)-4-methyl-3-(prop-1-en-2-yl)-4-vinylcyclohexyl)allyl acetate (78.7 mg, 0.300 mmol, 2.0 equiv). After purification by column chromatography (using 5% ethyl acetate in petroleum ether), the title compound was isolated in 75% yield (50.1 mg) as a colorless oil.

^{1}H NMR (500 MHz, $CDCl_3$): δ 8.01 (d, J = 7.7 Hz, 1H), 7.59 (d, J = 3.6 Hz, 1H), 7.32 (d, J = 7.5 Hz, 1H), 7.24 (t, J = 3.9 Hz, 1H), 6.72 (d, J = 3.3 Hz, 2H), 6.32 (dt, J = 15.7, 7.1 Hz, 1H), 5.80 (dd, J = 17.4, 10.9 Hz, 1H), 4.93–4.77 (m, 5H), 4.58 (s, 1H), 3.02 (d, J = 7.0 Hz, 2H), 2.00 (dd, J = 12.0, 3.9 Hz, 2H), 1.70 (s, 3H), 1.65 (s, 12H), 1.46 (ddd, J = 9.0, 7.8, 2.6 Hz, 3H), 1.00 (s, 3H).

^{13}C NMR (126 MHz, $CDCl_3$): δ 153.2, 150.4, 147.8, 135.6, 134.2, 130.4 (d, J = 8.5 Hz), 128.6, 125.9, 124.5, 119.4, 115.4, 114.7, 113.9, 112.3, 110.0, 109.1, 105.6, 83.8, 52.9, 44.4, 40.0 (d, J = 12.6 Hz), 39.2, 33.4, 29.9, 28.3, 27.4, 24.9, 16.8.

HRMS (ESI) m/z ($[M-H]^-$) calcd for $C_{30}H_{38}NO_2$: 444.2908. Found: 444.2908.

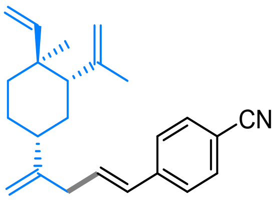

4-((*E*)-4-((1*R*,3*S*,4*S*)-4-Methyl-3-(prop-1-en-2-yl)-4-

vinylcyclohexyl)penta-1,4-dien-1-yl)benzonitrile (54).

The title compound was prepared following the general procedure using (*E*)-4-(2-bromovinyl)benzonitrile (31.2 mg, 0.150 mmol, 1.0 equiv), 2-((1*R*,3*S*,4*S*)-4-methyl-3-(prop-1-en-2-yl)-4-vinylcyclohexyl)allyl acetate (78.7 mg, 0.300 mmol, 2.0 equiv). After purification by column chromatography (using 5% ethyl acetate in petroleum ether), the title compound was isolated in 80% yield (39.7 mg) as a colorless oil.

^{1}H NMR (500 MHz, $CDCl_3$): δ 7.75 (d, J = 8.3 Hz, 2H), 7.43 (d, J = 8.3 Hz, 2H), 6.36 (ddd, J = 30.6, 15.8, 11.4 Hz, 2H), 5.81 (dd, J = 17.3, 11.0 Hz, 1H), 4.94–4.87 (m, 3H), 4.82 (d, J = 5.9 Hz, 2H), 4.59 (s, 1H), 2.99 (d, J = 6.8 Hz, 2H), 2.01 (dt, J = 18.0, 10.4 Hz, 2H), 1.71 (s, 3H), 1.64–1.56 (m, 3H), 1.52–1.42 (m, 3H), 1.01 (s, 3H).

^{13}C NMR (126 MHz, $CDCl_3$): δ 169.1, 152.7, 150.3, 147.8, 141.5, 131.6, 130.5, 127.9, 126.3, 112.3, 110.1, 109.3, 100.1, 52.9, 44.5, 40.0 (d, J = 9.9 Hz), 38.8, 33.4, 29.8, 27.3, 24.9, 16.8.

HRMS (ESI) m/z ([M+H]$^+$) calcd for $C_{24}H_{30}N$: 332.2373. Found: 332.2378.

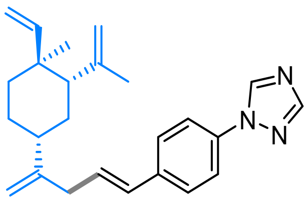

1-(4-((*E*)-4-((1*R*,3*S*,4*S*)-4-Methyl-3-(prop-1-en-2-yl)-4-vinylcyclohexyl)penta-1,4-dien-1-yl)phenyl)-1*H*-1,2,4-triazole (55).

The title compound was prepared following the general procedure using (*E*)-1-(4-(2-bromovinyl)phenyl)-1*H*-1,2,4-triazole (37.5 mg, 0.150 mmol, 1.0 equiv), 2-((1*R*,3*S*,4*S*)-4-methyl-3-(prop-1-en-2-yl)-4-vinylcyclohexyl)allyl acetate (78.7 mg, 0.300 mmol, 2.0 equiv). After purification by column chromatography (using 5% ethyl acetate in petroleum ether), the title compound was isolated in 77% yield (43.1 mg) as a colorless oil.

^{1}H NMR (500 MHz, $CDCl_3$): δ 8.54 (s, 1H), 8.10 (s, 1H), 7.61 (d, J = 8.5 Hz, 2H), 7.49 (d, J = 8.6 Hz, 2H), 6.44 (d, J = 15.8 Hz, 1H), 6.29 (dt, J = 15.8, 7.0 Hz, 1H), 5.82 (dd, J = 17.3, 11.0 Hz, 1H), 4.97–4.87 (m, 3H), 4.83 (d, J = 1.4 Hz, 2H), 4.60 (s, 1H), 2.99 (d, J = 6.9 Hz, 2H), 2.06–1.97 (m, 2H), 1.71 (s, 3H), 1.64–1.58 (m, 3H), 1.52–1.42 (m, 3H), 1.01 (s, 3H).

^{13}C NMR (126 MHz, $CDCl_3$): δ 152.8, 152.7, 150.3, 147.8, 140.8, 137.9, 135.7, 130.5, 130.1, 127.4, 120.3, 112.3, 110.1, 109.3, 52.9, 44.5, 40.1, 40.0, 38.8, 33.4, 27.3, 24.9, 16.8.

HRMS (ESI) m/z ([M+H]$^+$) calcd for $C_{25}H_{32}N_3$: 374.2591. Found: 374.2590.

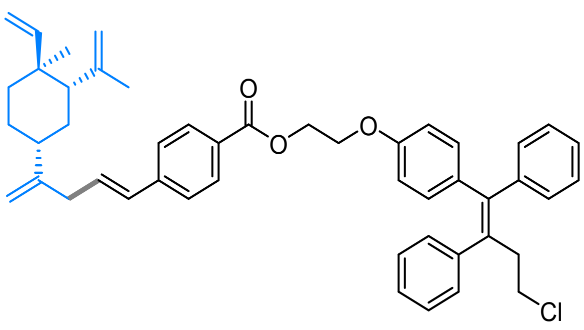

2-(4-((*Z*)-4-Chloro-1,2-diphenylbut-1-en-1-yl)phenoxy)ethyl-4-((*E*)-4-((1*R*,3*S*,4*S*)-4-methyl-3-(prop-1-en-2-yl)-4-vinylcyclohexyl)penta-1,4-dien-1-yl)benzoate (56).

The title compound was prepared following the general procedure using 2-(4-((*Z*)-4-chloro-1,2-diphenylbut-1-en-1-yl)phenoxy)ethyl 4-((*E*)-2-bromovinyl)benzoate (88.2 mg, 0.150 mmol, 1.0 equiv), 2-((1*R*,3*S*,4*S*)-4-methyl-3-(prop-1-en-2-yl)-4-vinylcyclohexyl)allyl acetate (78.7 mg, 0.300 mmol, 2.0 equiv). After purification by column chromatography (using 5% ethyl acetate in petroleum ether), the title compound was isolated in 81% yield (86.4 mg) as a colorless oil.

^{1}H NMR (500 MHz, CDCl$_3$): δ 7.96 (d, J = 8.2 Hz, 2H), 7.42–7.36 (m, 4H), 7.31 (d, J = 6.2 Hz, 3H), 7.23–7.19 (m, 2H), 7.16 (d, J = 7.2 Hz, 3H), 6.82 (d, J = 8.7 Hz, 2H), 6.61 (d, J = 8.7 Hz, 2H), 6.51–6.31 (m, 2H), 5.86–5.80 (m, 1H), 5.11 (d, J = 13.8 Hz, 1H), 4.92 (s, 1H), 4.91 (s, 1H), 4.84 (d, J = 6.9 Hz, 2H), 4.61 (s, 1H), 4.59–4.56 (m, 2H), 4.20–4.14 (m, 2H), 3.43 (t, J = 7.4 Hz, 2H), 3.00 (d, J = 8.7 Hz, 2H), 2.95–2.91 (m, 2H), 2.05–2.00 (m, 2H), 1.73 (s, 3H), 1.62 (dd, J = 7.5, 3.3 Hz, 2H), 1.51–1.45 (m, 4H), 1.03 (s, 3H).

^{13}C NMR (126 MHz, CDCl$_3$): δ 166.4, 156.9, 152.6, 150.2, 148.9, 147.7, 142.9, 142.4, 141.7, 141.0, 135.4 (d, J = 5.3 Hz), 131.93, 131.86, 130.6, 130.2, 129.6, 129.5, 128.5, 128.3, 127.1, 126.7, 126.0, 113.7, 113.1, 112.3, 110.1, 109.3, 79.5, 65.9, 63.3, 52.8, 44.4, 42.3, 41.6, 40.0 (d, J = 9.6 Hz), 38.7 (d, J = 14.8 Hz), 33.3, 29.8, 27.3, 24.9.

HRMS (ESI) m/z ([M+Na]$^+$) calcd for $C_{48}H_{51}ClNaO_3$: 733.3419. Found: 733.3482.

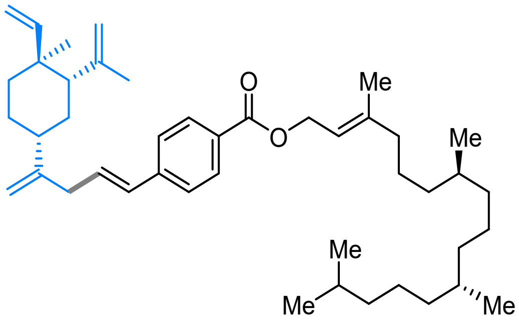

(7*S*,11*R*,*E*)-3,7,11,15-Tetramethylhexadec-2-en-1-yl-4-((*E*)-4-((1*R*,3*S*,4*S*)-4-methyl-3-(prop-1-en-2-yl)-4-vinylcyclohexyl)penta-1,4-dien-1-yl)benzoate (57).

The title compound was prepared following the

general procedure using (7*S*,11*R*,*E*)-3,7,11,15-tetramethylhexadec-2-en-1-yl 4-((*E*)-2-bromovinyl)benzoate (75.8 mg, 0.150 mmol, 1.0 equiv), 2-((1*R*,3*S*,4*S*)-4-methyl-3-(prop-1-en-2-yl)-4-vinylcyclohexyl)allyl acetate (78.7 mg, 0.300 mmol, 2.0 equiv). After purification by column chromatography (using 5% ethyl acetate in petroleum ether), the title compound was isolated in 78% yield (73.6 mg) as a colorless oil.

^{1}H NMR (500 MHz, $CDCl_3$): δ 7.98 (d, J = 8.4 Hz, 2H), 7.40 (d, J = 8.4 Hz, 2H), 6.54–6.26 (m, 2H), 5.82 (dd, J = 17.3, 11.0 Hz, 1H), 5.46 (dd, J = 7.0, 6.0 Hz, 1H), 4.93–4.89 (m, 2H), 4.83 (dd, J = 5.9, 4.0 Hz, 3H), 4.60 (s, 1H), 3.20 (dt, J = 13.3, 5.0 Hz, 1H), 2.99 (d, J = 6.8 Hz, 2H), 2.07–1.98 (m, 5H), 1.95–1.89 (m, 2H), 1.75 (s, 3H), 1.71 (s, 3H), 1.63–1.57 (m, 5H), 1.48 (ddd, J = 13.1, 9.9, 5.1 Hz, 8H), 1.16–1.12 (m, 5H), 1.09–1.05 (m, 5H), 1.01 (s, 3H), 0.86 (d, J = 5.5 Hz, 12H).

^{13}C NMR (126 MHz, $CDCl_3$): δ 166.7, 152.7, 150.3, 147.7, 142.9, 142.1, 131.7, 130.7, 130.1, 129.0, 126.0, 118.4, 112.3, 110.1, 109.3, 62.0, 55.9, 52.9, 44.5, 40.1, 39.8, 39.5, 38.8, 37.6, 37.5, 37.4, 36.8, 35.1, 33.4, 32.9 (d, J = 15.3 Hz), 29.8, 28.1, 27.3, 25.6, 25.2, 24.9, 24.6, 22.8 (d, J = 11.7 Hz), 19.9 (d, J = 3.7 Hz), 16.7 (d, J = 18.4 Hz), 14.3.

HRMS (ESI) m/z ($[M+H]^+$) calcd for $C_{44}H_{69}O_2$: 629.5292. Found: 629.5291.

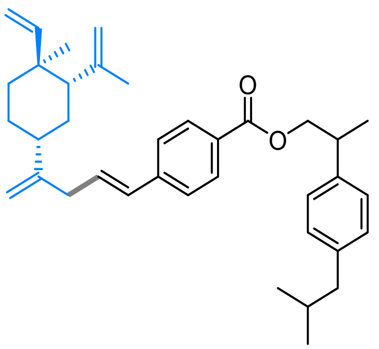

2-(4-Isobutylphenyl)propyl-4-((*E*)-4-((1*R*,3*S*,4*S*)-4-methyl-3-(prop-1-en-2-yl)-4-vinylcyclohexyl)penta-1,4-dien-1-yl)benzoate (58).

The title compound was prepared following the general procedure using 2-(4-isobutylphenyl)propyl (*E*)-4-(2-bromovinyl)benzoate (60.2 mg, 0.150 mmol, 1.0 equiv), 2-((1*R*,3*S*,4*S*)-4-methyl-3-(prop-1-en-2-yl)-4-vinylcyclohexyl)allyl acetate (78.7 mg, 0.300 mmol, 2.0 equiv). After purification by column chromatography (using 5% ethyl acetate in petroleum ether), the title compound was isolated in 86% yield (67.6 mg) as a colorless oil.

^{1}H NMR (500 MHz, $CDCl_3$): δ 7.91 (d, J = 7.9 Hz, 2H), 7.38 (d, J = 8.0 Hz, 2H), 7.19 (d, J = 7.7 Hz,

2H), 7.10 (d, J = 7.7 Hz, 2H), 6.49–6.30 (m, 2H), 5.82 (d, J = 6.4 Hz, 1H), 5.01 (s, 1H), 4.93–4.92 (m, 1H), 4.89 (s, 1H), 4.83 (s, 2H), 4.58–4.57 (m, 1H), 4.42–4.31 (m, 2H), 3.21 (dd, J = 13.9, 7.0 Hz, 1H), 2.99 (d, J = 6.7 Hz, 2H), 2.45 (d, J = 7.1 Hz, 2H), 2.09 (s, 3H), 2.01 (d, J = 6.8 Hz, 2H), 1.85 (dt, J = 13.3, 6.7 Hz, 1H), 1.60 (s, 2H), 1.47 (s, 4H), 1.38 (d, J = 6.8 Hz, 3H), 0.99 (s, 3H), 0.90 (d, J = 6.6 Hz, 6H).

^{13}C NMR (126 MHz, $CDCl_3$): δ 170.8, 152.6, 150.1, 148.5, 147.6, 147.4, 142.1, 140.5, 140.1, 130.7, 130.0, 129.3, 127.1, 125.9, 112.4, 111.0, 110.1, 66.2, 52.8, 45.1, 44.4, 41.9, 39.9, 38.8, 33.1, 30.3, 27.1, 24.9, 22.5 (d, J = 3.1 Hz), 21.1, 18.1, 16.7.

HRMS (ESI) m/z ($[M+H]^+$) calcd for $C_{37}H_{49}O_2$: 525.3727. Found: 525.3722.

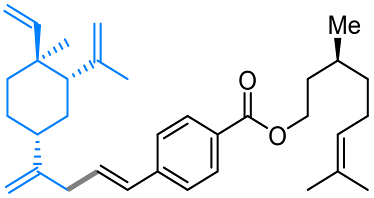

(*S*)-3,7-Dimethyloct-6-en-1-yl-4-((*E*)-4-((1*R*,3*S*,4*S*)-4-methyl-3-(prop-1-en-2-yl)-4-vinylcyclohexyl)penta-1,4-dien-1-yl)benzoate (59).

The title compound was prepared following the general procedure using (*S*)-3,7-dimethyloct-6-en-1-yl (*E*)-4-(2-bromovinyl)benzoate (54.8 mg, 0.150 mmol, 1.0 equiv), 2-((1*R*,3*S*,4*S*)-4-methyl-3-(prop-1-en-2-yl)-4-vinylcyclohexyl)allyl acetate (78.7 mg, 0.300 mmol, 2.0 equiv). After purification by column chromatography (using 5% ethyl acetate in petroleum ether), the title compound was isolated in 87% yield (63.7 mg) as a colorless oil.

^{1}H NMR (500 MHz, $CDCl_3$): δ 7.97 (d, J = 7.9 Hz, 2H), 7.41 (d, J = 8.0 Hz, 2H), 6.50–6.29 (m, 2H), 5.82 (dd, J = 17.4, 10.8 Hz, 1H), 5.10 (dd, J = 6.9, 5.9 Hz, 1H), 4.92 (s, 1H), 4.91 (s, 1H), 4.89 (d, J = 2.6 Hz, 1H), 4.82 (d, J = 5.1 Hz, 2H), 4.60 (s, 1H), 4.46–4.27 (m, 2H), 2.99 (d, J = 6.8 Hz, 2H), 2.07–1.97 (m, 4H), 1.92 (d, J = 10.1 Hz, 1H), 1.86–1.79 (m, 1H), 1.75 (d, J = 9.2 Hz, 1H), 1.71 (s, 3H), 1.68 (s, 3H), 1.61 (s, 3H), 1.56 (d, J = 8.1 Hz, 1H), 1.51–1.44 (m, 3H), 1.31 (dd, J = 17.7, 7.5 Hz, 4H), 1.01 (s, 3H), 0.97 (d, J = 6.5 Hz, 3H).

^{13}C NMR (126 MHz, $CDCl_3$): δ 166.6, 152.7, 150.3, 147.7, 142.1, 131.2, 131.5, 130.7, 130.0, 129.0, 126.0, 124.7, 112.3, 110.1, 109.3, 63.5, 52.9, 44.5, 40.0 (d, J = 10.8 Hz), 38.8, 37.1, 35.7, 35.1, 33.4, 29.7, 27.3, 25.8, 25.5, 24.3, 19.6, 17.8, 16.8.

HRMS (ESI) m/z ($[M+H]^+$) calcd for $C_{34}H_{49}O_2$: 489.3727. Found: 489.3730.

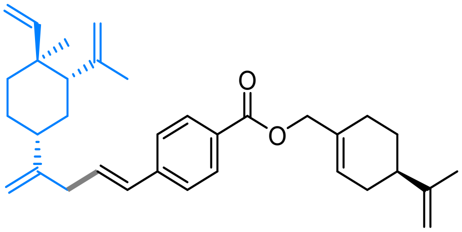

((*R*)-4-(Prop-1-en-2-yl)cyclohex-1-en-1-yl)methyl-4-

((*E*)-4-((1*R*,3*S*,4*S*)-4-methyl-3-(prop-1-en-2-yl)-4-vinylcyclohexyl)penta-1,4-dien-1-yl)benzoate (60).

The title compound was prepared following the general procedure using (*R*)-(4-(prop-1-en-2-yl)cyclohex-1-en-1-yl)methyl (*E*)-4-(2-bromovinyl)benzoate (54.2 mg, 0.150 mmol, 1.0 equiv), 2-((1*R*,3*S*,4*S*)-4-methyl-3-(prop-1-en-2-yl)-4-vinylcyclohexyl)allyl acetate (78.7 mg, 0.300 mmol, 2.0 equiv). After purification by column chromatography (using 5% ethyl acetate in petroleum ether), the title compound was isolated in 82% yield (59.5 mg) as a colorless oil.

^{1}H NMR (500 MHz, $CDCl_3$): δ 7.97 (d, J = 8.3 Hz, 2H), 7.40 (d, J = 8.3 Hz, 2H), 6.38 (dt, J = 15.8, 12.4 Hz, 2H), 5.82–5.80 (m, 2H), 5.00 (s, 1H), 4.91 (s, 1H), 4.88 (d, J = 2.1 Hz, 1H), 4.81 (s, 2H), 4.71 (dd, J = 8.9, 5.0 Hz, 4H), 4.57 (s, 1H), 2.98 (d, J = 6.7 Hz, 2H), 2.17 (d, J = 5.1 Hz, 3H), 2.08 (s, 3H), 2.01 (s, 2H), 1.90 (d, J = 10.0 Hz, 3H), 1.70–1.69 (m, 3H), 1.59 (s, 2H), 1.46 (d, J = 3.4 Hz, 4H), 1.29 (s, 1H), 1.00–0.99 (m, 3H).

^{13}C NMR (126 MHz, $CDCl_3$): δ 170.8, 154.9, 152.6, 150.1, 148.5, 147.4, 142.2, 132.9, 131.7, 130.7, 130.0, 126.0, 125.6, 112.4, 111.0, 110.1, 108.9, 68.8, 66.2, 55.8, 52.8, 48.5, 45.7, 44.4, 41.9, 40.9, 39.9, 38.8, 36.5, 35.0, 33.1, 30.6.

HRMS (ESI) m/z ($[M+H]^+$) calcd for $C_{34}H_{45}O_2$: 485.3414. Found: 485.3409.

V. In Vitro Anti-Tumour Activity of Compound 55

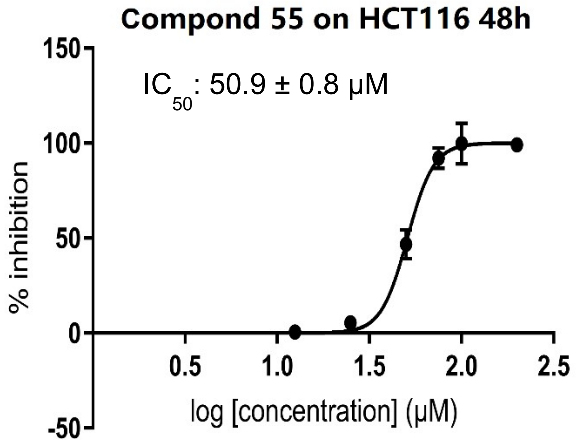

Figure S1. IC_{50} of Compond **55** on HCT116

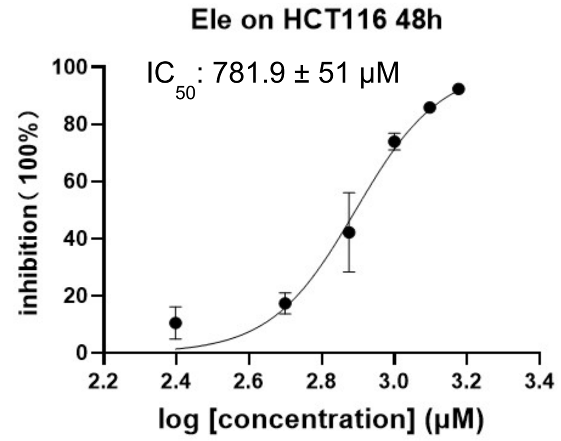

Figure S2. IC_{50} of *β*-elemene on HCT116

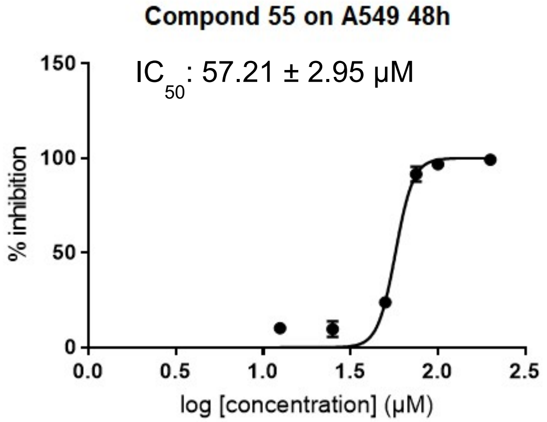

Figure S3. IC_{50} of Compond **55** on A549

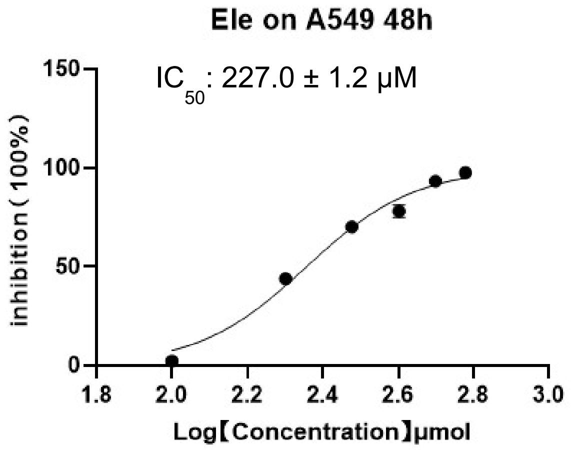

Figure S4. IC_{50} of *β*-elemene on A549

VI. Preparation of Alkenyl Bromides

A general procedure for the preparation of vinyl bromides. [14-16]

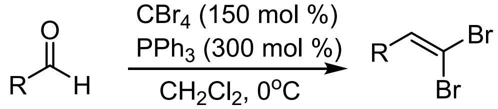

Step 1: To a flame-dried flask was added aldehyde (20 mmol, 100 mol%), CBr_4 (30 mmol, 150 mol%), and CH_2Cl_2 (80 mL). The flask was cooled to 0 °C, at which point a solution of PPh_3 (60 mmol, 300 mol%) in CH_2Cl_2 (70 mL) was added dropwise via addition funnel over 30 min. The solution was stirred at 0 °C under N_2 for 1 h. About half of the volume of CH_2Cl_2 was removed under reduced pressure. Pentane (100 mL) was added, and triphenylphosphine oxide (TPPO) precipitated out. After filtration and evaporation of the solvent, the residue was dissolved in pentane (50 mL) which led to further precipitation of TPPO. Filtration and evaporation of the solvent afforded the crud dibromide which was directly used for the next step.

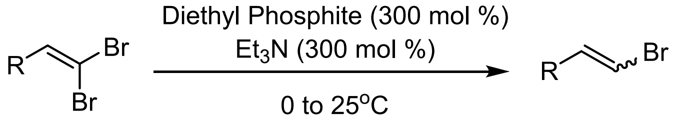

Step 2: To a solution of the crude dibromide (~ 20.0 mmol, 100 mol%) and NEt_3 (60 mmol, 300 mol%) in DMF (20 mL) was added dimethyl phosphonate (60.0 mmol, 300 mol%). The solution was stirred over night at room temperature. Water (60 mL) was added to the mixture, which was extracted with pentane (2 × 100 mL). The combined organic phases were washed with an aqueous solution of HCl (1 M, 55 mL) and dried over Na_2SO_4, filtered, and concentrated. The crude material was purified by flash chromatography.

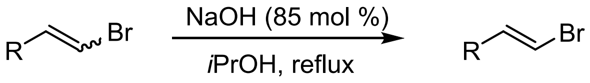

Step 3: The crude product (~20.0 mmol, 100 mol%) from the previous step was dissolved in *i*PrOH

(30 mL). Solid NaOH (17.0 mmol, 85 mol%) was added and the mixture was heated to reflux for 1.5 hours. The reaction mixture was cooled to room temperature, diluted with pentane (100 mL), and partitioned with distillated H_2O (2 × 100 mL). The organic phase was collected, and washed with an aqueous solution of HCl (1 M, 75 mL), dried over Na_2SO_4. The solvent was removed under reduced pressure. The crude material was purified by flash chromatography.

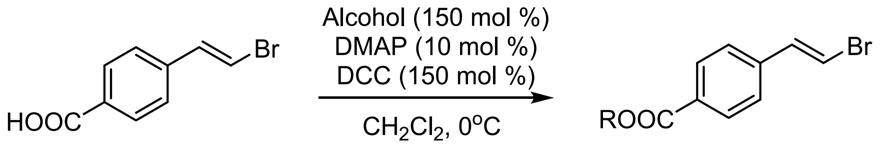

Step 4: To a flame-dried flask was added acid (10 mmol, 100 mol%), DMAP (1 mmol, 10 mol%), DCC (15 mmol, 150 mol%), and CH_2Cl_2 (30 mL). The flask was cooled to 0 °C, at which point a solution of alcohol (15 mmol, 150 mol%) in CH_2Cl_2 (30 mL) was added dropwise via addition funnel over 30 min. The solution was stirred at 0 °C under N_2 over night. After the reaction was finshed the CH_2Cl_2 was removed under reduced pressure. And The crude residue was purified by silicagel chromatography (hexanes) to give the target compound.

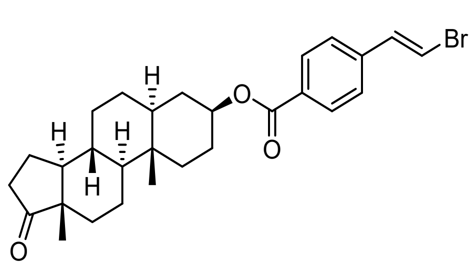

(3*S*,5*S*,8*R*,9*S*,10*S*,13*S*,14*S*)-10,13-Dimethyl-17-oxohexadecahydro-1*H*-cyclopenta[*a*]phenanthren-3-yl 4-((*E*)-2-bromovinyl)benzoate.

The title compound was prepared following the general procedure using (*E*)-4-(2-bromovinyl)benzoic acid (2.3 g, 10 mmol, 1.0 equiv), epiandrosterone (4.4 g, 15 mmol, 1.5 equiv). After purification by column chromatography (using 5% ethyl acetate in petroleum ether), the title compound was isolated in 80% yield (4.0 g) as a white solid.

^{1}H NMR (500 MHz, $CDCl_3$): δ 8.02–7.92 (m, 1H), 7.33 (t, J = 5.8 Hz, 1H), 7.18–7.08 (m, 1H), 6.97–6.83 (m, 1H), 5.01–4.83 (m, 1H), 2.48–2.38 (m, 1H), 2.14–2.01 (m, 1H), 1.93 (ddd, J = 15.1, 10.4, 6.2 Hz, 1H), 1.82–1.72 (m, 1H), 1.69–1.62 (m, 1H), 1.58–1.46 (m, 1H), 1.36–1.24 (m, 1H), 1.10 (dq, J = 13.9, 3.9

Hz, 1H), 0.99 (ddd, J = 19.4, 9.7, 4.9 Hz, 1H), 0.91–0.84 (m, 1H), 0.72 (d, J = 4.1 Hz, 1H).

^{13}C NMR (126 MHz, $CDCl_3$): δ 165.7, 140.0, 136.5, 132.1, 130.2, 129.5, 126.0, 109.4, 74.4, 54.4, 51.5, 47.9, 44.8, 36.9, 36.0, 35.8, 35.2, 34.2, 31.7, 30.9, 28.4, 27.6, 21.9, 20.6, 13.9, 12.4.

HRMS (ESI) m/z ([M+H]$^+$) calcd for $C_{28}H_{36}BrO_3$: 499.1842. Found: 499.1843.

M.p.: 240-241 ºC.

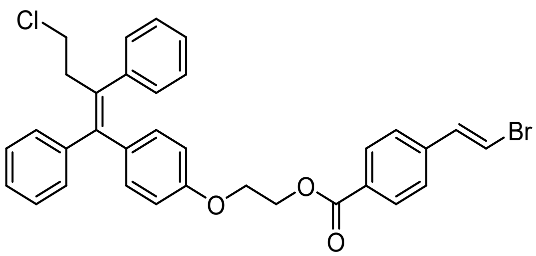

2-(4-((*Z*)-4-Chloro-1,2-diphenylbut-1-en-1-yl)phenoxy)ethyl 4-((*E*)-2-bromovinyl)benzoate. The title compound was prepared following the general procedure using (*E*)-4-(2-bromovinyl)benzoic acid (2.3 g, 10 mmol, 1.0 equiv), ospemifene (5.7 g, 15 mmol, 1.5 equiv). After purification by column chromatography (using 5% ethyl acetate in petroleum ether), the title compound was isolated in 79% yield (4.6 g) as a white solid.

^{1}H NMR (500 MHz, $CDCl_3$): δ 7.98 (d, J = 8.3 Hz, 2H), 7.40–7.35 (m, 2H), 7.33 (d, J = 8.3 Hz, 2H), 7.30 (t, J = 6.7 Hz, 3H), 7.23–7.18 (m, 2H), 7.14 (dd, J = 13.3, 6.1 Hz, 4H), 6.92 (d, J = 14.0 Hz, 1H), 6.81 (d, J = 8.8 Hz, 2H), 6.60 (d, J = 8.7 Hz, 2H), 4.64–4.51 (m, 2H), 4.23–4.13 (m, 2H), 3.42 (t, J = 7.4 Hz, 2H), 2.93 (t, J = 7.4 Hz, 2H).

^{13}C NMR (126 MHz, $CDCl_3$): δ 166.2, 156.9, 143.0, 141.8, 141.1, 140.4, 136.5, 135.52, 135.49, 132.2, 131.9, 130.4, 129.7, 129.5, 128.5, 128.4, 127.1, 126.8, 126.1, 113.7, 109.7, 65.8, 63.6, 43.0, 38.7.

HRMS (ESI) m/z ([M+K]$^+$) calcd for $C_{33}H_{28}BrClKO_3$: 625.0542. Found: 625.0544.

M.p.: 165-166 ºC.

VII. Preparation of β-Elemene Derived-Acetate 1c:

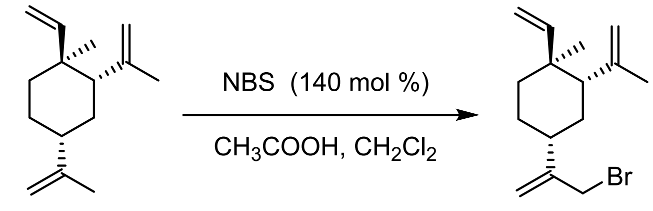

β-elemene **1c'**

Step 1: To a flame-dried flask was added β-elemene (10.0 mmol, 100 mol%), NBS (14.0 mmol, 140 mol%), CH_3COOH (30 mL), and CH_2Cl_2 (10 mL). The solution was stirred at 25 °C under air for 3 h. After the reaction was completed, water (60 mL) was then added to the mixture and extracted with pentane (2 × 100 mL). The combined organic phases were collected and dried over Na_2SO_4, filtered, and concentrated. After purification by column chromatography (only using petroleum ether), the title compound **1c'** was isolated in 28% yield (0.8 g) as a colorless oil.

^{1}H NMR (400 MHz, $CDCl_3$): δ 5.82 (dd, J = 17.8, 10.5 Hz, 1H), 5.20 (s, 1H), 5.04 (s, 1H), 4.93 (dd, J = 5.2, 1.3 Hz, 1H), 4.90 (d, J = 1.4 Hz, 1H), 4.87–4.82 (m, 1H), 4.59 (s, 1H), 4.03 (s, 2H), 2.26 (ddd, J = 15.0, 8.1, 3.8 Hz, 1H), 2.05 (dd, J = 12.6, 3.4 Hz, 1H), 1.72 (s, 3H), 1.68–1.57 (m, 2H), 1.56–1.40 (m, 4H), 1.01 (s, 3H).

^{13}C NMR (101 MHz, $CDCl_3$): δ 149.6, 149.0, 147.5, 116.4, 113.5, 111.6, 51.1, 47.8, 47.5, 41.0, 39.8, 39.7, 33.8, 27.0, 15.8.

HRMS (ESI) m/z ([M+Na]$^+$) calcd for $C_{15}H_{23}BrNa$: 305.0875. Found: 305.0876.

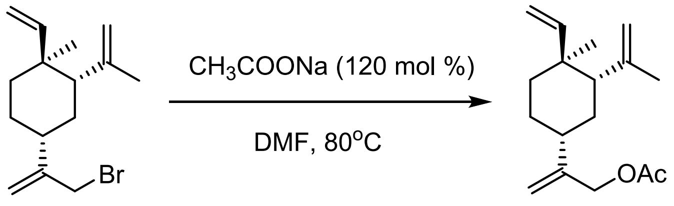

1c' **1c**

Step 2: To a flame-dried flask was added the compound **1c'** (10.0 mmol, 100 mol%) and CH_3COONa (12.0 mmol, 120 mol%) in DMF (20 mL), which was stirred at 80 ^{o}C over night. Then water (60 mL) was added to the mixture, which was extracted with pentane (2 × 100 mL). The combined organic phases were collected and dried over Na_2SO_4, filtered, and concentrated. After purification by column

chromatography (only using petroleum ether), the title compound **1c** was isolated in 67% yield (1.7 g) as a colorless oil.

^{1}H NMR (500 MHz, $CDCl_3$): δ 5.86–5.77 (m, 1H), 5.08–4.98 (m, 2H), 4.92 (dd, J = 6.2, 1.3 Hz, 1H), 4.89 (s, 1H), 4.85–4.80 (m, 1H), 4.58 (s, 3H), 2.09 (s, 3H), 2.08–1.99 (m, 2H), 1.70 (d, J = 0.4 Hz, 3H), 1.65 (ddd, J = 9.3, 8.4, 5.2 Hz, 1H), 1.60–1.33 (m, 5H), 1.00 (s, 3H).

^{13}C NMR (126 MHz, $CDCl_3$): δ 170.9, 150.2, 148.6, 147.5, 112.4, 111.1, 110.2, 66.3, 52.8, 42.0, 39.95, 39.89, 33.2, 27.2, 24.9, 21.2, 16.7.

HRMS (ESI) m/z ($[M+Na]^+$) calcd for $C_{17}H_{26}NaO_2$: 285.1825. Found: 285.1850.

VIII. Mechanistic Investigations

1. Organozinc Experiment

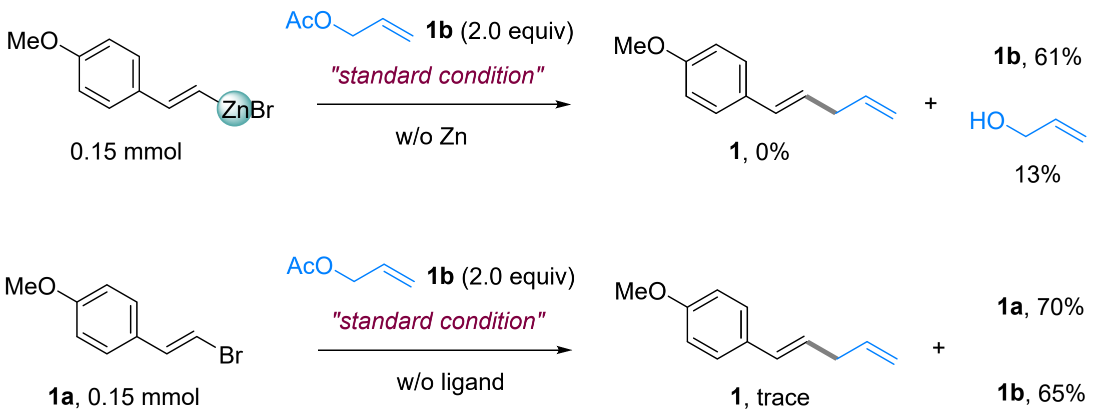

Figure S5. Organozinc experiment. (Yields determined by GC-MS)

2. Control Experiments

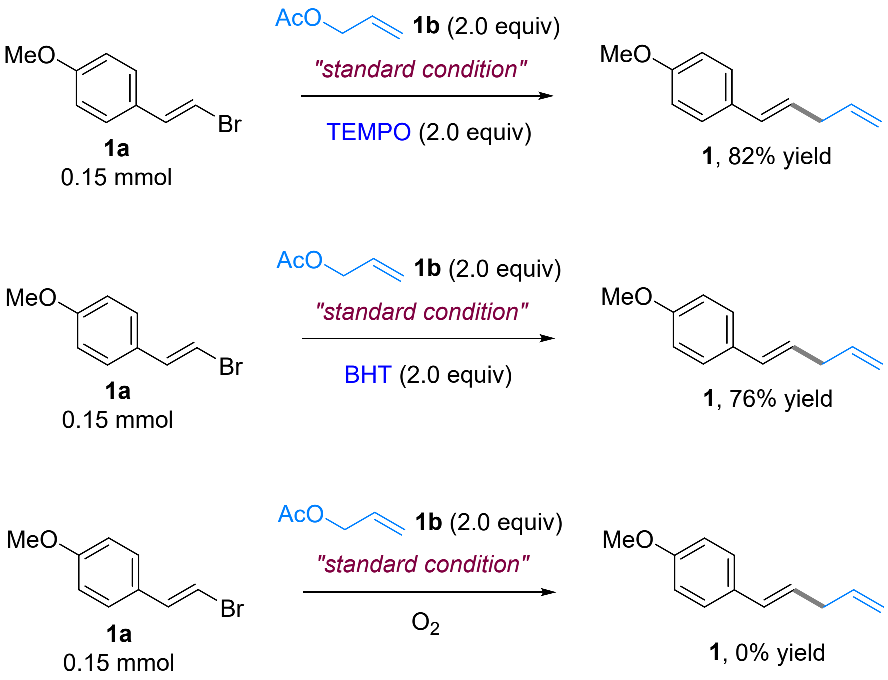

Figure S6. Control experiments.

IX. References

[1] Ye, Y.; Liu, J.; Xu, B.; Jiang, S.; Bai, R.; Li, S.; Xie, T.; Ye, X.-Y. *Chem. Sci.* **2021**, *12*, 13209.

[2] Yang, B.; Wang, Z.-X. *J. Org. Chem.* **2017**, *82*, 4542.

[3] Gumrukcu, Y.; de Bruin, B.; Reek, J. N. H. *Chem. Eur. J.* **2014**, *20*, 10905.

[4] Frye, E. C.; O'Connor, C. J.; Twigg, D. G.; Elbert, B.; Laraia, L.; Hulcoop, D. G.; Venkitaraman, A. R.; Spring, D. R. *Chem. Eur. J.* **2012**, *18*, 8774.

[5] Li, Y.; Luo, Y.; Peng, L.; Li, Y.; Zhao, B.; Wang, W.; Pang, H.; Deng, Y.; Bai, R.; Lan, Y.; Yin, G. *Nat. Commun.* **2020**, *11*, 417.

[6] Kabalka, G. W.; Dadusha, E.; Al-Masum, M. *Tetrahedron Lett.* **2006**, *47*, 7459.

[7] Mailig, M.; Hazra, A.; Armstrong, M. K.; Lalic, G. *J. Am. Chem. Soc.* **2017**, *139*, 6969.

[8] Dey, R.; Chattopadhyay, K.; Ranu, B. C. *J. Org. Chem.* **2008**, *73*, 9461.

[9] Nguyen, B. H.; Perkins, R. J.; Smith, J. A.; Moeller, K. D. *J. Org. Chem*. **2015**, *80*, 11953.

[10] Takeda, T.; Matsumura, R.; Wasa, H.; Tsubouchi, A. *Asian J. Org. Chem.* **2014**, *3*, 838.

[11] Rimkus, A. M.; Alt, H. G. *Polyhedron* **2017**, *126*, 72.

[12] Matsubara, R.; Jamison, T. F. *Chem. Asian J.* **2011**, *6*, 1860.

[13] Song, F.; Wang, F.; Guo, L.; Feng, X.; Zhang, Y.; Chu, L. *Angew. Chem. Int. Ed.* **2020**, *59*, 177.

[14] Ramirez, F.; Desai, N. B.; Kelvie, N. *J. Am. Chem. Soc.* **1962**, *84*, 1745.

[15] Abbas, S.; Hayes, C. J.; Worden, S. *Tetrahedron Lett.* **2000**, *41*, 3215.

[16] Dolby, L. J.; Wilkins, D. C.; Frey, T. G. *J. Org. Chem.* **1966**, *31*, 1110.

X. Spectral Data (NMR Spectrum and HPLC Trace)

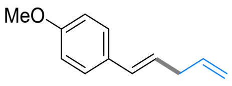

1

^{1}H NMR (500 MHz, $CDCl_3$)

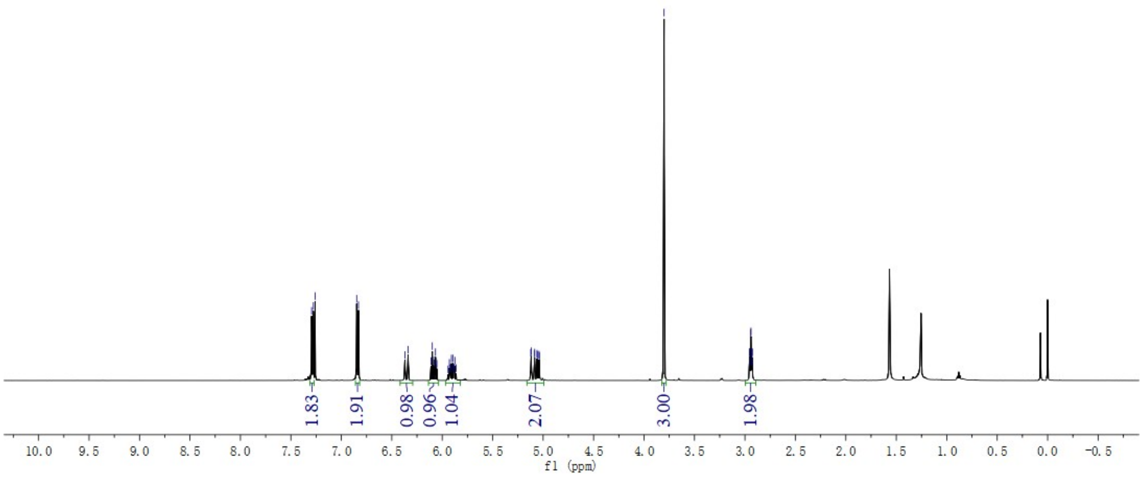

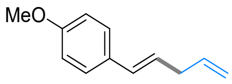

1

^{13}C NMR (126 MHz, $CDCl_3$)

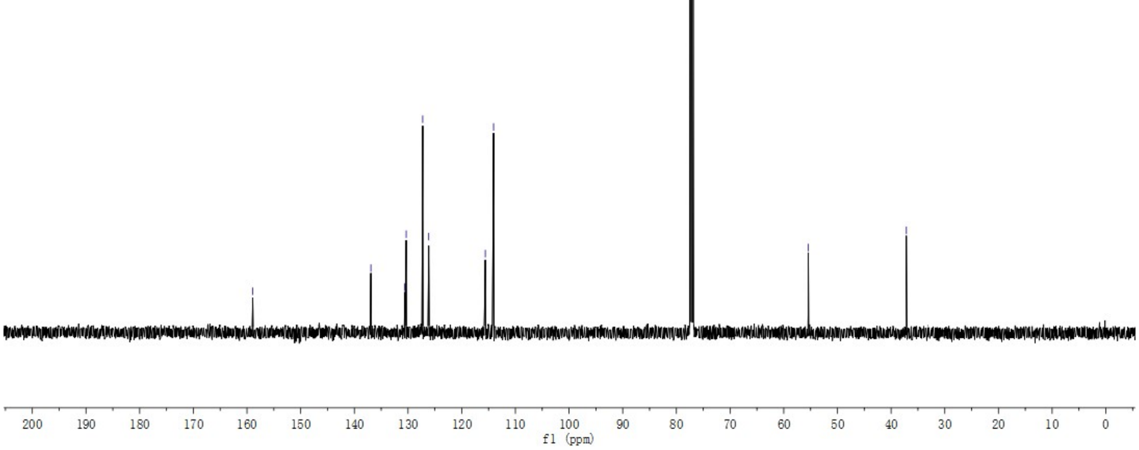

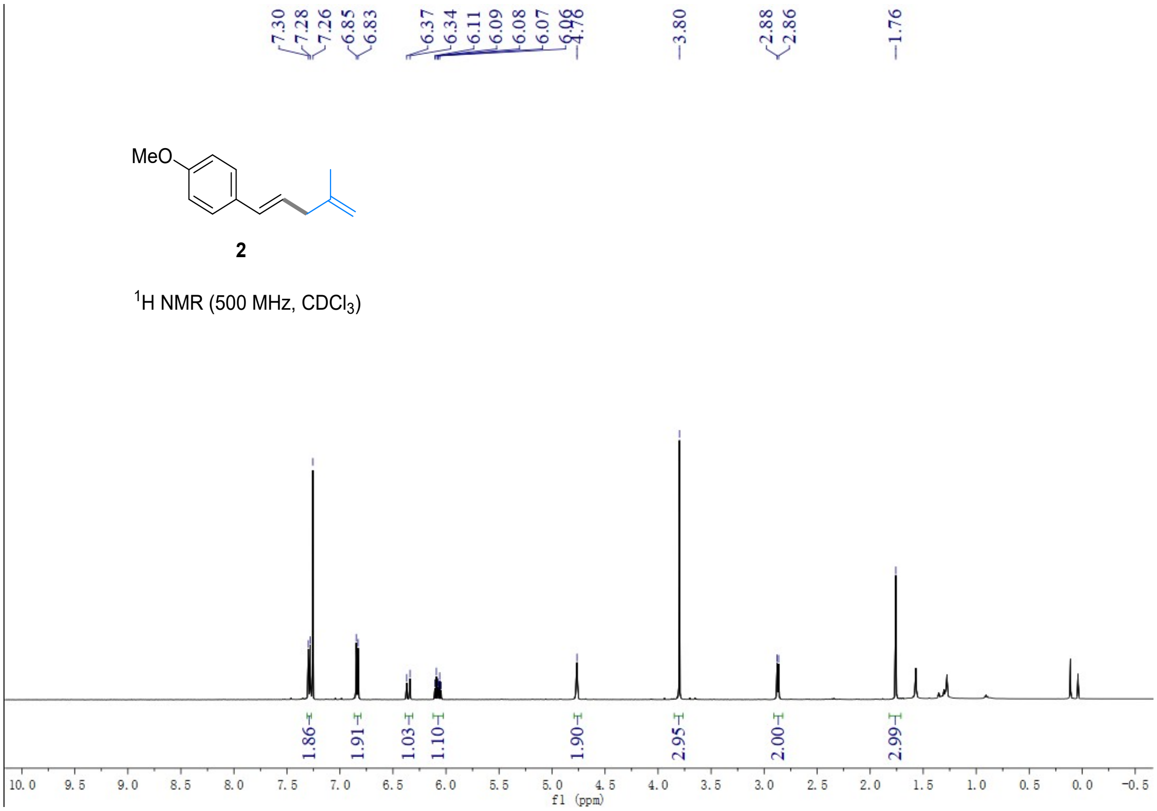

7.30
7.28
7.26
6.85
6.83
6.37
6.34
6.11
6.09
6.08
6.07
6.06
4.76
3.80
2.88
2.86
1.76
MeO
2
1H NMR (500 MHz, CDCl3)
1.86
1.91
1.03
1.10
1.90
2.95
2.00
2.99
f1 (ppm)

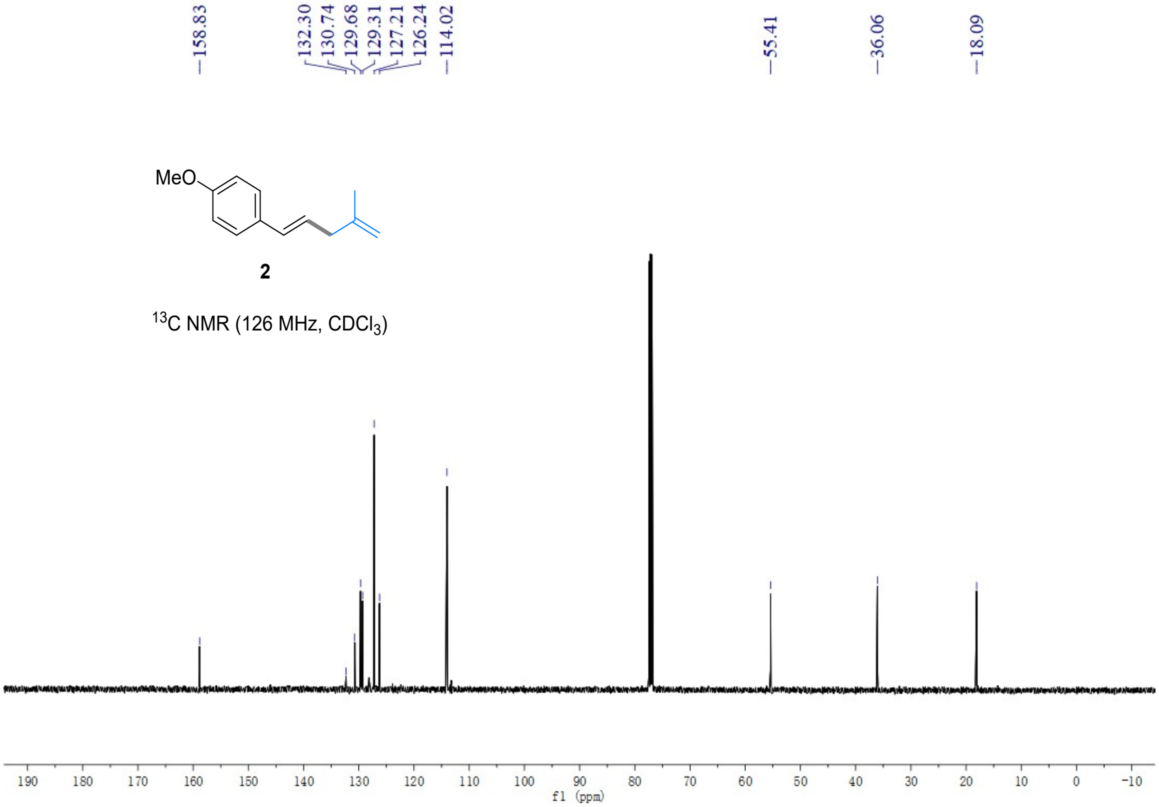

158.83
132.30
130.74
129.68
129.31
127.21
126.24
114.02
55.41
36.06
18.09
MeO
2
13C NMR (126 MHz, CDCl3)
f1 (ppm)

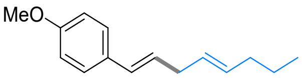

3

^{1}H NMR (500 MHz, $CDCl_3$)

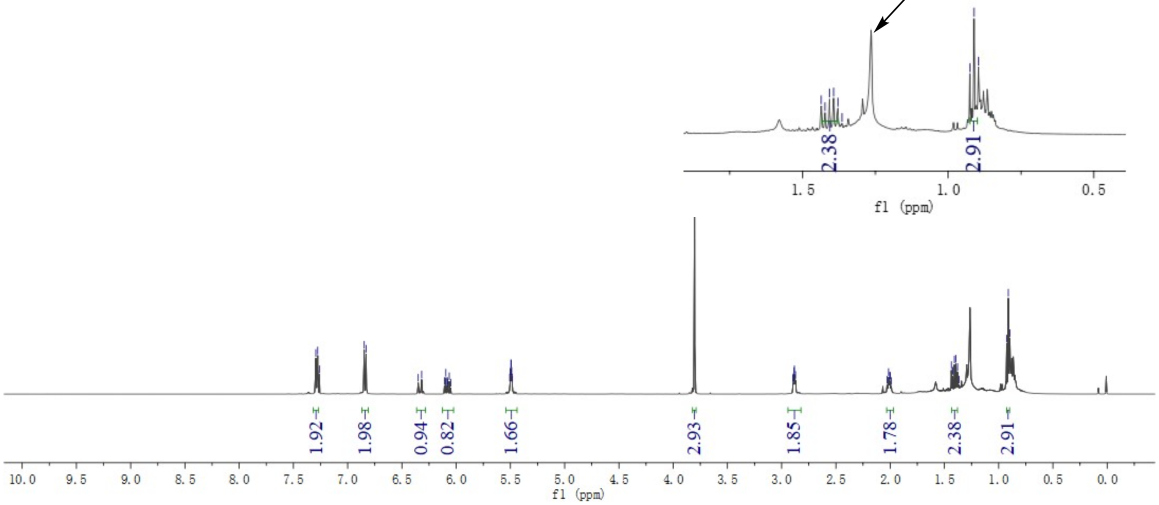

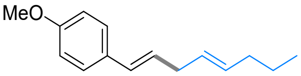

3

^{13}C NMR (126 MHz, $CDCl_3$)

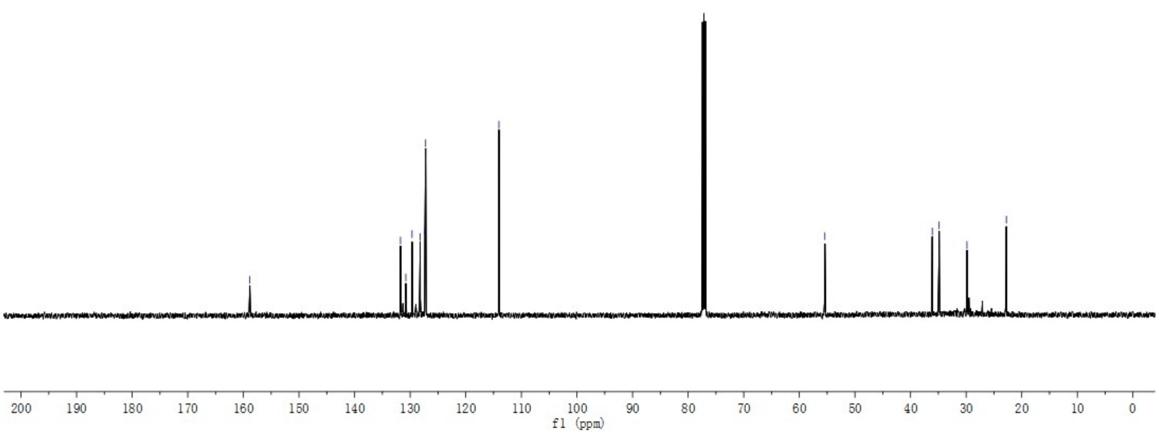

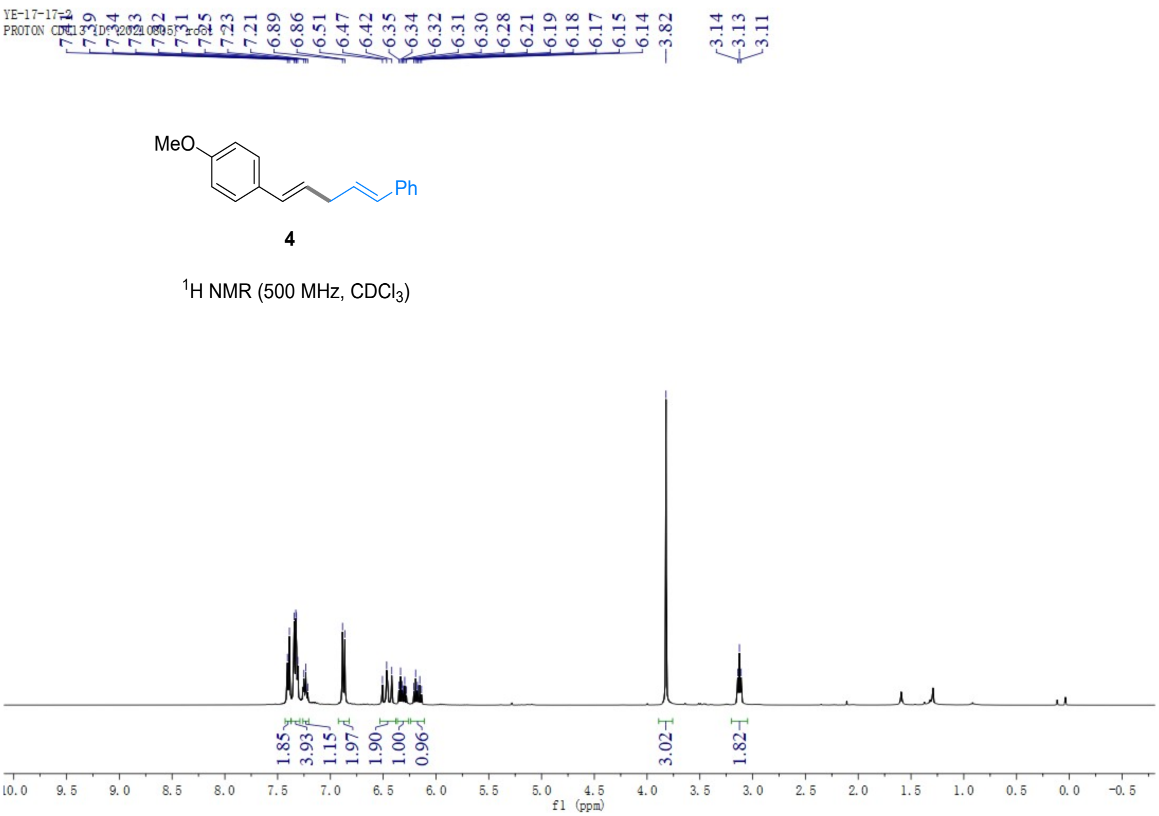
YE-17-17-2
PROTON CDCl3 1D
MeO
Ph
4
1H NMR (500 MHz, CDCl3)
3.82
3.14
3.13
3.11
3.02
1.82

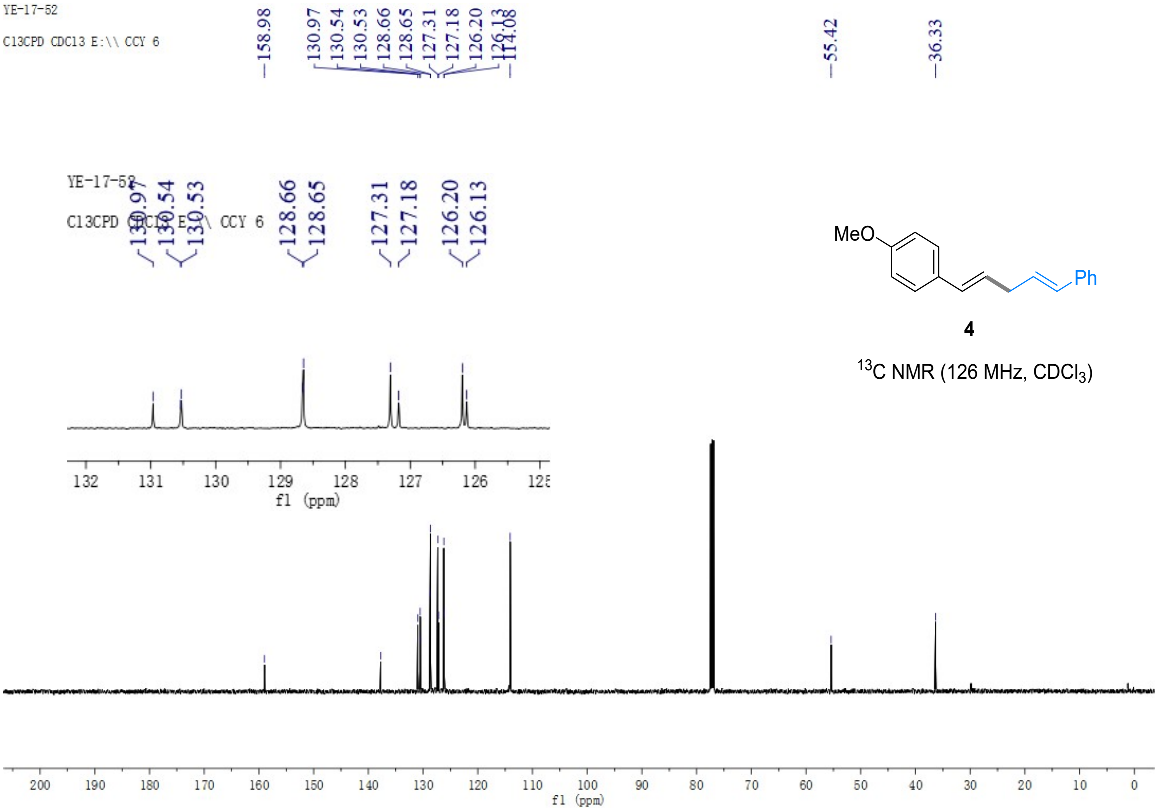
YE-17-52
C13CPD CDCl3 E:\\ CCY 6
158.98
130.97
130.54
130.53
128.66
128.65
127.31
127.18
126.20
126.13
114.08
55.42
36.33
MeO
Ph
4
13C NMR (126 MHz, CDCl3)

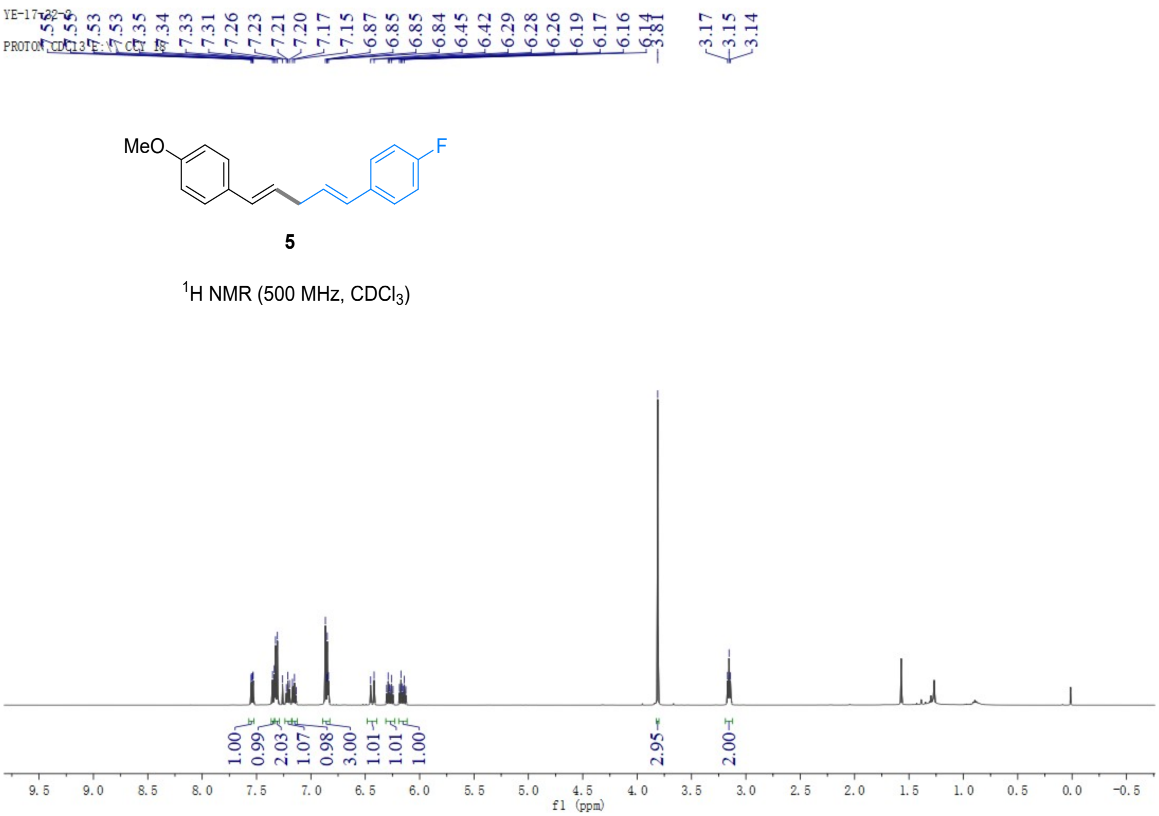

5

^{1}H NMR (500 MHz, $CDCl_3$)

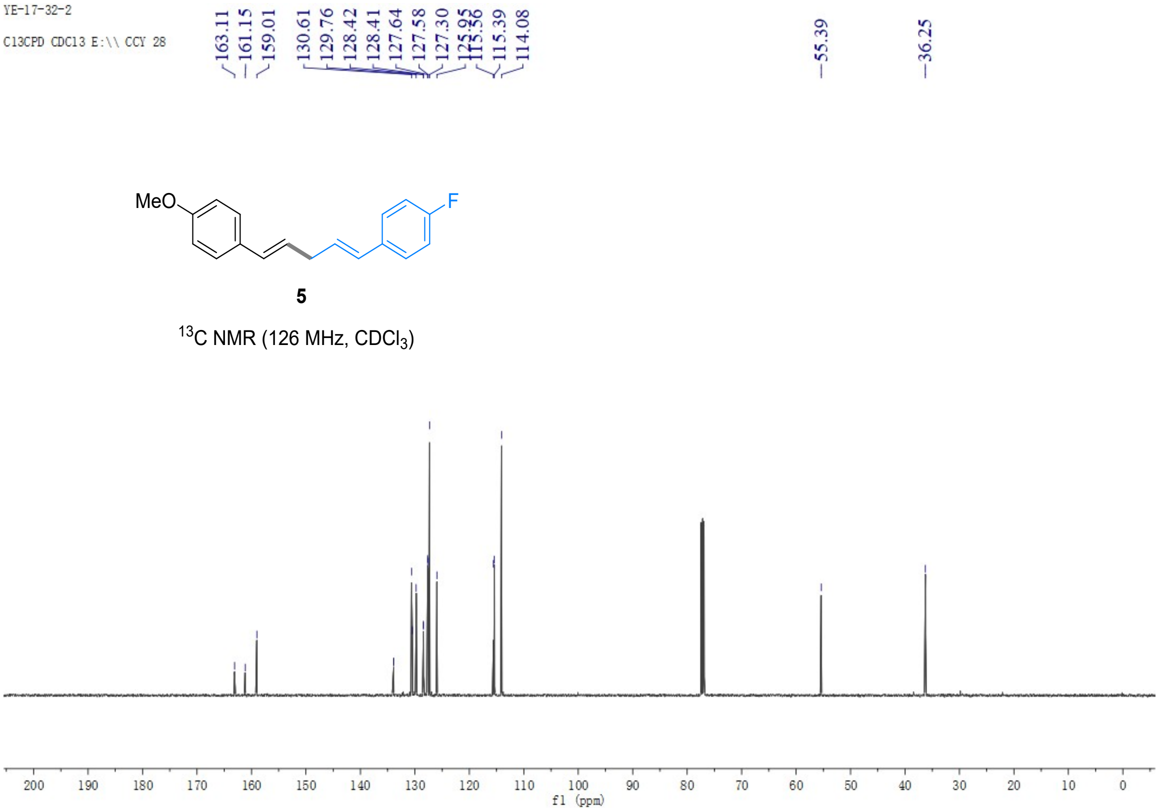

5

^{13}C NMR (126 MHz, $CDCl_3$)

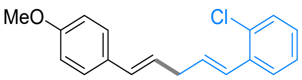

6

^{1}H NMR (500 MHz, CDCl3)

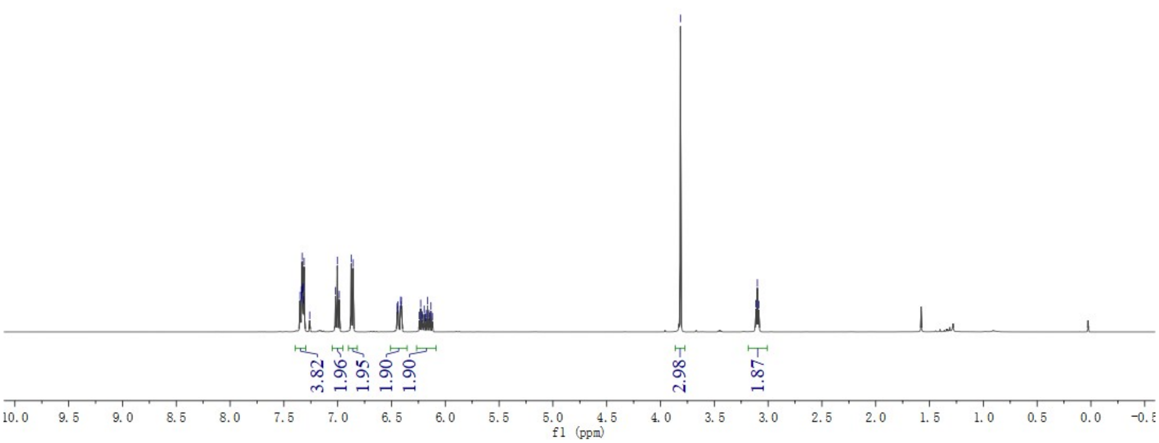

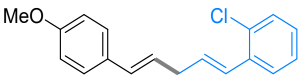

6

^{13}C NMR (126 MHz, CDCl3)

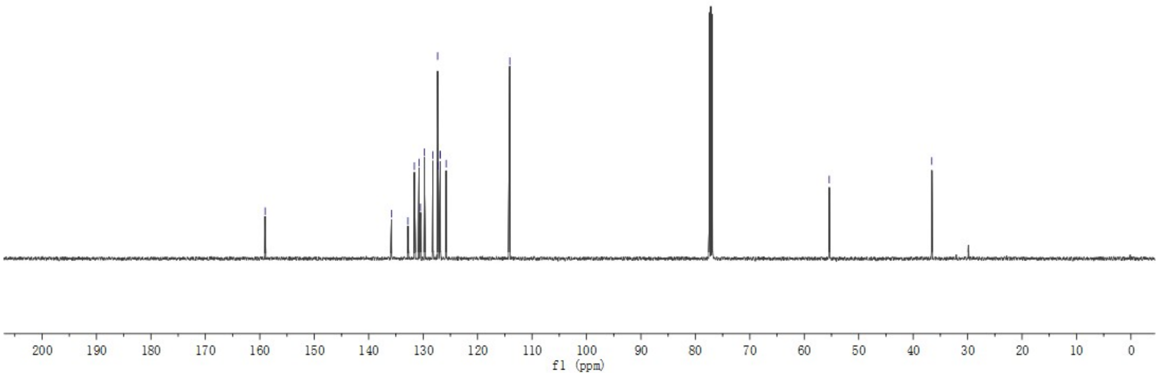

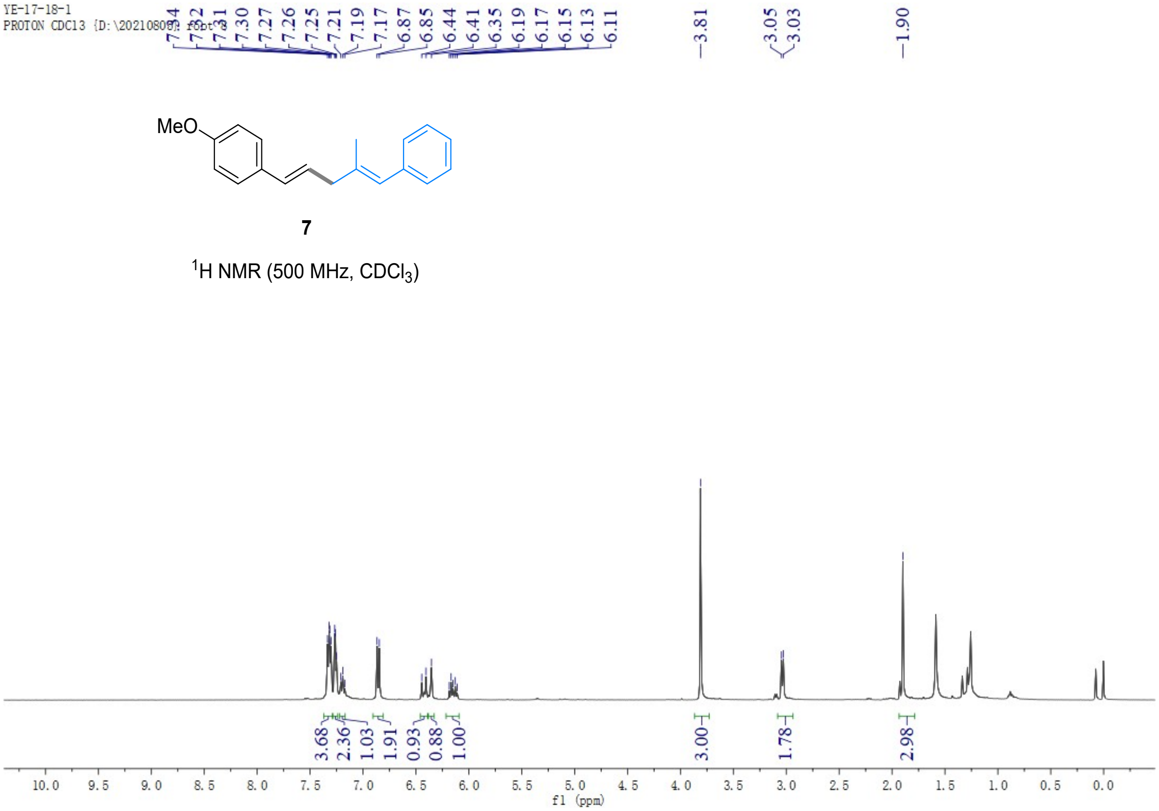

7

^{1}H NMR (500 MHz, $CDCl_3$)

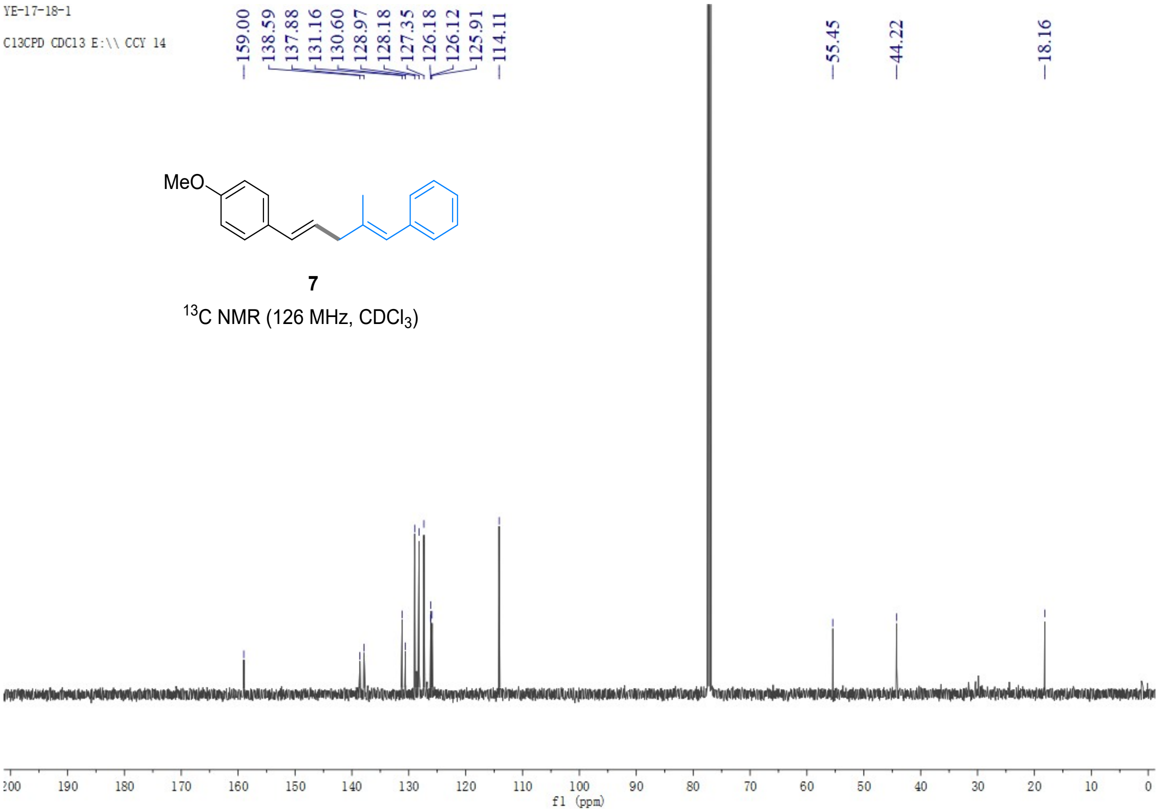

7

^{13}C NMR (126 MHz, $CDCl_3$)

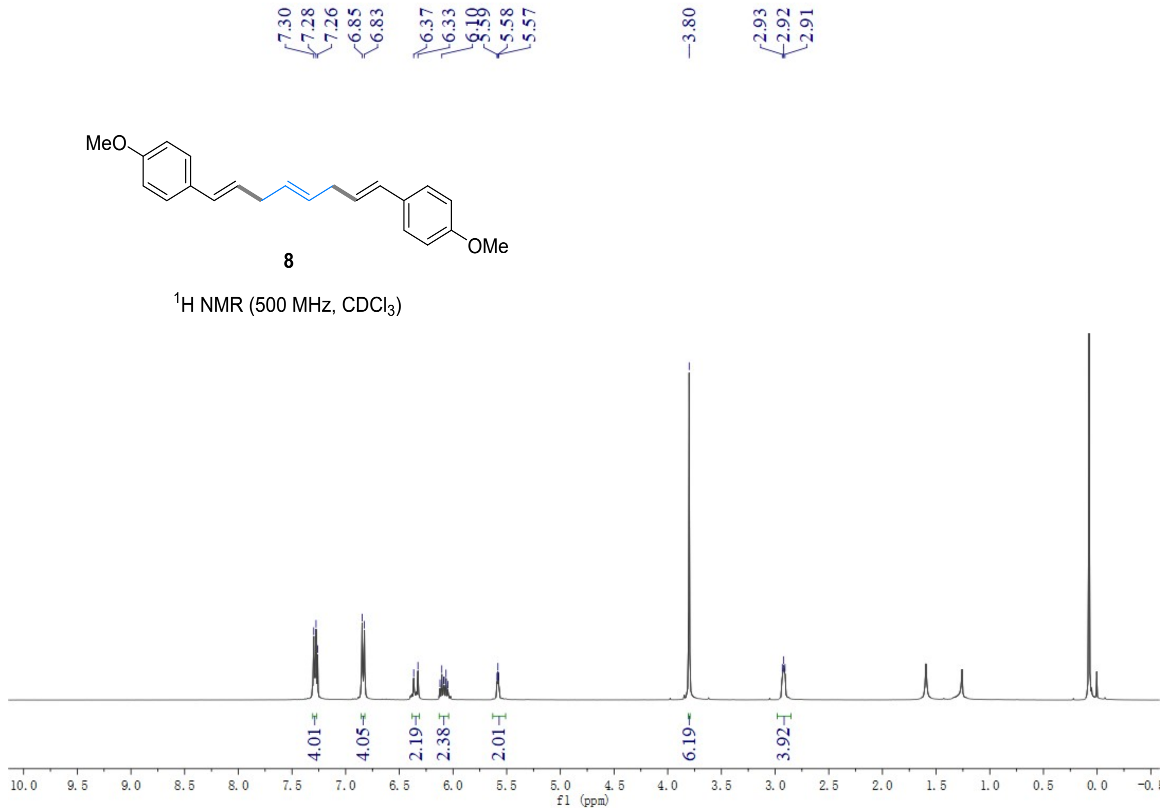
7.30
7.28
7.26
6.85
6.83
6.37
6.33
6.10
5.59
5.58
5.57
3.80
2.93
2.92
2.91
MeO
OMe
8
^{1}H NMR (500 MHz, CDCl$_3$)
4.01
4.05
2.19
2.38
2.01
6.19
3.92
10.0
9.5
9.0
8.5
8.0
7.5
7.0
6.5
6.0
5.5
5.0
4.5
4.0
3.5
3.0
2.5
2.0
1.5
1.0
0.5
0.0
f1 (ppm)

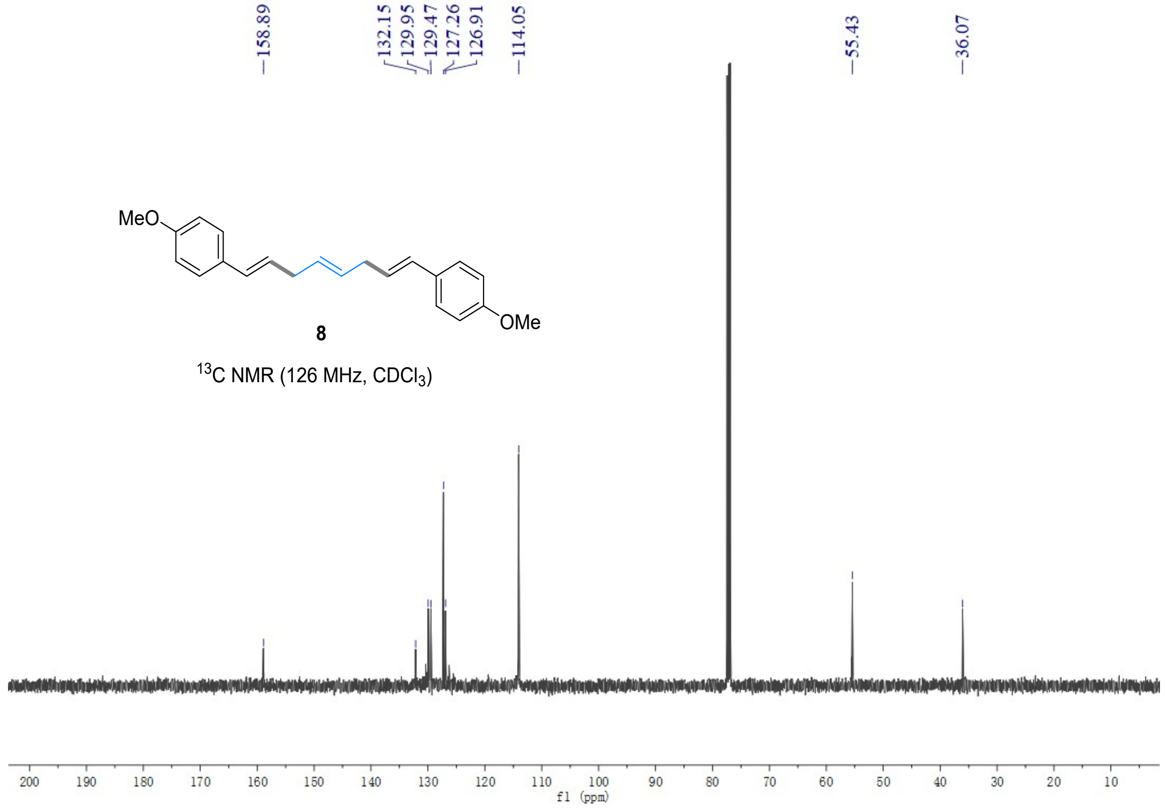
158.89
132.15
129.95
129.47
127.26
126.91
114.05
55.43
36.07
MeO
OMe
8
^{13}C NMR (126 MHz, CDCl$_3$)
200
190
180
170
160
150
140
130
120
110
100
90
80
70
60
50
40
30
20
10
f1 (ppm)

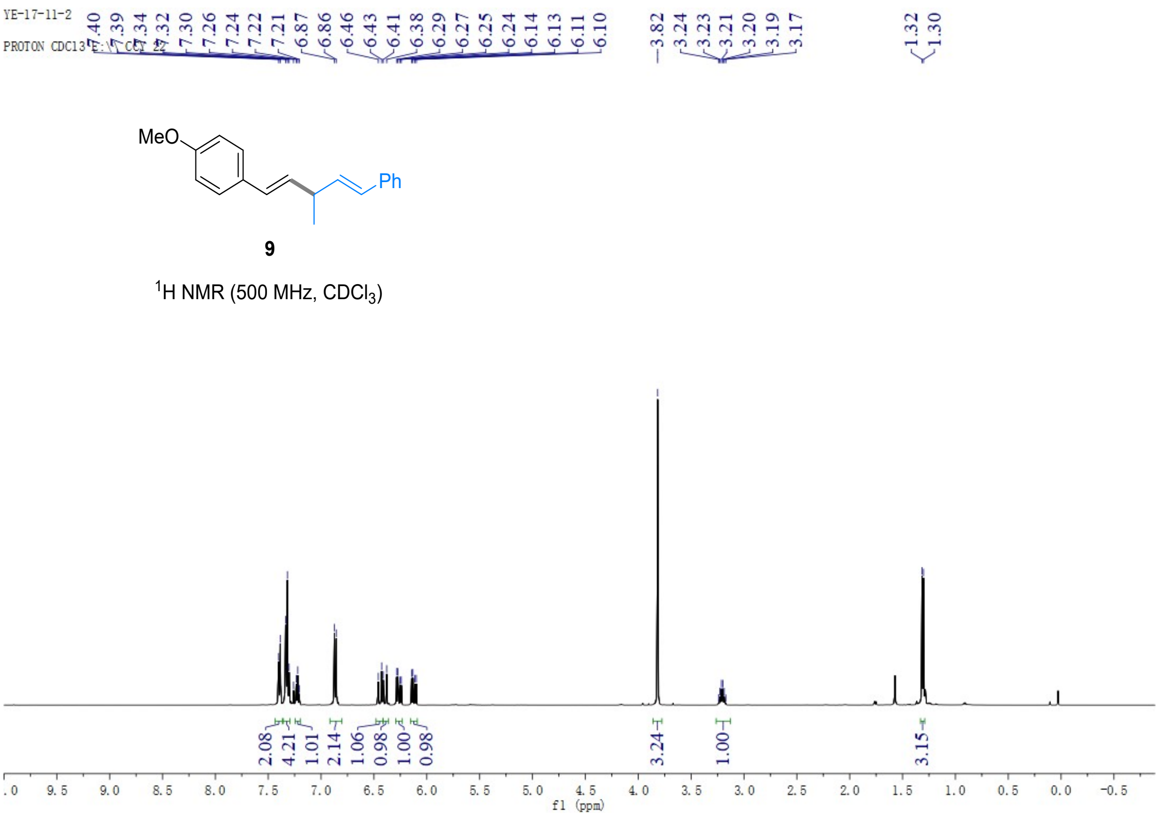

9

^{1}H NMR (500 MHz, $CDCl_3$)

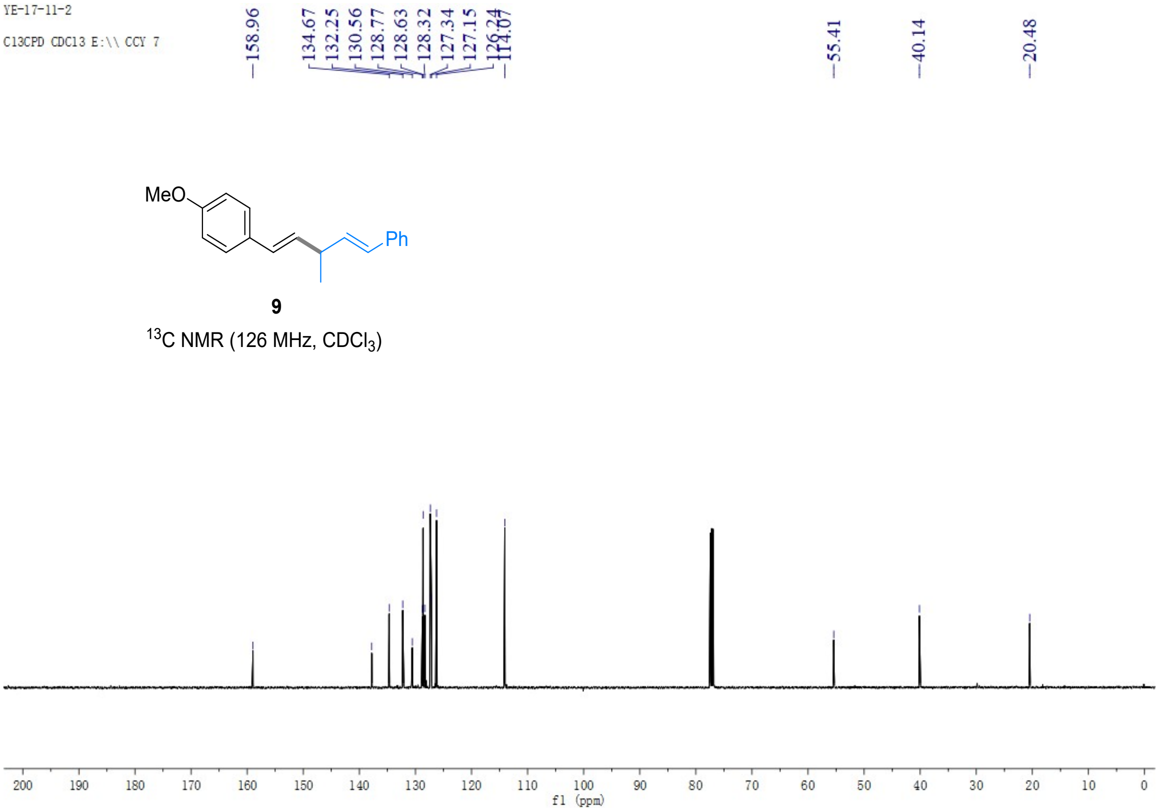

9

^{13}C NMR (126 MHz, $CDCl_3$)

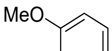

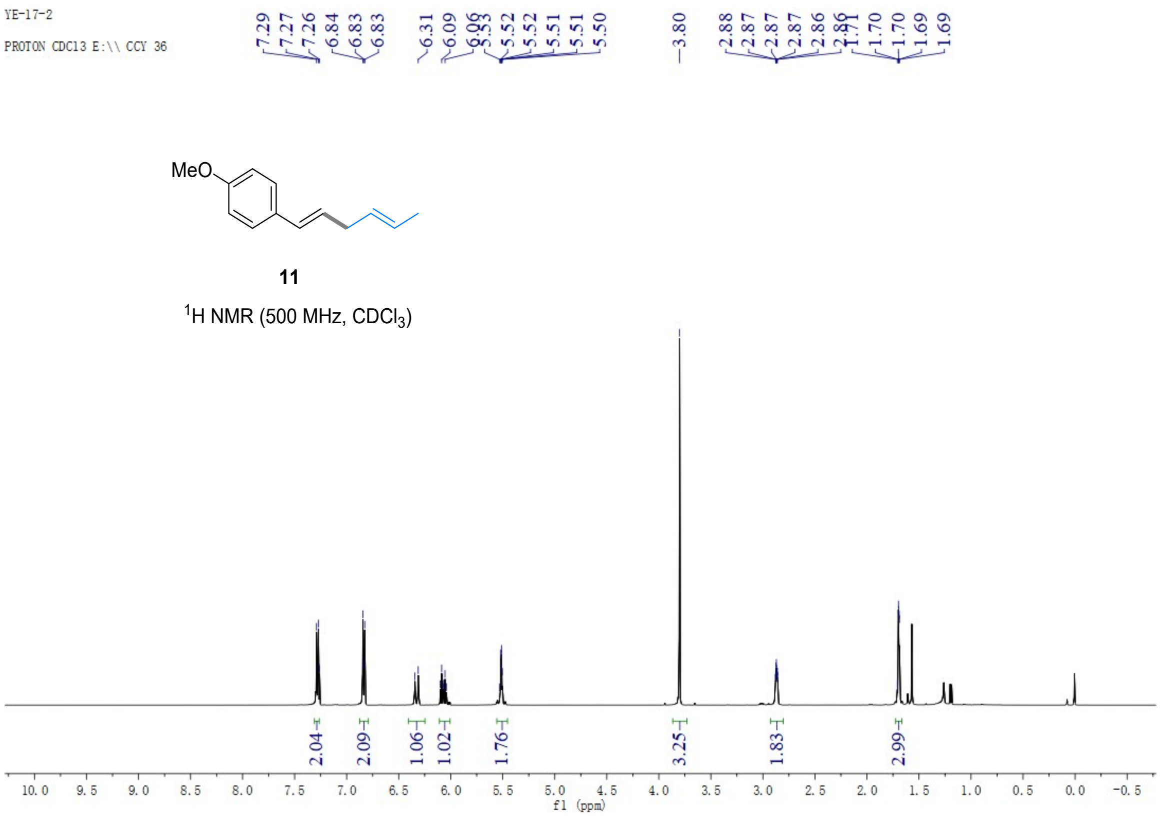

11

^{1}H NMR (500 MHz, $CDCl_3$)

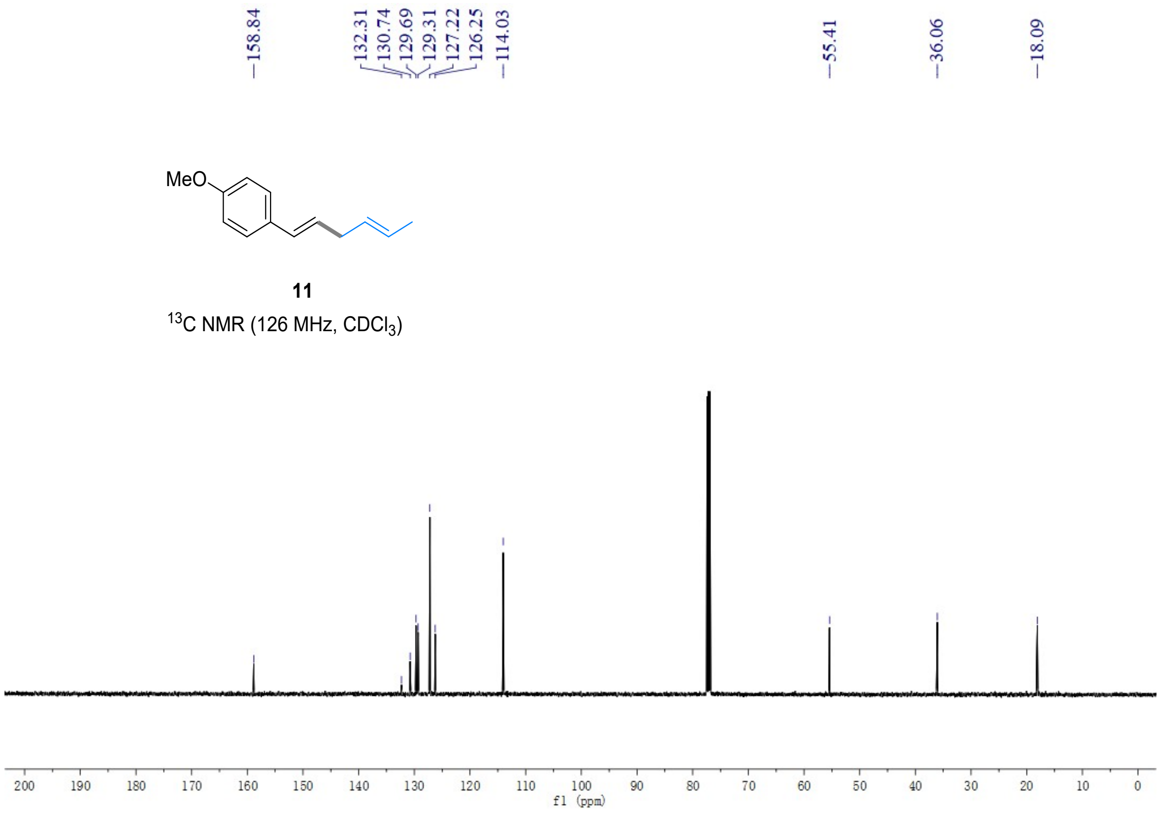

11

^{13}C NMR (126 MHz, $CDCl_3$)

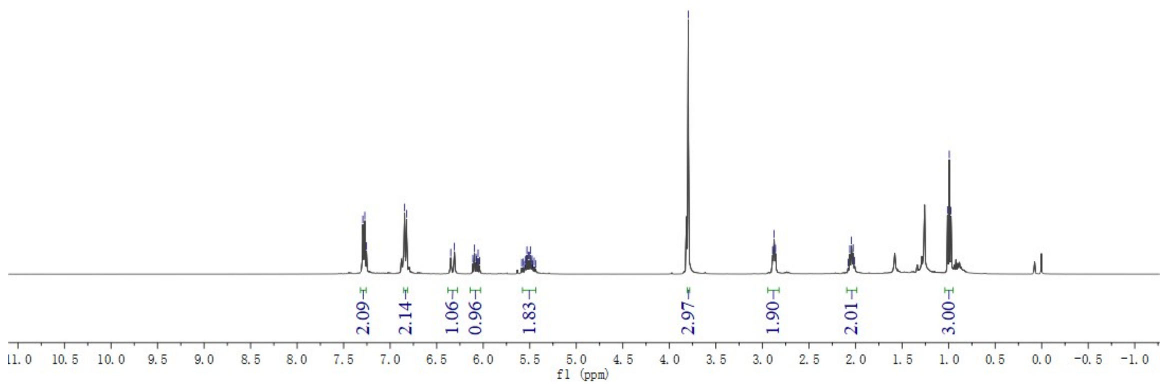

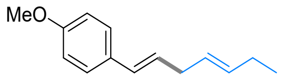

12

^{1}H NMR (500 MHz, $CDCl_3$)

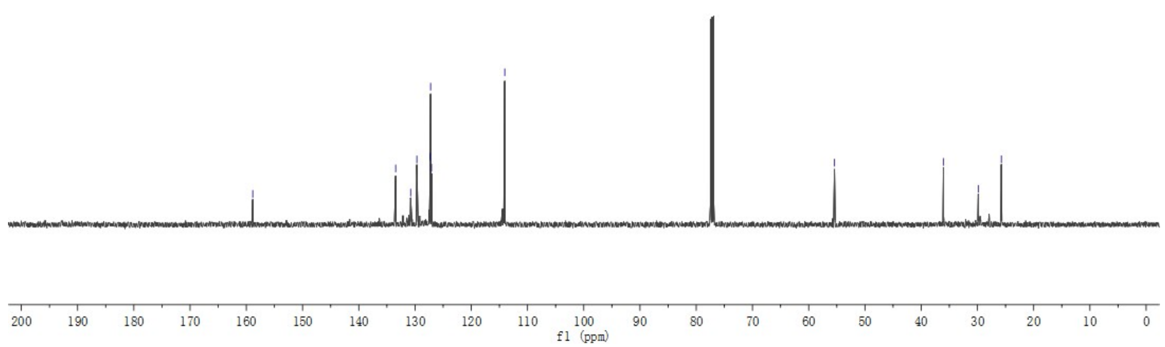

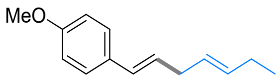

12

^{13}C NMR (126 MHz, $CDCl_3$)

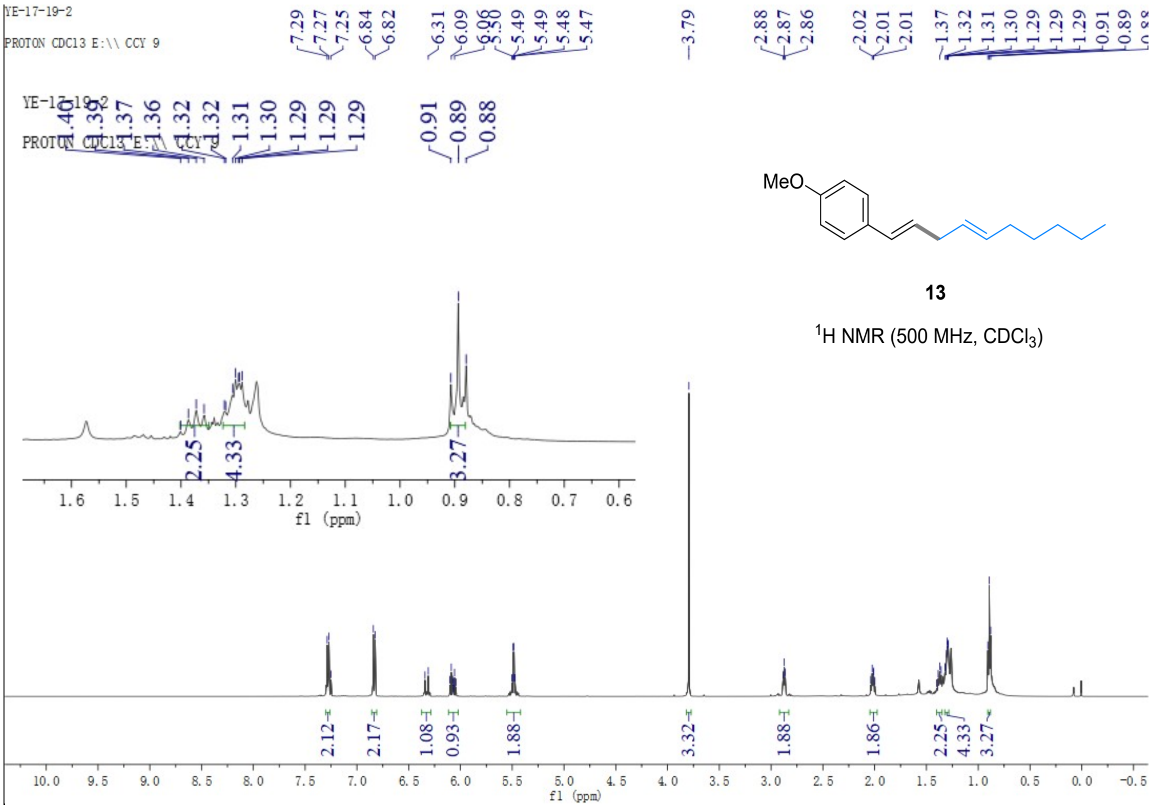

13

^{1}H NMR (500 MHz, $CDCl_3$)

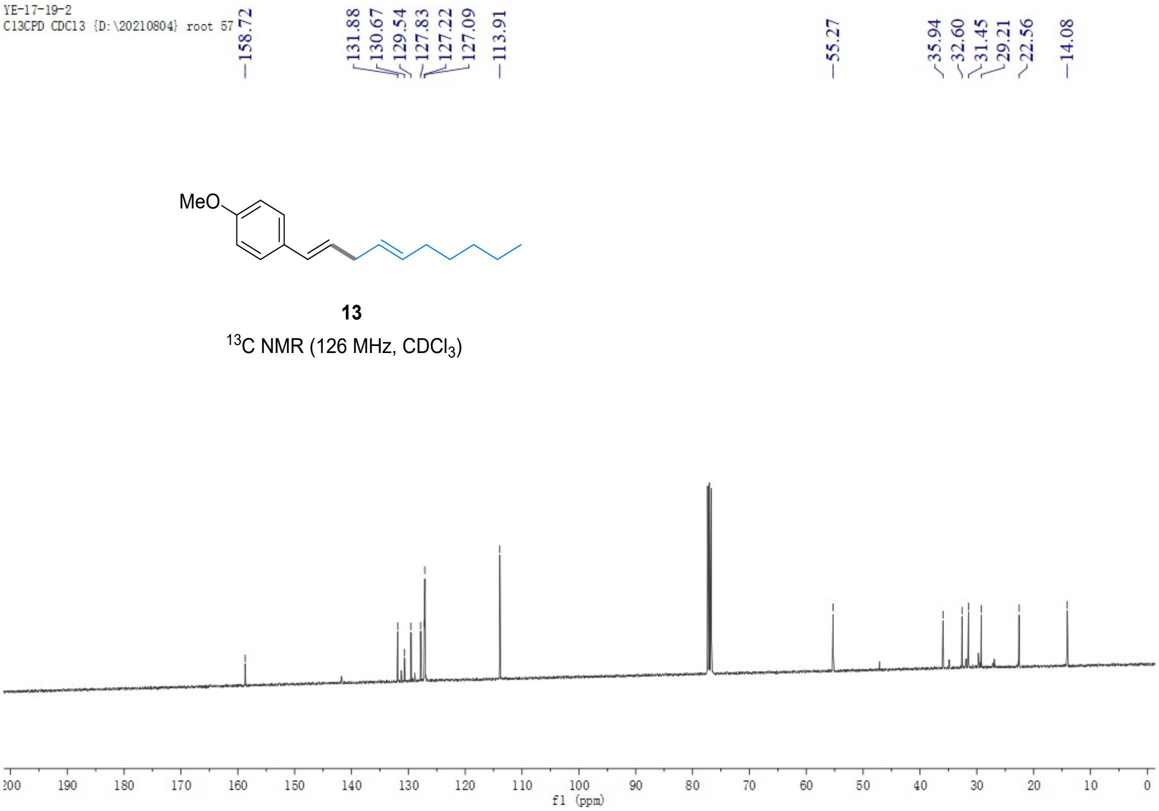

13

^{13}C NMR (126 MHz, $CDCl_3$)

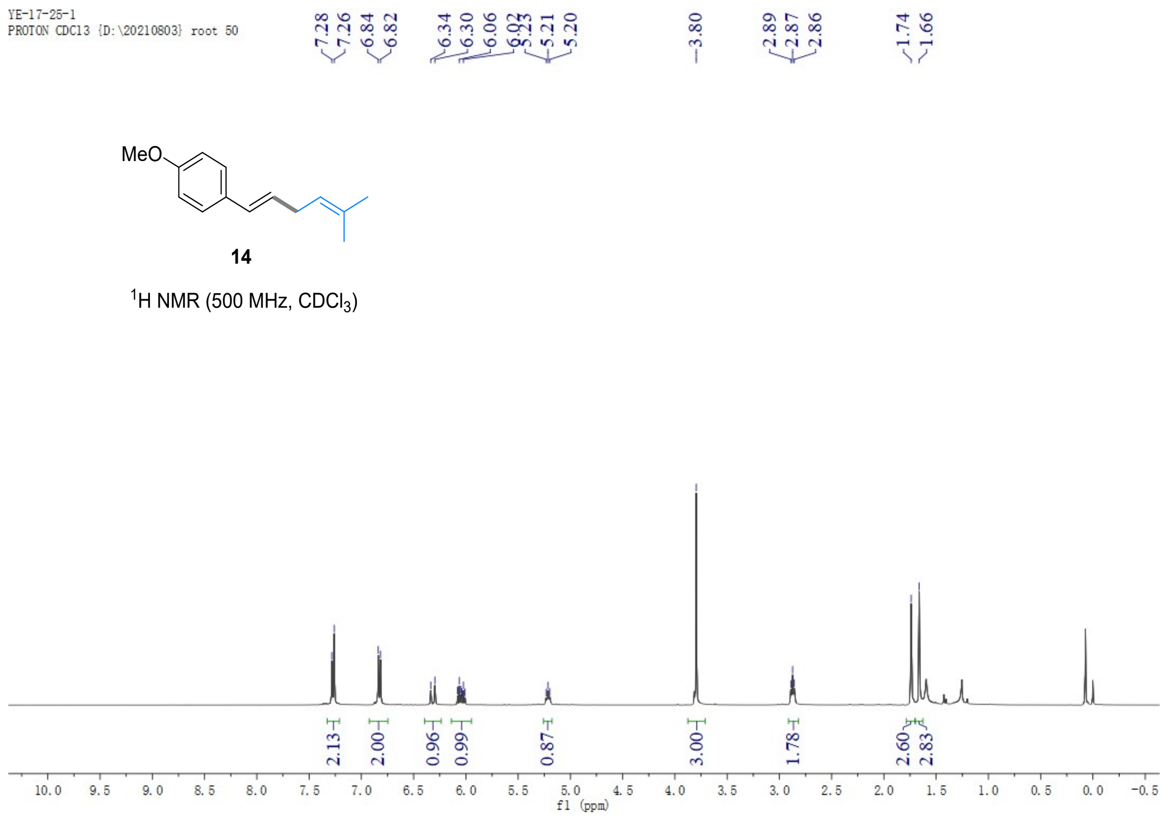
YE-17-25-1
PROTON CDCl3 {D:\20210803} root 50
7.28
7.26
6.84
6.82
6.34
6.30
6.06
6.02
5.23
5.21
5.20
3.80
2.89
2.87
2.86
1.74
1.66
MeO
14
1H NMR (500 MHz, CDCl3)
2.13
2.00
0.96
0.99
0.87
3.00
1.78
2.60
2.83
f1 (ppm)

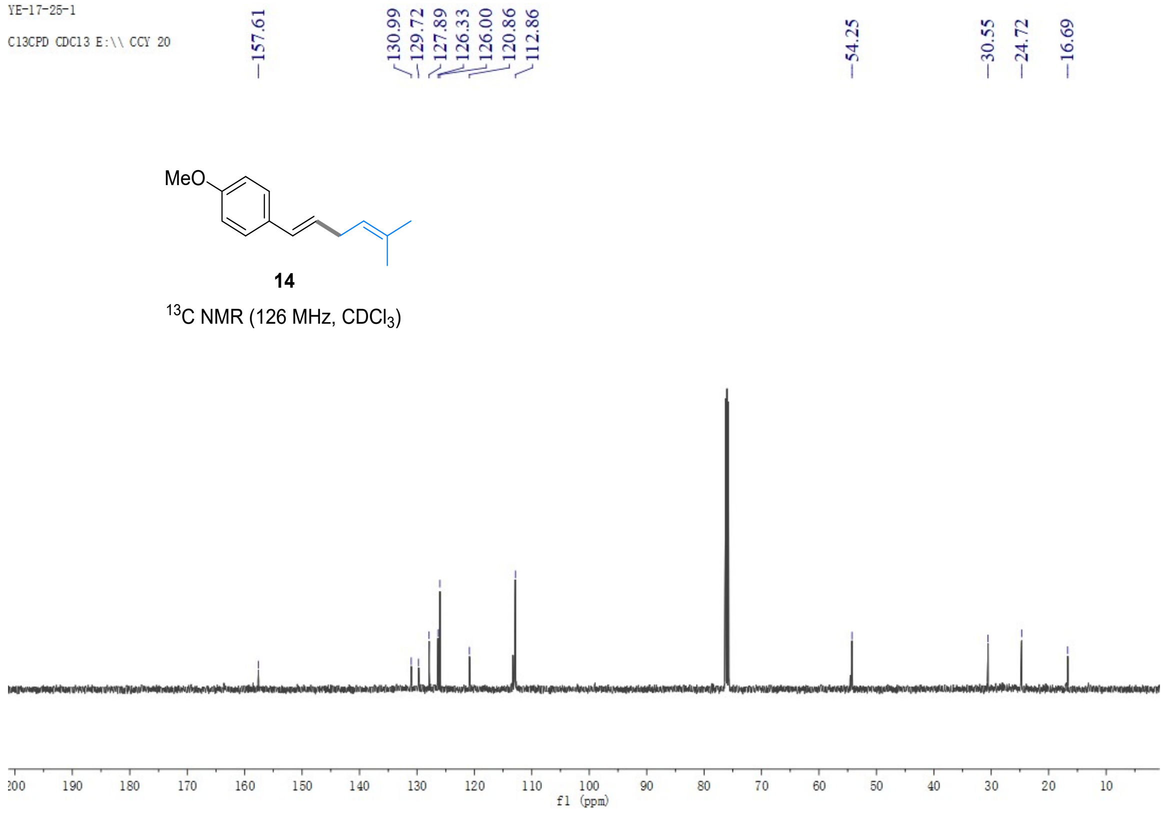
YE-17-25-1
C13CPD CDCl3 E:\\ CCY 20
157.61
130.99
129.72
127.89
126.33
126.00
120.86
112.86
54.25
30.55
24.72
16.69
MeO
14
13C NMR (126 MHz, CDCl3)
f1 (ppm)

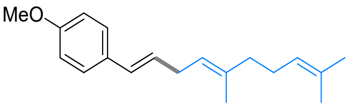

15

^{1}H NMR (500 MHz, $CDCl_3$)

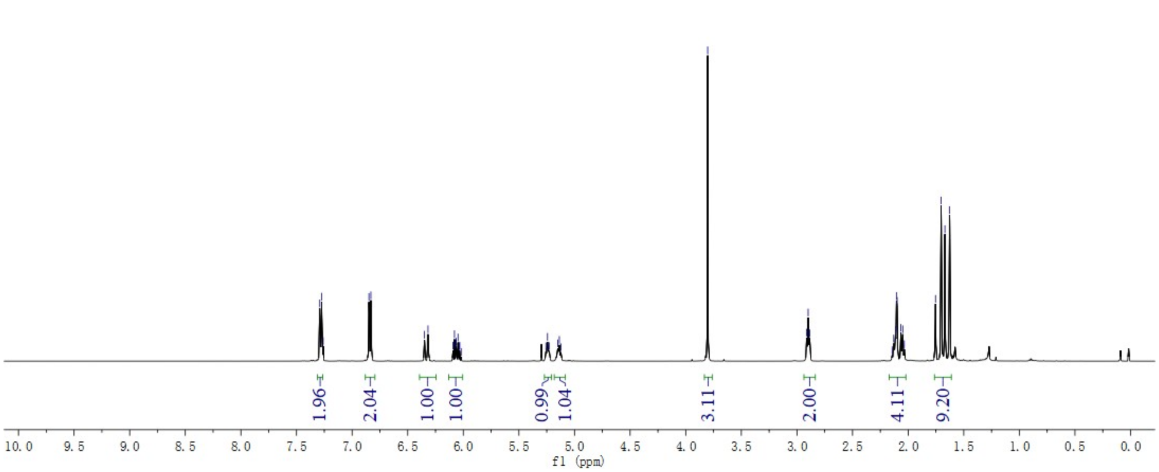

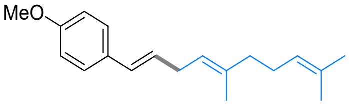

15

^{13}C NMR (126 MHz, $CDCl_3$)

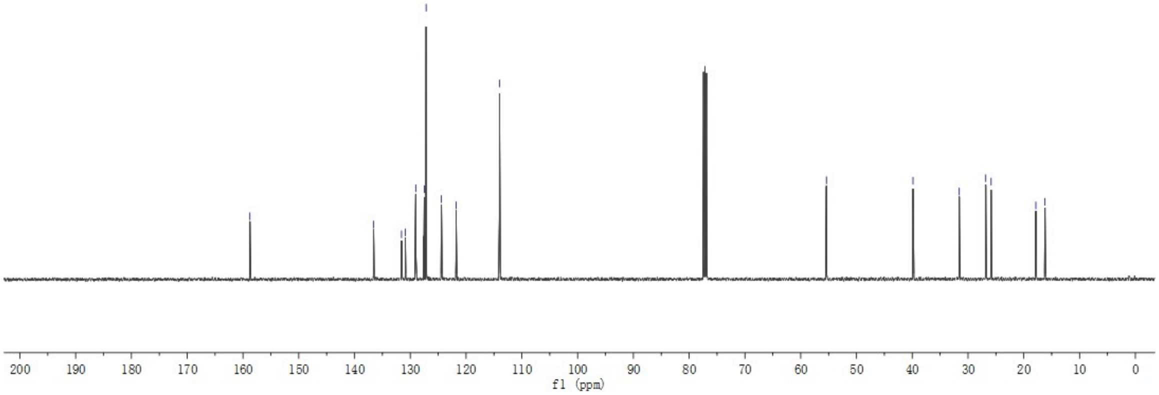

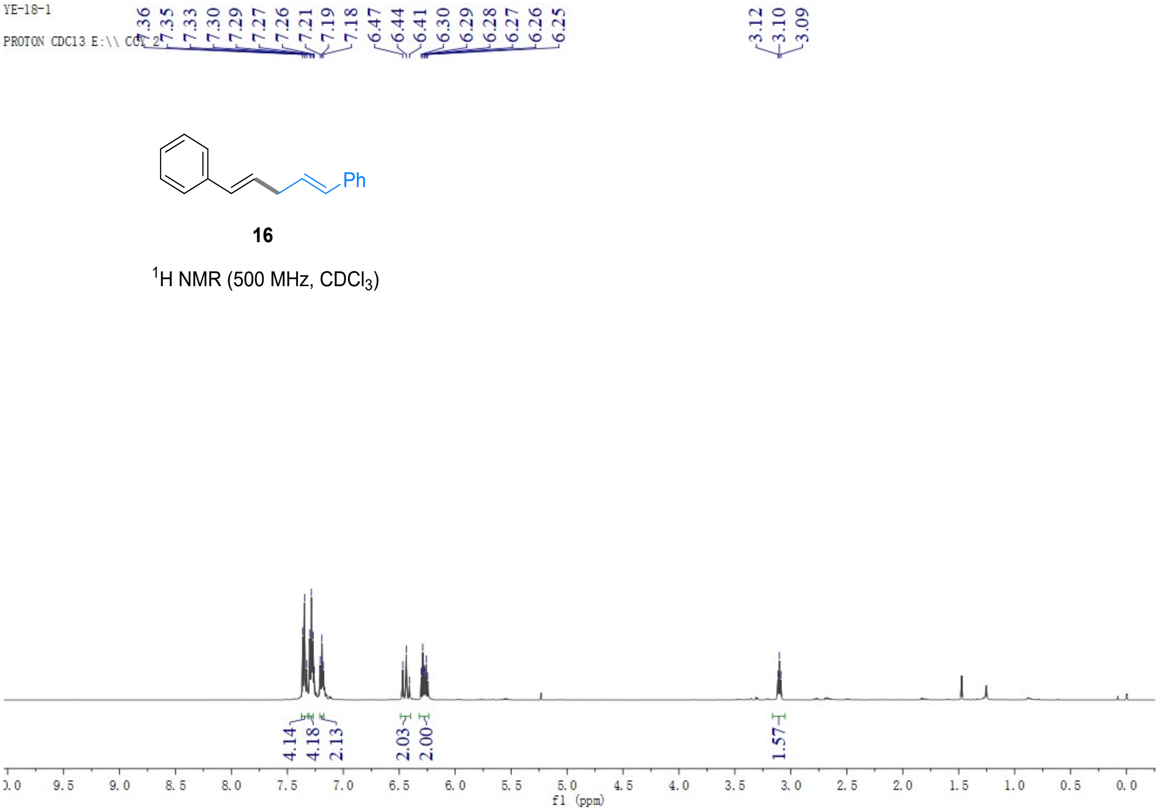

16

^{1}H NMR (500 MHz, $CDCl_3$)

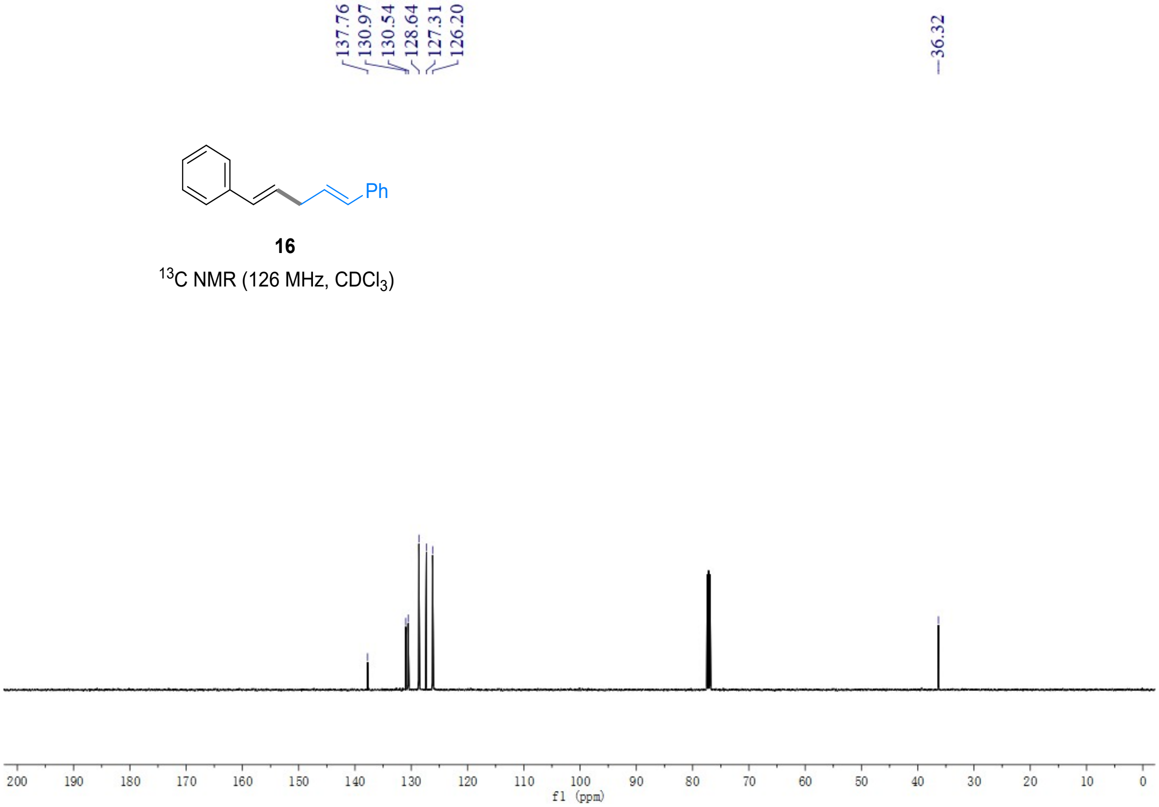

16

^{13}C NMR (126 MHz, $CDCl_3$)

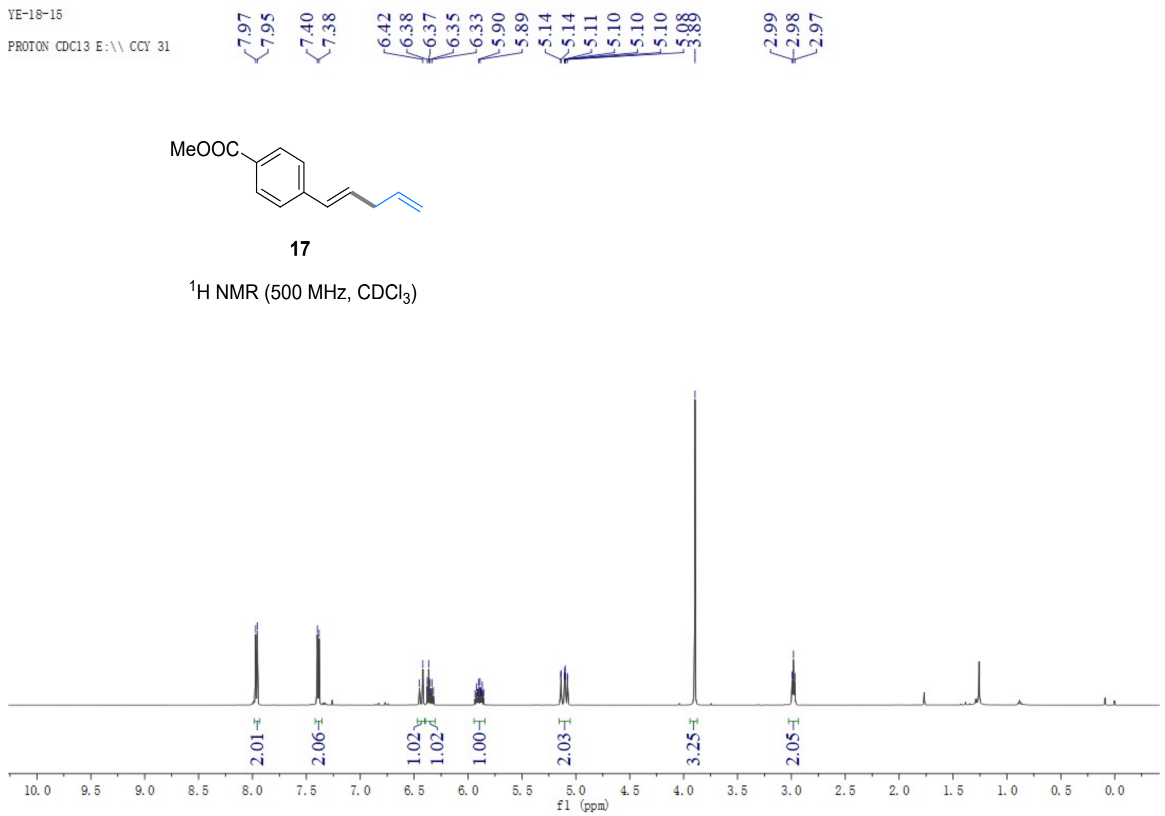
YE-18-15
PROTON CDCl3 E:\\ CCY 31
7.97
7.95
7.40
7.38
6.42
6.38
6.37
6.35
6.33
5.90
5.89
5.14
5.14
5.11
5.10
5.10
5.10
5.08
3.89
2.99
2.98
2.97
MeOOC
17
1H NMR (500 MHz, CDCl3)
2.01
2.06
1.02
1.02
1.00
2.03
3.25
2.05
f1 (ppm)

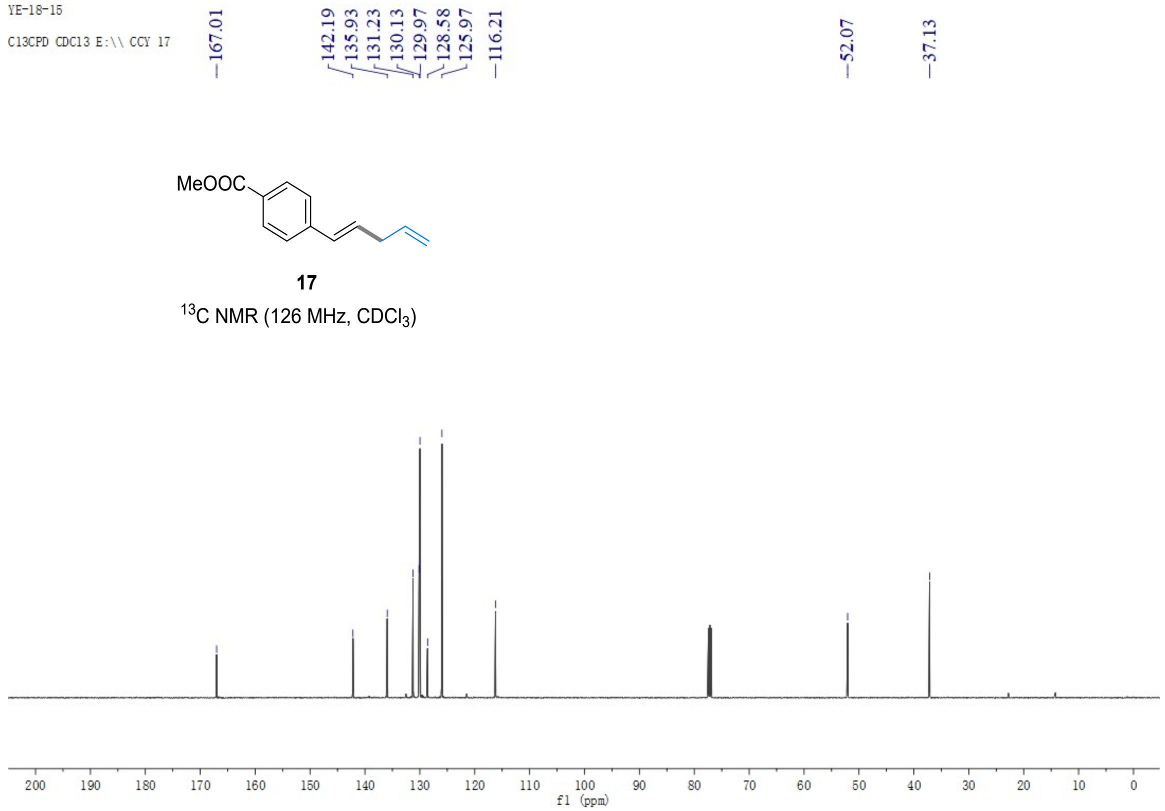
YE-18-15
C13CPD CDCl3 E:\\ CCY 17
167.01
142.19
135.93
131.23
130.13
129.97
128.58
125.97
116.21
52.07
37.13
MeOOC
17
13C NMR (126 MHz, CDCl3)
f1 (ppm)

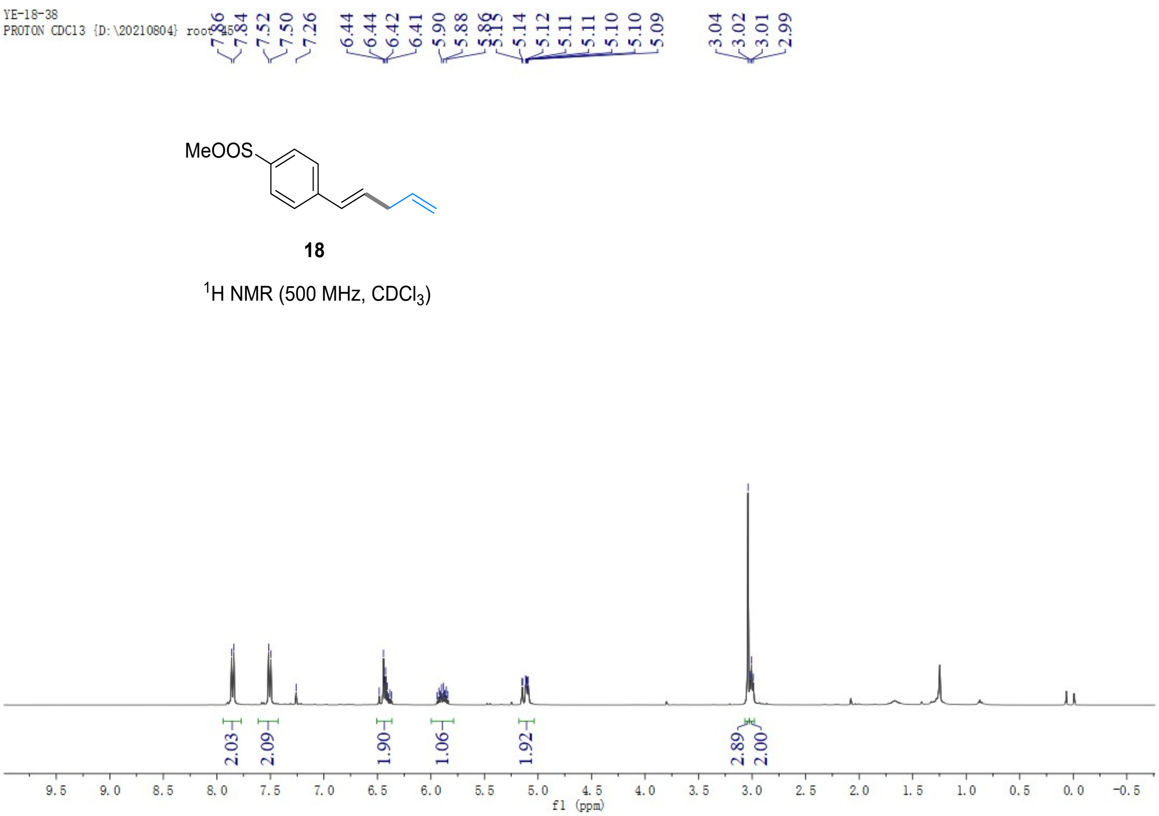
YE-18-38
PROTON CDCl3 {D:\20210804} root 45
7.86 7.84 7.52 7.50 7.26 6.44 6.44 6.42 6.41 5.90 5.88 5.86 5.15 5.14 5.12 5.11 5.11 5.10 5.10 5.09 3.04 3.02 3.01 2.99
MeOOS
18
1H NMR (500 MHz, CDCl3)
2.03 2.09 1.90 1.06 1.92 2.89 2.00
9.5 9.0 8.5 8.0 7.5 7.0 6.5 6.0 5.5 5.0 4.5 4.0 3.5 3.0 2.5 2.0 1.5 1.0 0.5 0.0 -0.5
f1 (ppm)

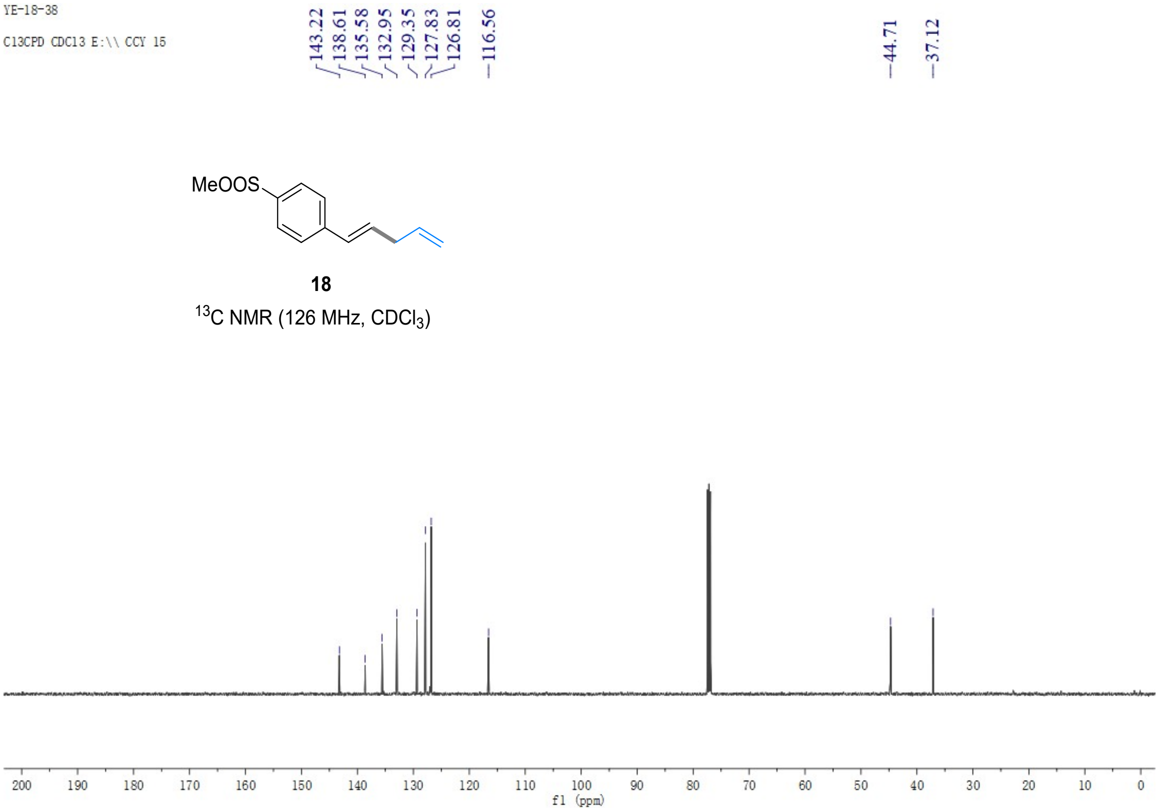
YE-18-38
C13CPD CDCl3 E:\\ CCY 15
143.22 138.61 135.58 132.95 129.35 127.83 126.81 116.56 44.71 37.12
MeOOS
18
13C NMR (126 MHz, CDCl3)
200 190 180 170 160 150 140 130 120 110 100 90 80 70 60 50 40 30 20 10 0
f1 (ppm)

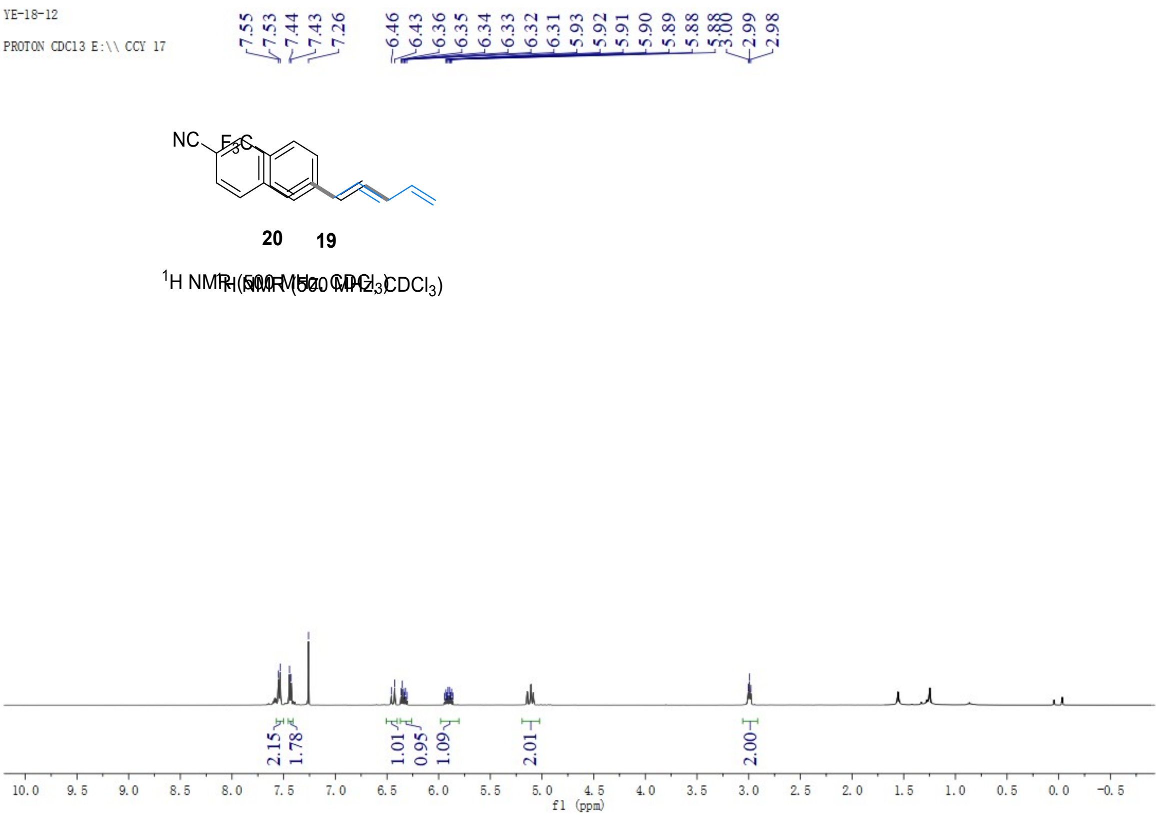

^{1}H NMR (500 MHz, $CDCl_3$)

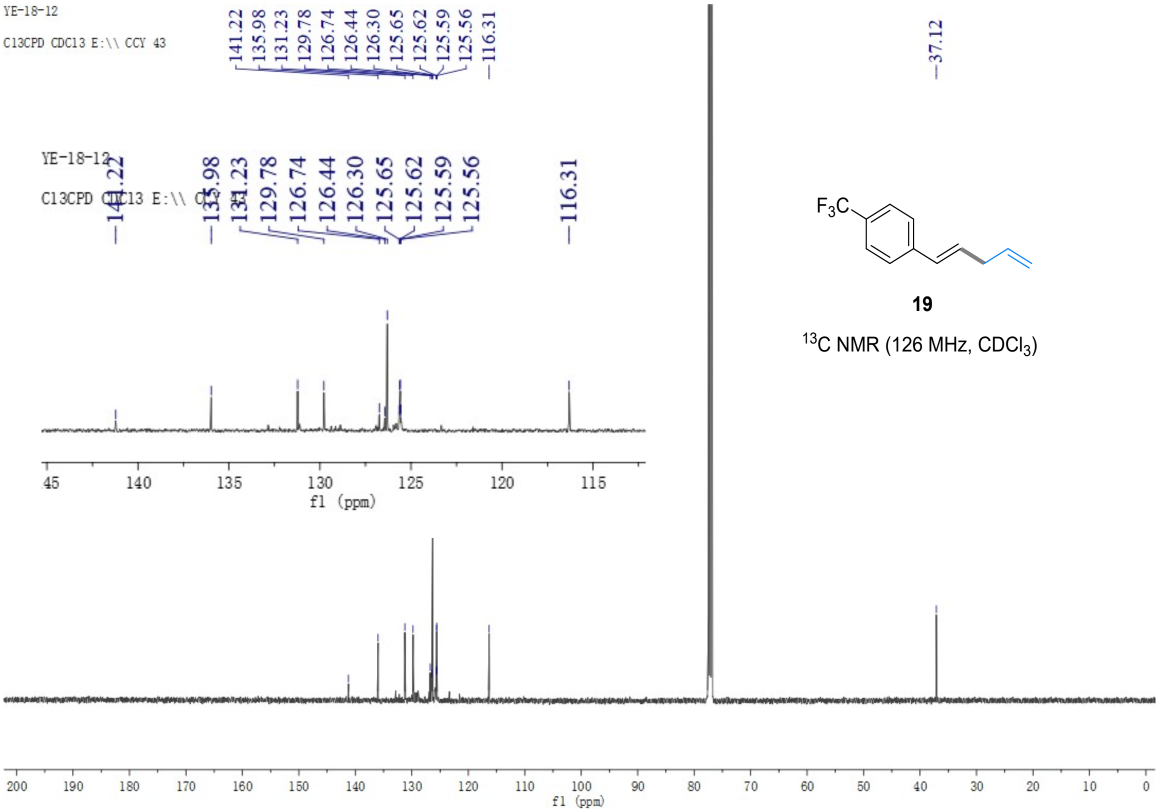

^{13}C NMR (126 MHz, $CDCl_3$)

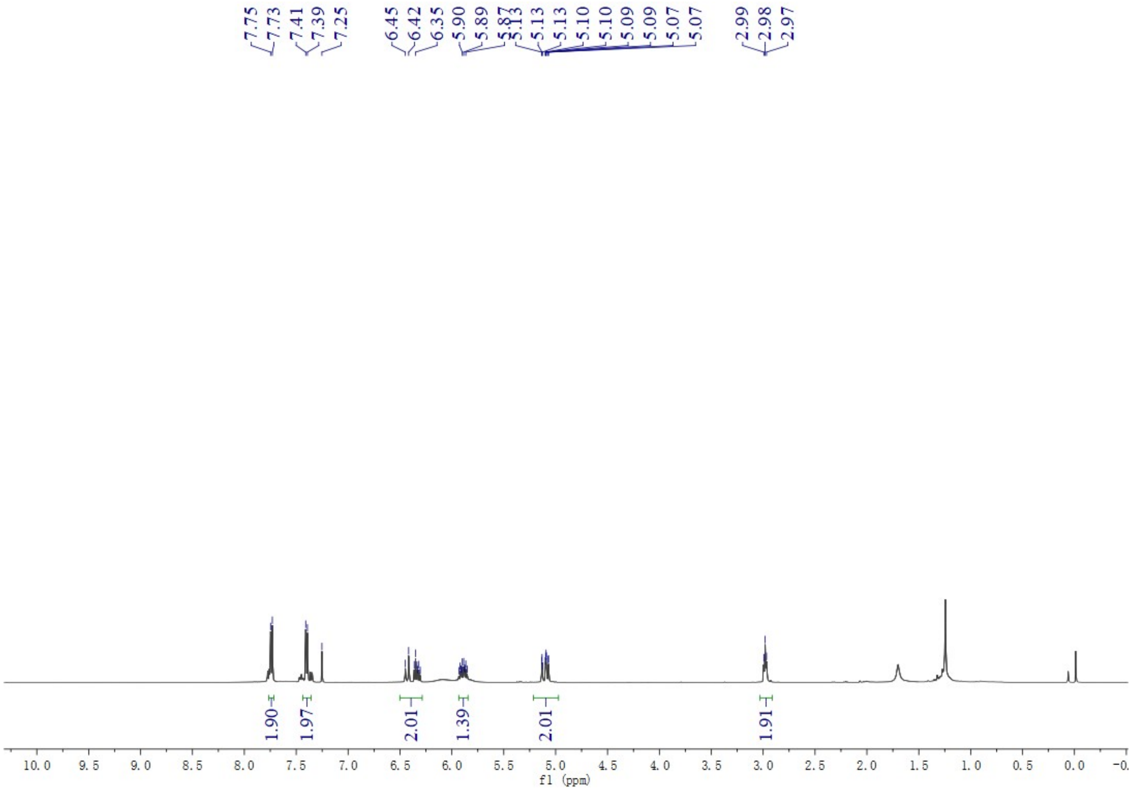

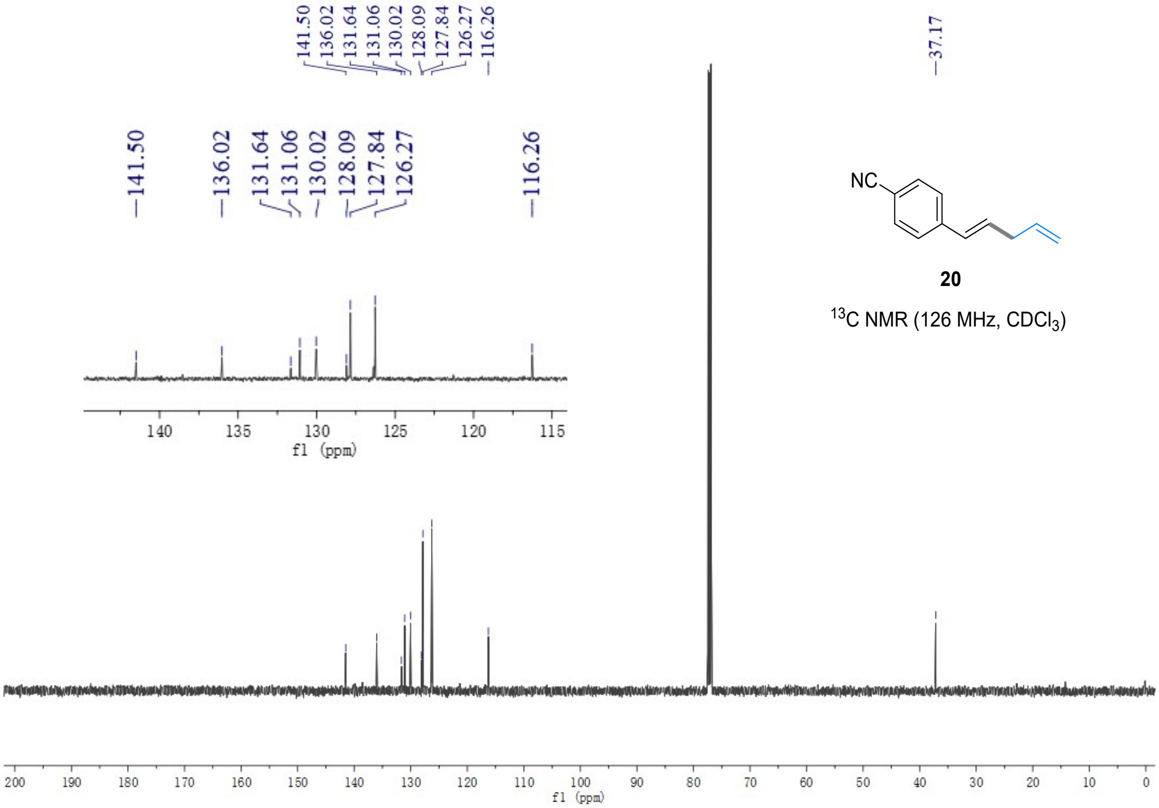

20

^{13}C NMR (126 MHz, $CDCl_3$)

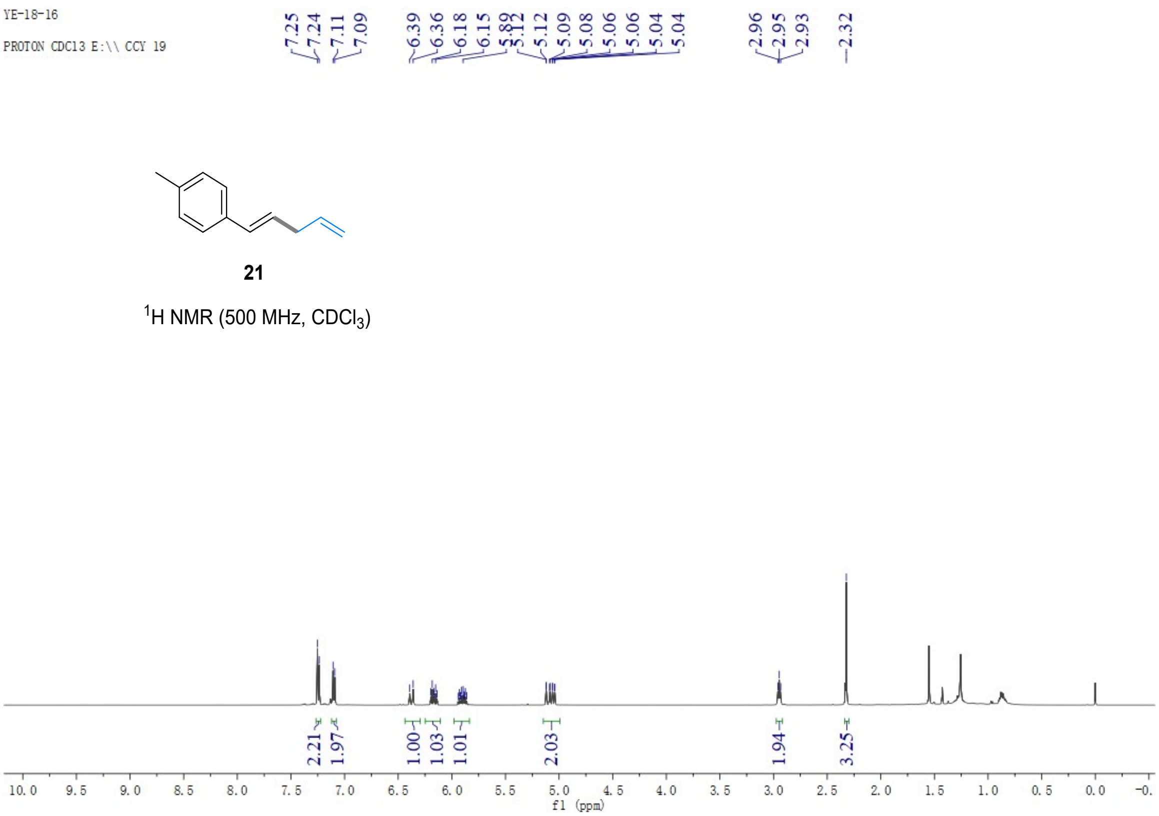
YE-18-16
PROTON CDC13 E:\\ CCY 19
7.25 7.24 7.11 7.09 6.39 6.36 6.18 6.15 5.89 5.12 5.09 5.08 5.06 5.06 5.04 5.04 2.96 2.95 2.93 2.32
21
1H NMR (500 MHz, CDCl3)
2.21 1.97 1.00 1.03 1.01 2.03 1.94 3.25
f1 (ppm)

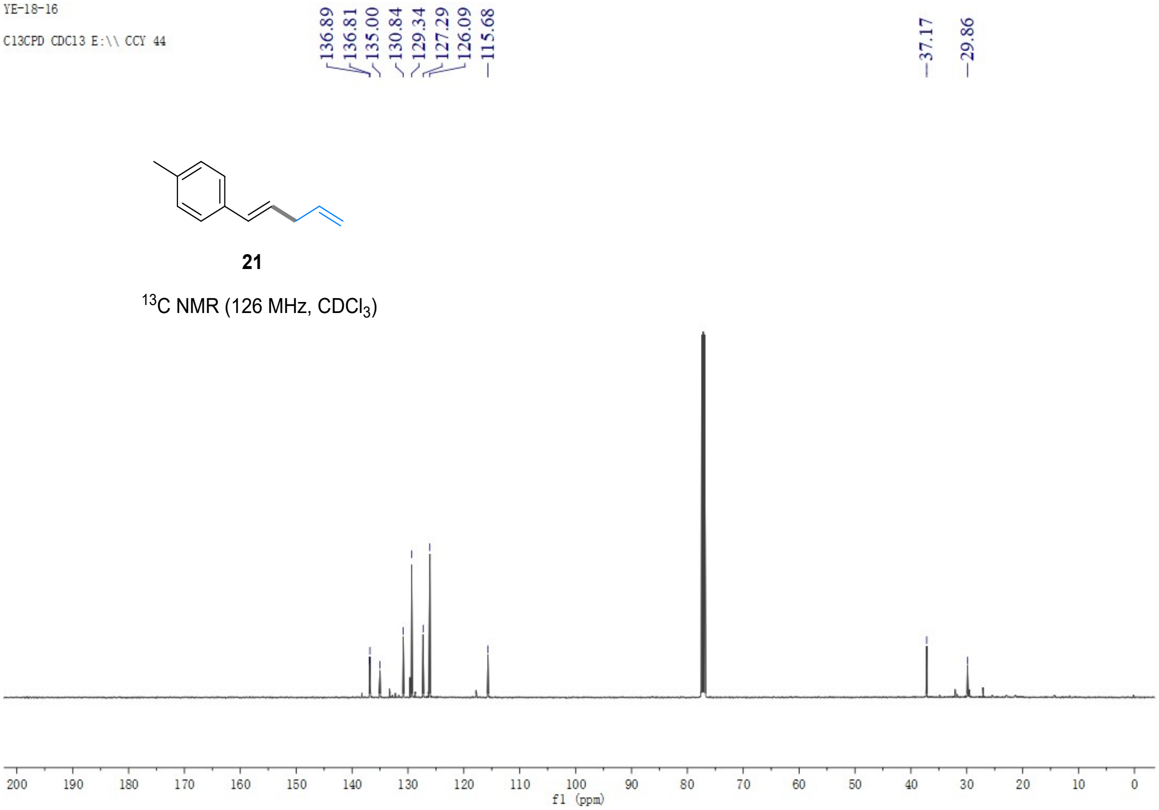
YE-18-16
C13CPD CDC13 E:\\ CCY 44
136.89 136.81 135.00 130.84 129.34 127.29 126.09 115.68 37.17 29.86
21
13C NMR (126 MHz, CDCl3)
f1 (ppm)

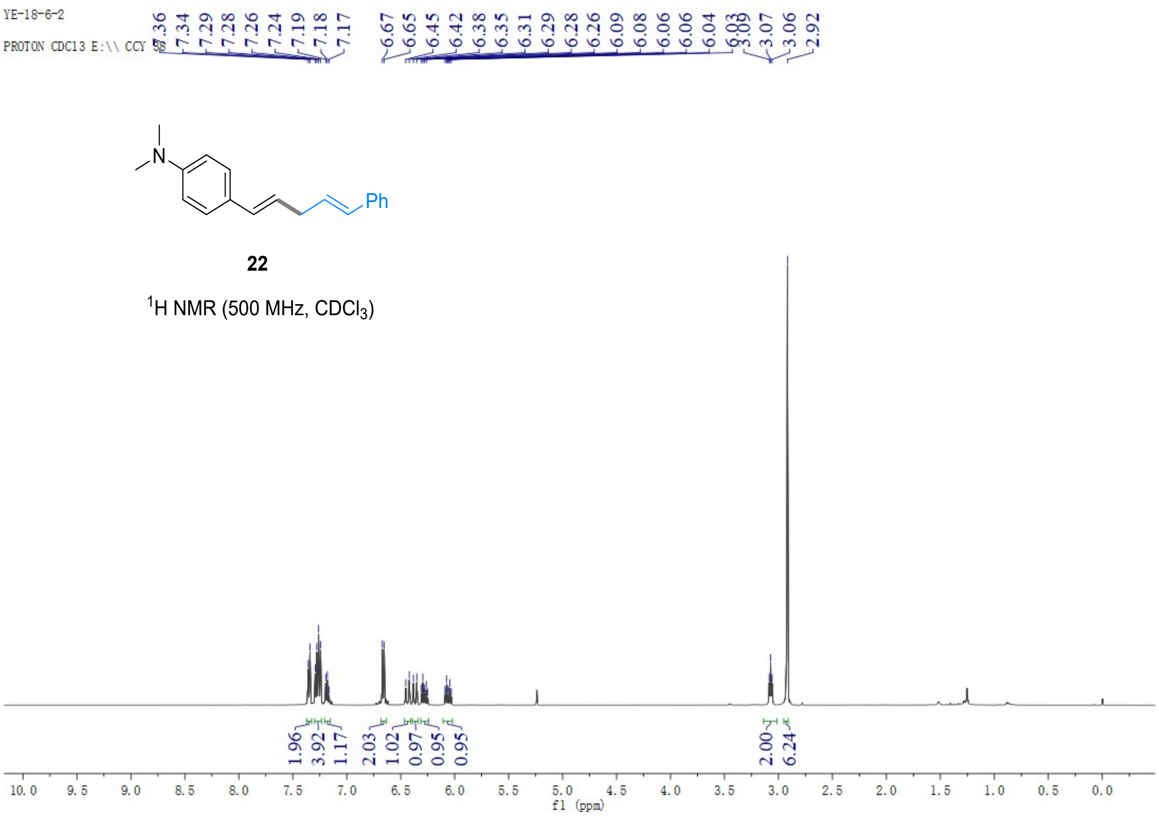
YE-18-6-2
PROTON CDCl3 E:\\ CCY 58
7.36 7.34 7.29 7.28 7.26 7.24 7.19 7.18 7.17
6.67 6.65 6.45 6.42 6.38 6.35 6.31 6.29 6.28 6.26 6.09 6.08 6.06 6.06 6.04 6.03 3.09 3.07 3.06 2.92
Ph
22
1H NMR (500 MHz, CDCl3)
1.96 3.92 1.17 2.03 1.02 0.97 0.95 0.95 2.00 6.24
f1 (ppm)

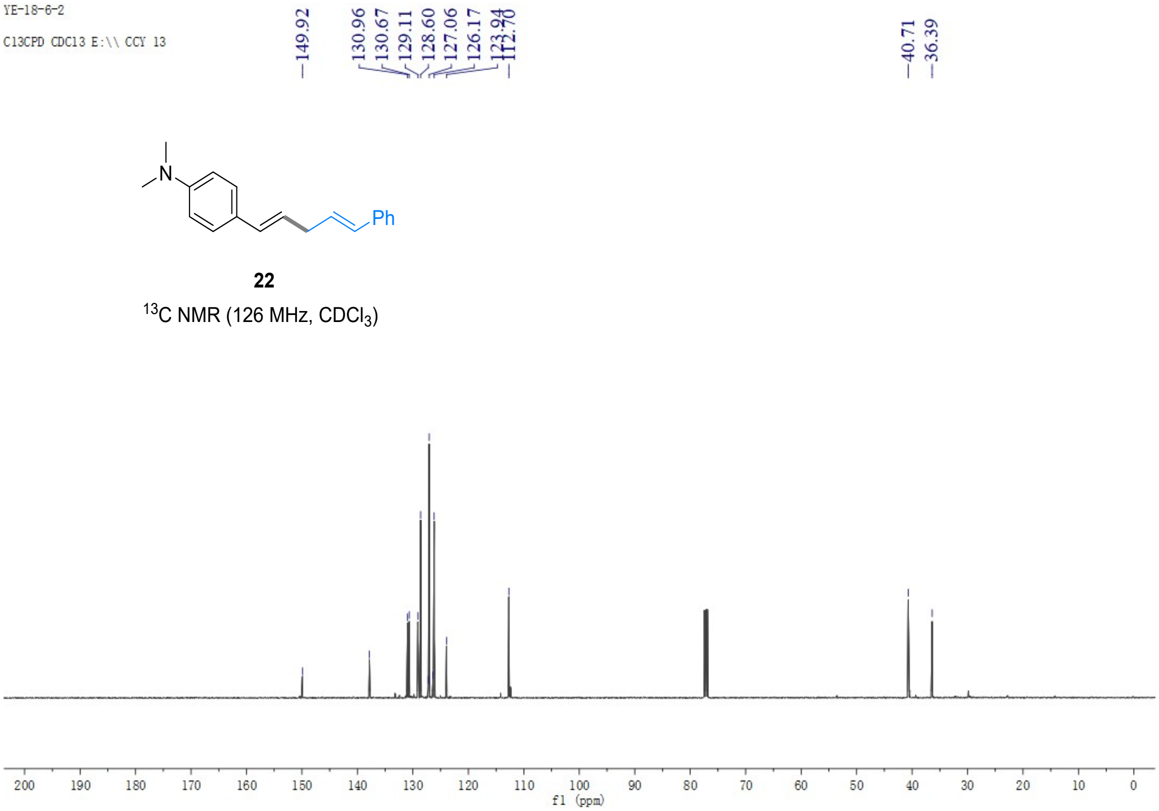
YE-18-6-2
C13CPD CDCl3 E:\\ CCY 13
149.92
130.96 130.67 129.11 128.60 127.06 126.17 123.94 112.70
40.71 36.39
Ph
22
13C NMR (126 MHz, CDCl3)
f1 (ppm)

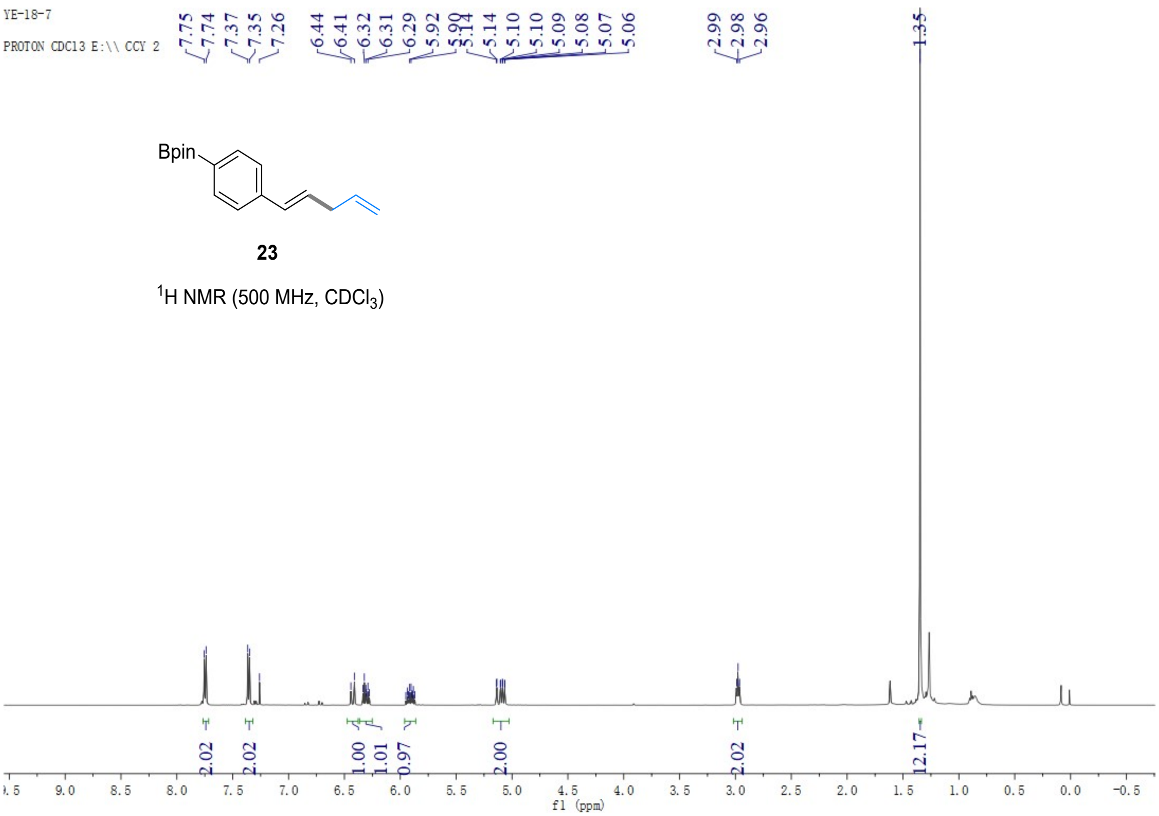

23

^{1}H NMR (500 MHz, $CDCl_3$)

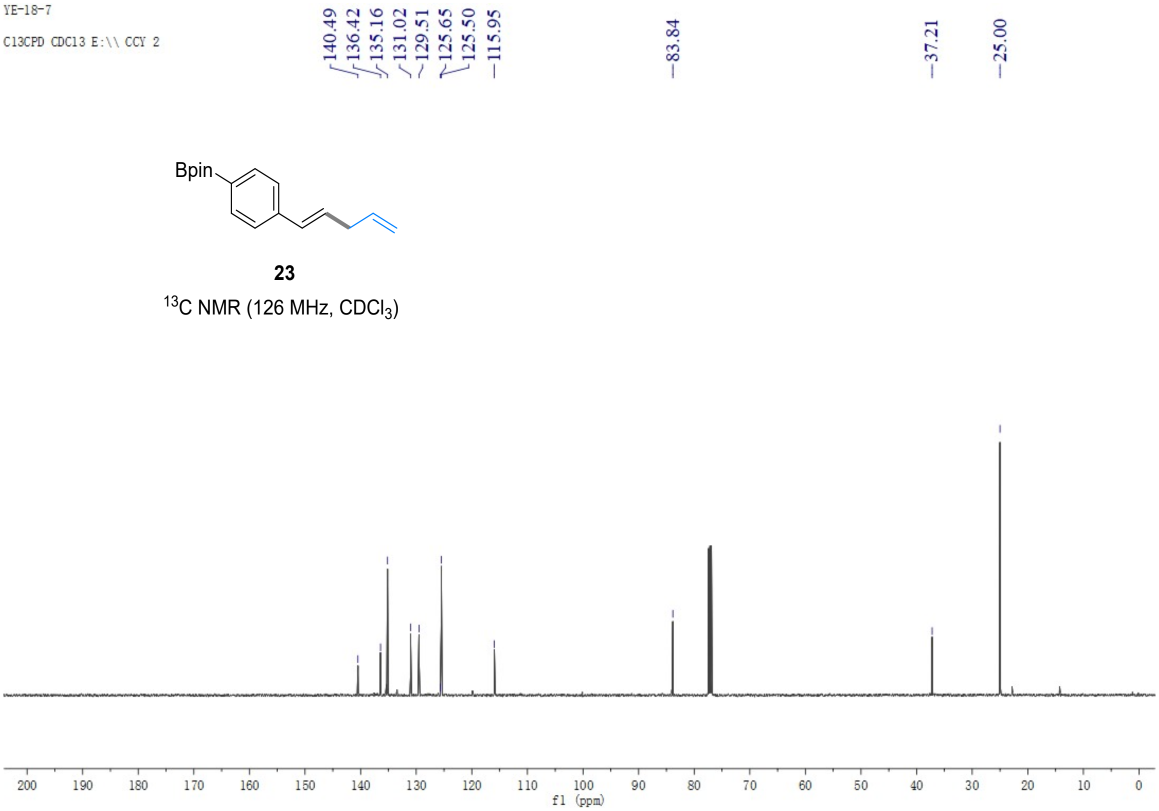

23

^{13}C NMR (126 MHz, $CDCl_3$)

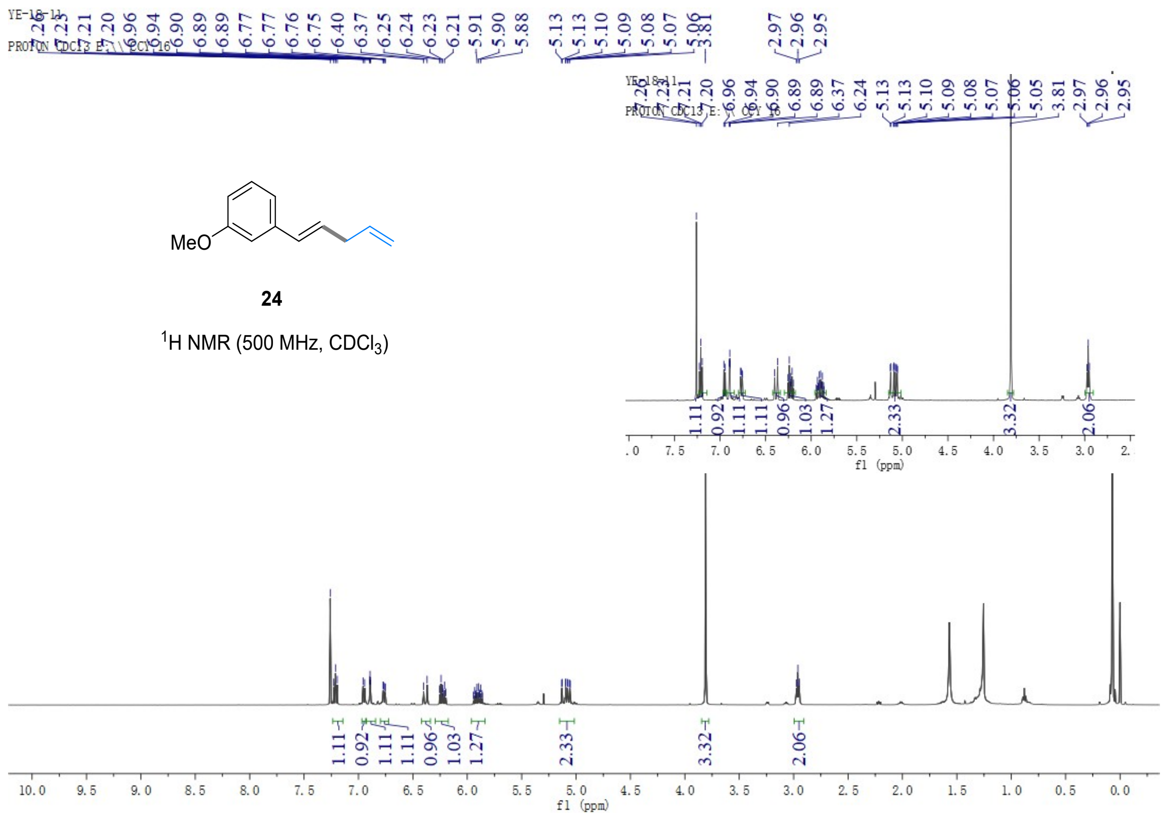

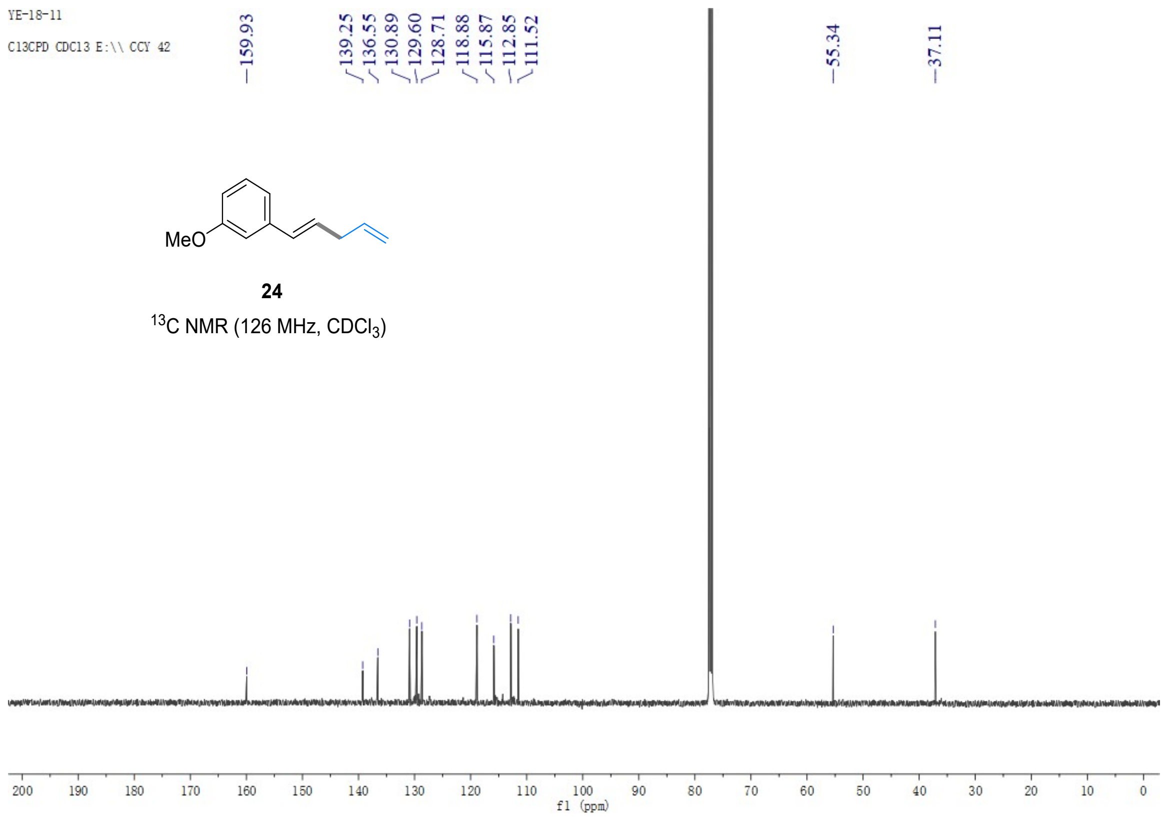

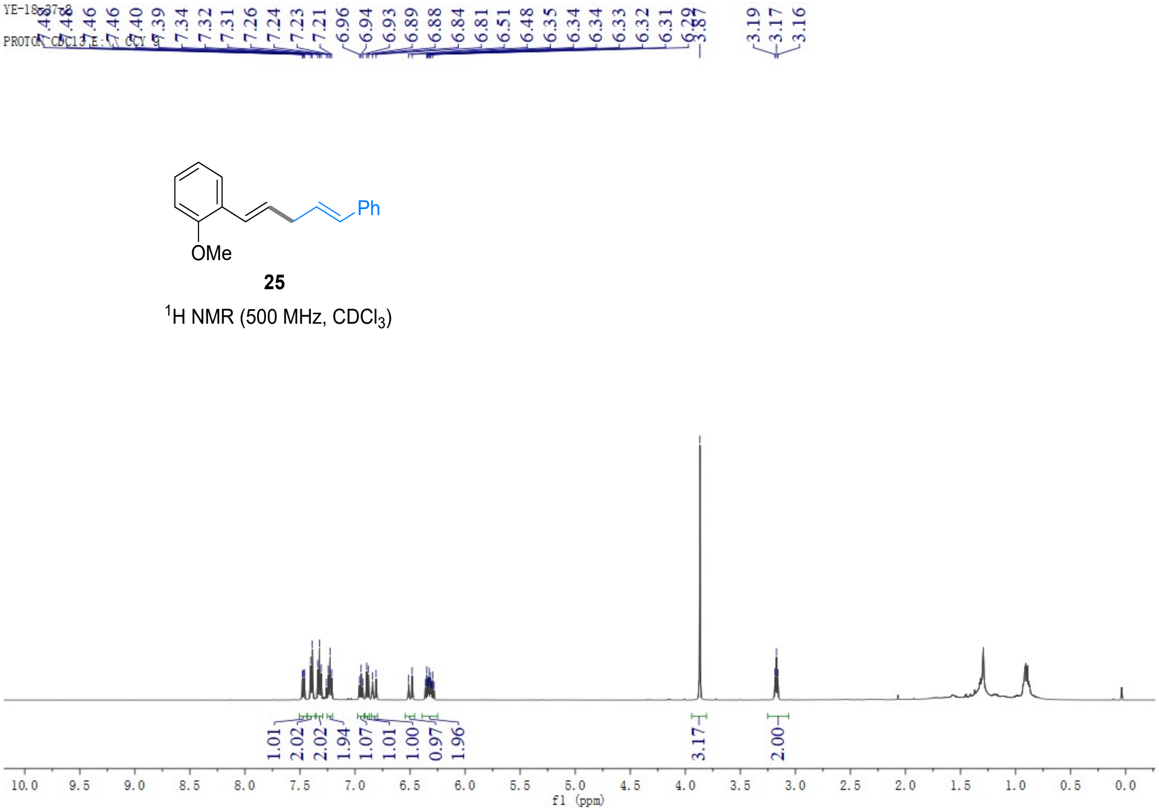

25

^{1}H NMR (500 MHz, $CDCl_3$)

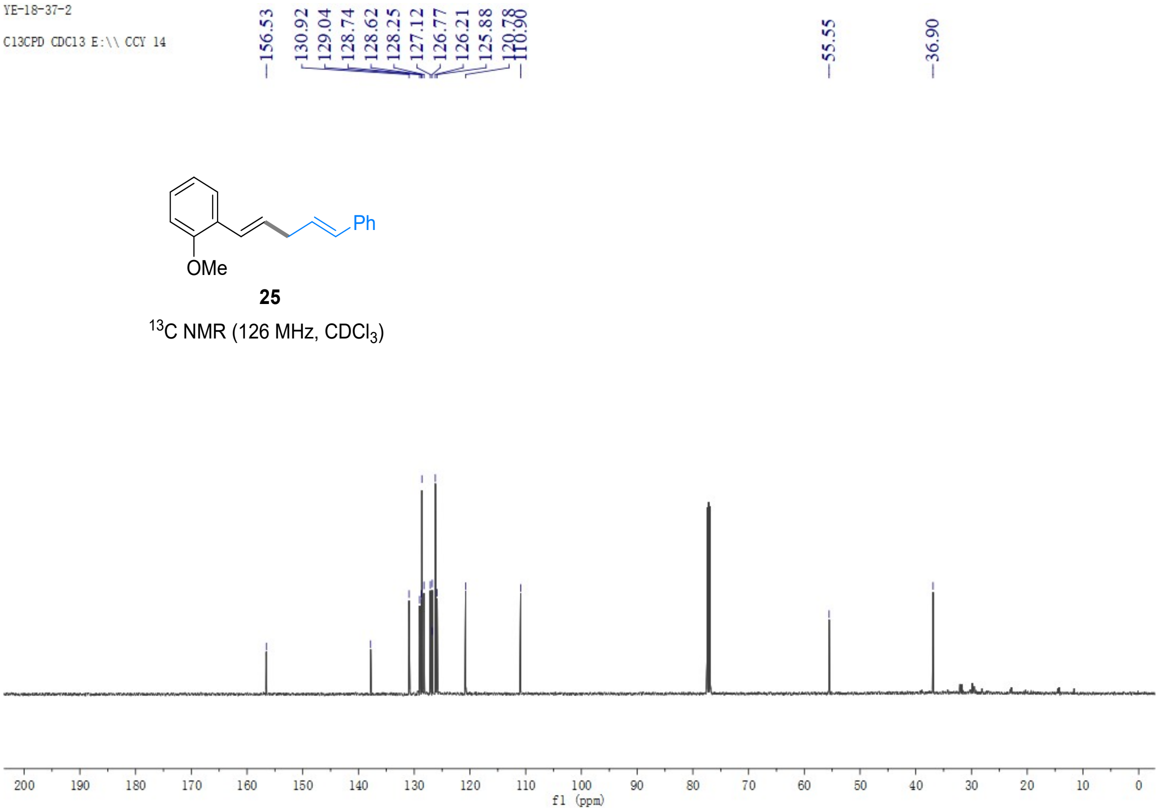

25

^{13}C NMR (126 MHz, $CDCl_3$)

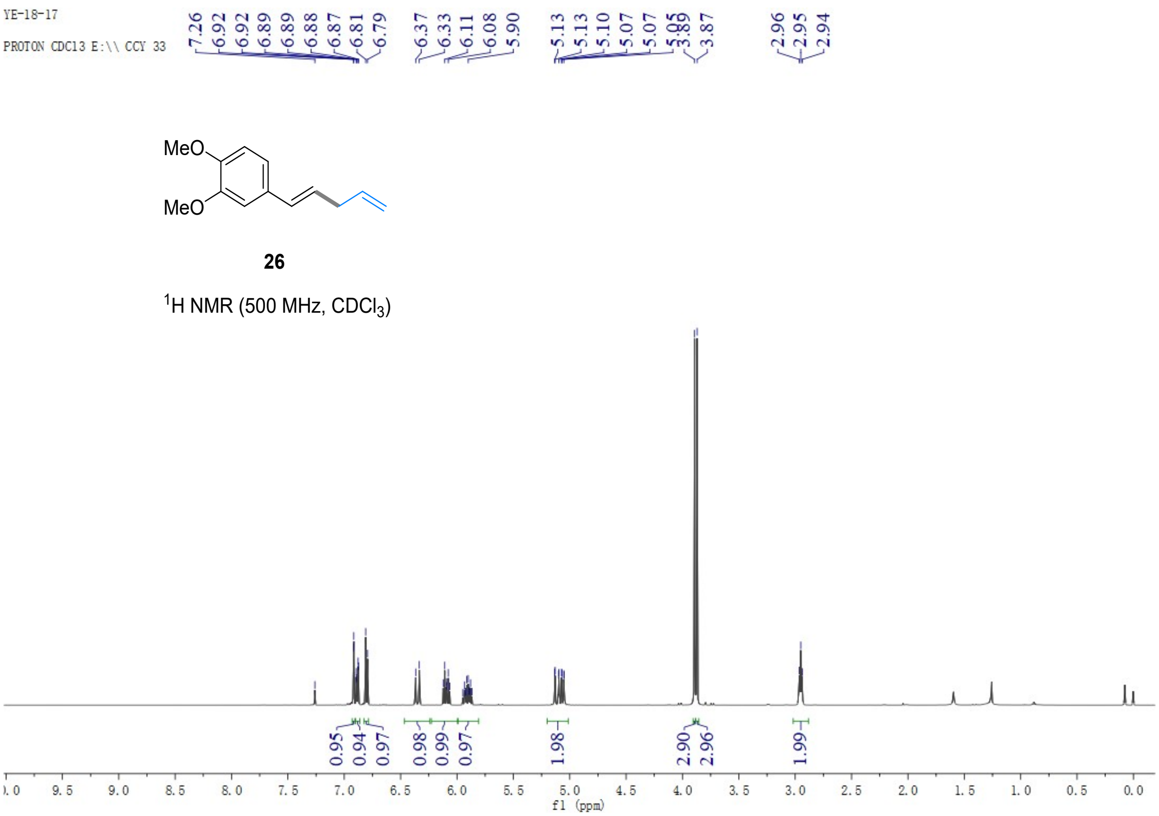

26

^{1}H NMR (500 MHz, $CDCl_3$)

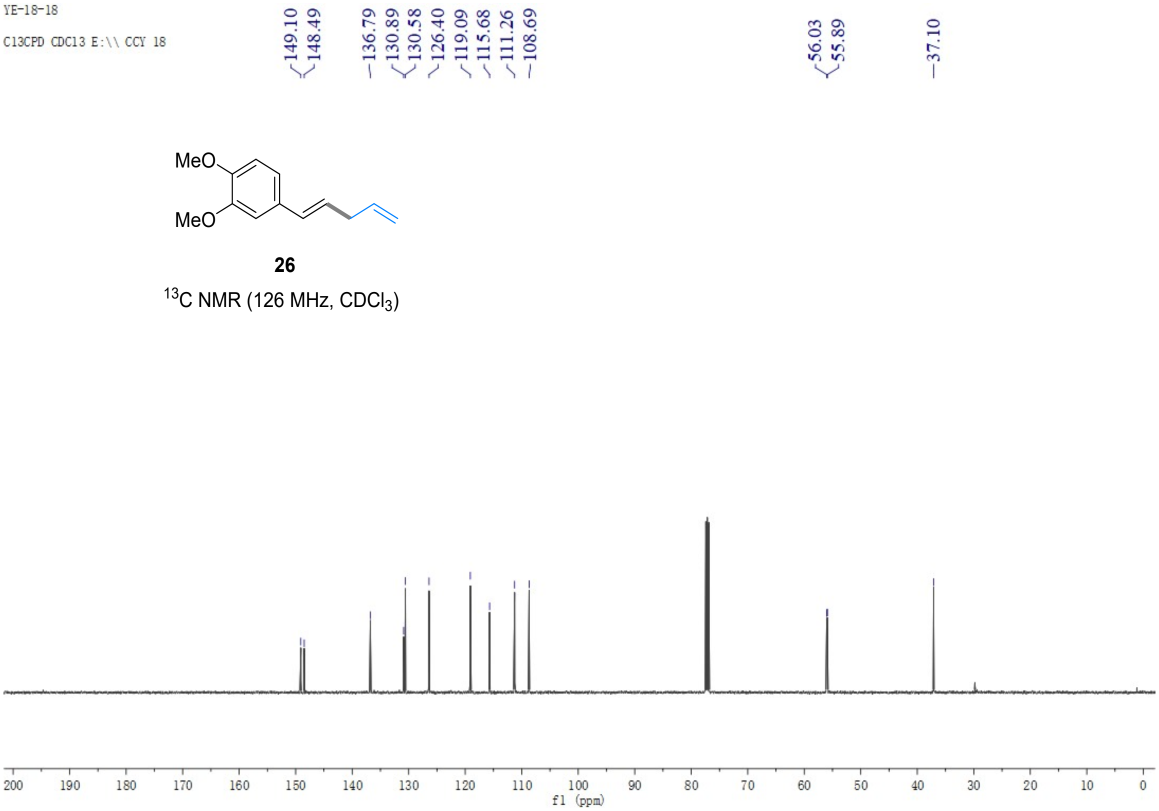

26

^{13}C NMR (126 MHz, $CDCl_3$)

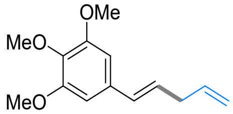

27

^{1}H NMR (500 MHz, $CDCl_3$)

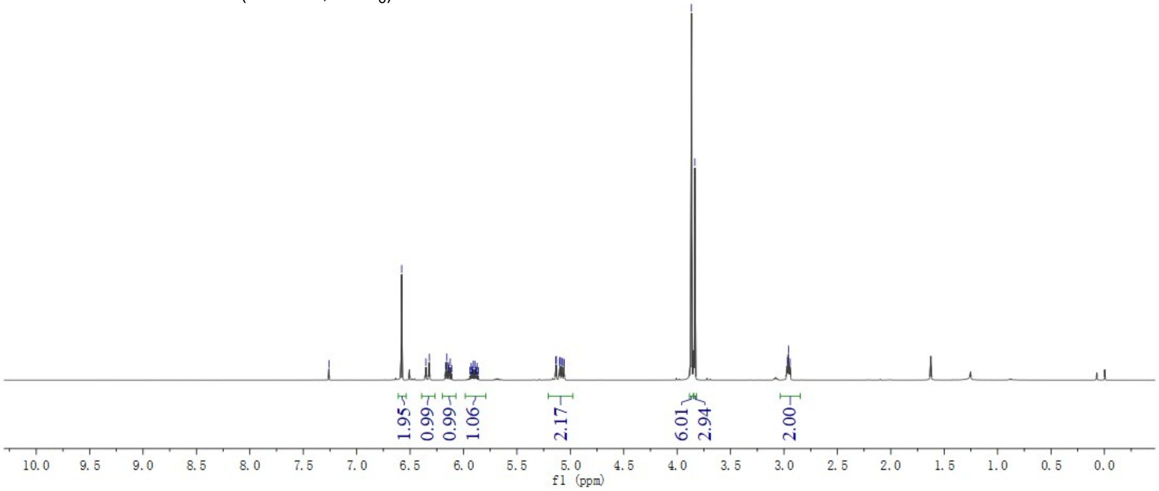

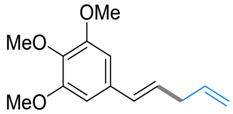

27

^{13}C NMR (126 MHz, $CDCl_3$)

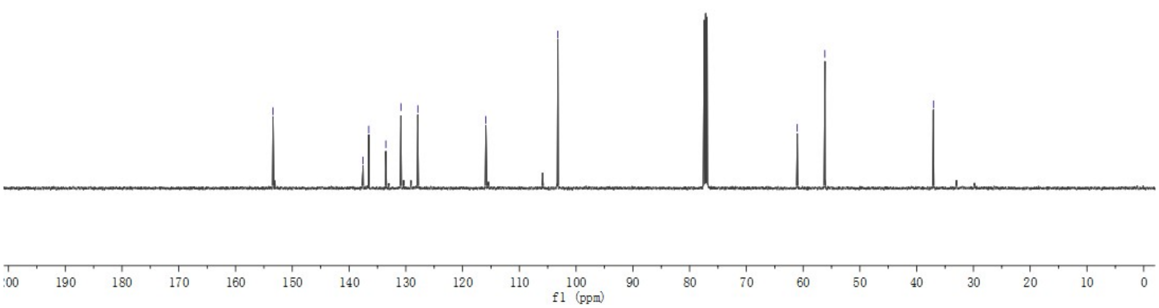

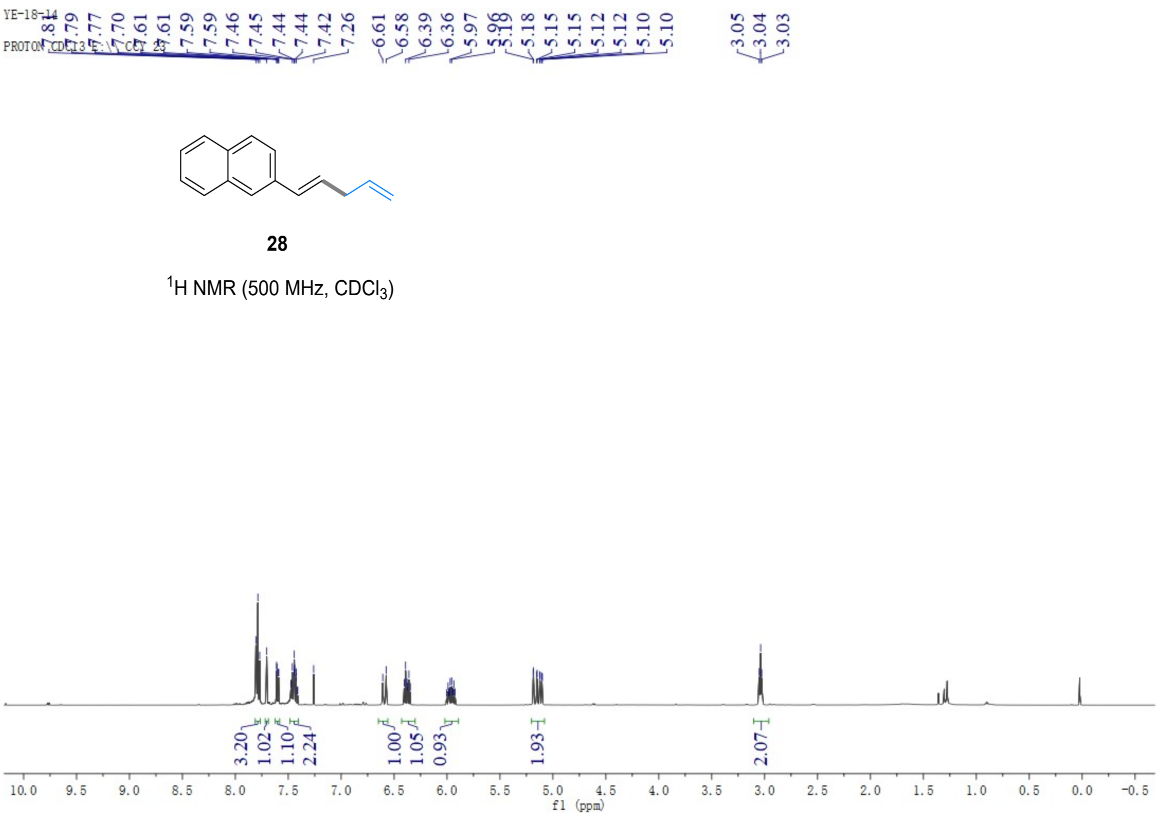

28

^{1}H NMR (500 MHz, $CDCl_3$)

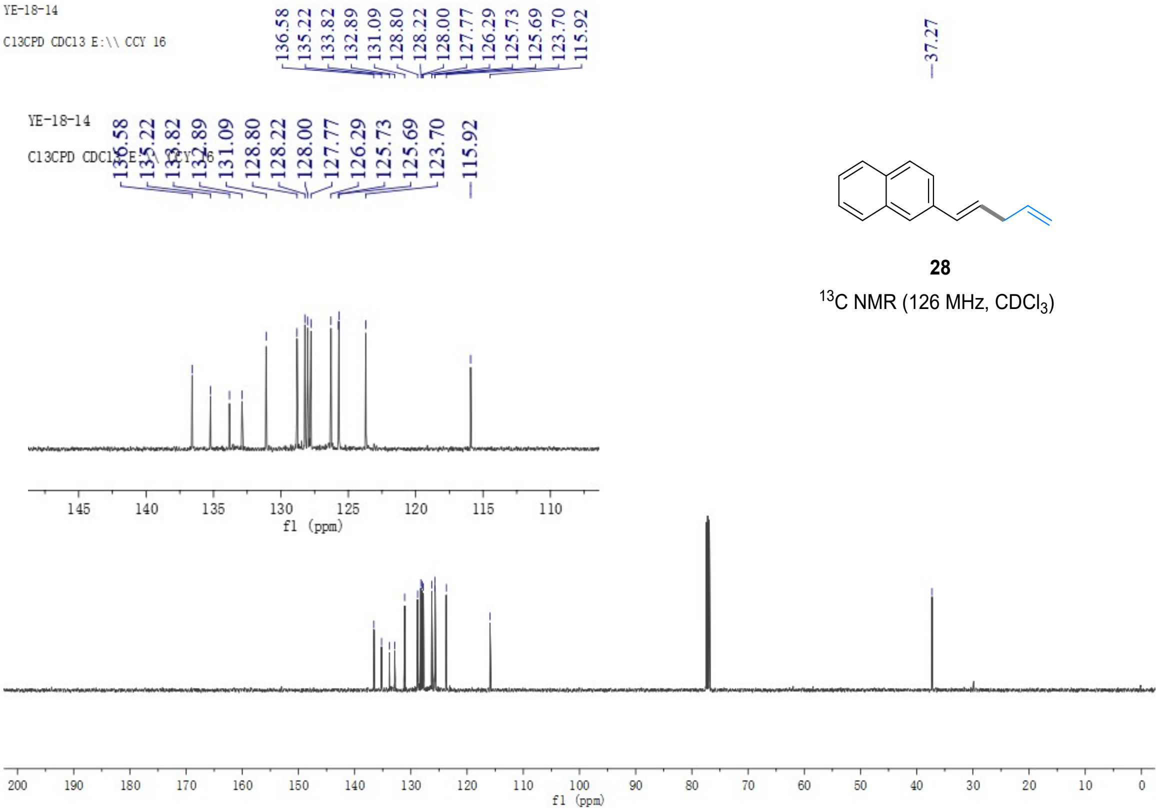

28

^{13}C NMR (126 MHz, $CDCl_3$)

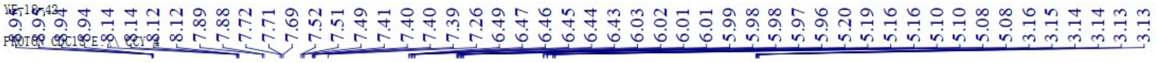

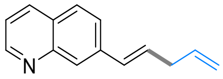

29

^{1}H NMR (500 MHz, $CDCl_3$)

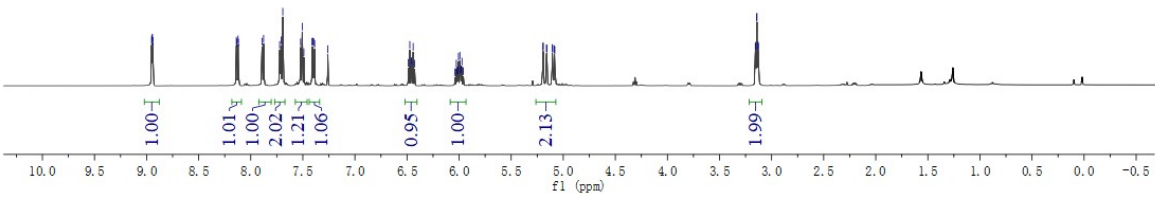

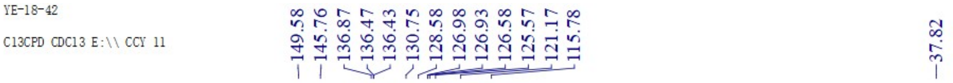

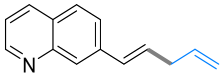

29

^{13}C NMR (126 MHz, $CDCl_3$)

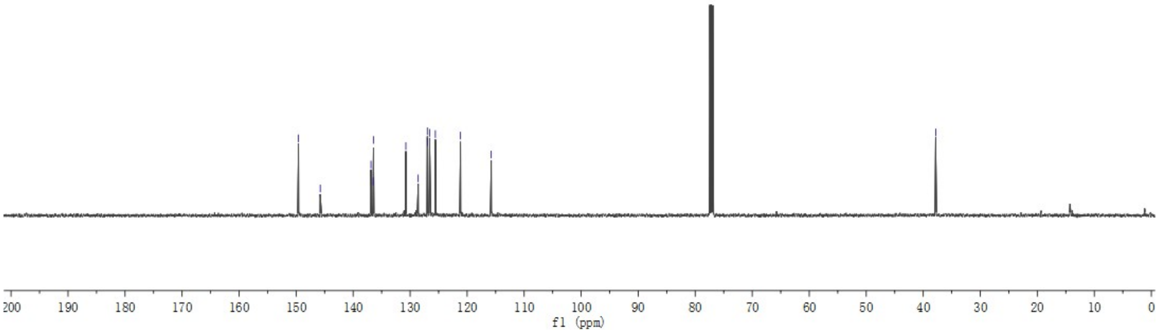

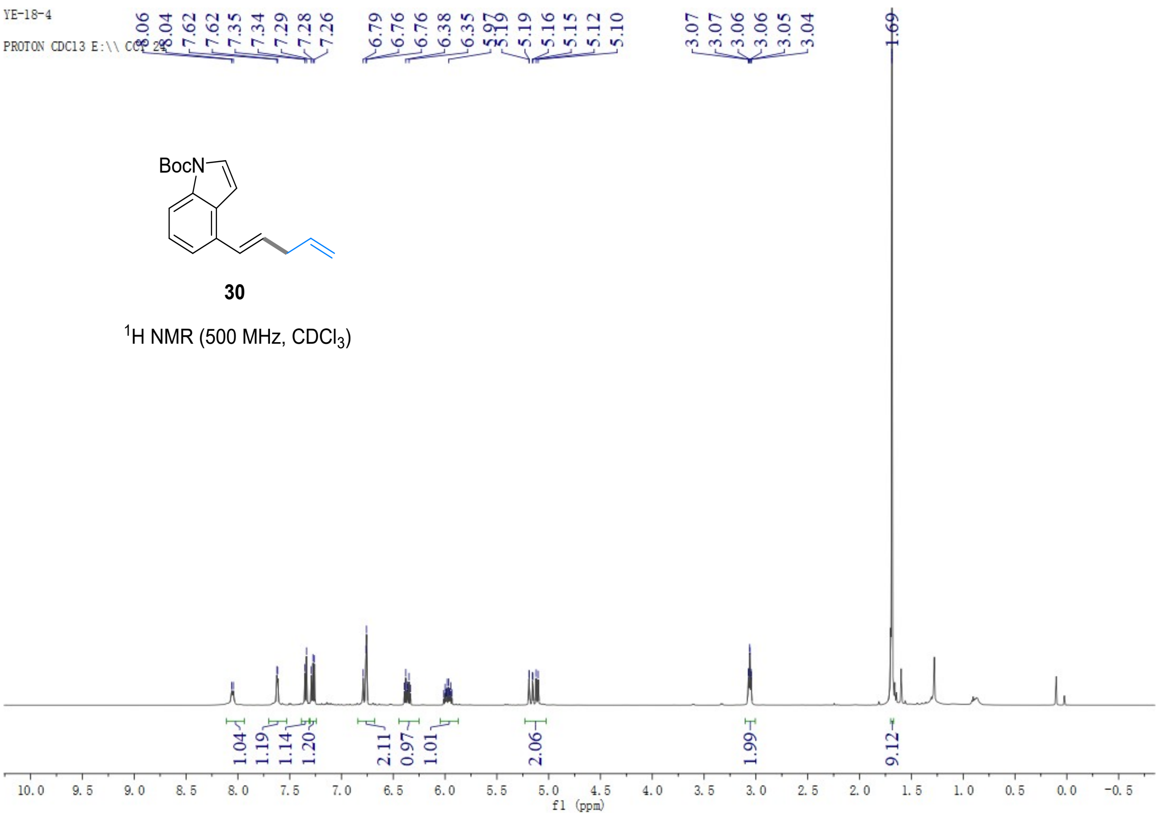
YE-18-4
PROTON CDCl3 E:\\ CCY 24
8.06 8.04 7.62 7.62 7.35 7.34 7.29 7.28 7.26 6.79 6.76 6.76 6.38 6.35 5.97 5.19 5.19 5.16 5.15 5.12 5.10 3.07 3.07 3.06 3.06 3.05 3.04 1.69
BocN
30
^{1}H NMR (500 MHz, CDCl$_3$)
1.04 1.19 1.14 1.20 2.11 0.97 1.01 2.06 1.99 9.12
10.0 9.5 9.0 8.5 8.0 7.5 7.0 6.5 6.0 5.5 5.0 4.5 4.0 3.5 3.0 2.5 2.0 1.5 1.0 0.5 0.0 -0.5
f1 (ppm)

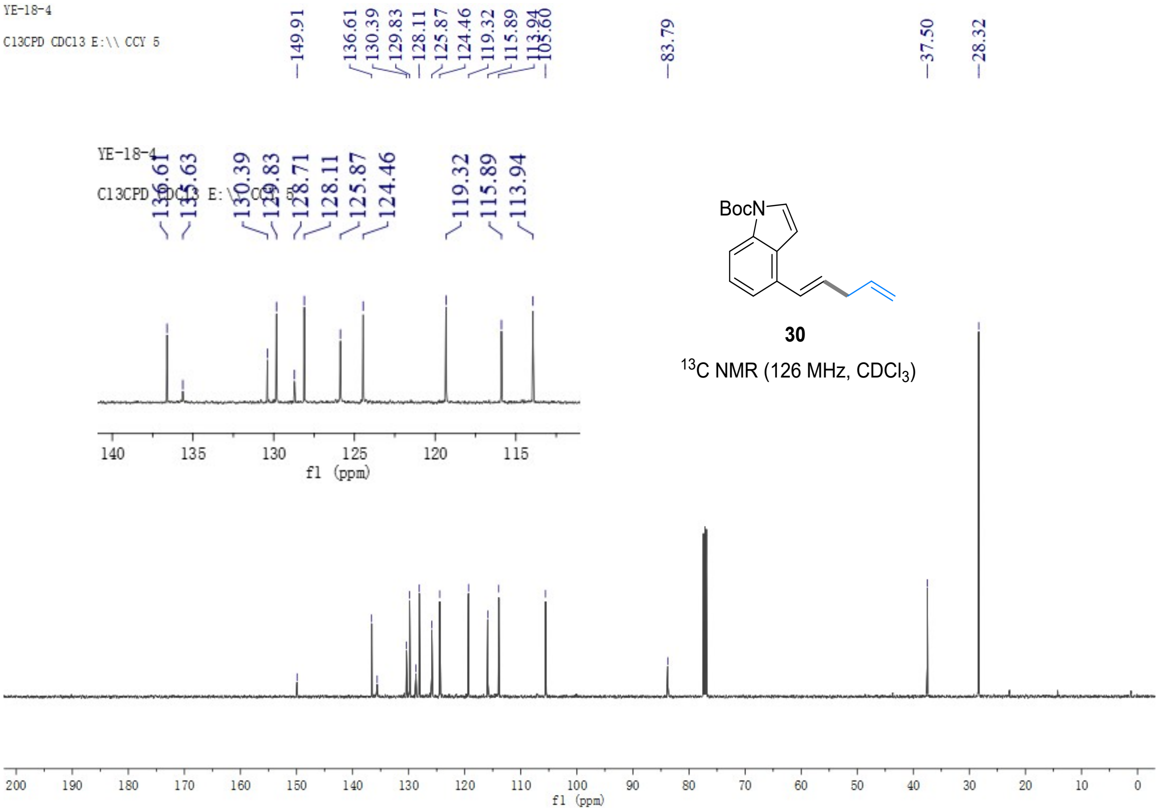
YE-18-4
C13CPD CDCl3 E:\\ CCY 5
149.91 136.61 130.39 129.83 128.11 125.87 124.46 119.32 115.89 113.94 105.60 83.79 37.50 28.32
130.39 128.71 128.11 125.87 124.46 119.32 115.89 113.94
140 135 130 125 120 115
f1 (ppm)
BocN
30
^{13}C NMR (126 MHz, CDCl$_3$)
200 190 180 170 160 150 140 130 120 110 100 90 80 70 60 50 40 30 20 10 0
f1 (ppm)

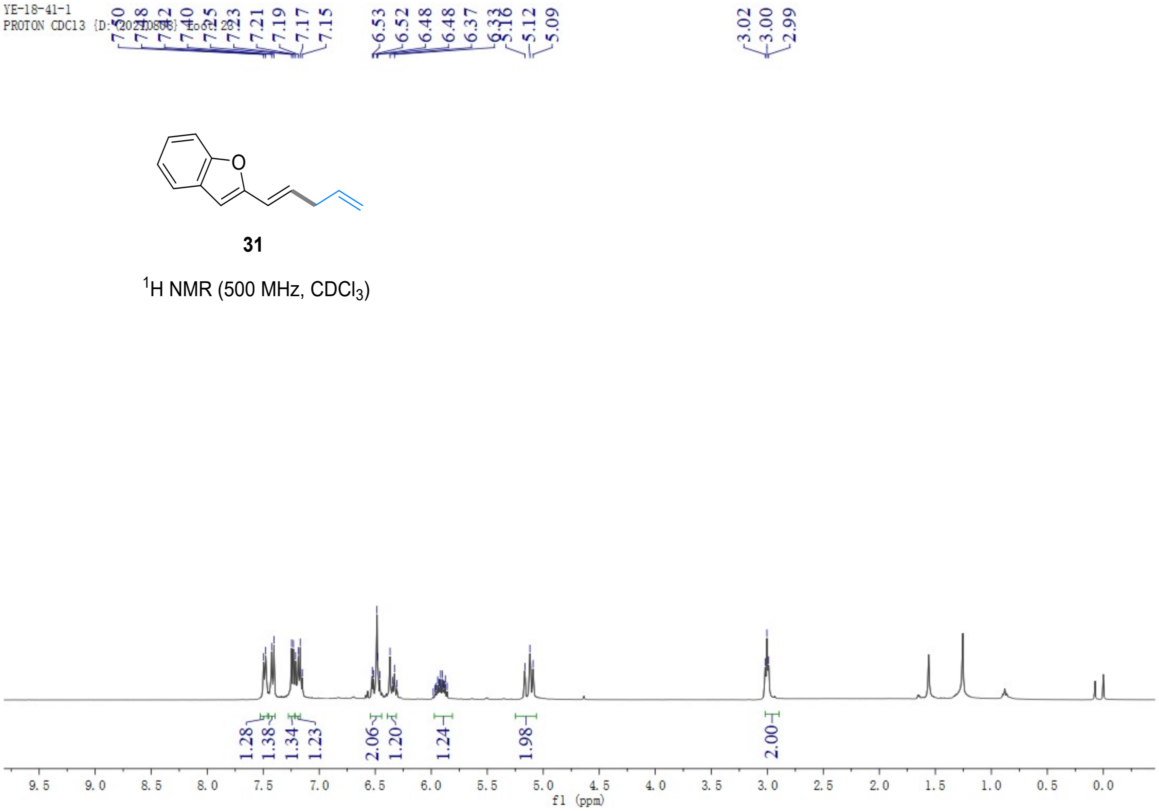
YE-18-41-1
7.21
7.19
7.17
7.15
6.53
6.52
6.48
6.48
6.37
5.12
5.09
3.02
3.00
2.99
31
1H NMR (500 MHz, CDCl3)
1.28
1.38
1.34
1.23
2.06
1.20
1.24
1.98
2.00
f1 (ppm)

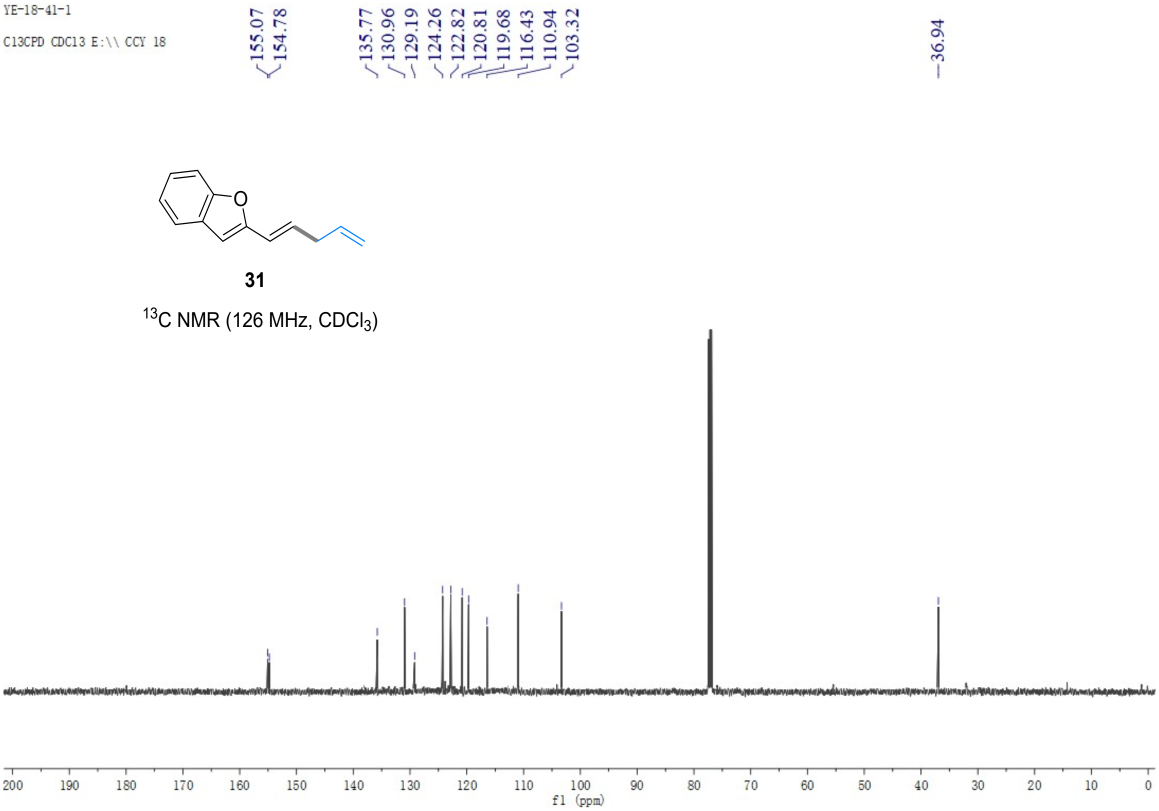
YE-18-41-1
C13CPD CDCl3 E:\\ CCY 18
155.07
154.78
135.77
130.96
129.19
124.26
122.82
120.81
119.68
116.43
110.94
103.32
36.94
31
13C NMR (126 MHz, CDCl3)
f1 (ppm)

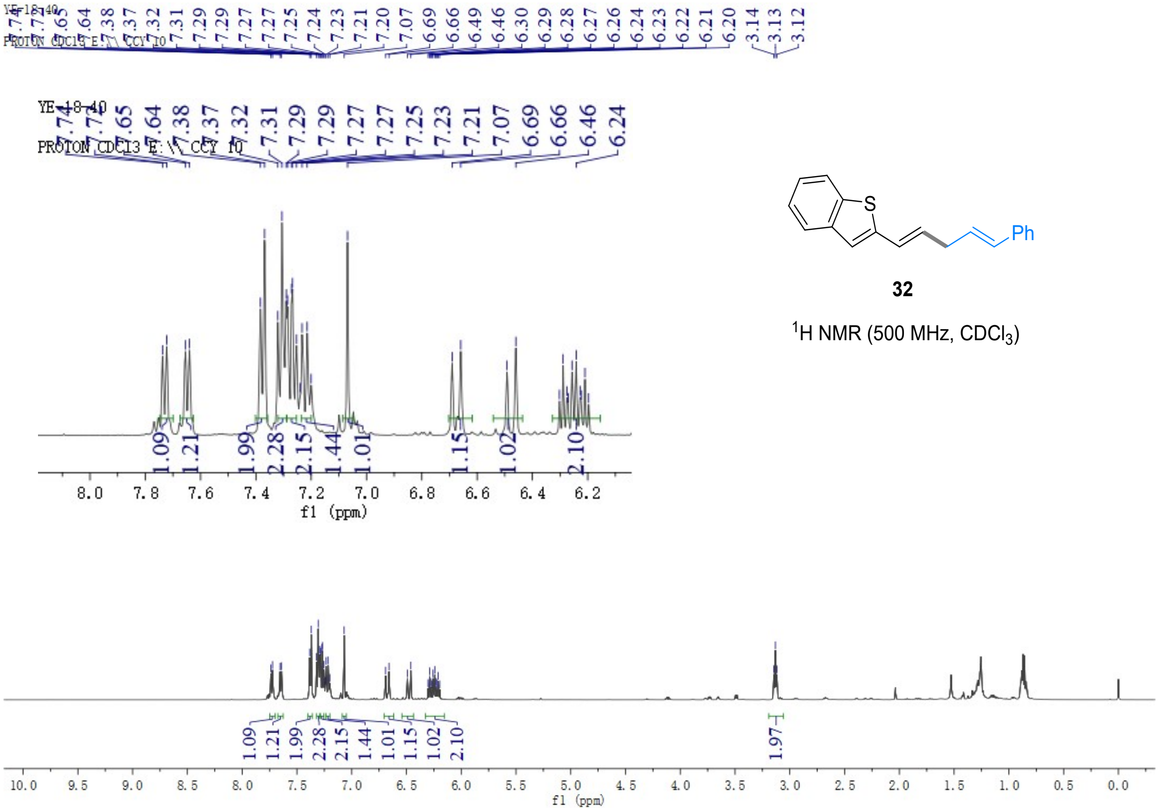

32

^{1}H NMR (500 MHz, $CDCl_3$)

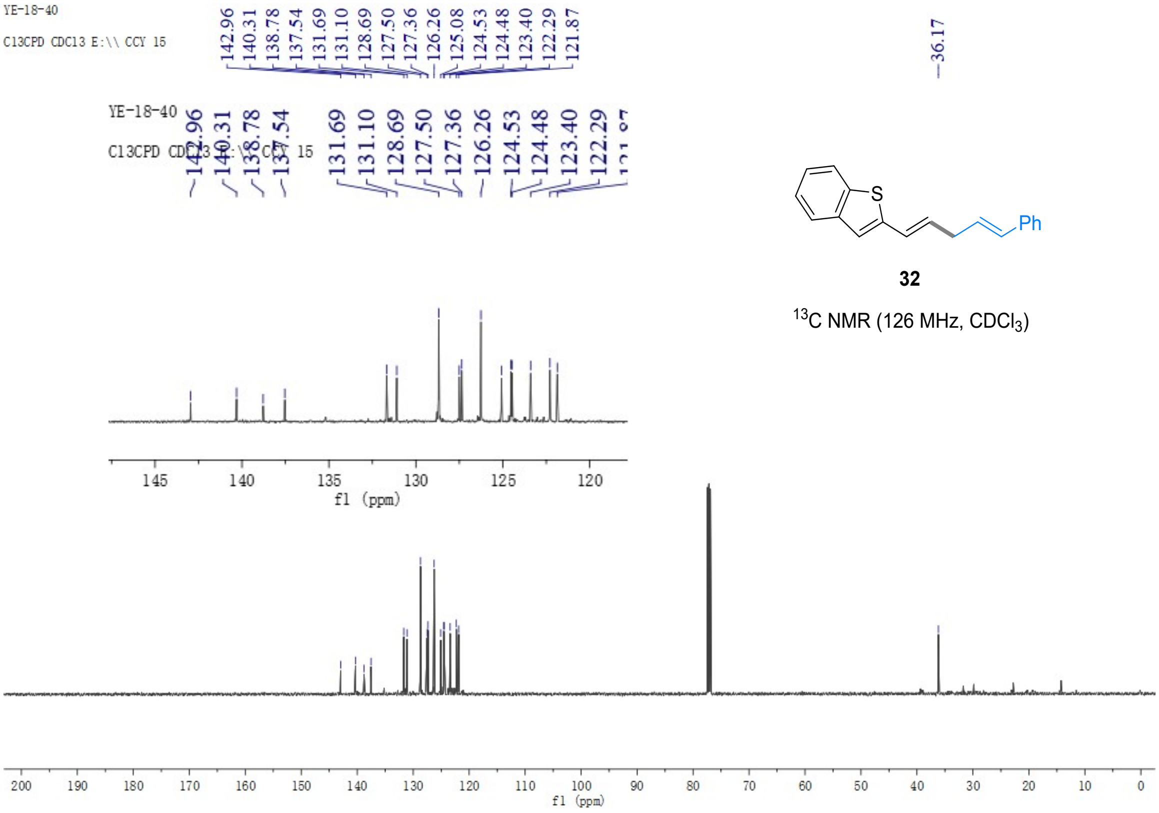

32

^{13}C NMR (126 MHz, $CDCl_3$)

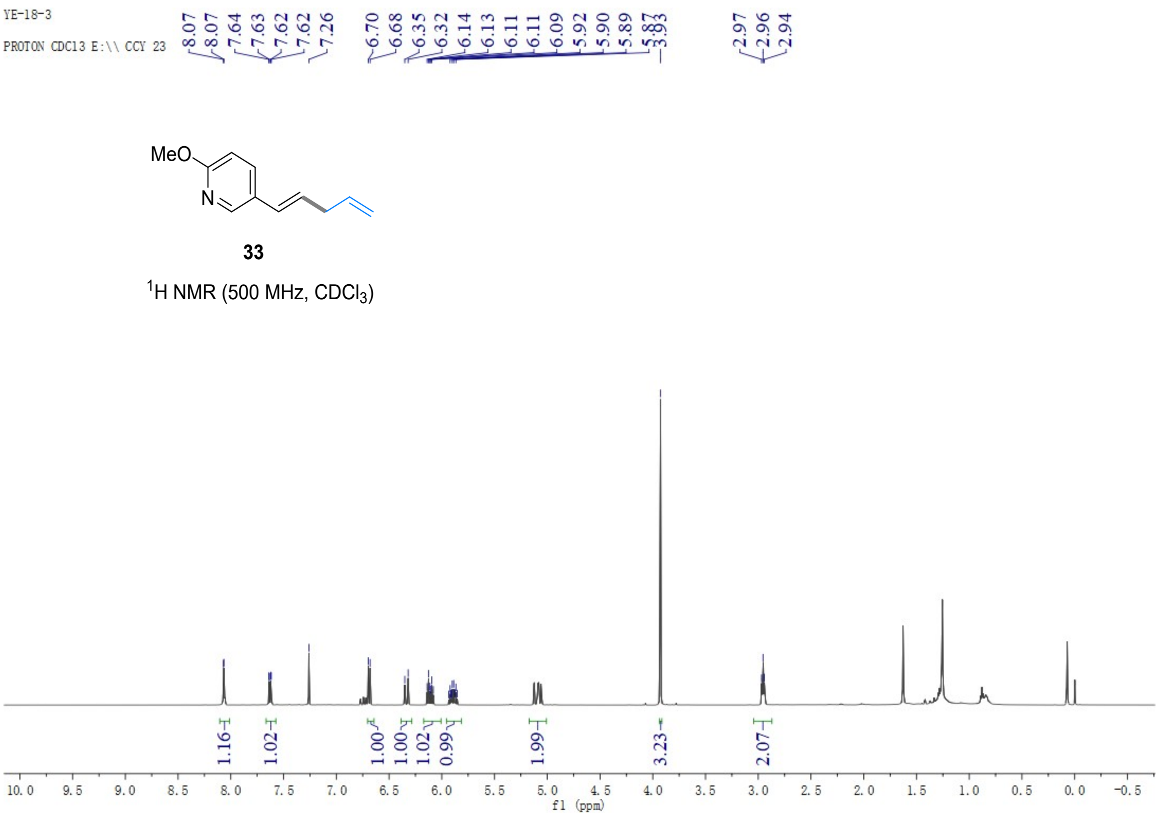

33

^{1}H NMR (500 MHz, $CDCl_3$)

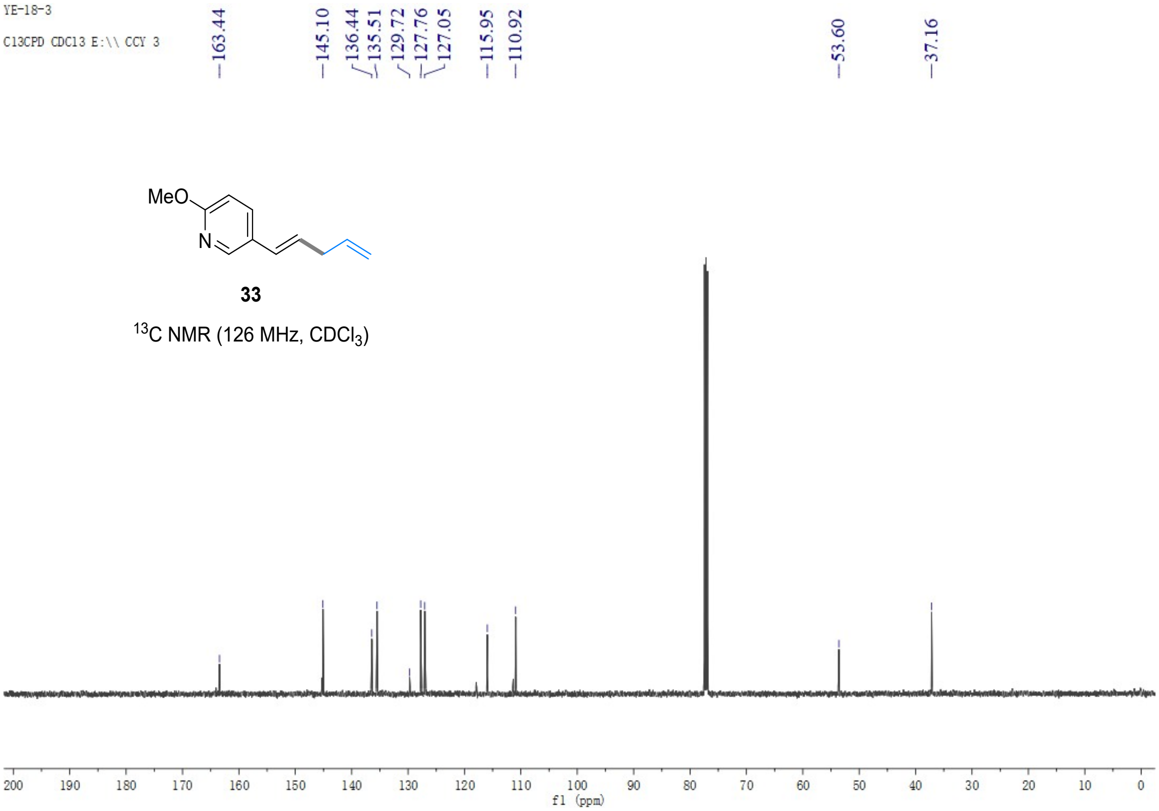

33

^{13}C NMR (126 MHz, $CDCl_3$)

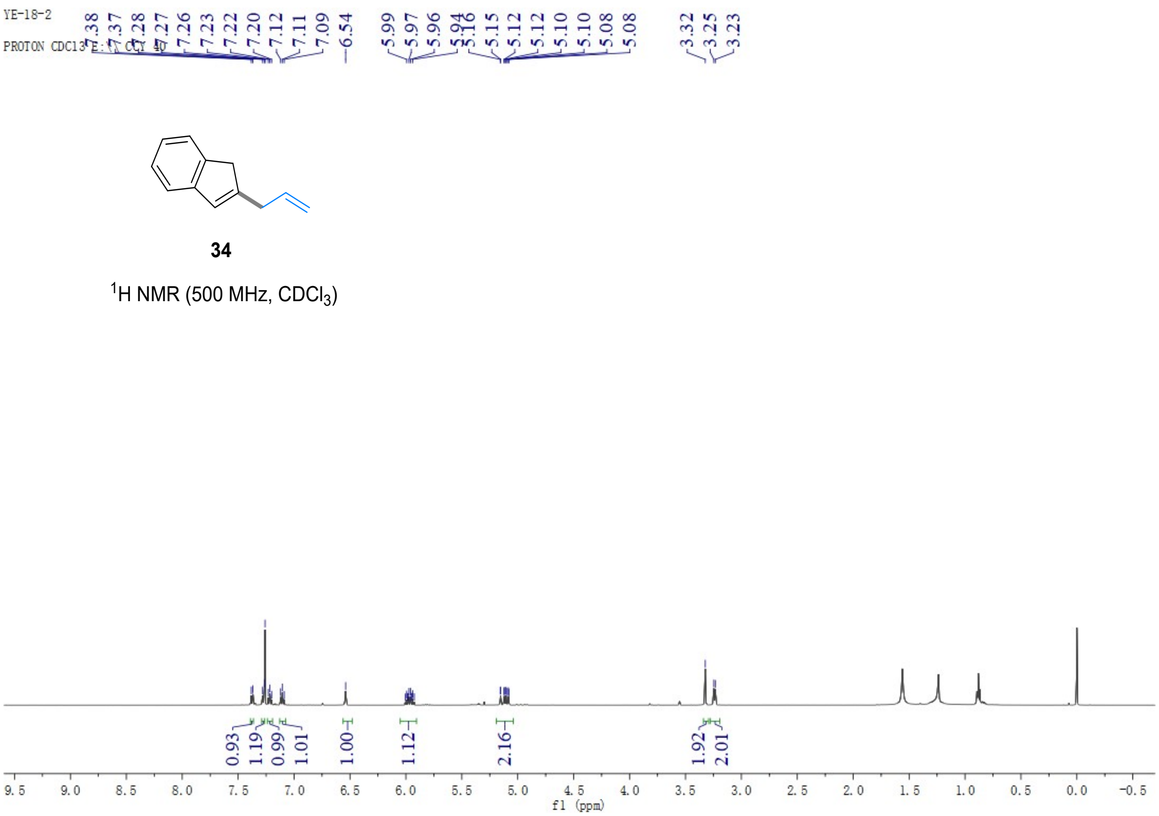

34

^{1}H NMR (500 MHz, $CDCl_3$)

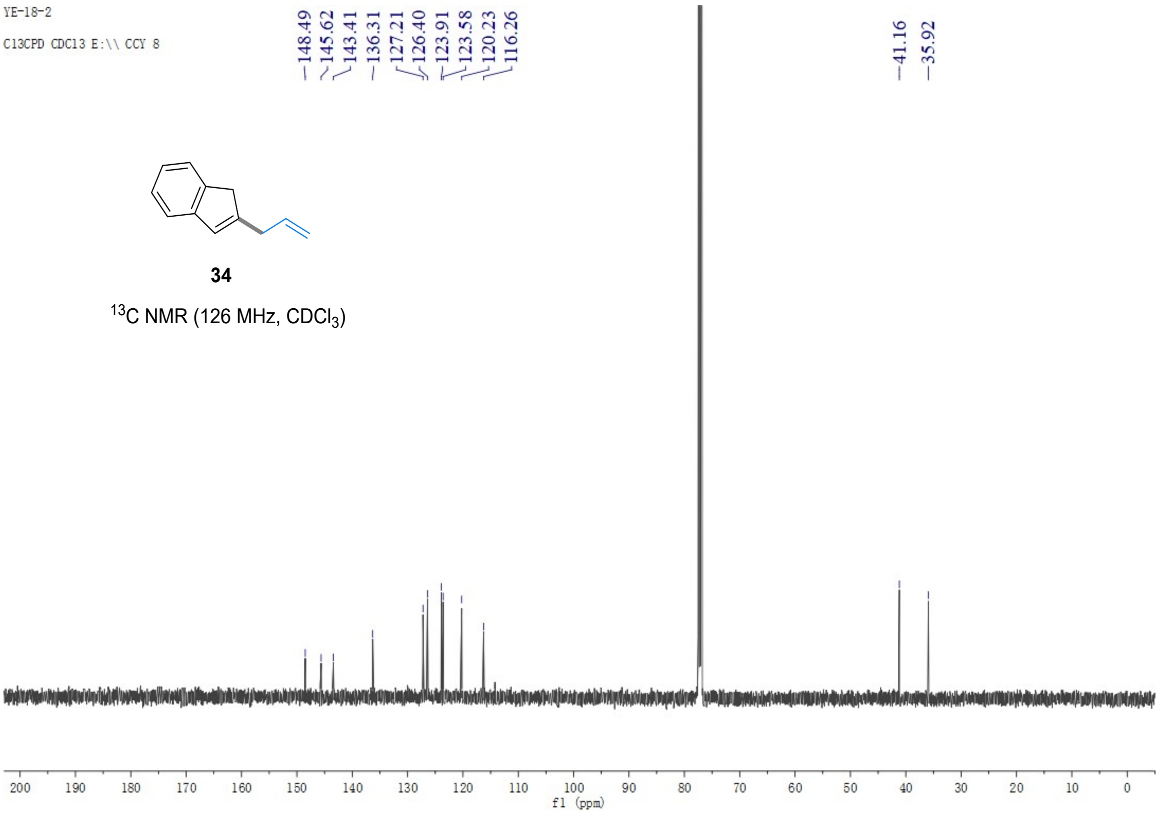

34

^{13}C NMR (126 MHz, $CDCl_3$)

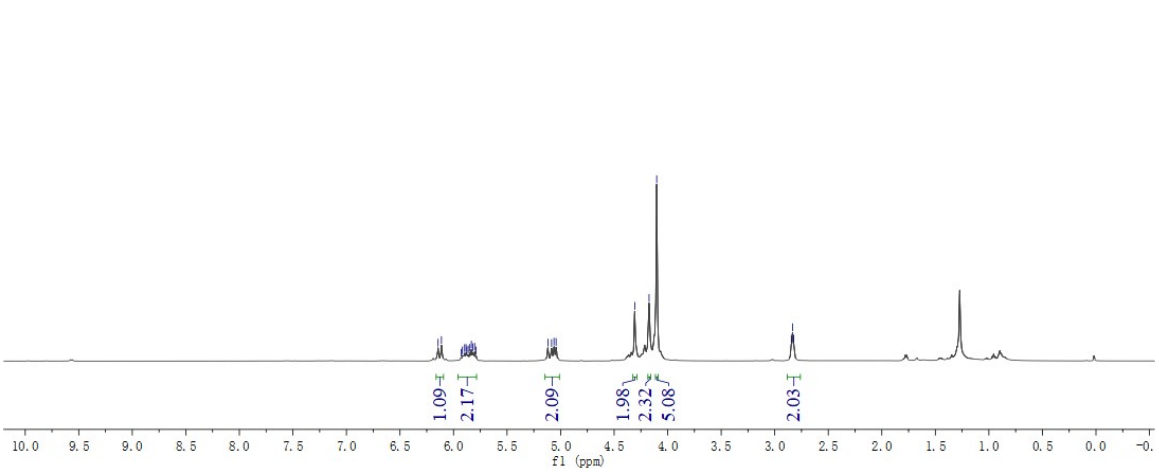

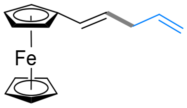

35

^{1}H NMR (500 MHz, $CDCl_3$)

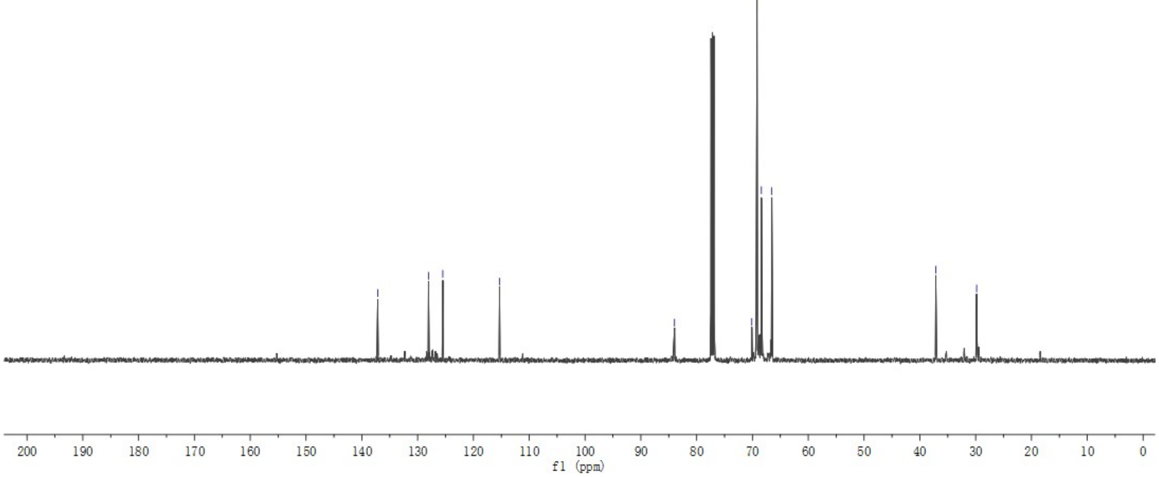

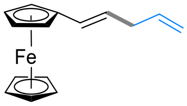

35

^{13}C NMR (126 MHz, $CDCl_3$)

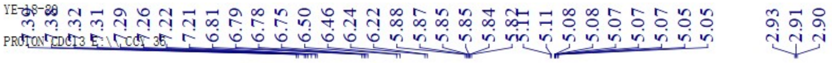

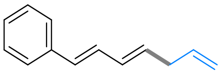

36

^{1}H NMR (500 MHz, $CDCl_3$)

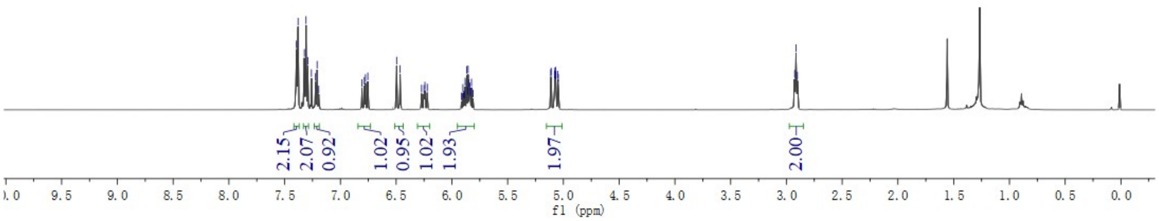

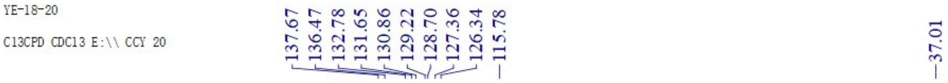

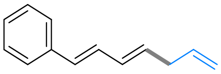

36

^{13}C NMR (126 MHz, $CDCl_3$)

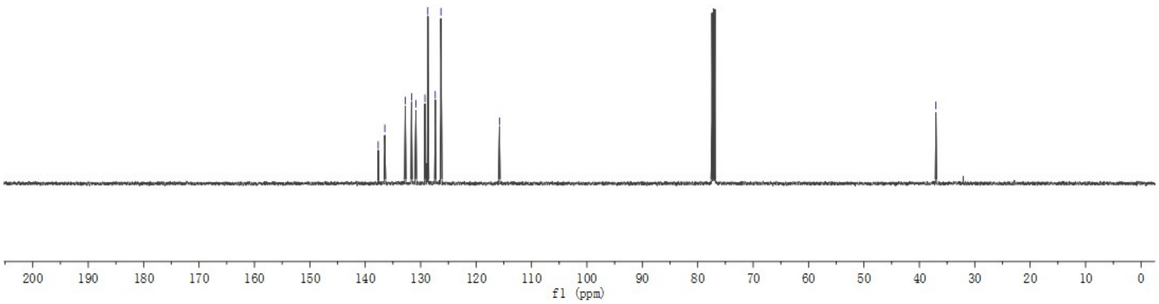

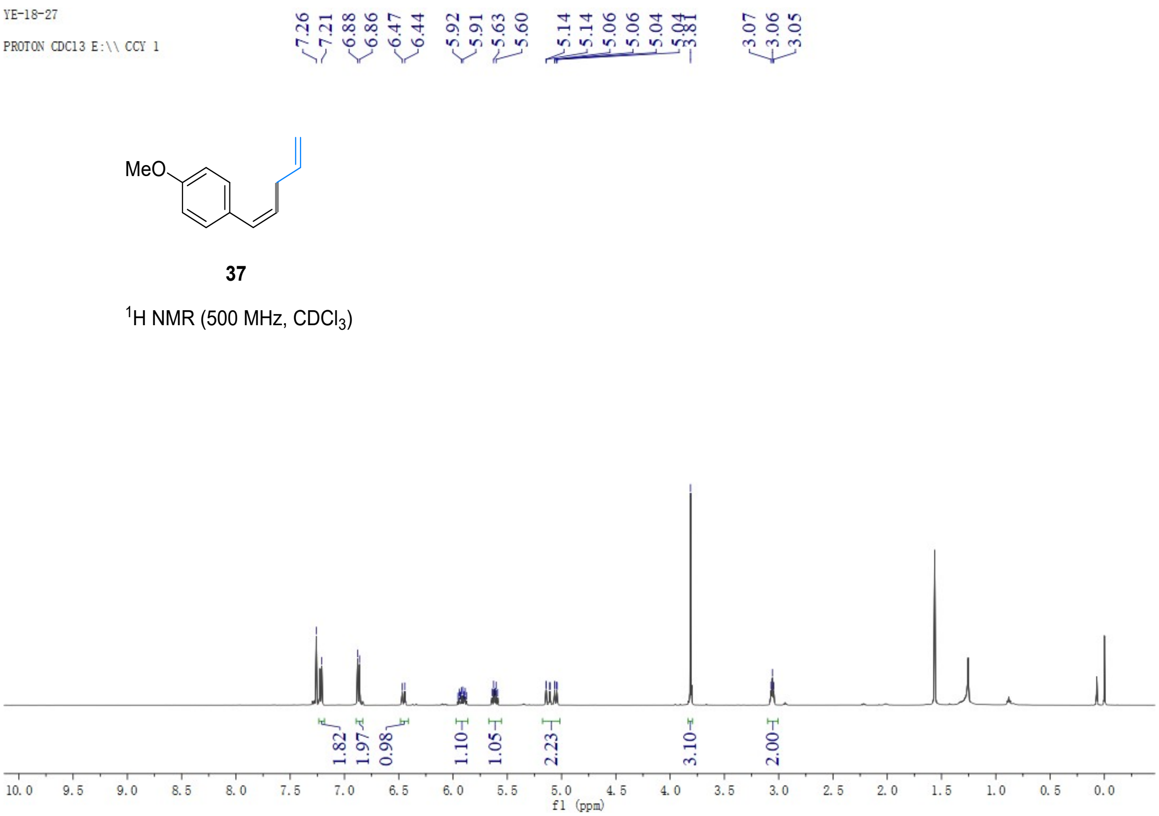

37

^{1}H NMR (500 MHz, $CDCl_3$)

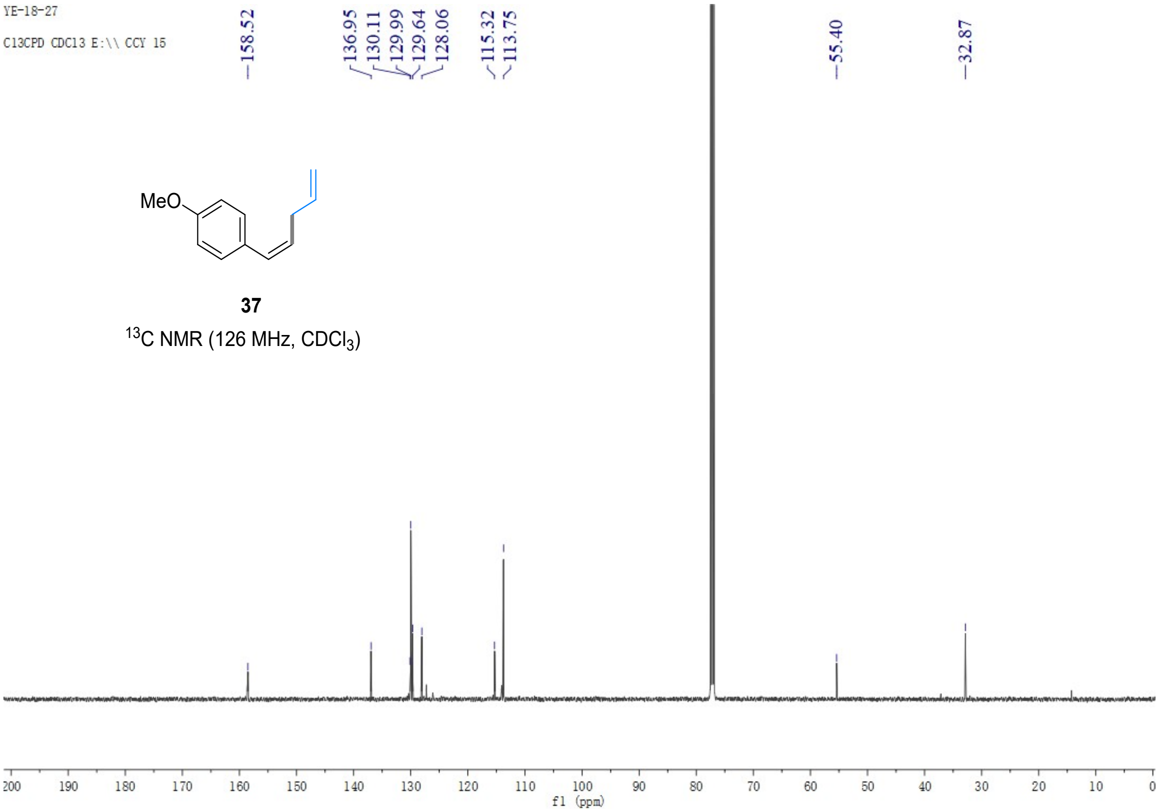

37

^{13}C NMR (126 MHz, $CDCl_3$)

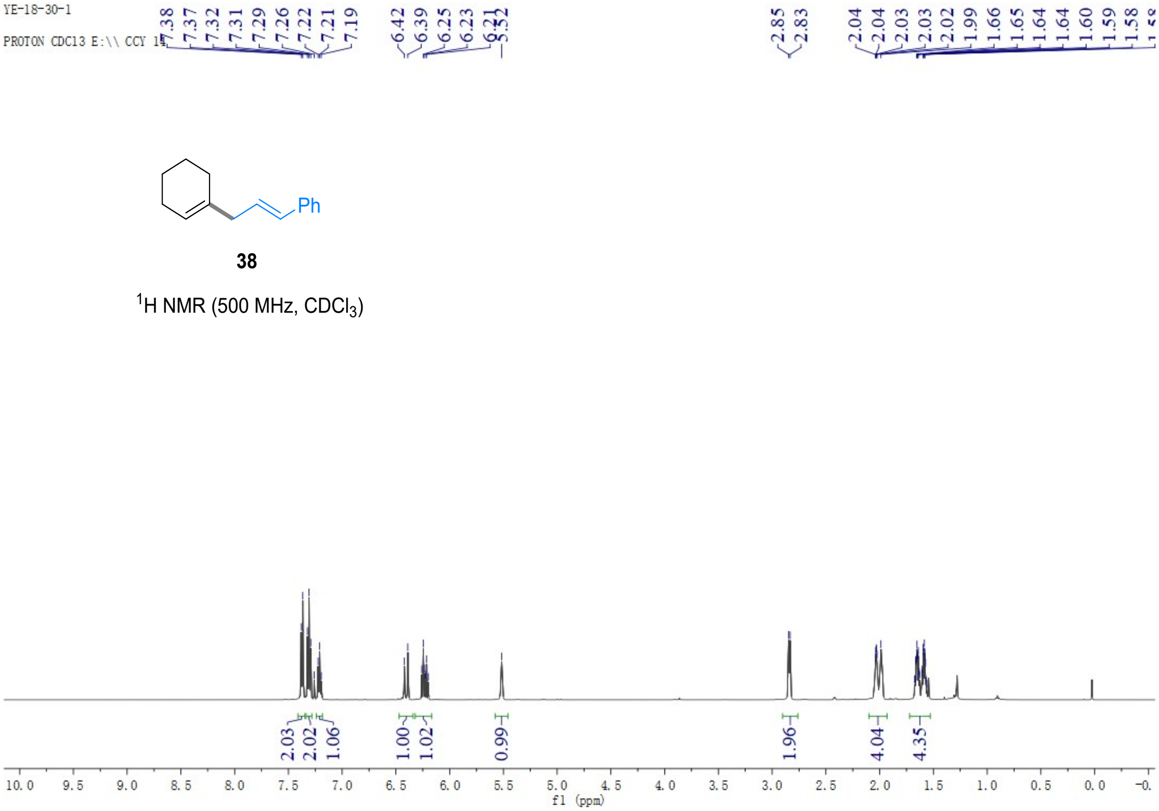
YE-18-30-1
PROTON CDCl3 E:\\ CCY 14
Ph
38
1H NMR (500 MHz, CDCl3)
f1 (ppm)

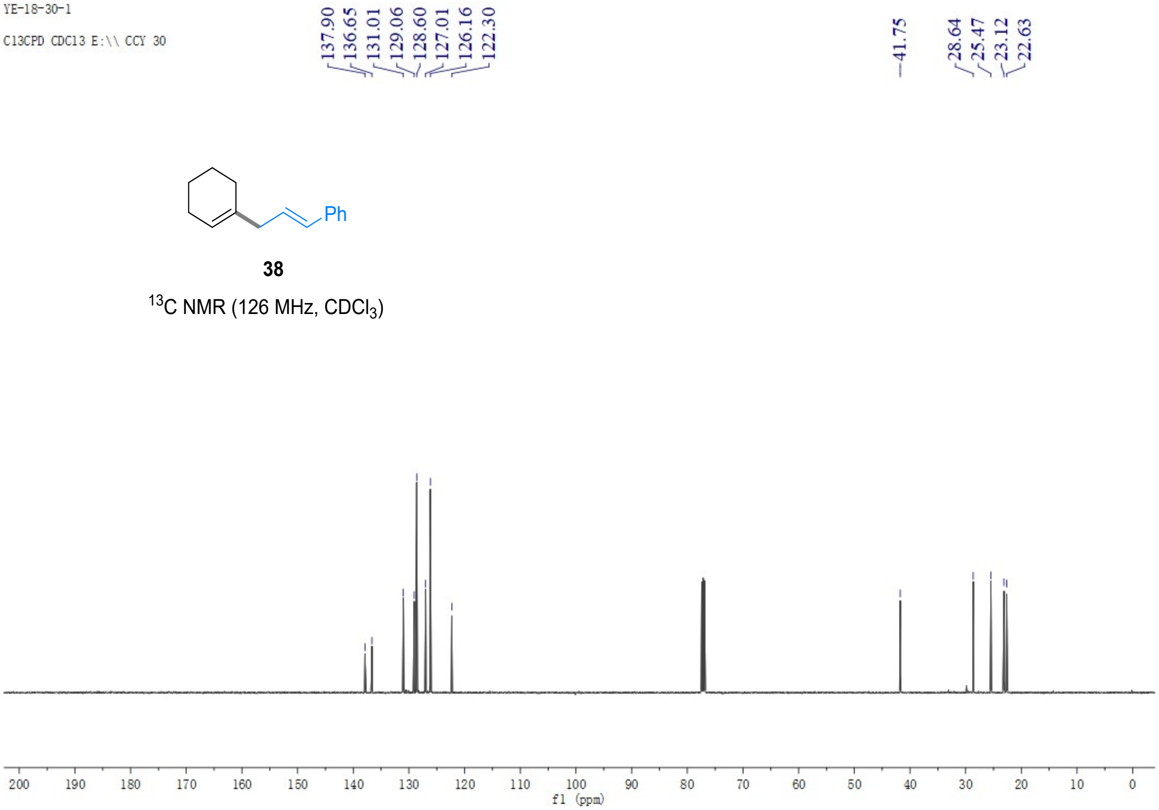
YE-18-30-1
C13CPD CDCl3 E:\\ CCY 30
Ph
38
13C NMR (126 MHz, CDCl3)
f1 (ppm)

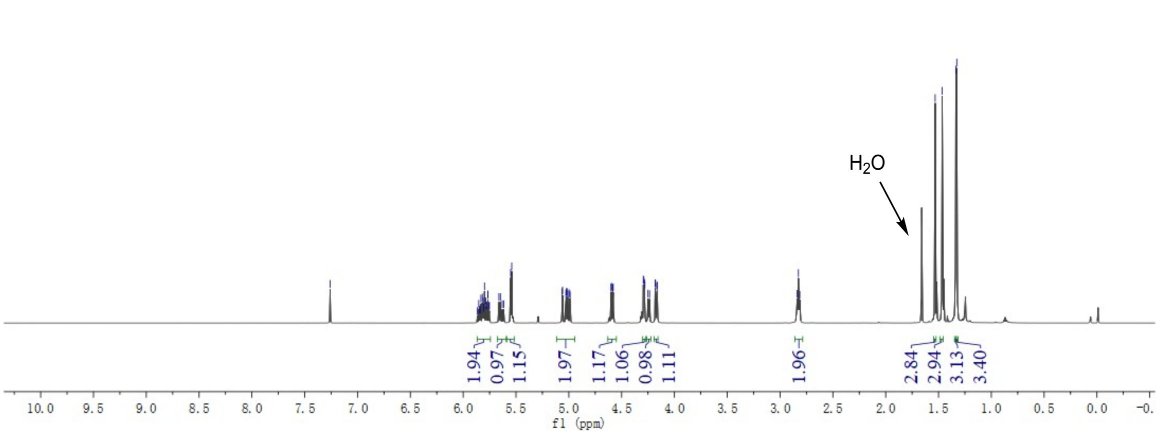

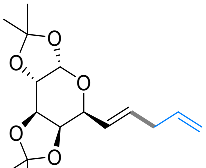

39

^1H NMR (500 MHz, $CDCl_3$)

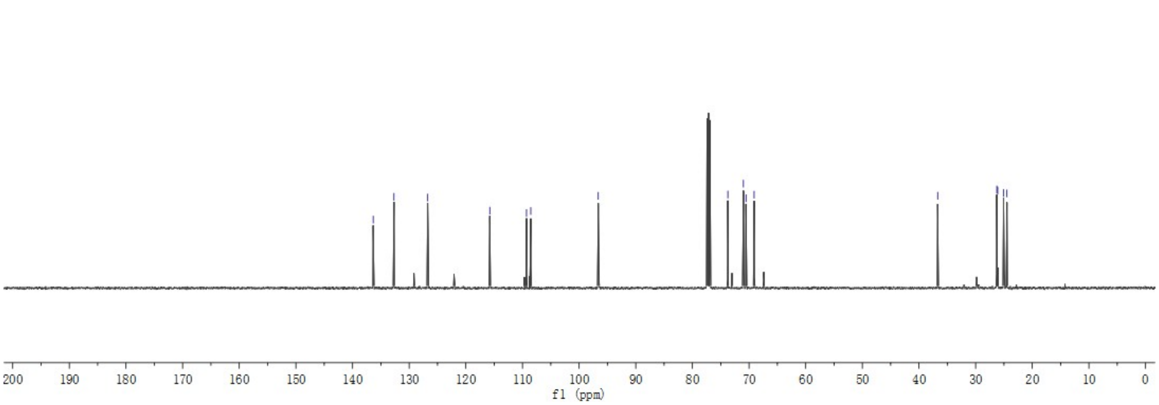

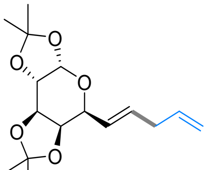

39

^{13}C NMR (126 MHz, $CDCl_3$)

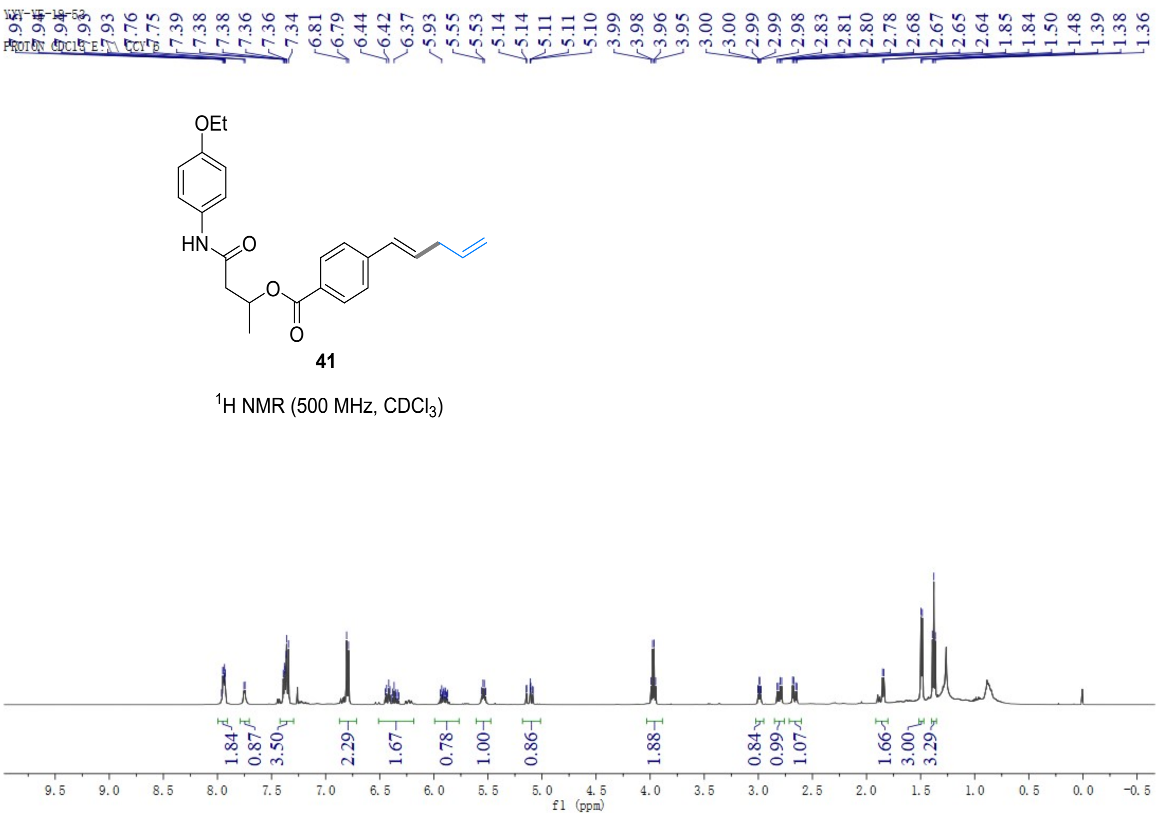
OEt
HN
O
O
O
41
^{1}H NMR (500 MHz, $CDCl_3$)

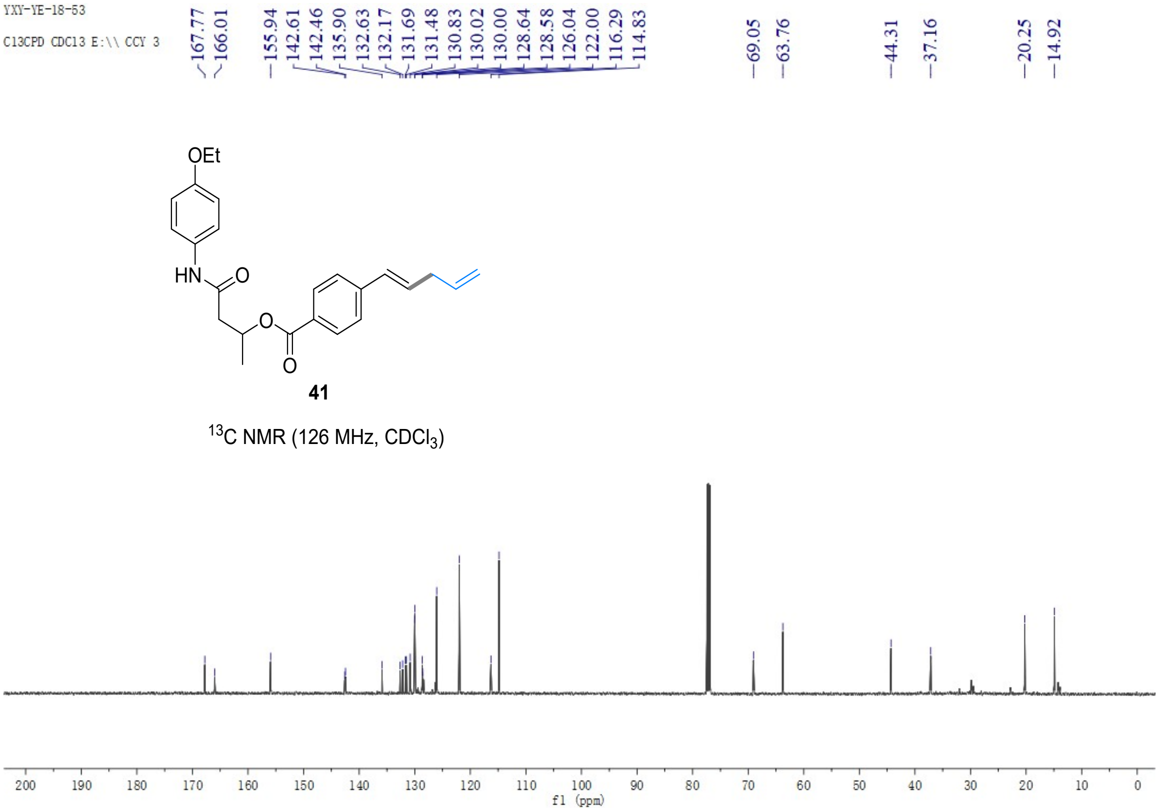
OEt
HN
O
O
O
41
^{13}C NMR (126 MHz, $CDCl_3$)

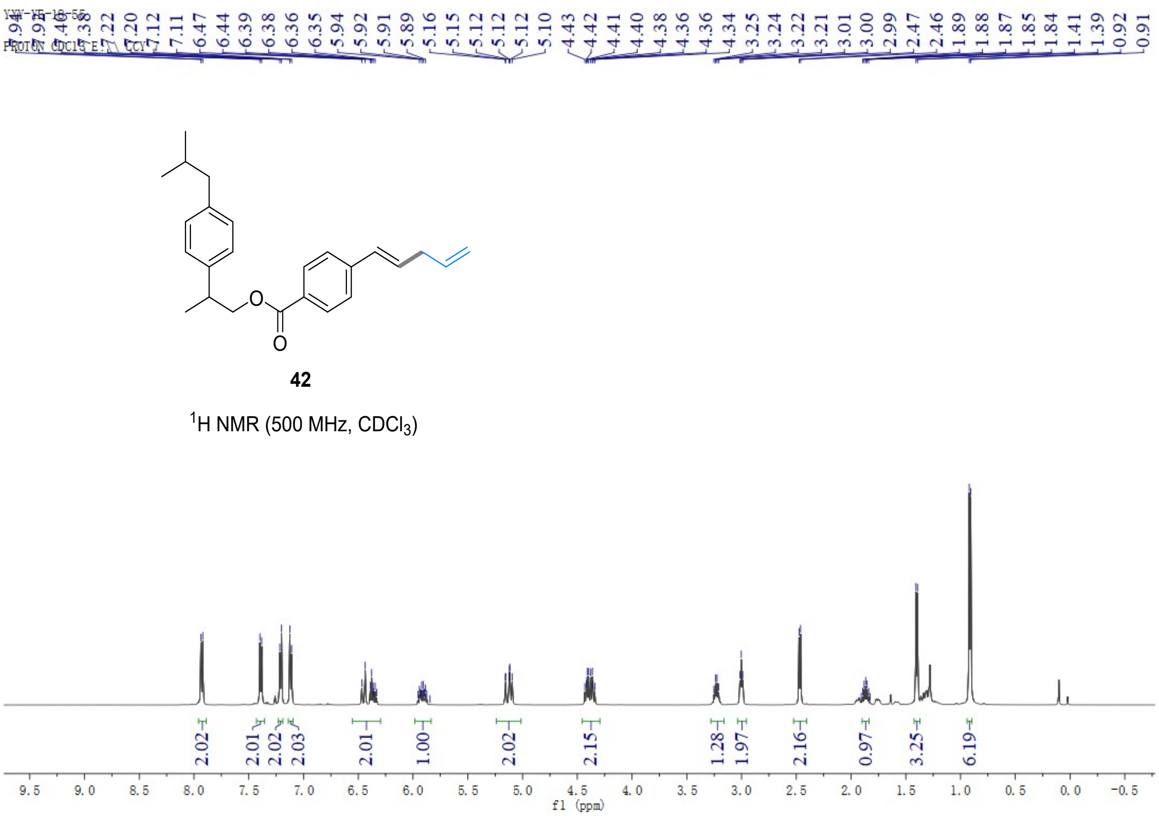

42

^{1}H NMR (500 MHz, $CDCl_3$)

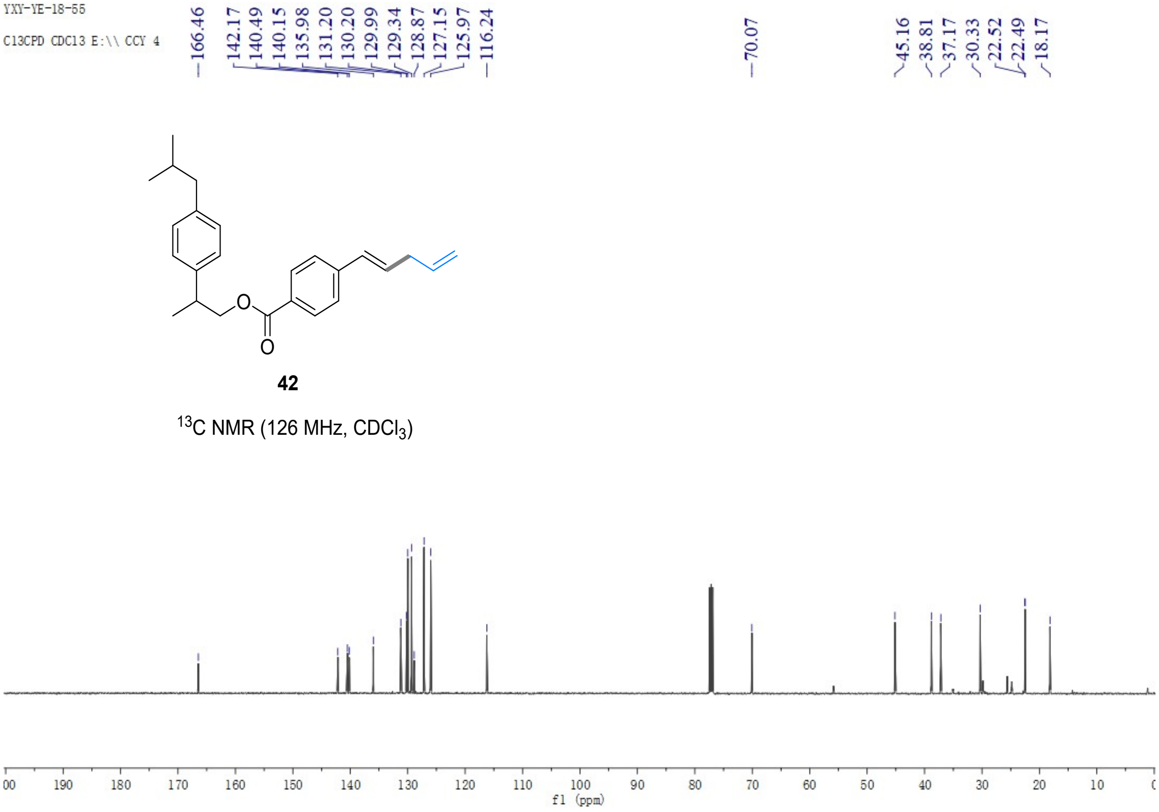

42

^{13}C NMR (126 MHz, $CDCl_3$)

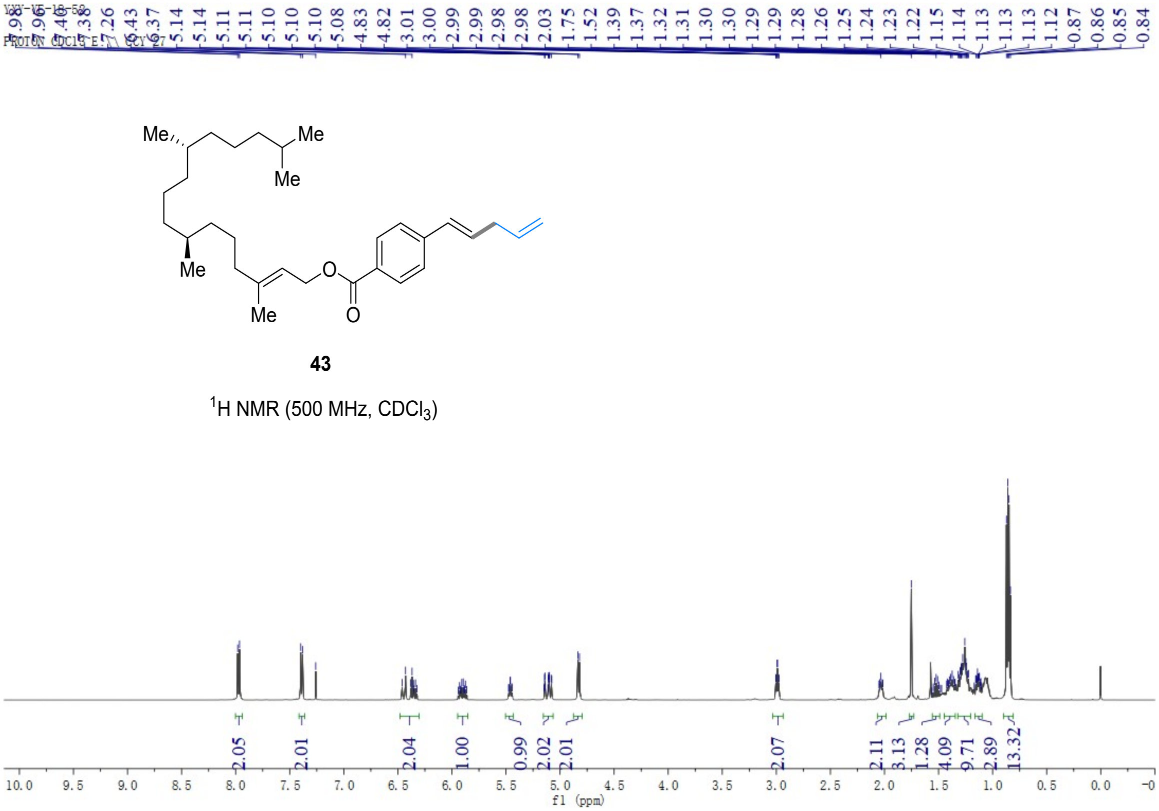

43

^{1}H NMR (500 MHz, $CDCl_3$)

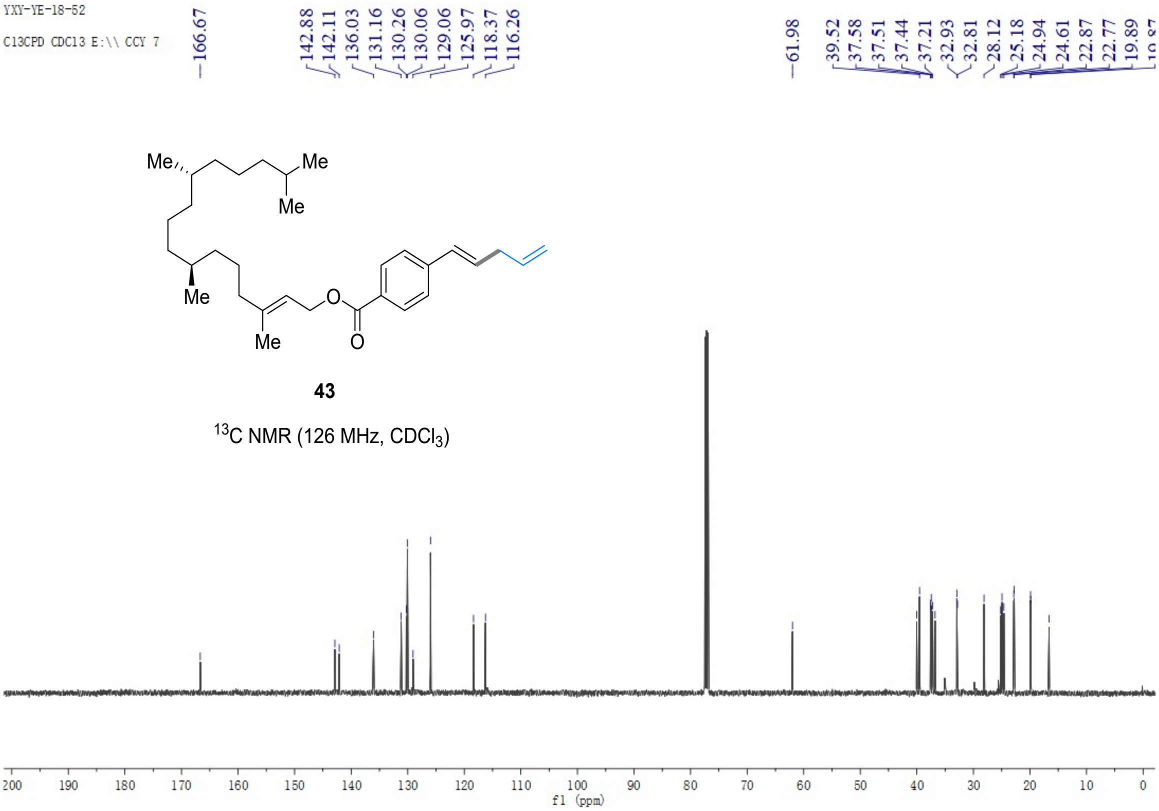

43

^{13}C NMR (126 MHz, $CDCl_3$)

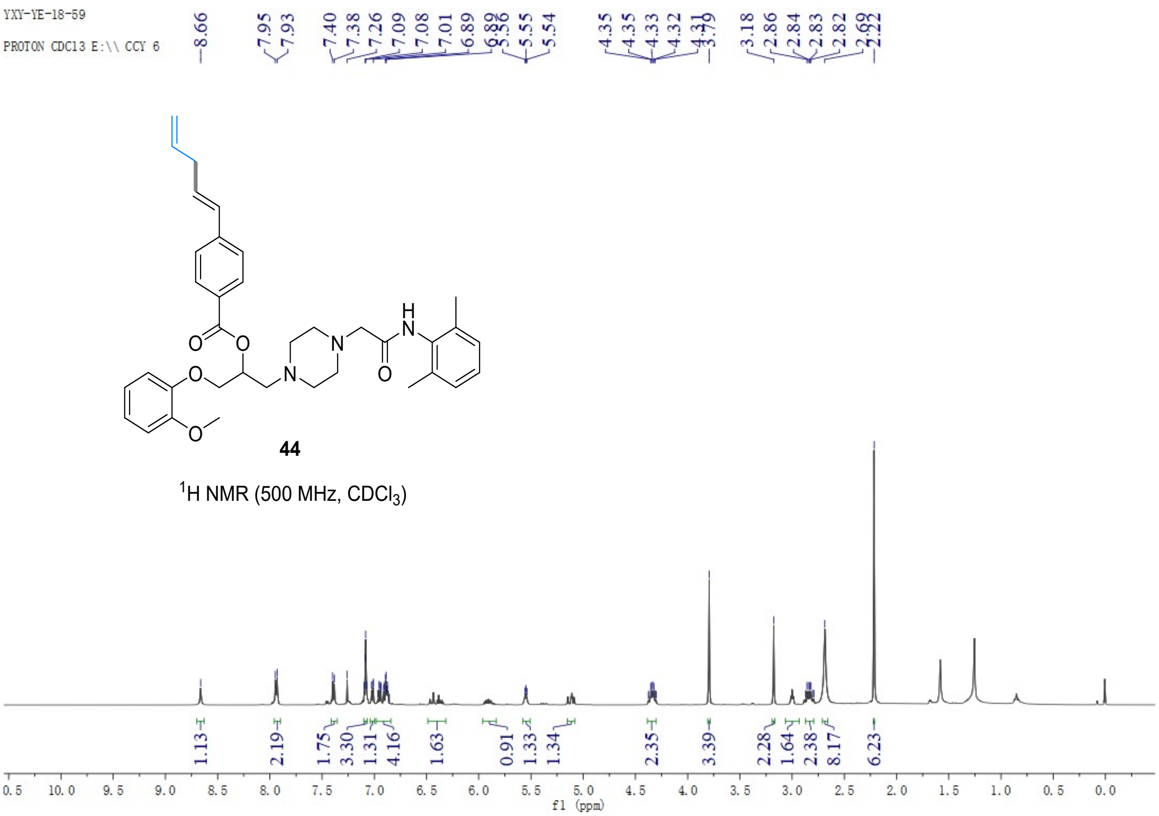

44

^{1}H NMR (500 MHz, $CDCl_3$)

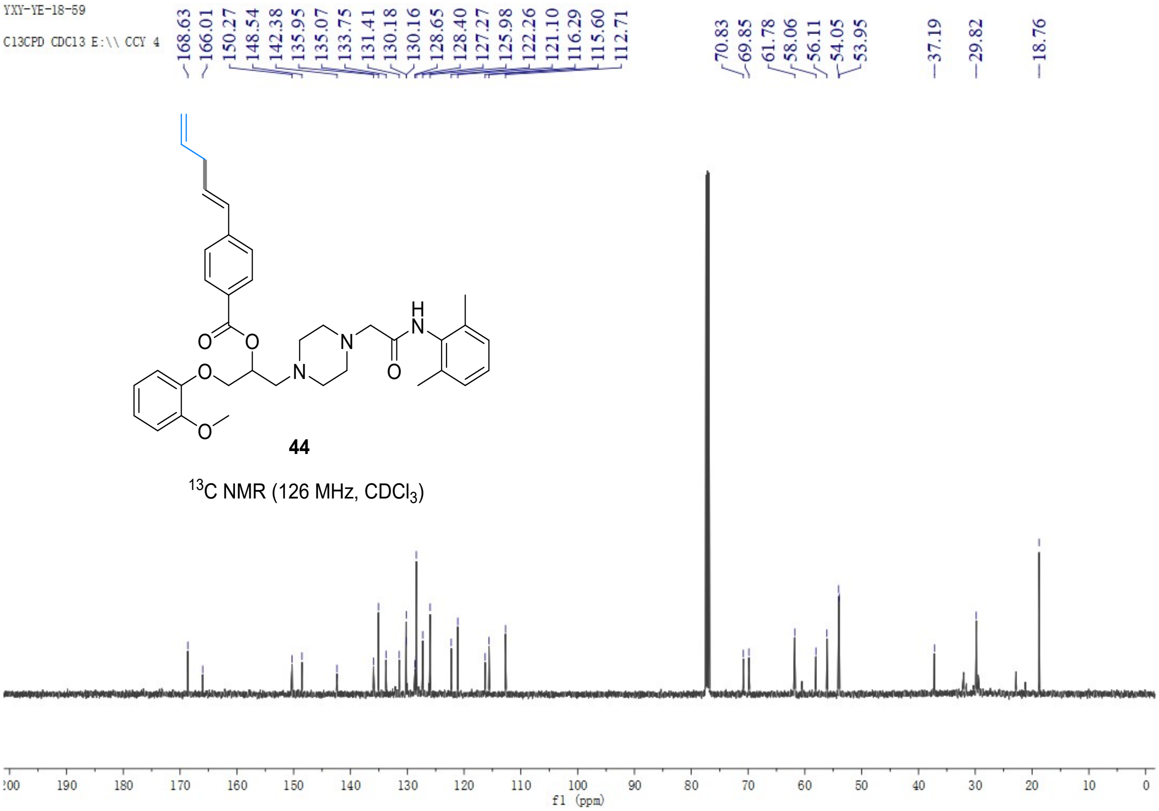

44

^{13}C NMR (126 MHz, $CDCl_3$)

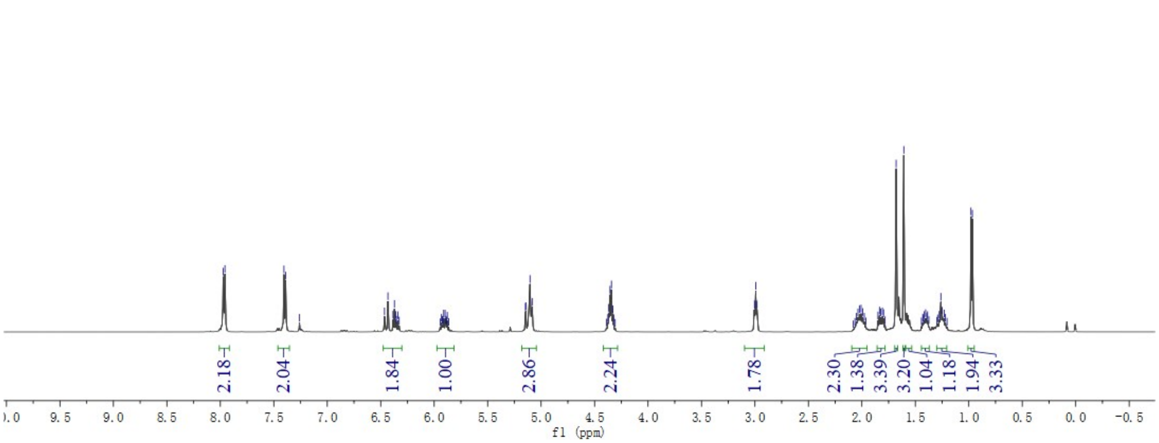

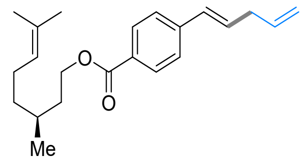

45

^{1}H NMR (500 MHz, $CDCl_3$)

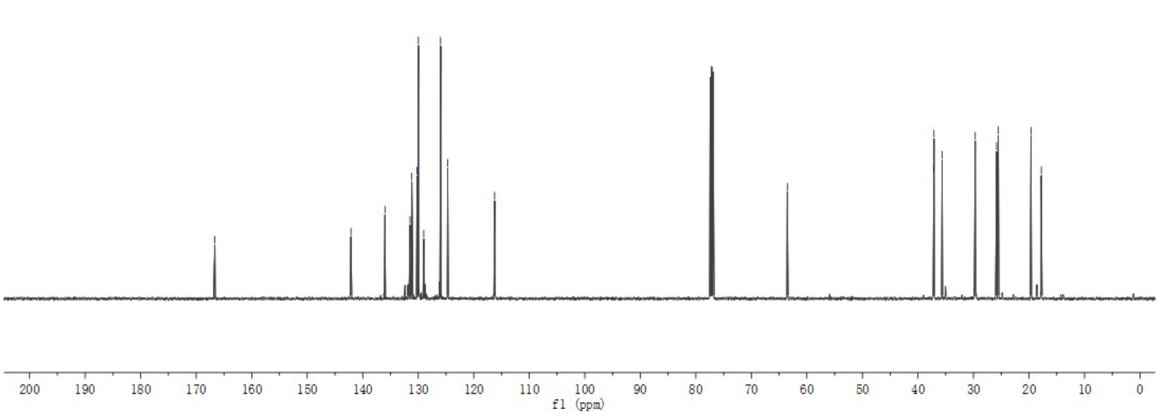

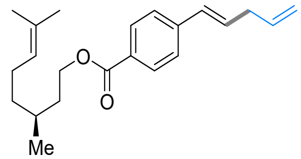

45

^{13}C NMR (126 MHz, $CDCl_3$)

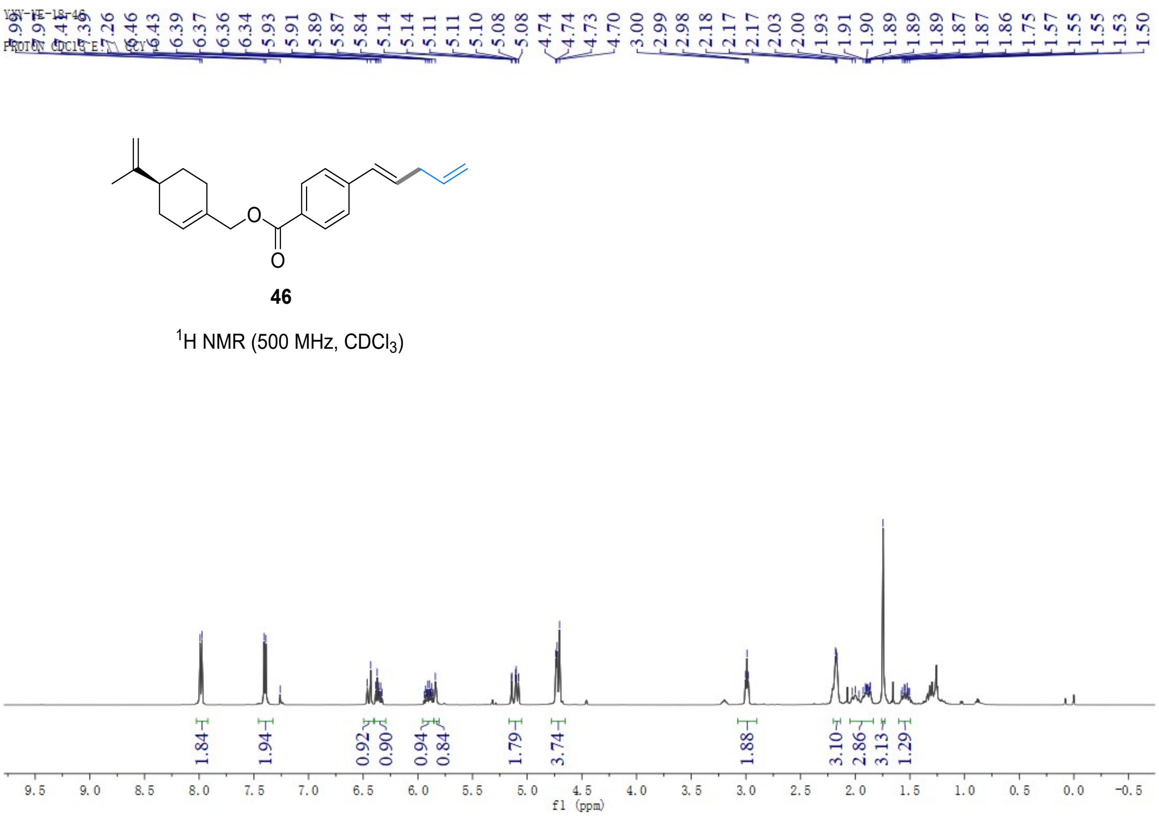

46

^{1}H NMR (500 MHz, $CDCl_3$)

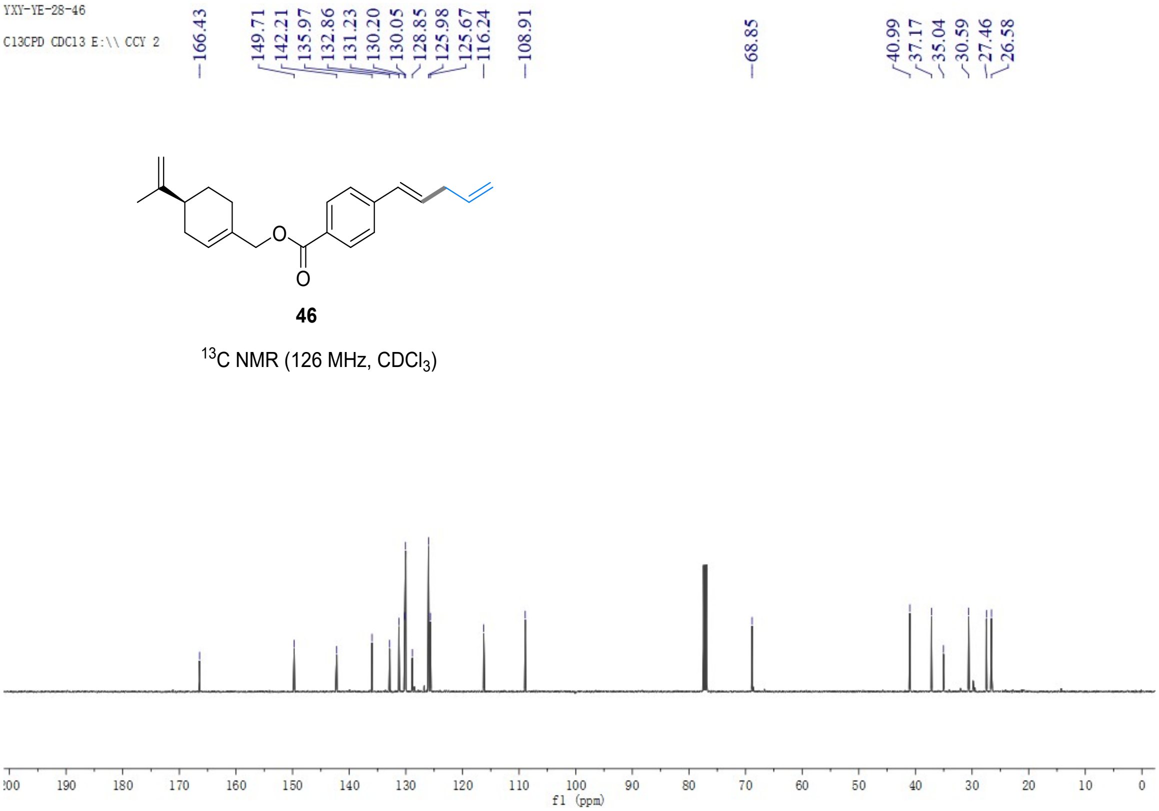

46

^{13}C NMR (126 MHz, $CDCl_3$)

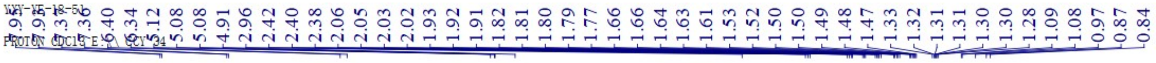

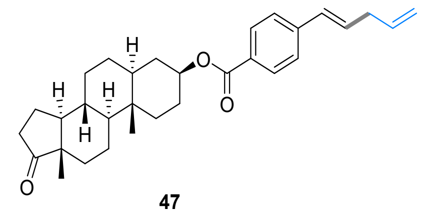

47

^{1}H NMR (500 MHz, $CDCl_3$)

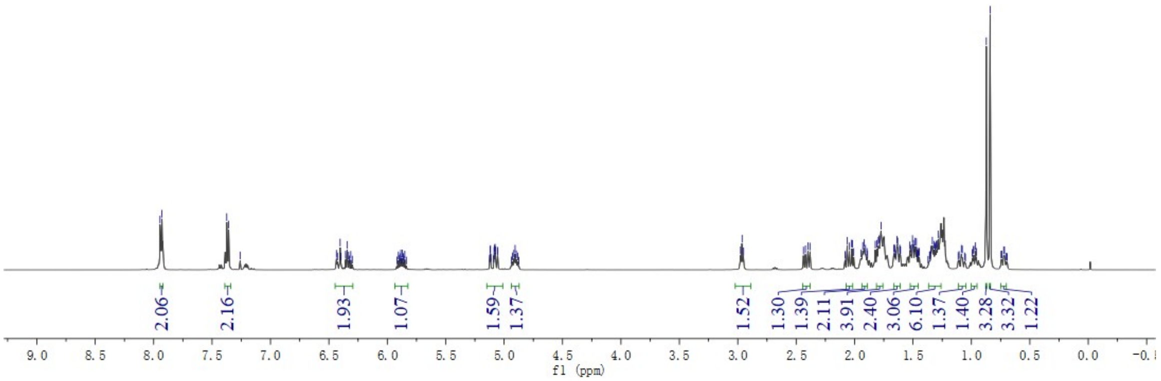

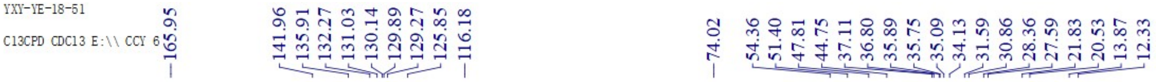

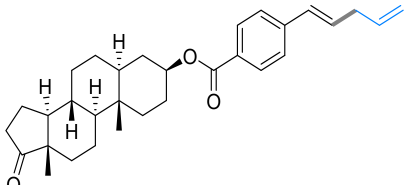

47

^{13}C NMR (126 MHz, $CDCl_3$)

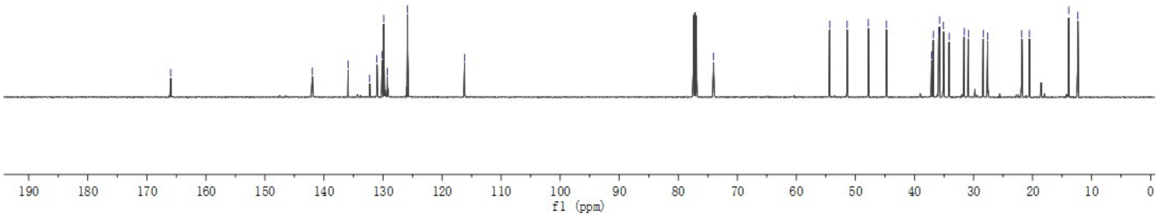

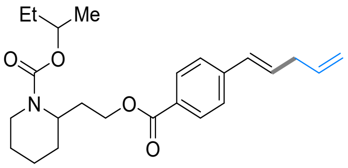

48

^{1}H NMR (500 MHz, $CDCl_3$)

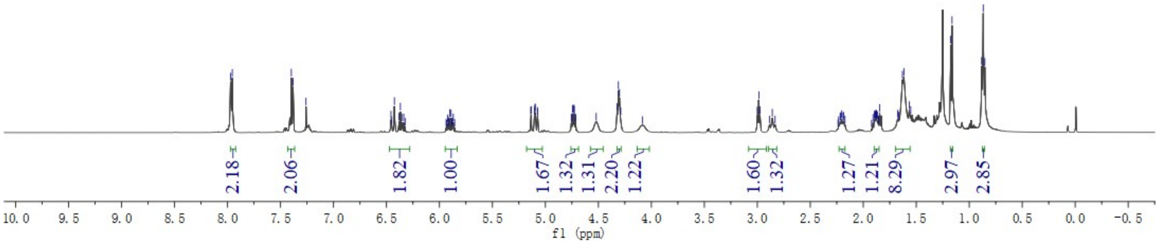

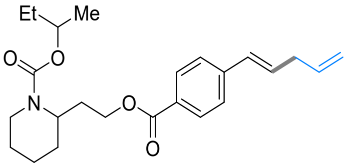

48

^{13}C NMR (126 MHz, $CDCl_3$)

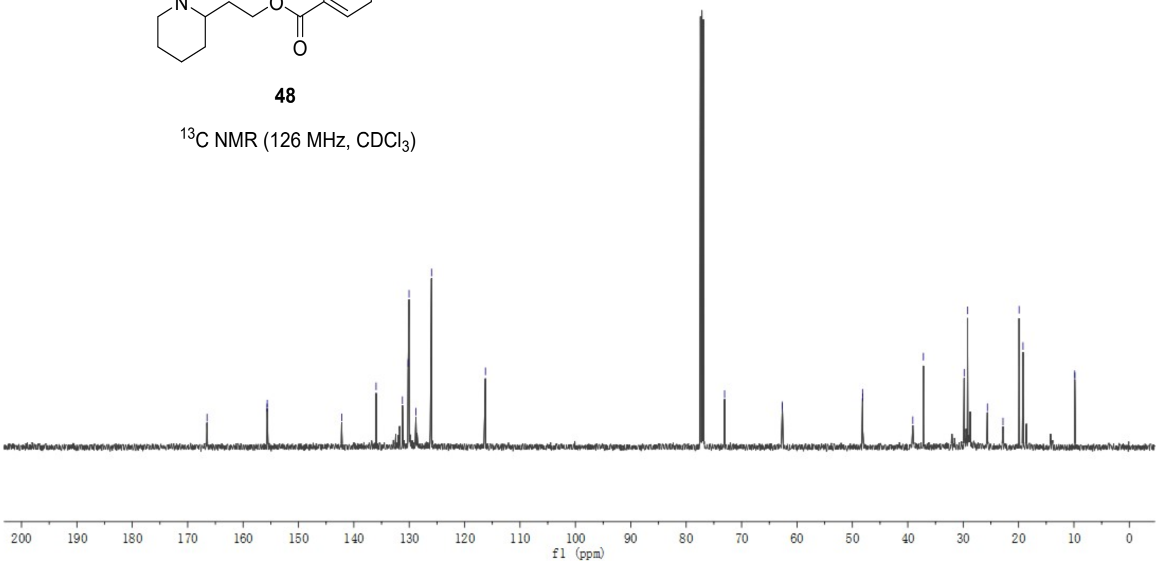

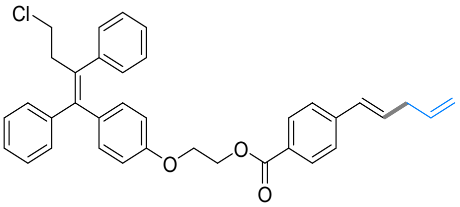

49

^{1}H NMR (500 MHz, $CDCl_3$)

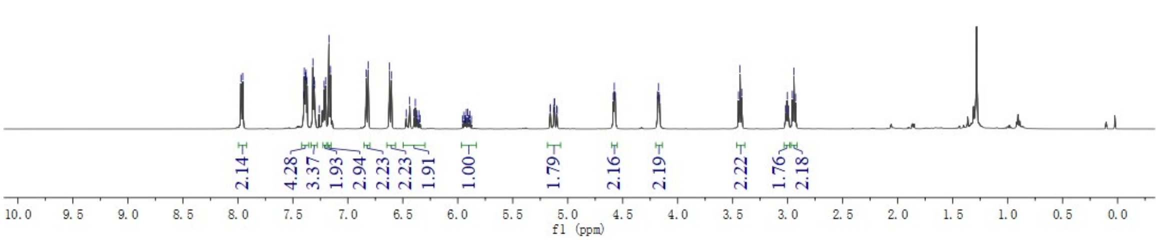

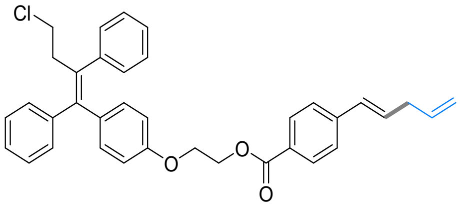

49

^{13}C NMR (126 MHz, $CDCl_3$)

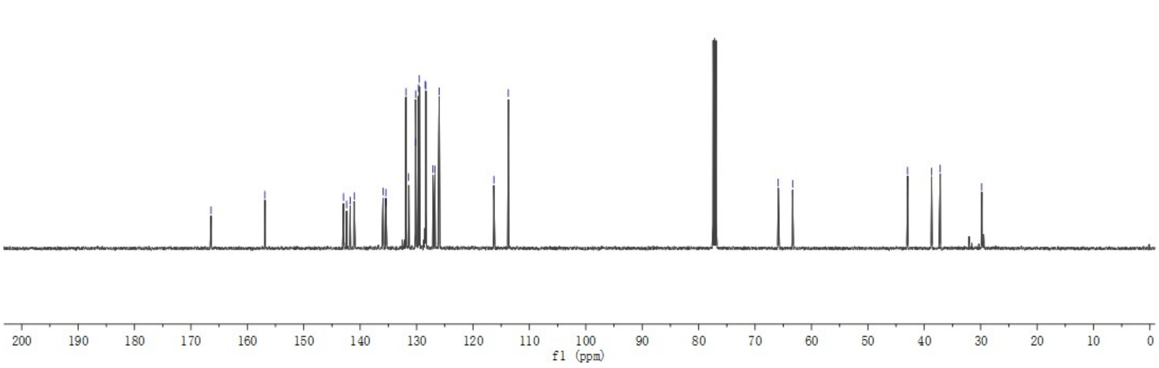

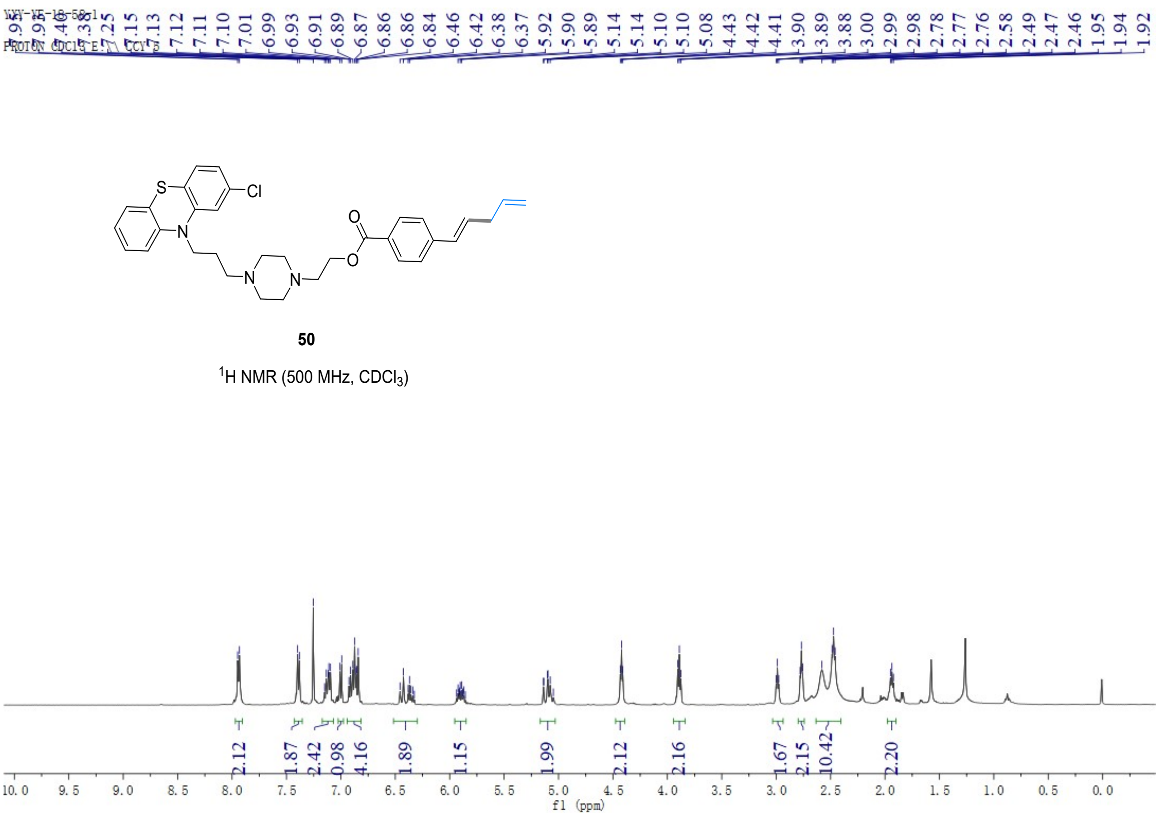

50

^{1}H NMR (500 MHz, $CDCl_3$)

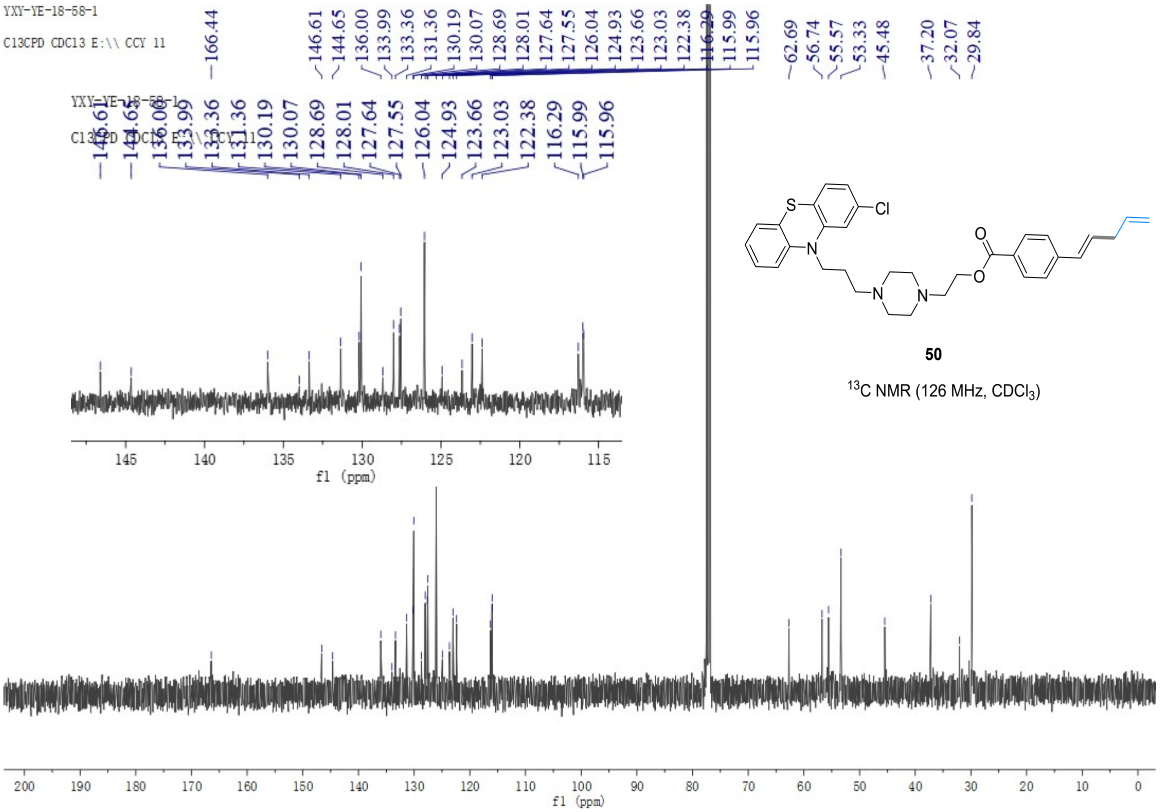

50

^{13}C NMR (126 MHz, $CDCl_3$)

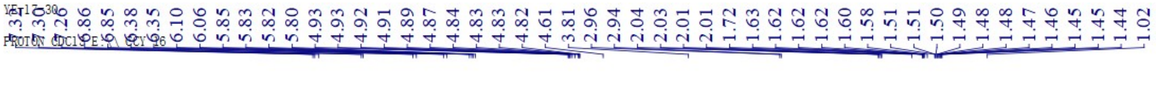

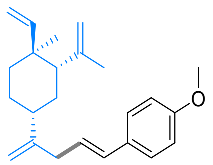

51

^{1}H NMR (500 MHz, $CDCl_3$)

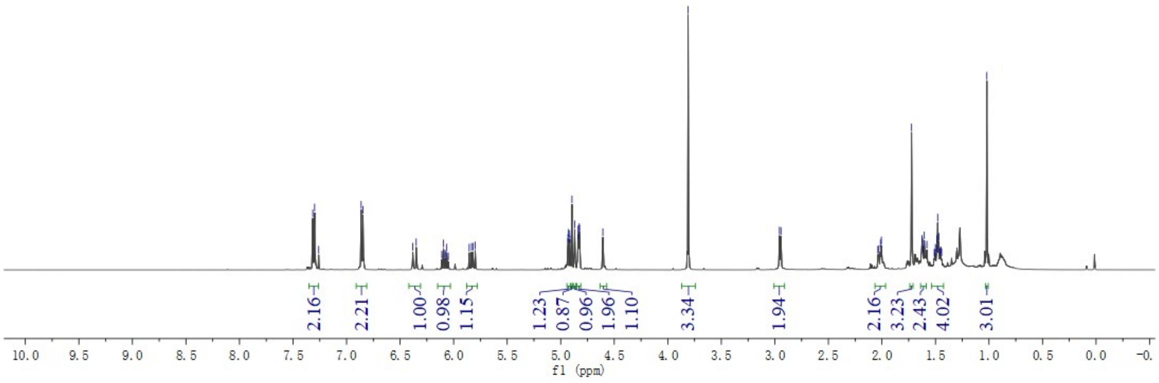

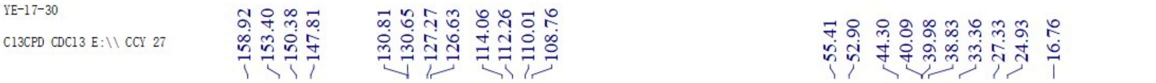

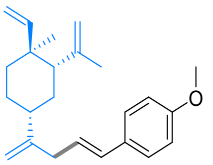

51

^{13}C NMR (126 MHz, $CDCl_3$)

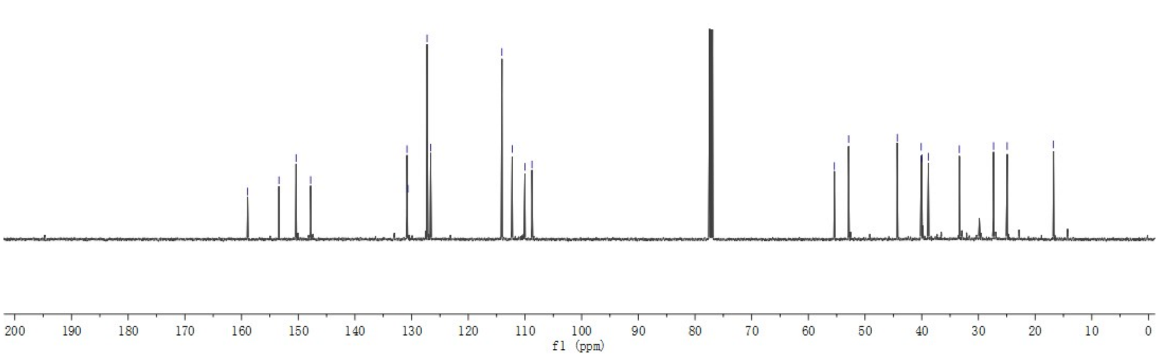

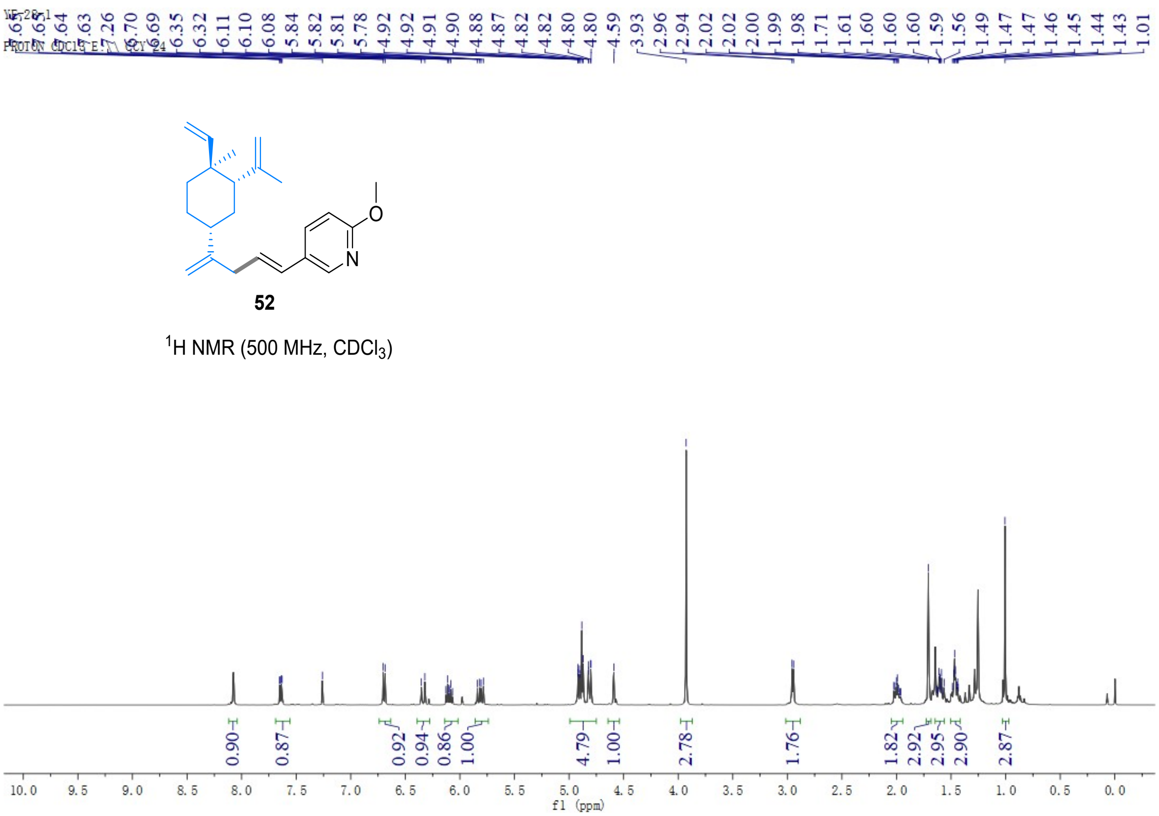

52

^{1}H NMR (500 MHz, $CDCl_3$)

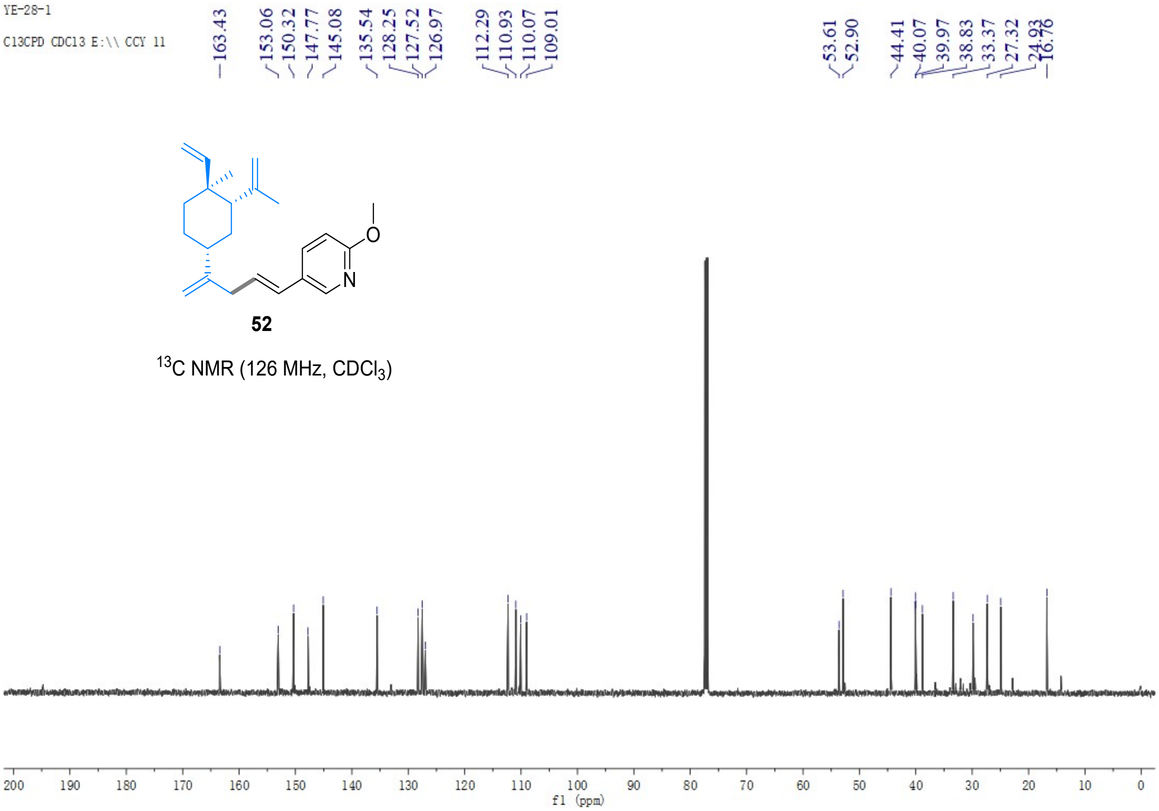

52

^{13}C NMR (126 MHz, $CDCl_3$)

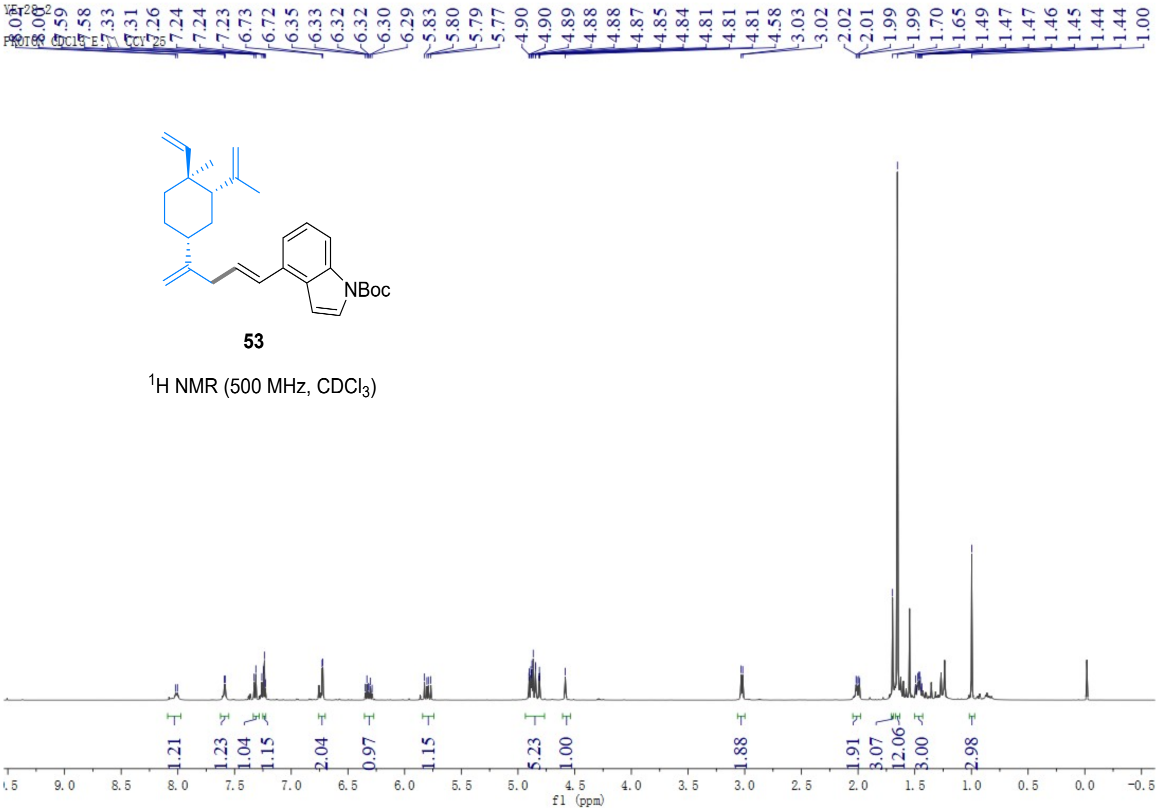

53

^{1}H NMR (500 MHz, $CDCl_3$)

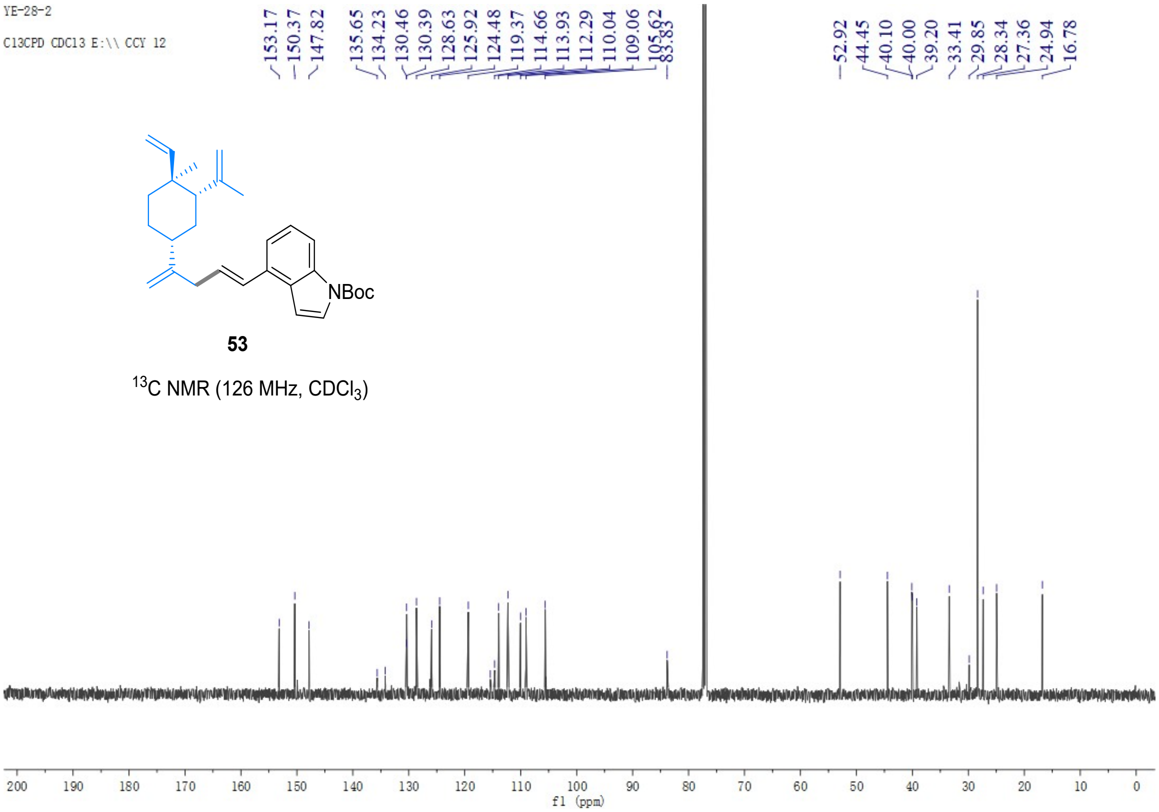

53

^{13}C NMR (126 MHz, $CDCl_3$)

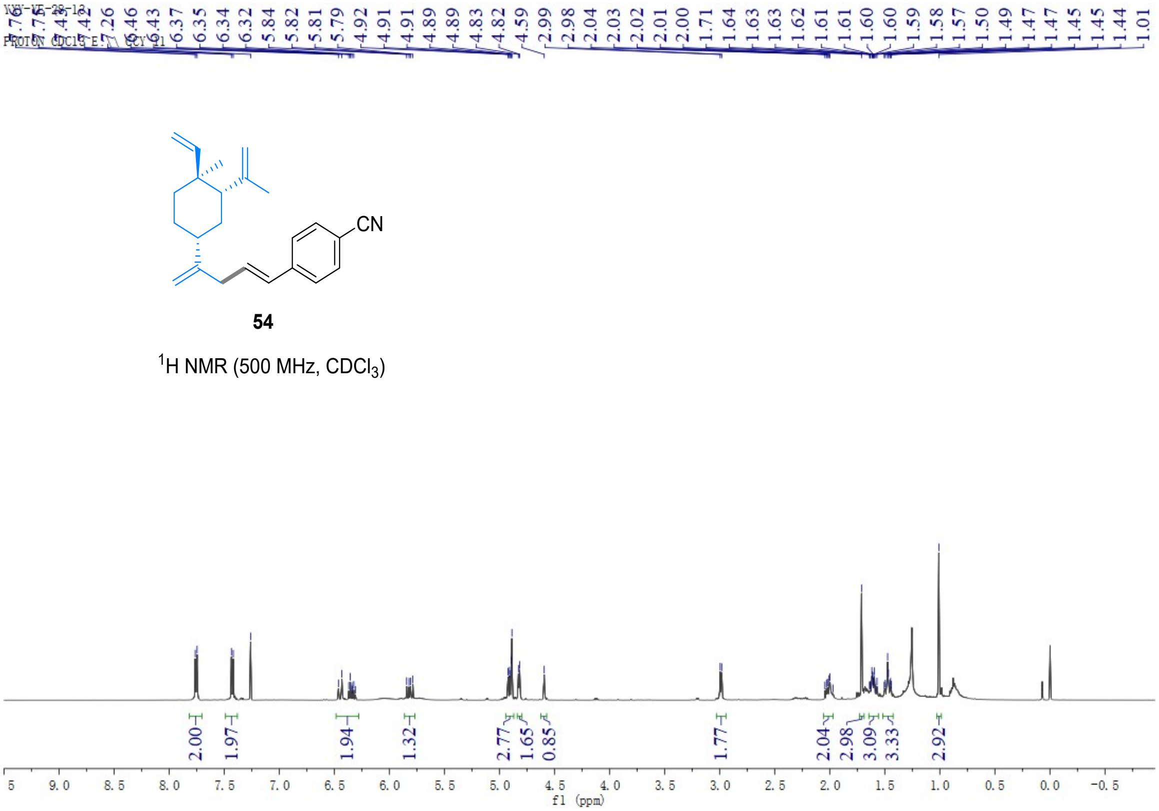
YXY-YE-28-13
PROTON CDCl3 E:\\ CCY 11
7.76 7.75 7.43 7.42 7.26 6.46 6.43 6.37 6.35 6.34 6.32 5.84 5.82 5.81 5.79 4.92 4.91 4.91 4.89 4.89 4.83 4.82 4.59 2.99 2.98 2.04 2.03 2.02 2.01 2.00 1.71 1.64 1.63 1.63 1.62 1.61 1.61 1.60 1.60 1.59 1.58 1.57 1.50 1.49 1.47 1.47 1.45 1.45 1.44 1.01
CN
54
1H NMR (500 MHz, CDCl3)
2.00 1.97 1.94 1.32 2.77 1.65 0.85 1.77 2.04 2.98 3.09 3.33 2.92
f1 (ppm)

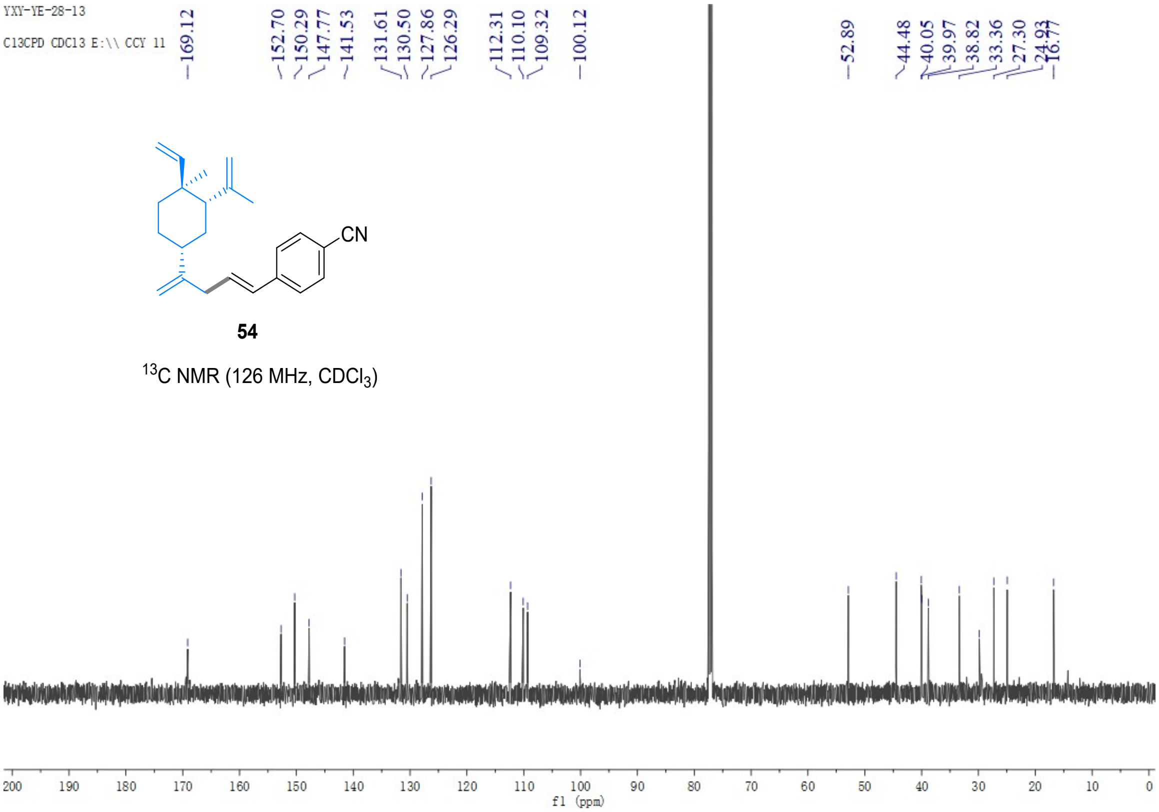
YXY-YE-28-13
C13CPD CDCl3 E:\\ CCY 11
169.12 152.70 150.29 147.77 141.53 131.61 130.50 127.86 126.29 112.31 110.10 109.32 100.12 52.89 44.48 40.05 39.97 38.82 33.36 27.30 24.93 16.77
CN
54
13C NMR (126 MHz, CDCl3)
f1 (ppm)

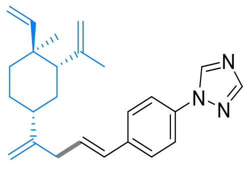

55

^{1}H NMR (500 MHz, $CDCl_3$)

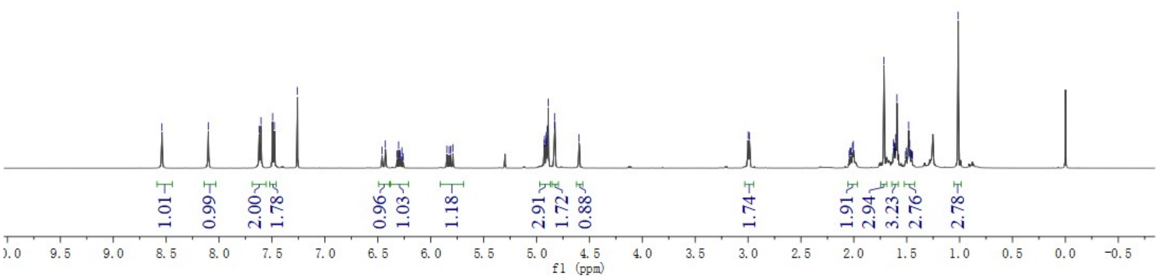

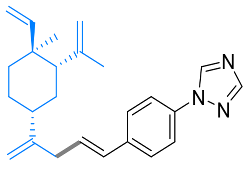

55

^{13}C NMR (126 MHz, $CDCl_3$)

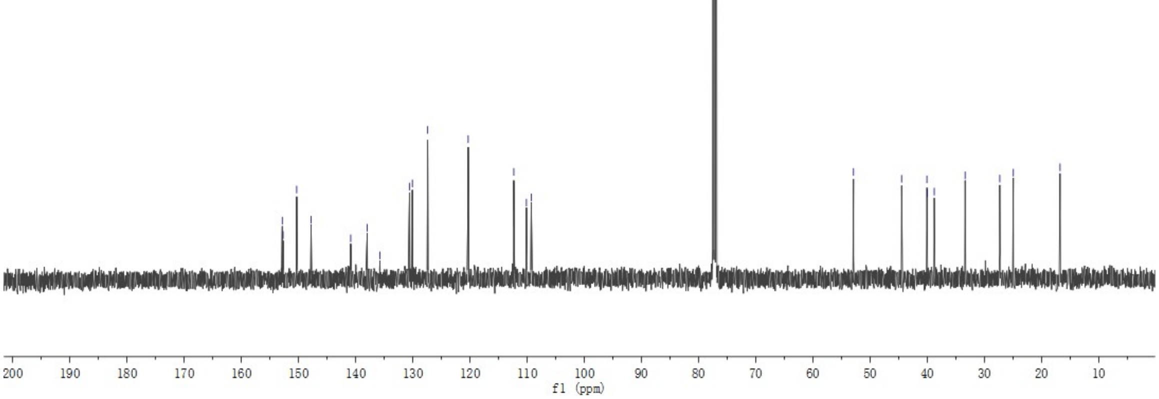

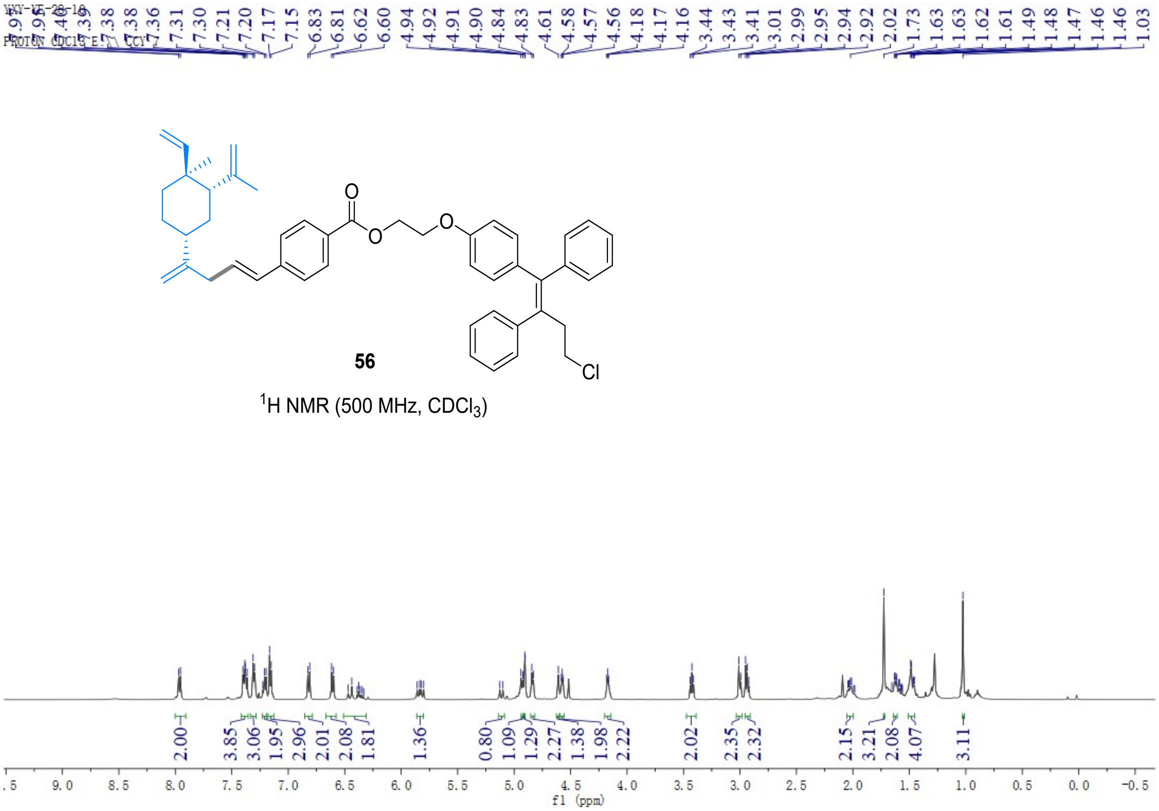

56

^{1}H NMR (500 MHz, $CDCl_3$)

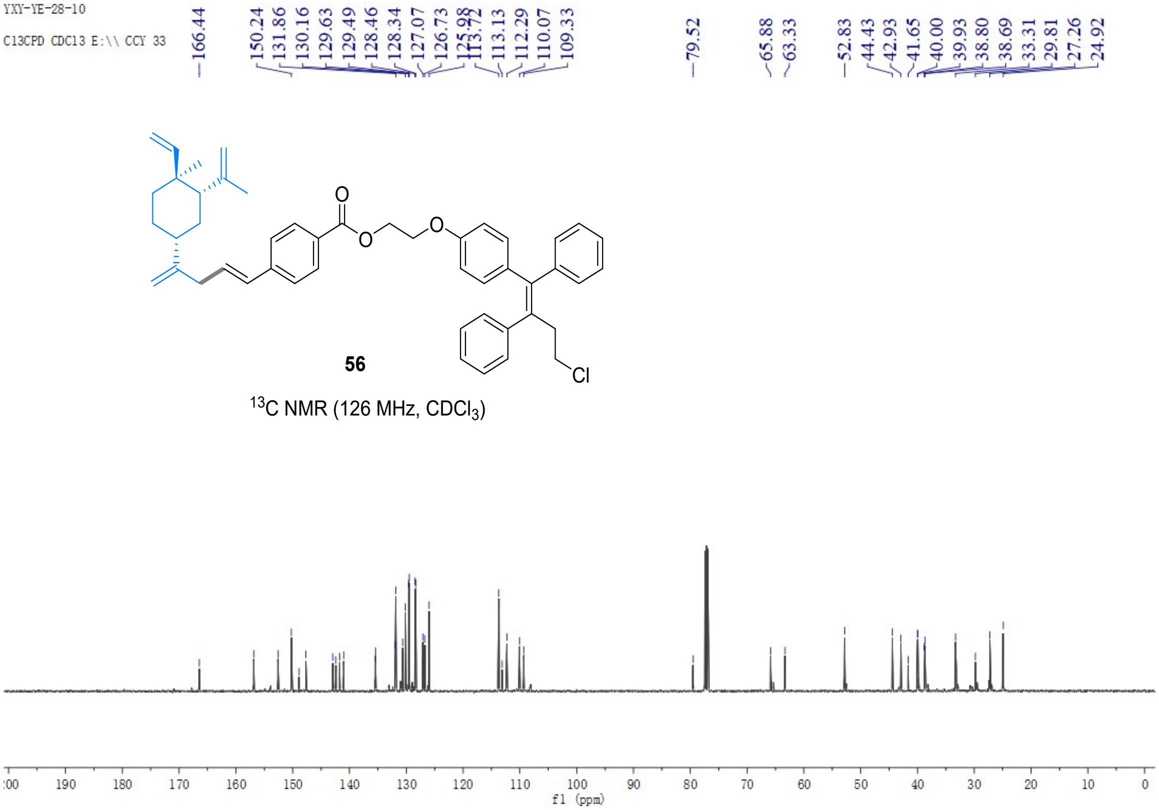

56

^{13}C NMR (126 MHz, $CDCl_3$)

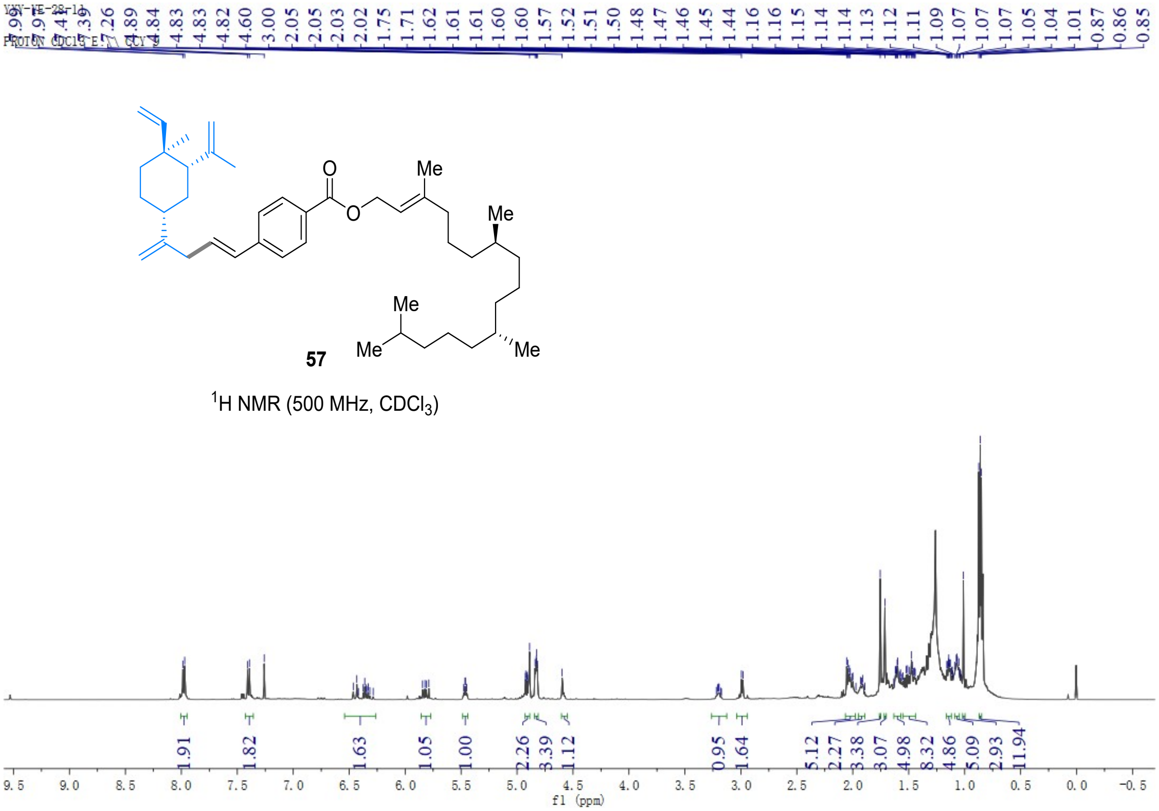

^{1}H NMR (500 MHz, $CDCl_3$)

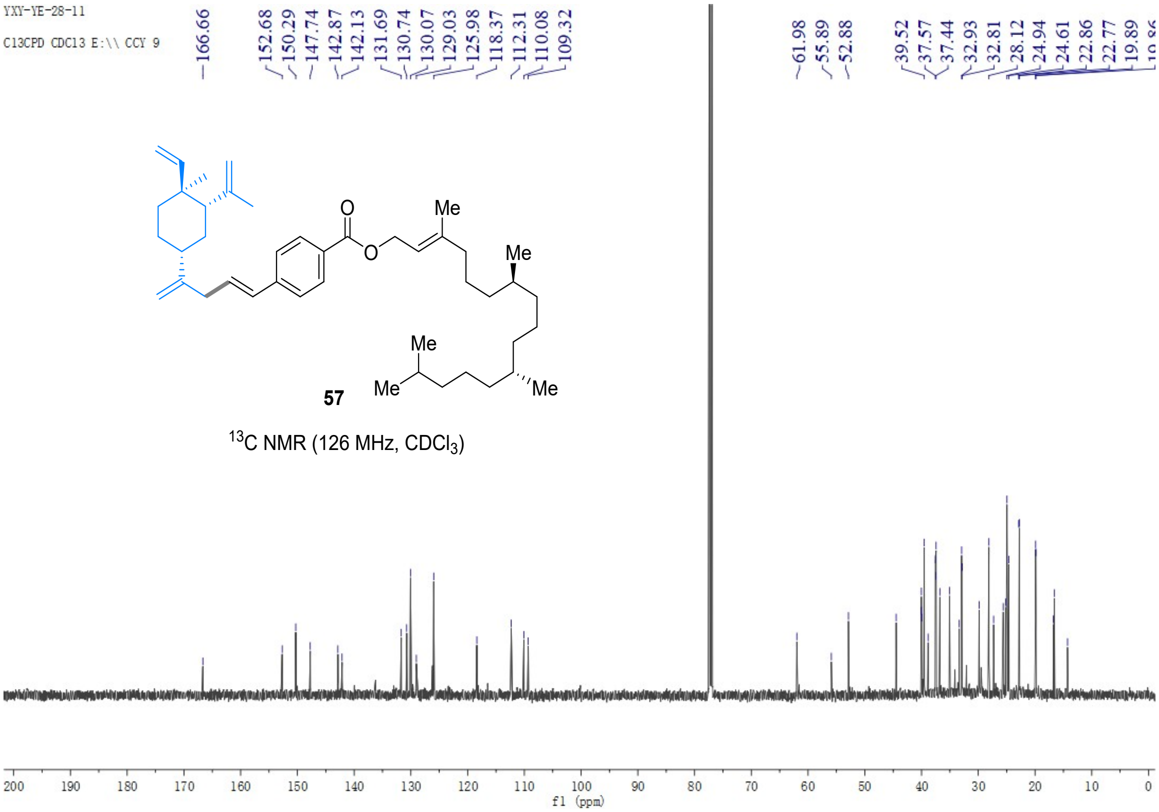

^{13}C NMR (126 MHz, $CDCl_3$)

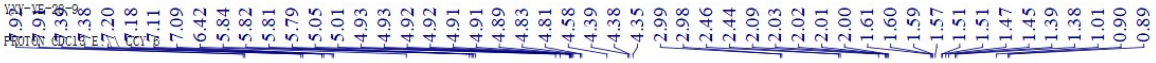

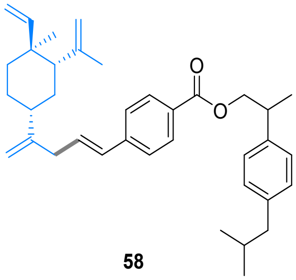

58

^{1}H NMR (500 MHz, $CDCl_3$)

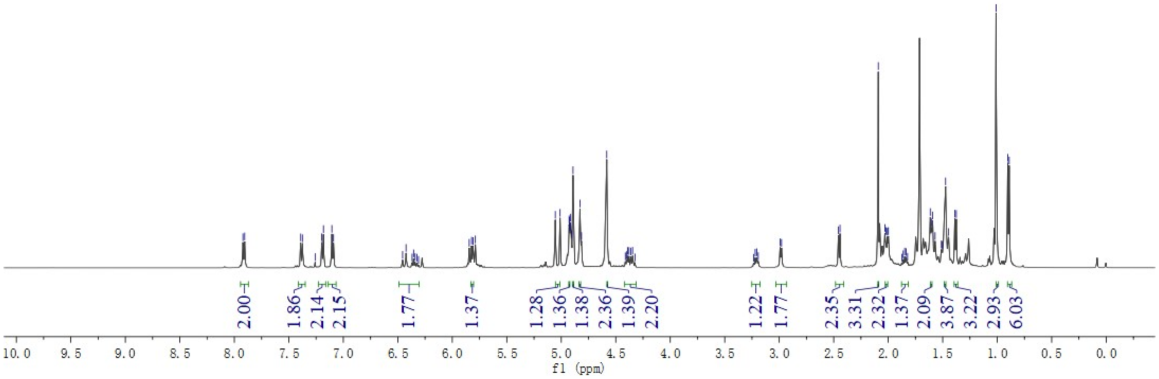

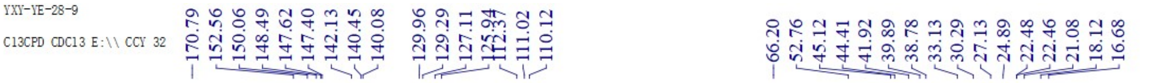

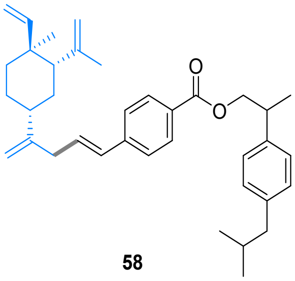

58

^{13}C NMR (126 MHz, $CDCl_3$)

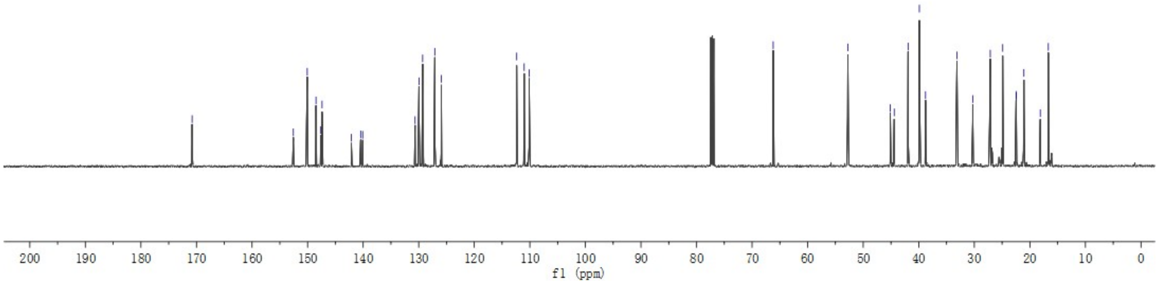

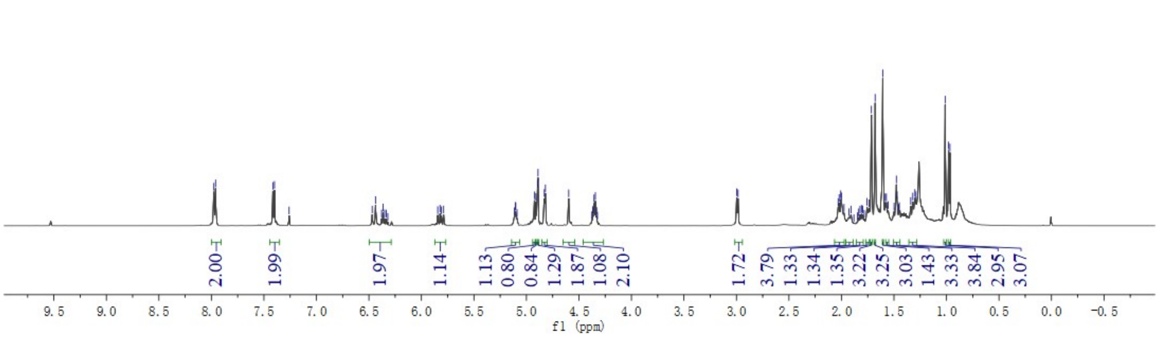

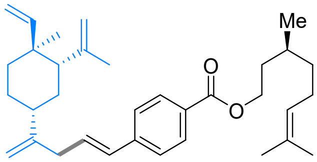

59

^{1}H NMR (500 MHz, $CDCl_3$)

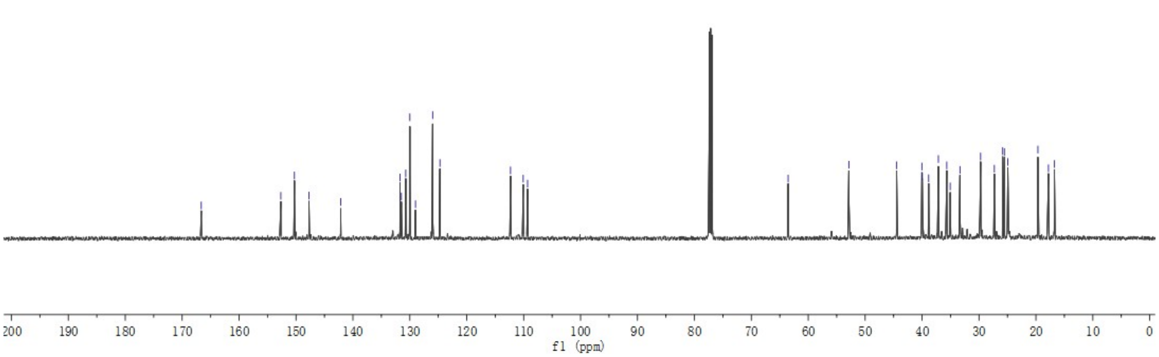

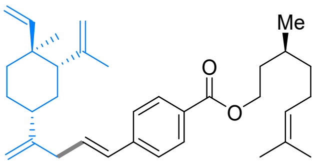

59

^{13}C NMR (126 MHz, $CDCl_3$)

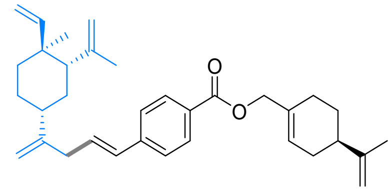

60

^{1}H NMR (500 MHz, $CDCl_3$)

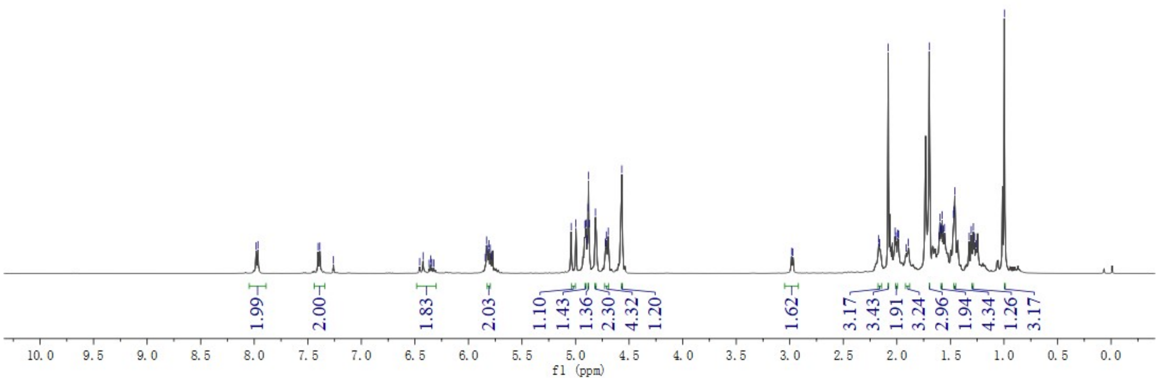

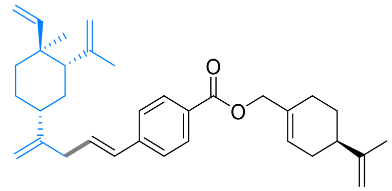

60

^{13}C NMR (126 MHz, $CDCl_3$)

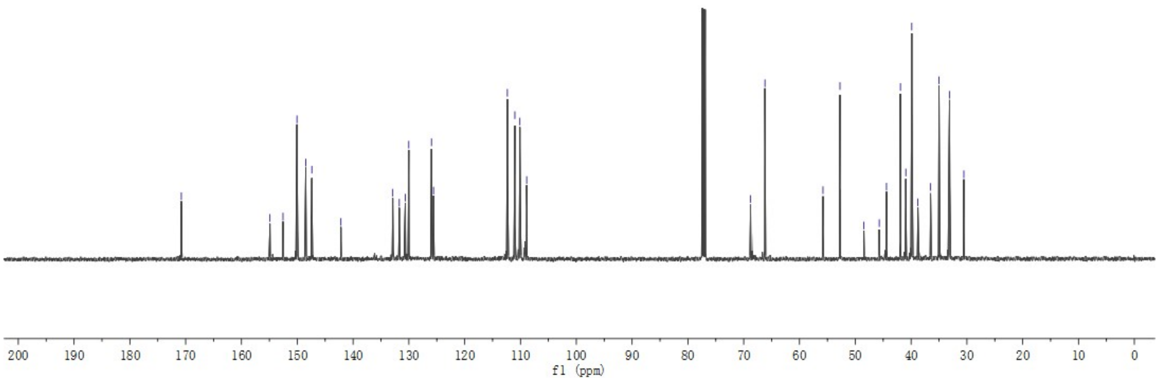

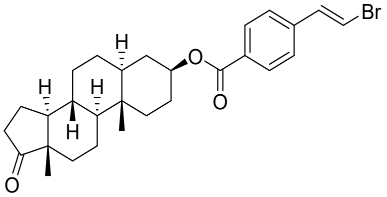

^{1}H NMR (500 MHz, $CDCl_3$)

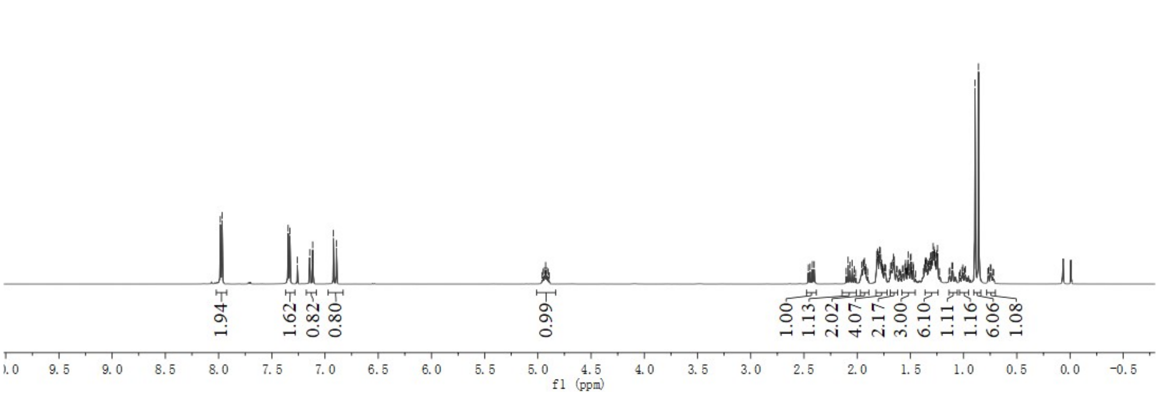

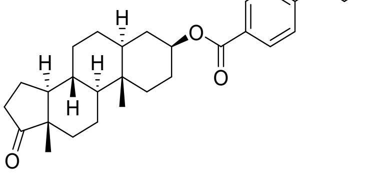

^{13}C NMR (126 MHz, $CDCl_3$)

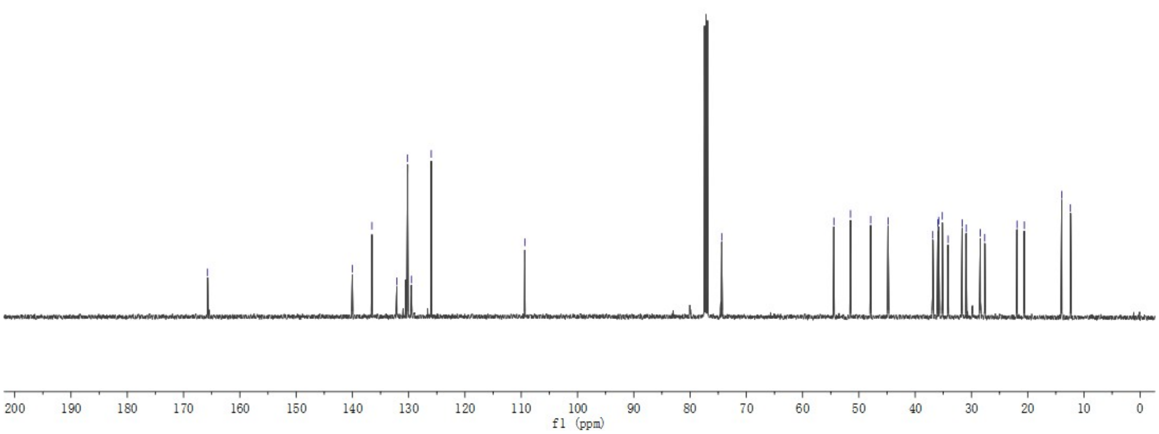

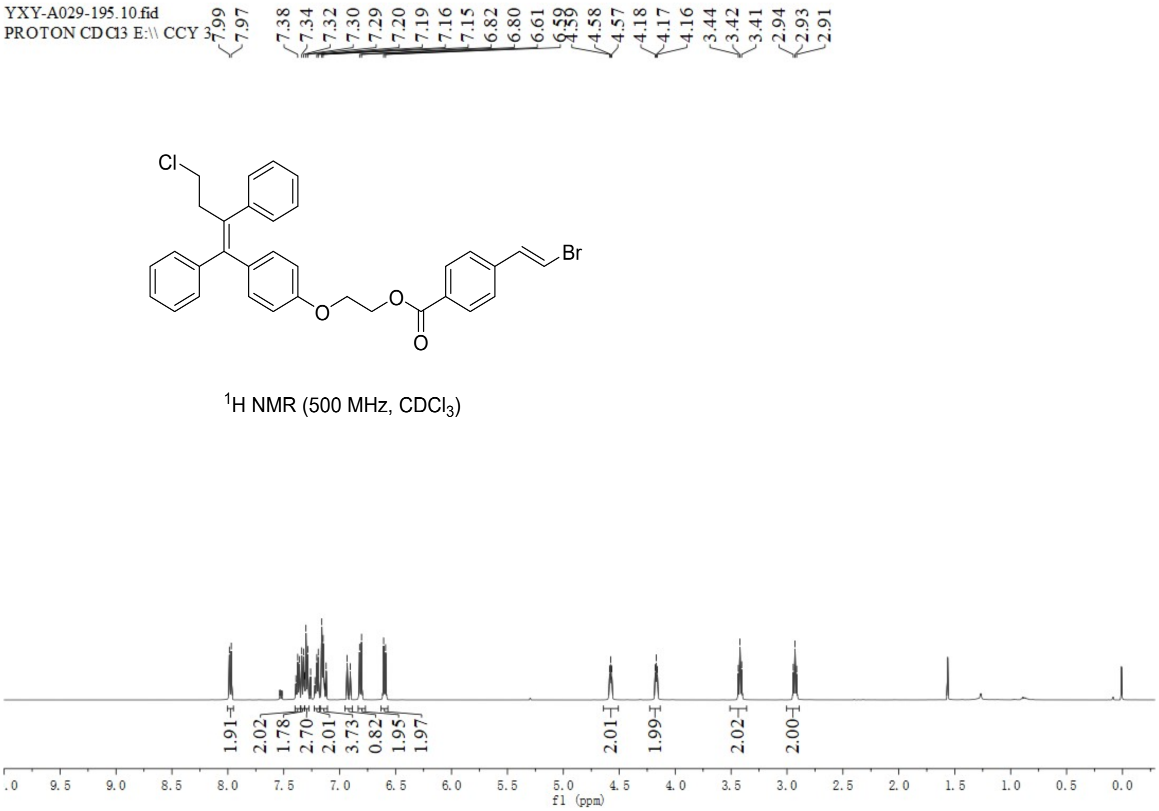

^{1}H NMR (500 MHz, $CDCl_3$)

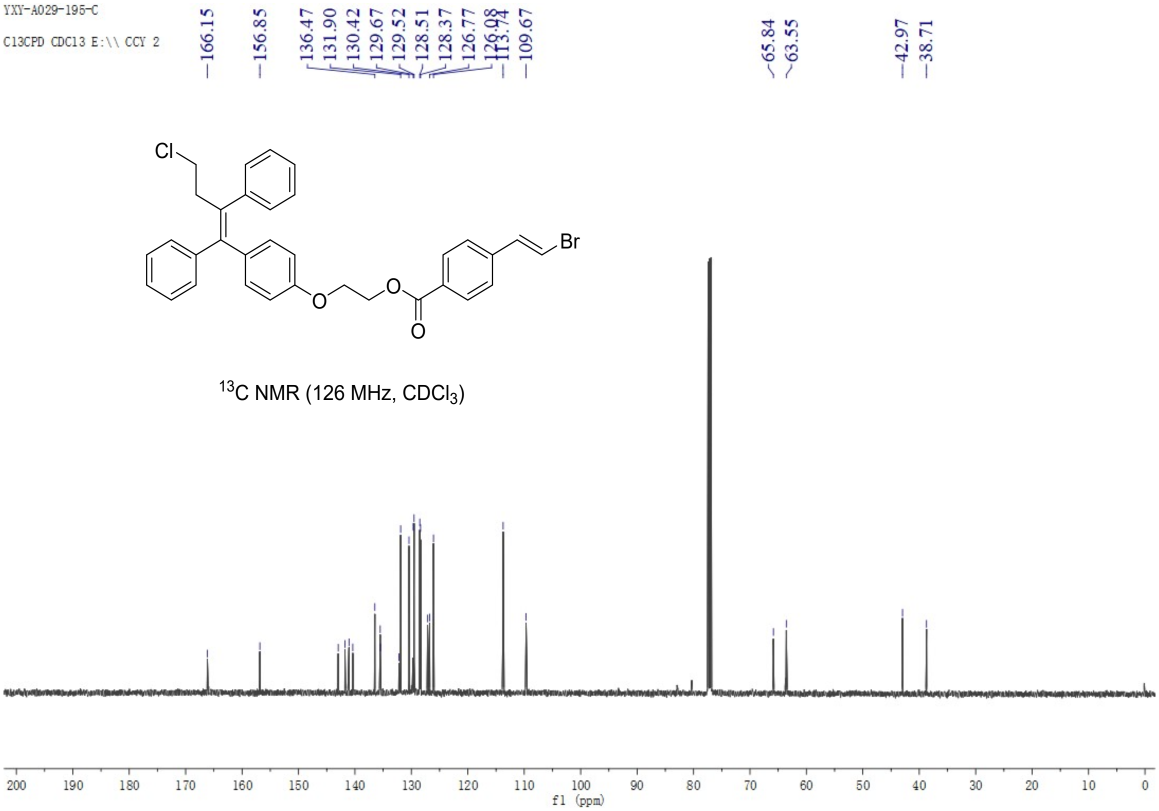

^{13}C NMR (126 MHz, $CDCl_3$)

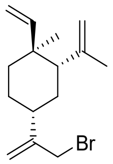

1c'

^{1}H NMR (400 MHz, $CDCl_3$)

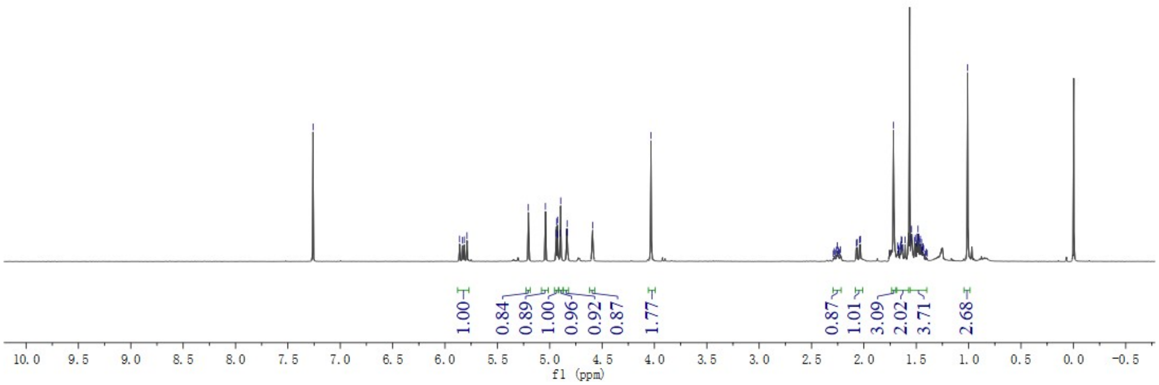

BZQSCL
13C

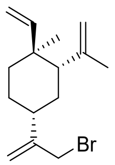

1c'

^{13}C NMR (101 MHz, $CDCl_3$)

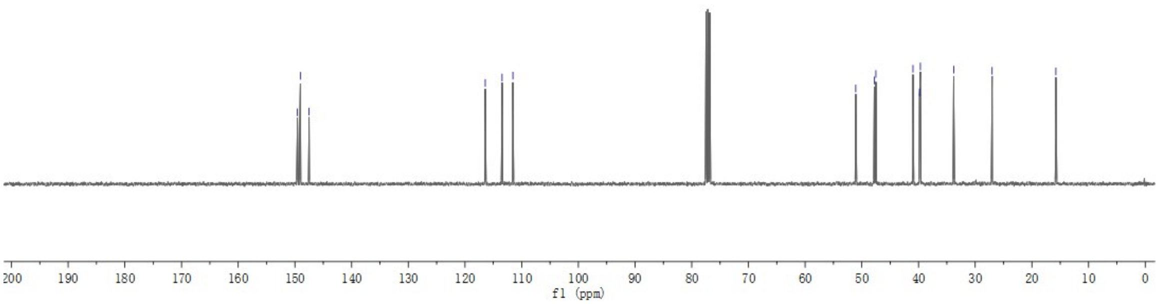

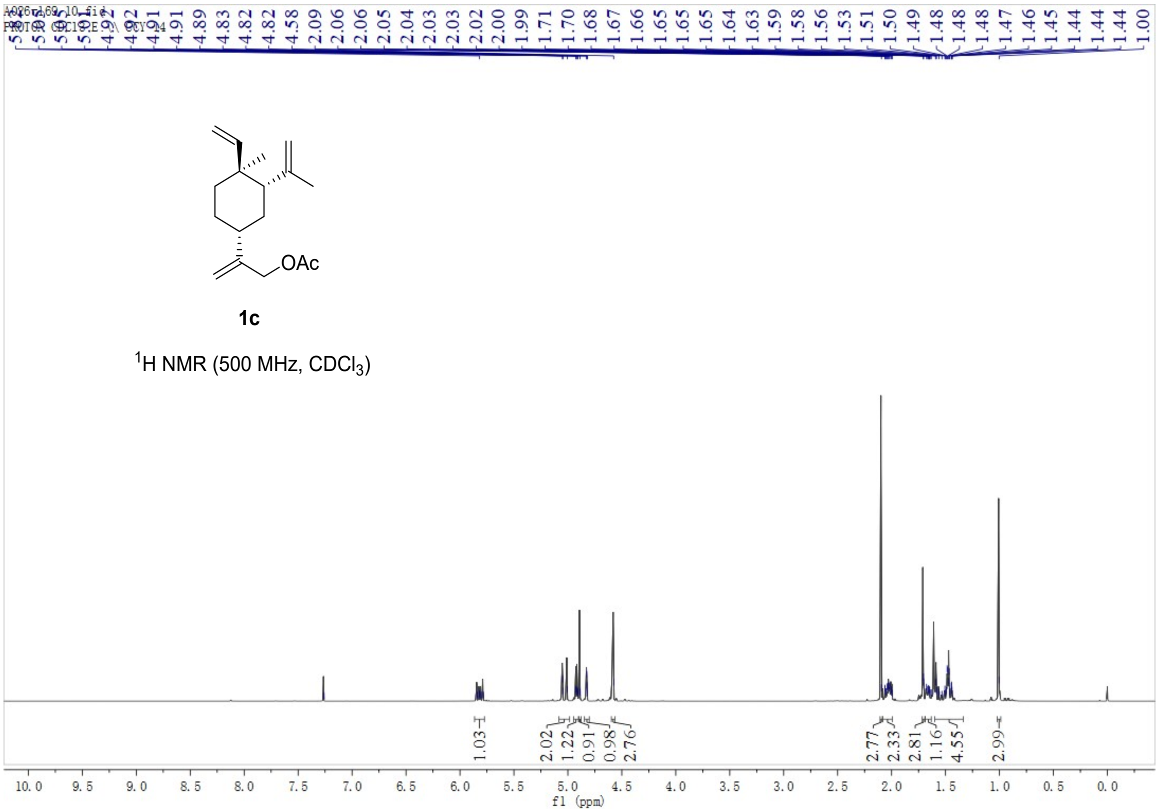

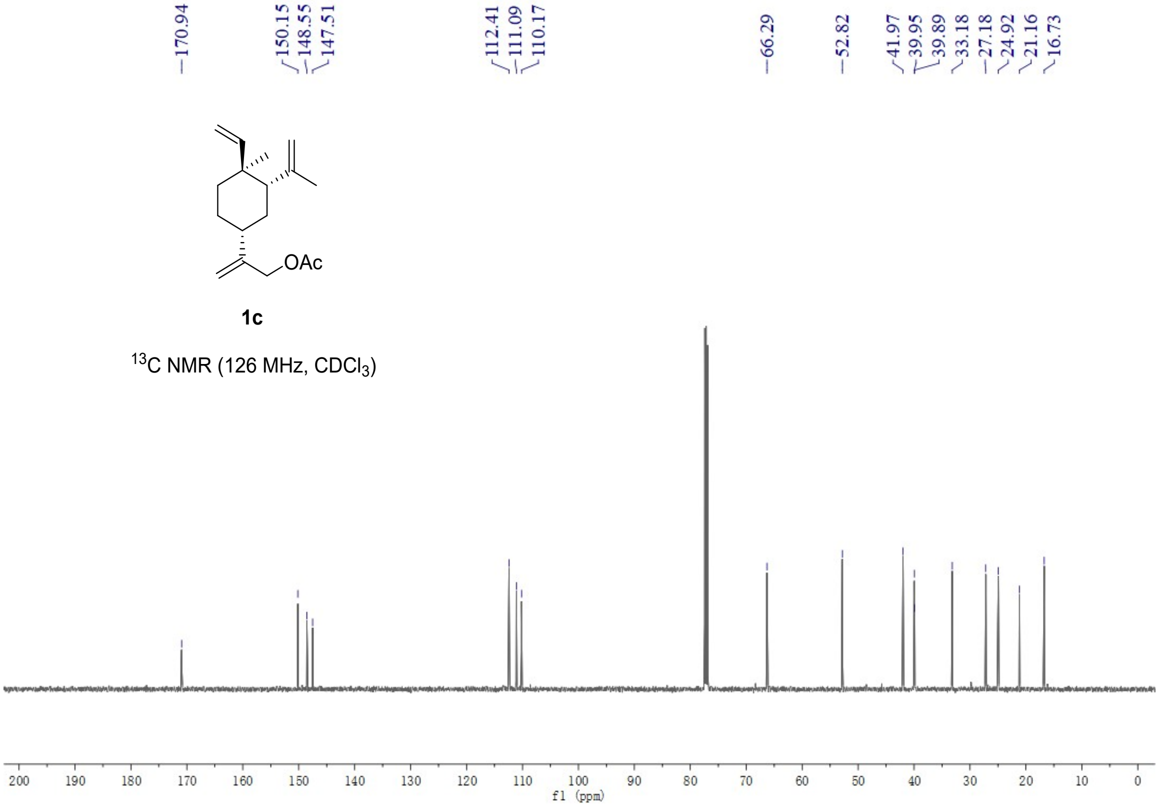

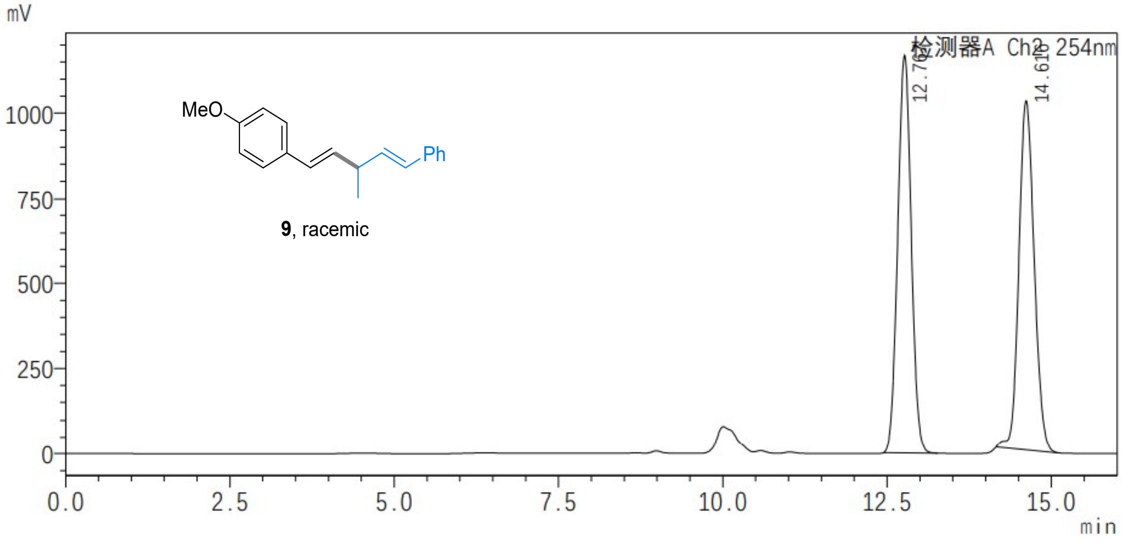

PDA Ch2 254 nm

Peak #	Resolution Time	Area	Height	Area %	Height %
1	12.767	16640490	1167181	50.043	53.255
2	14.616	16611791	1024494	49.957	46.745
Total		33252281	2191675	100.000	100.000

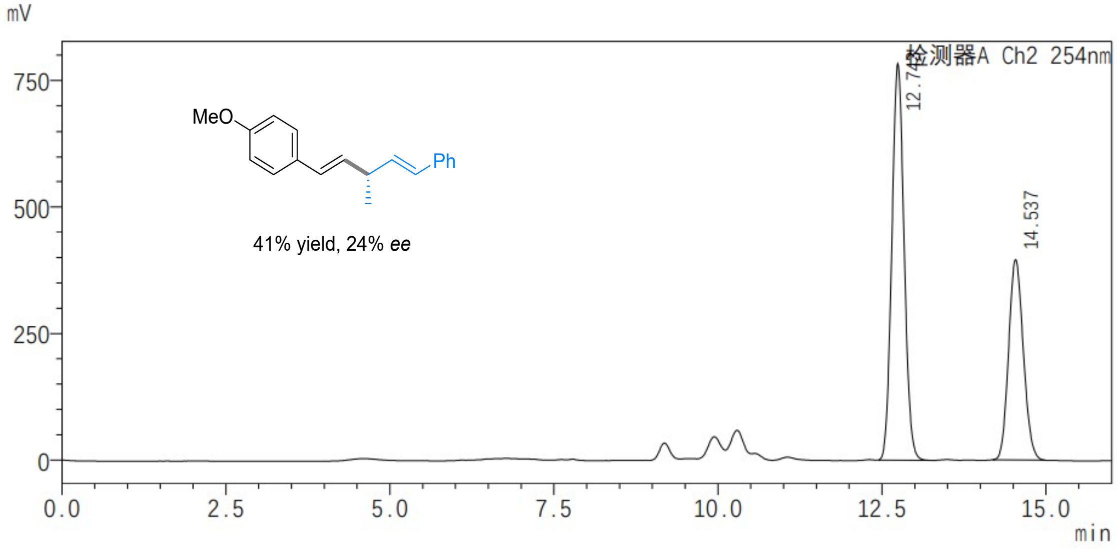

PDA Ch2 254 nm

Peak#	Resolution Time	Area	Height	Area %	Height %
1	12.743	10172598	783104	62.278	66.438
2	14.537	6161622	395598	37.722	33.562
Total		16334220	1178702	100.000	100.000
